# Direct-to-Biology Enabled Molecular Glue Discovery

**DOI:** 10.1021/jacs.5c13496

**Published:** 2025-12-24

**Authors:** Maowei Hu, Jason Ochoada, Marisa Actis, Kevin McGowan, Jamie A. Jarusiewicz, Satoshi Yoshimura, Logan McGrath, Uma Neelakantan, Anup Aggarwal, Anand Mayasundari, Sarah M. Young, Meng Zhang, Lei Yang, Yong Li, Shea Mercer, M. Madan Babu, Marcus Fischer, Brandon M. Young, Jun J. Yang, Gisele Nishiguchi, Anang A. Shelat, Daniel J. Blair

**Affiliations:** † Department of Chemical Biology and Therapeutics, 541707 St Jude Children’s Research Hospital, Memphis, Tennessee 38105, United States; ‡ Lead Discovery Informatics Center, Department of Chemical Biology and Therapeutics, St Jude Children’s Research Hospital, Memphis, Tennessee 38105, United States; § Targeted Protein Degradation Center, Department of Chemical Biology and Therapeutics, St Jude Children’s Research Hospital, Memphis, Tennessee 38015, United States; ∥ Medicinal Chemistry Center, Department of Chemical Biology and Therapeutics, St Jude Children’s Research Hospital, Memphis, Tennessee 38105, United States; ⊥ Department of Pharmacy and Pharmaceutical Sciences, St Jude Children’s Research Hospital, Memphis, Tennessee 38105, United States; # Center of Excellence for Data Driven Discovery, Department of Structural Biology, St Jude Children’s Research Hospital, Memphis, Tennessee 38105, United States; g Analytical Technologies Center, Department of Chemical Biology and Therapeutics, St Jude Children’s Research Hospital, Memphis, Tennessee 38105, United States; h Program Management, Department of Chemical Biology and Therapeutics, St Jude Children’s Research Hospital, Memphis, Tennessee 38015, United States

## Abstract

Molecular glues powerfully control protein proximity but have largely eluded direct screening. A promising avenue for addressing this challenge lies within pinpointing the fundamental features for function-first identification of molecular gluing events. In the widely accepted mechanism, a molecular glue stabilizes two proteins within a ternary complex—here, we show how differences in affinity for ternary and binary complexes directly categorize glues from nonglues. We leverage these differences together with high-throughput chemical synthesis and affinity-selection mass-spectrometry to discover a molecular glue from a suite of over 20,000 crude chemical reaction mixtures. Orthogonal assays robustly support the identification of molecular glues via ternary complex stability. Our findings suggest that a roadmap for *de novo* molecular glue discovery lies within kinetic profiling of unpurified mixtures of small organic molecules against protein pairs.

## Graphical abstract

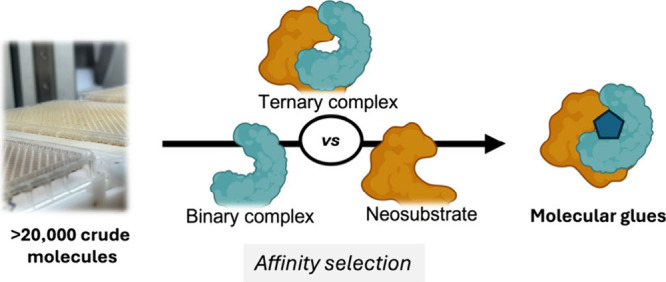

Chemically induced proximity has established biomolecular redistribution as a viable therapeutic approach.^1–4^ Unlike inhibitor-centric strategies, chemically induced proximity exerts its biological effects by relocating biomolecules to alter function, historically exemplified by rapamycin-induced protein dimerization.^5^ Small molecules which induce proximity fall into two distinct categories: bifunctional molecules containing ligands for two separate biomolecules joined by a linker and monofunctional molecules, which bind a single biomolecular partner and stabilize its interaction with another biomolecule. These monofunctional molecules are commonly known as molecular glues (Figure 1a).

**Figure 1:**
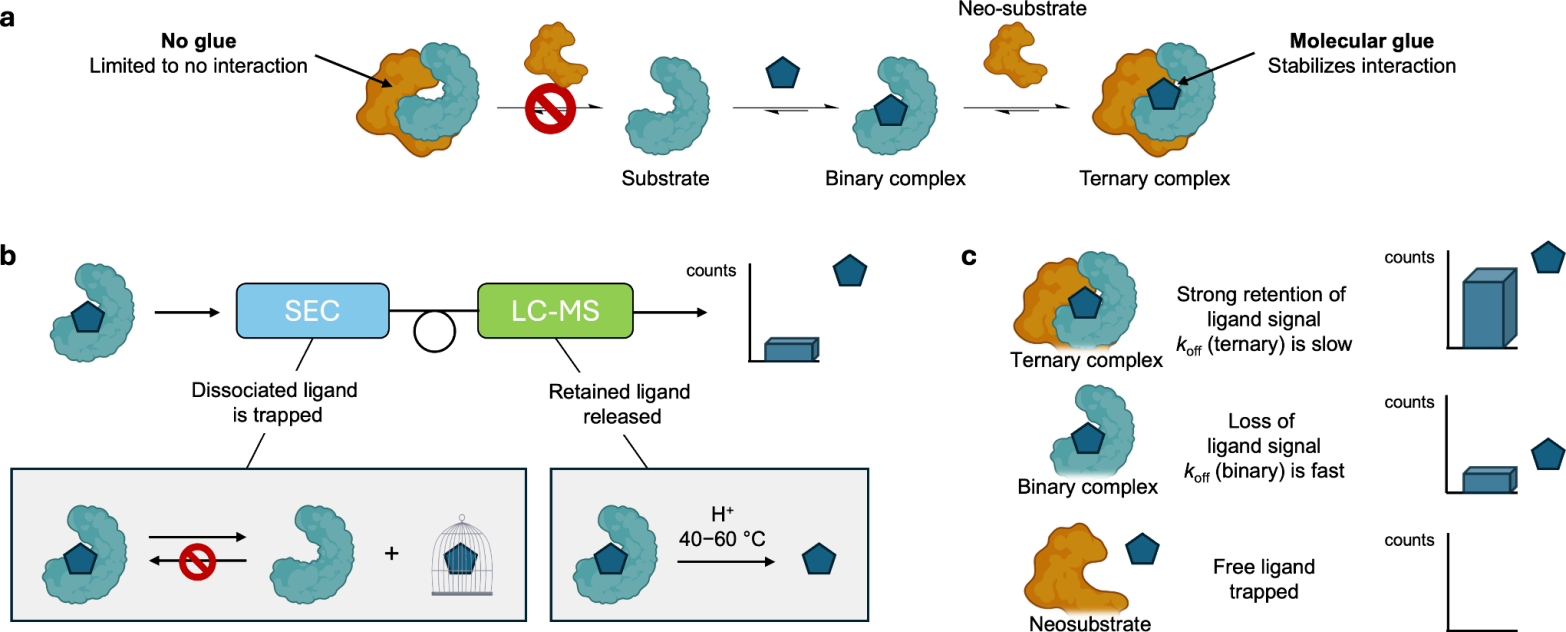
Dissociation kinetics drives affinity enrichment, identifying molecular glues through ternary complex stabilization. (a) Molecular glues exert a stabilizing effect between protein pairs and enforce proximity. (b) Affinity selection mass spectrometry (ASMS) involving hyphenated SEC and LC-MS to exploit differences in protein–ligand dissociation kinetics to distinguish molecular glues from conventional ligands. Pools of small molecules are incubated with a candidate protein and injected onto an SEC column. Here, weakly or unbound ligands are trapped by the SEC column; the remaining protein–ligand complex is redirected to an LCMS system which denatures the protein–ligand complex for detection by high-resolution mass spectrometry. (c) A ligand which behaves as a molecular glue experiences attenuated dissociation kinetics from a ternary complex compared to either binary complex or neosubstrate. Such differences would manifest an increase in ligand signal by ASMS for the ternary complex relative to binary complex and neosubstrate data.

Chemically induced proximity has garnered significant interest in drug discovery, primarily through advances in bifunctional Proteolysis Targeting Chimeras (PROTACs),^6–8^ which recruit candidate proteins to E3 ligases for targeted degradation.^9,10^ While numerous examples demonstrate the powerful potential of proximity induction to initiate degradation of target proteins through proximity to E3 ligases,^11^ current discovery technologies have predominantly focused on directly degradative outcomes,^12,13^ facilitated in many ways by PROTACs suitability for conventional ligand–receptor screening strategies.^14^

Unlike their bifunctional counterparts monofunctional molecular glues^15^ have been more challenging to identify. Current strategies depend on prior knowledge of phenotypes (such as degradation),^16,17^ or mandate the use of linkers,^18^ underscoring the need for additional advances which link core molecular glue properties with candidate protein pairs. Challenges notwithstanding, a series of molecular glues have progressed into clinical settings,^11^ highlighting their therapeutic utility. Yet, intentionally identifying molecular glues remains largely serendipitous.^19^

A promising avenue to discover molecular glues would be to adopt direct-to-biology strategies, which bypass the purification of small molecules and directly screen for proximity *in vitro*. Recent advances in high-throughput chemical synthesis have facilitated such methods by reducing the opportunity cost of chemical analoging.^20^ These approaches have been successfully employed in developing PROTACs,^21,22^ covalent ligands,^23^ and noncovalent ligands.^24,25^

The requirements for identifying a molecular glue are distinct from those of inhibitor and ligand/receptor centric approaches. Adopting a direct-to-biology method necessitates a generalized feature which can functionally distinguish molecular glues from nonglues. To achieve this, we considered leveraging ligand dissociation kinetics as a fundamental differentiator (Figure 1b,c). Specifically, molecular glues could be uniquely identified by their slower dissociation rates from ternary complexes compared with binary complexes or neosubstrates alone (Figure 1c). This kinetic signature might be efficiently identified using affinity-driven methods such as Affinity Selection Mass Spectrometry (ASMS)^24,26,27^ (Figure 1b) and related techniques,^28^ which enable high-throughput ligand prioritization based on differential dissociation rates (*k*_off_). While affinity-selection methods have thus far been applied primarily to single biomolecular targets,^26^ extending their use to maintain protein–protein interactions throughout the ASMS lifecycle could offer a powerful approach for systematically discovering molecular glues.

To establish the foundational principles for using ASMS in the discovery of molecular glues, we selected the cereblon–neosubstrate protein–protein interface as a test platform because it would provide robust downstream assays for method validation. Here we report how, by searching through crude mixtures of small molecules, we can identify novel small molecules that act as molecular glues for protein pairs.

To test our hypothesis that ternary complex dissociation kinetics could serve as a readout for molecular glues by affinity-selection mass spectrometry, we chose a model system with an established glueable pocket. Specifically, we selected the CRBN–GSPT1 protein pair by virtue of its validated molecular glue SJ6986 ^29^ (Figure 2a).

**Figure 2:**
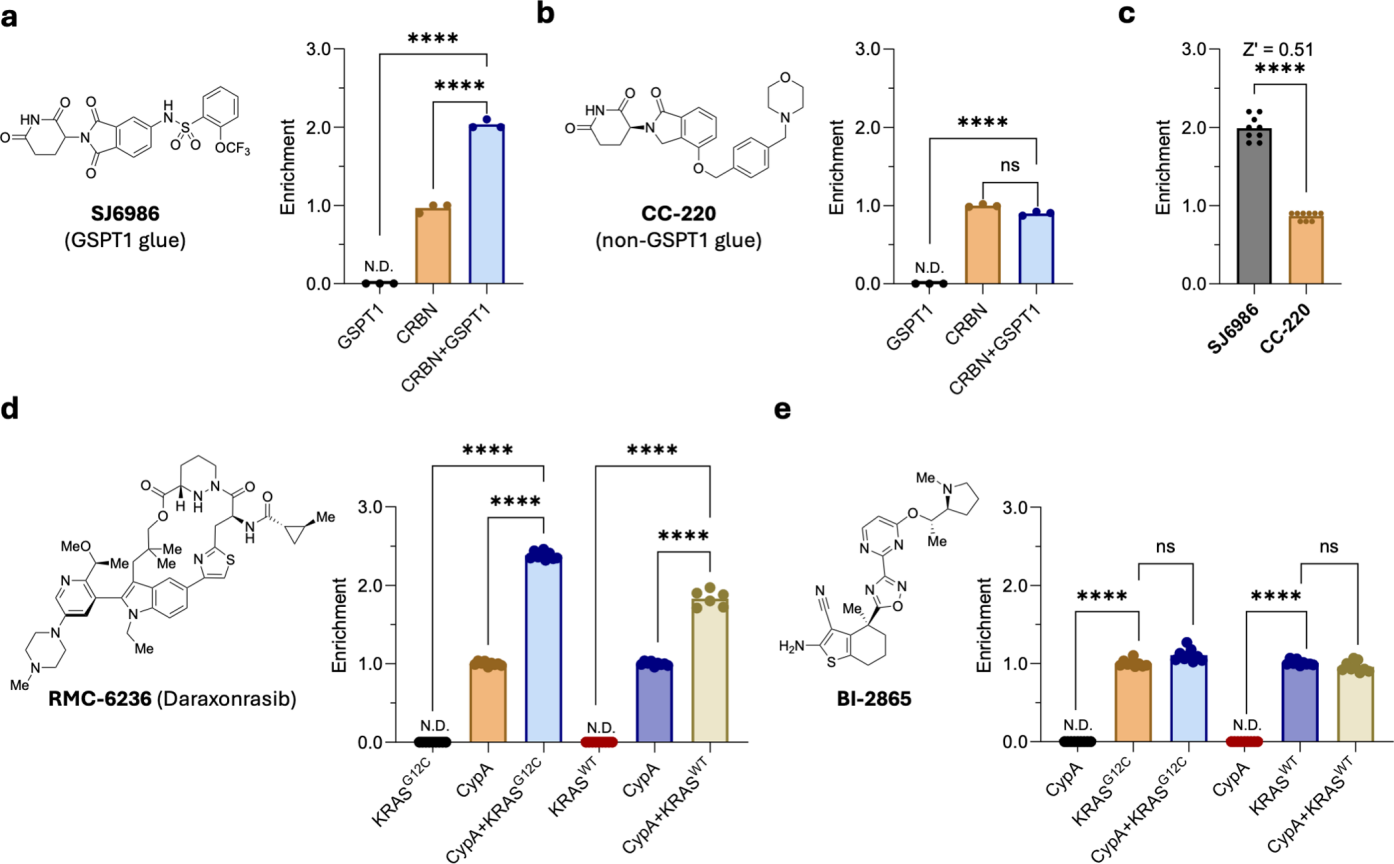
Protein–protein interactions enrich ligand signals by ASMS. (a) Molecular glue SJ6986 exhibits ternary complex mediated signal enrichment by ASMS upon addition of GSPT1. (b) Non-GSPT1 glue CC-220 shows no enrichment upon addition of GSPT1. (c) Reproducibility of enrichment by ASMS was sufficient to discern glues from nonglues. (d) Cyclophilin A (CypA) binding molecular glue daraxonrasib was enriched by ASMS upon addition of wildtype KRAS or its G12C mutant. (e) Noncovalent pan-RAS binder BI-2865 was not enriched by addition of cyclophilin A.

At the outset, it was unclear whether the conditions associated with ASMS would compromise ternary complex stability and eliminate any kinetic dissociation biases permissive of molecular glue identification. Direct comparison of known CRBN–GSPT1 glue SJ6986 against CRBN, GSPT1, and CRBN–GSPT1 by ASMS indicated that relative to CRBN there was a 2-fold enrichment of SJ6986 ligand signal when exposed to CRBN–GSPT1 ternary complex (Figure 2a). These data allayed concerns surrounding ternary complex stability during ASMS. Ligand identity should underlie ternary complex mediated enrichment, so we utilized the known cereblon E3 ligase modulatory drug CC-220 ^30^ which is devoid of GSPT1-glue activity. Accordingly, CC-220 was not enriched by CRBN–GSPT1 relative to CRBN (Figure 2b). Titration of GSPT1 into CRBN–SJ6986 showed a concentration dependent increase in ligand signal by ASMS, validating that enrichment was a ternary complex driven event (Supplementary Figure 1a). Again, showing that ternary complexes drive ligand signal enrichment by ASMS, we did not find CC-220 enriched upon titration of GSPT1. The reproducibility of these experiments provided excellent ability to distinguish glue from nonglue (Figure 2C, *Z*′ = 0.51), and the interference from nonparticipating proteins was low (Supplementary Figure 1f).

In the knowledge that we planned to utilize crude unpurified small molecules to scout for novel molecular glues, a logical confounding feature would be unreacted chemical building blocks arising from synthesis. Such impurities might interfere with ternary complex mediated enrichment. To test this, we added ∼100 drug-like decoys and obtained comparable enrichment for SJ6986 (Supplementary Figure 1b–e).

Further validation of the utility of molecular glue identification by affinity-selection mass spectrometry in nondegradative contexts was sought using the cyclophilin A/KRAS protein pair and the cyclophilin A binding molecular glue daraxonrasib (RMC-6236).^31^ Consistent with ternary-complex mediated enrichment addition of either KRAS^G12C^ or KRAS^WT^ to cyclophilin A bound daraxonrasib led to 2.4- and 1.9-fold enrichment, respectively (Figure 2d). As a control for this process the noncovalent KRAS^G12C^ binder BI-2865 ^32^ was not enriched through addition of cyclophilin A (Figure 2e). These data were again found to be highly reproducible with an excellent *Z*′ score (0.75 and 0.76, Supplementary Figure 1g,h).

To pressure test our affinity-selection mass spectrometry method for identifying molecular glues we needed to prepare a large library of molecules in a crude unpurified format.^33^ We recently reported that common lost chemical fragments could drive rapid analysis of chemical reaction outcomes by tandem mass spectrometry in a process we call fragmentation-first experimentation.^34^ This strategy should maximize overall access to a library of molecular glue derivatives by enabling rapid profiling of reaction conditions across chemical substrates to select the best performers (Figure 3).

**Figure 3:**
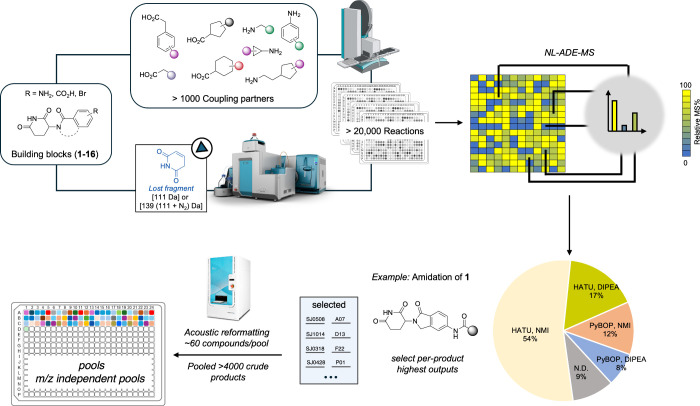
Nanoscale synthesis of a library of molecular glues via fragmentation-first experimentation. Chemical building blocks featuring known cereblon (CRBN) binding motifs were elaborated in high-throughput miniaturized formats to generate >20,000 reaction mixtures. MS/MS analysis profiles could be determined directly from the starting materials to inform analysis of reaction mixtures spanning different reaction conditions, thereby allowing the selection of the highest relative performers. Identified best performing reactions were pooled via acoustic liquid handling to generate 70 pools of 60 compounds with a minimum mass separation of 0.050 Da.

With the goal of constructing a library of >5000 molecular glue derivatives we selected 16 chemical building blocks featuring the key glutarimide motif known to bind to CRBN.^35^ To maximize coverage of local fragment-vector space, each of these 16 building blocks featured unique exit vectors and chemical handles primed for diversification by Buchwald–Hartwig coupling or amide bond formation. Aligned with this workflow we performed MS/MS analysis of the 16 building blocks which pinpointed two key fragmentation patterns (Supplementary Figure 2) which would enable rapid analysis.

Armed with a set of MS/MS barcoded chemical building blocks, we assembled libraries of amines and carboxylic acids which interface with each connecting point on the 16 CRBN-binding building blocks (Supplementary Figures 3–5). Using automated liquid handling we created 4 μL scale reaction mixtures across 4 conditions per target molecule to yield ∼20,000 reactions. The neutral lost fragments associated with each parent building block allowed rapid analysis via acoustic droplet ejection mass spectrometry at a pace of 1.2 s/sample to determine the outcomes of all 20,000 reaction mixtures in just 13 h. From these data, we were able to select the best performing condition sets on a per product basis for downstream study (Figure 3, Supplementary Figures 3–5). To enable direct screening of the resulting chemical library, we developed a pooling algorithm which selected unique masses based on a resolution threshold; for the purpose of this study, we set that to 0.050 Da to generate entirely mass unique pools where their exact masses served as analytical barcodes (see the Supporting Information). In this way we could avoid downstream deconvolution, while maximizing the compression of our high throughput generated library. The 4435 successfully formed products were consolidated into ∼70 pools of ∼60 compounds (Figure 3).

As a target for screening our library of molecular glues we selected LCK because of its validated dependency in Acute Lymphoblastic Leukemia (ALL) and established degradability by PROTACs.^36,37^ The latter indicated that proximity induction of LCK to CRBN should yield ubiquitination and ultimately degradation, thereby providing a route to validate any discovered molecular glues.

A two-phase strategy was followed to identify molecular glues for the CRBN–LCK protein pair by ASMS. First we subjected our entire library to ASMS against 5 μM CRBN–LCK which provided ∼500 candidate molecules agnostic of preferences for either protein partner or ternary complex. Next, candidate molecules were repooled and screened against 5 μM CRBN–LCK, 5 μM CRBN, and 5 μM LCK individually. From here an enrichment of >1.5-fold signified compounds for follow-up, which yielded 5 candidate hits (**17**–**21**) all featuring indoles (Figure 4a,b). Resynthesis and validation of these 5 hits provided essentially the same data (Figure 4c, Supplementary Figure 6). Moreover, hit calling was found to be largely independent of the pool identity when changing the location of hits **17**–**19** (Figure 4d, Supplementary Figure 7).

**Figure 4:**
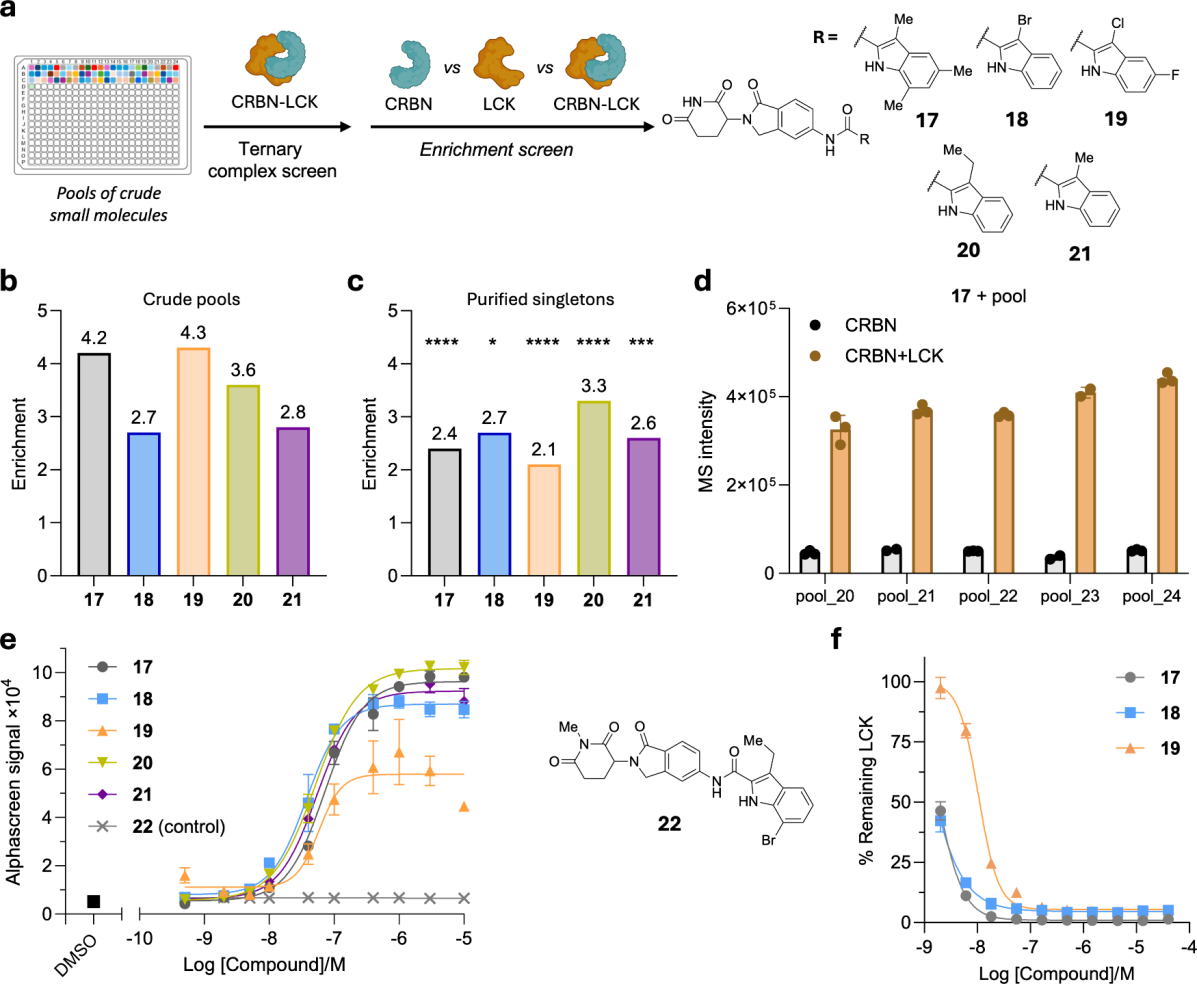
Iterative affinity selection mass spectrometry yields a glue for CRBN–LCK. (a) Sequential ASMS categorization of small molecules yielded five candidate molecular glues, which demonstrated affinity enrichment. (b) Enrichment values for selected molecules **17**–**21** from the initial screen. (c) Resynthesized pure **17**–**21** were analyzed as singletons by ASMS against CRBN–LCK (*n* = 3) and compared for their enrichment relative to CRBN-only controls. Data are presented as the mean enrichment. One-way ANOVA was performed to analyze the differences between protein–ligand complexes which confirmed consistent ternary complex mediated affinity enrichment. (d) Randomizing pooling confirmed that hit identification was independent of pool identity. (e) Alphascreen proximity induction data indicated that **17**–**21** induced proximity *in vitro*. Non-CRBN binding negative control **22** confirmed **17**–**21** as orthosteric molecular glues for CRBN. (f) Degradation of LCK was validated using a HiBit assay. See the Supporting Information for experimental details.

Molecules selected by our affinity-selection workflow behaved as molecular glues in orthogonal assays, which confirmed they could affect proximity induction within recombinant systems and whole cells (Figure 4e,f). Alphascreen assays showed that compounds **17**–**21** initiated highly effective proximity indication with EC_50_ values in the double-digit nM range (Figure 4e). Supportive HiBit experiments involving whole cells similarly validated that **17**–**19** were active LCK degraders (Figure 4f). Further affirmation that these effects are driven by ternary complex formation was found in methylated analog **22** which exhibited no proximity induction *in vitro* and was consistent with methylation-mediated abolishment of CRBN engagement (Figure 4e). Immunoblot data promisingly support the idea that indole scaffolds can be the basis for the design of CRBN-mediated degraders of native LCK (Supplementary Figure 8). These data demonstrated that ASMS could identify molecular glues for protein targets by using crude mixtures of small molecules. Moreover, medicinal chemistry optimization of these indole-appended CRBN binders was found to be effective at degrading LCK toward addressing resistance to dasatinib-based PROTACs in T-cell acute lymphoblastic leukemia.^38^

Having utilized ASMS to identify small molecules based on their capacity to act as molecular glues, we sought to further validate the potential of our method for lead discovery by establishing the operational characteristics underlying the *in vitro* identification of molecular glues by affinity enrichment. To achieve this, we selected a series of 21 additional analogs to better scrutinize the dynamic range of features underlying the effective molecular glues for CRBN–LCK (**23**–**42**, Supplementary Figure 9a). Individual affinity enrichment experiments were performed by ASMS for these CRBN–LCK glues. Head-to-head comparisons against alphascreen proximity induction data showed that enrichment directly characterizes ternary complex stability (Figure 5a,b). Indeed, by using a threshold of 1.5-fold enrichment we could categorize most molecular glues with EC50s of <200 nM (Figure 5a, Supplementary Figure 9b). We found that these trends similarly tracked with AUC measurements from alphascreen assays (Figure 5b, Supplementary Figure 9c).

**Figure 5:**
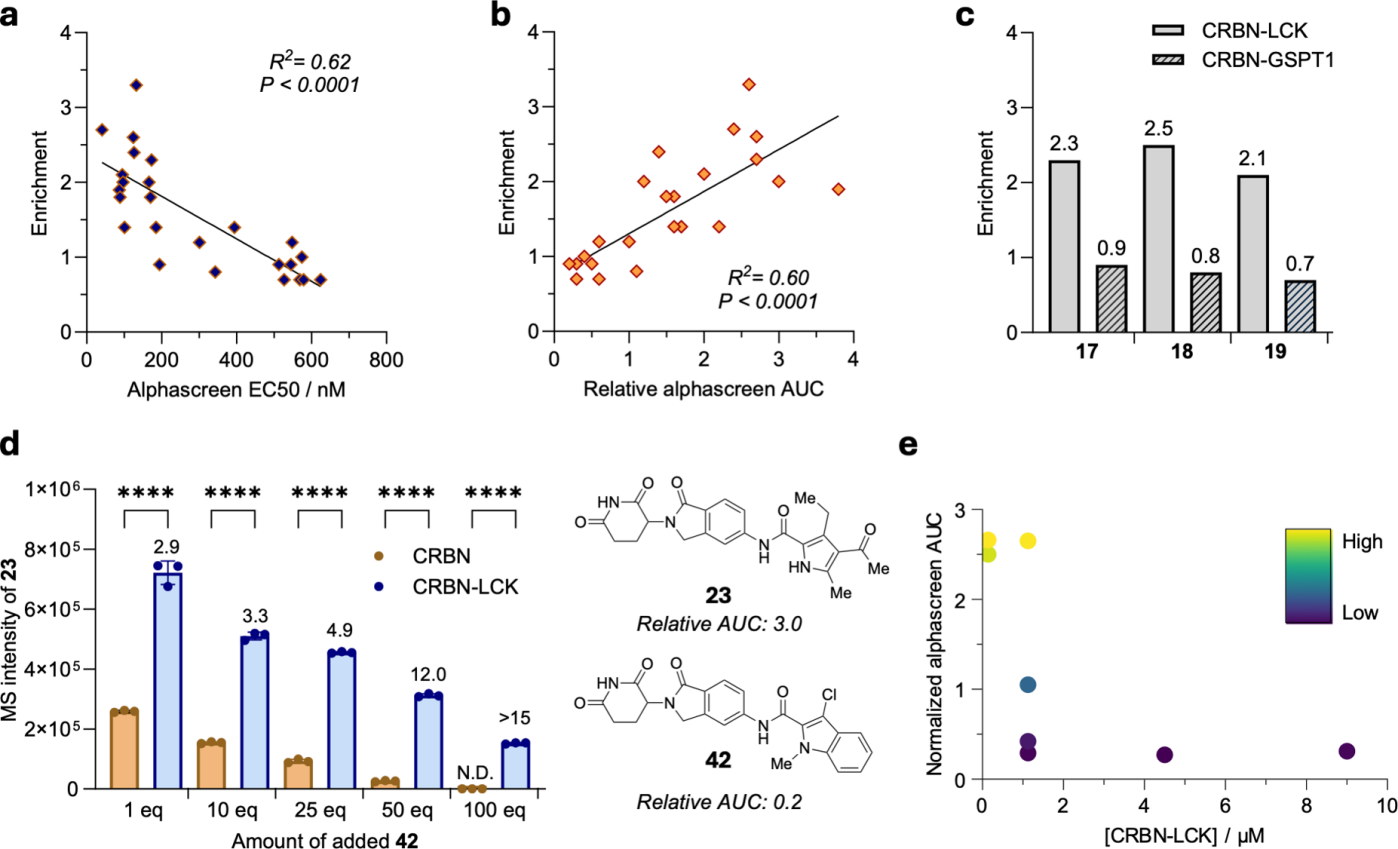
Ternary complex identity and stability drive ligand enrichment by ASMS. (a) Linear regression analysis of a suite of small molecule glues for CRBN–LCK comparing EC50 values determined by alphascreen and ASMS enrichment. The molecules with highest activity (EC50 < 200 nM) showed >1.5-fold enrichment by affinity selection. (b) AUC data from these alphascreen experiments were similarly captured by ASMS at a threshold of 1.5-fold enrichment. (c) Consistent with ternary complex mediated enrichment, neosubstrate identity determines affinity enrichment shown here for three LCK glues which are exclusively enriched through addition of LCK and not by GSPT1. (d) Increased competition of nonglue **42** against LCK glue **23** enhances affinity enrichment at the expense of signal intensity, further increasing the likelihood of discovering molecular glues by ASMS under competitive conditions. Data are presented as the mean ± SD (*n* = 3). One-way ANOVA was performed to analyze the differences between ligand complexes. (e) Decreasing concentrations of CRBN–LCK ternary complex drive ligand competition allowing rank ordering of analogs by ASMS. AUC = Area under the curve. See the Supporting Information for the experimental details.

A critical determinant of an effective molecular glue is its capacity to stabilize specific substrate–neosubstrate interactions. Taking newly discovered CRBN–LCK molecular glues **17**, **18**, and **19** we found that the addition of a GSPT1 in place of LCK led to no signal enrichment by ASMS despite being a CRBN neosubstrate (Figure 5c). These data again support ternary complex formation as the driver of increased ligand signal and affirm neosubstrate identity as a selectivity determining element for discovering molecular glues by ASMS.

To better understand the behavior of potential lead compounds within a screening scenario, we considered that good performers would compete with other ligands for CRBN–LCK. In this scenario the exchange processes between the different ligand bound states should be such that the strongest ternary complex serves as a thermodynamic sink, thereby leading to enrichment relative to CBRN. To demonstrate the tolerance of this thermodynamic sink to competitive binding, we employed a CRBN binder (**42**) with limited effectivity as a molecular glue for CRBN–LCK (relative alphascreen AUC = 0.2) and a highly effective molecular glue (**23**, relative alphascreen AUC = 3.0). We found that enrichment of **23** persisted even in the presence of up to 100 equiv of **42** (Figure 5d). Thus, the thermodynamic reorganization of protein bound ligands upon ternary complex formation leads to elevated levels of enrichment as competition increases due to the depletion of the CRBN bound ligand reference that determines enrichment.

As a final test of the resolution of ASMS for fine-tuned *in vitro* discovery of molecular glues, we investigated the competition of a suite of advanced derivatives for the CRBN–LCK ternary complex. Thus, the 8 molecular glues were pooled and competed against CRBN–LCK across a range of 5 to 0.165 μM (Figure 5e). Rapid deprioritization of inactive, weak, and moderate molecular glues was consistent with our alphascreen data. Indeed, the best performing molecule within the alphascreen assays (**20**) was the final remaining molecule within this ternary complex competition experiment.

In conclusion, a major bottleneck in leveraging the functional potential of small molecules remains the rapid selection of hits—direct-to-biology methods are increasingly providing a fast-track to on-target ligand prioritization. For direct-to-biology approaches, the utilization of crude unpurified small molecules is a central acceleratory advantage that avoids laborious purifications that would otherwise limit both the volume and diversity of analogs. Here we show how this strategy can be applied to the profiling of crude small molecules against protein pairs for the gluing behavior. In this manifold, our function-first strategy stands to facilitate a more detailed understanding of molecular and biomolecular requirements for the design of effective molecular glues, spurring a pivotal shift from serendipity toward rational design. More broadly, this represents another important step toward increasing the adoption of direct-to-biology approaches.

## Supplementary Material



## Notes

All supplementary figures, experimental protocols, and NMR data (PDF)

## References

[1] Schreiber S. L. (2024). Molecular
glues and bifunctional compounds: Therapeutic modalities based on
induced proximity. Cell Chem. Biol..

[2] Schreiber S. L. (2021). The rise
of molecular glues. Cell.

[3] Konstantinidou M., Arkin M. R. (2024). Molecular glues
for protein-protein interactions: Progressing
toward a new dream. Cell Chem. Biol..

[4] Robinson S. A., Co J. A., Banik S. M. (2024). Molecular
glues and induced proximity:
An evolution of tools and discovery. Cell Chem.
Biol..

[5] Fegan A., White B., Carlson J. C., Wagner C. R. (2010). Chemically controlled
protein assembly: techniques and applications. Chem. Rev..

[6] Sakamoto K.
M., Kim K. B., Kumagai A., Mercurio F., Crews C. M., Deshaies R. J. (2001). Protacs:
chimeric molecules that target proteins to
the skp1-cullin-F box complex for ubiquitination and degradation. Proc. Natl. Acad. Sci. U.S.A..

[7] Schapira M., Calabrese M. F., Bullock A. N., Crews C. M. (2019). Targeted protein
degradation: expanding the toolbox. Nat. Rev.
Drug Discovery.

[8] Winter G. E., Buckley D. L., Paulk J., Roberts J. M., Souza A., Dhe-Paganon S., Bradner J. E. (2015). Phthalimide conjugation
as a strategy
for in vivo target protein degradation. Science.

[9] Békés M., Langley D. R., Crews C. M. (2022). PROTAC
targeted protein degraders:
the past is prologue. Nat. Rev. Drug. Disc..

[10] Fan A. T., Gadbois G. E., Huang H.-T., Chaudhry C., Jiang J., Sigua L. H., Smith E. R., Wu S., Poirier G. J., Dunne-Dombrink K., Goyal P., Tao A. J., Sellers W. R., Fischer E. S., Donovan K. A., Ferguson F. M. (2025). A Kinetic
Scout
Approach Accelerates Targeted Protein Degrader Development. Angew. Chem., Int. Ed..

[11] Hinterndorfer M., Spiteri V. A., Ciulli A., Winter G. E. (2025). Targeted protein
degradation for cancer therapy. Nature Reviews
Cancer.

[12] Baek K., Metivier R. J., Roy Burman S. S., Bushman J. W., Yoon H., Lumpkin R. J., Ryan J. K., Abeja D. M., Lakshminarayan M., Yue H., Ojeda S., Xiong Y., Che J., Verano A. L., Schmoker A. M., Gray N. S., Donovan K. A., Fischer E. S. (2025). Unveiling
the hidden interactome of CRBN molecular glues. Nat. Commun..

[13] Forrest I., Conway L. P., Gathmann C., Jadhav A. M., Chiu T.-Y., Chaheine C. M., Estrada M., Shrestha A., Sarris K., Reitsma J. M., Warder S. E., Vasudevan A., McLoughlin S. M., Parker C. G. (2025). Proteome-wide discovery
of degradable
proteins using bifunctional molecules. ACS Cent.
Sci..

[14] Petrilli W. L., Adam G. C., Erdmann R. S., Abeywickrema P., Agnani V., Ai X., Baysarowich J., Byrne N., Caldwell J. P., Chang W., DiNunzio E., Feng Z., Ford R., Ha S., Huang Y., Hubbard B., Johnston J. M., Kavana M., Lisnock J.-M., Liang R., Lu J., Lu Z., Meng J., Orth P., Palyha O., Parthasarathy G., Salowe S. P., Sharma S., Shipman J., Soisson S. M., Strack A. M., Youm H., Zhao K., Zkin D. L., Zokian H., Addona G. H., Akinsanya K., Tata J. R., Xiong Y., Imbriglio J. E. (2020). From screening
to targeted degradation: strategies for the discovery and optimization
of small molecules ligands for PCSK9. Cell Chem.
Biol..

[15] Baek K., Schulman B. A. (2020). Molecular glue concept solidifies. Nat. Chem. Biol..

[16] Jiang W., Jiang Y., Luo Y., Qiao W., Yang T. (2024). Facilitating
the development of molecular glues: Opportunities from serendipity
and rational design. Eur. J. Med. Chem..

[17] Wang Z., Shaabani S., Gao X., Ng Y. L. D., Sapozhnikova V., Mertins P., Krönke J., Dömling A. (2023). Direct-to-biology,
automated, nano-scale synthesis, and phenotypic screening-enabled
E3 ligase modulator discovery. Nat. Commun..

[18] Mason J. W., Chow Y. T., Hudson L., Tutter A., Michaud G., Westphal M. V., Shu W., Ma X., Tan Z. Y., Coley C. W., Clemons P. A., Bonazzi S., Berst F., Briner K., Liu S., Zécri F., Schreiber S. L. (2024). DNA-encoded library-enabled discovery of proximity-inducing
small molecules. Nat. Chem. Biol..

[19] Dong G., Ding Y., He S., Shang C. (2021). Molecular glues for
targeted protein degradation: from serendipity to rational discovery. J. Med. Chem..

[20] Mennen S. M., Alhambra C., Allen C. L., Barberis M., Berritt S., Brandt T. A., Campbell A. D., Castañón J., Cherney A. H., Christensen M., Damon D. B., de Diego J. E., García-Cerrada S., García-Losada P., Haro R., Janey J., Leitch D. C., Li L., Liu F., Lobben P. C., MacMillan D. W. C., Magano J., McInturff E., Monfette S., Post R. J., Schultz D., Sitter B. J., Stevens J. M., Strambeanu I. I., Twilton J., Wang K., Zajac M. A. (2019). The evolution of high-throughput experimentation in
pharmaceutical development and perspectives on the future. Org. Process Res. Dev..

[21] Stevens R., Bendito-Moll E., Battersby D. J., Miah A. H., Wellaway N., Law R. P., Stacey P., Klimaszewska D., Macina J. M., Burley G. A., Harling J. D. (2023). Integrated
direct-to-biology
platform for the nanoscale synthesis and biological evaluation of
PROTACs. J. Med. Chem..

[22] Stevens R., Shrives H. J., Cryan J., Klimaszewska D., Stacey P., Burley G. A., Harling J. D., Battersby D. J., Miah A. H. (2025). Expanding the reaction toolbox for
nanoscale direct-to-biology
PROTAC synthesis and biological evaluation. RSC Med. Chem..

[23] Rowe S. M., Price A., Murphy D. J., Lin J., Nartey E. N., Chaikaud A., Wong K., Cottom J. E., Concha N. O., Reid R. A., Dickinson E. R., Jundt M., Kammerer K., Steidel M., Mathieson T., Werner T., Grant E. K., Stanborough C. K., Rouah M., Wojno-Picon J., Pogány P., Pettinger J., Norman D. J., Wilders H., Rainjongdee F., Valdes-Garcia G., Nevins N., Shenje R., Thalji R. K., Chung C.-W., Eberl H. C., Neubauer G., House D., Rao Y., Martino M. P., Bush J. T. (2025). Direct-to-biology
drives optimization of a cell-active covalent inhibitor of WRN helicase. ChemRxiv.

[24] Gesmundo N. J., Sauvagnat B., Curran P. J., Richards M. P., Andrews C. L., Dandliker P. J., Cernak T. (2018). Nanoscale synthesis and affinity
ranking. Nature.

[25] Douthwaite J. L., Houde D. J., Pardo E. H., Moran M. S., Baird J., Yeyer S. R., Bahjour B., Zhao Q., Larrow J. F., Juang Y. P., Han C. K., Kelley B., Dunstan D. R., Billings K. J., Mader M. M., Taylor A. M., Sexton J. Z., Boezio A. A., Cernak T. (2025). Nanoscale
direct-to-biology optimization
of Cdk2 inhibitors. ChemRxiv.

[26] Prudent R., Annis D. A., Dandliker P. J., Ortholand J.-Y., Roche D. (2021). Exploring new targets and chemical space with affinity selection-mass
spectrometry. Nat. Rev. Chem..

[27] Prudent R., Lemoine H., Walsh J., Roche D. (2023). Affinity selection
mass spectrometry speeding drug discovery. Drug
Discovery Today.

[28] Muckenschnabel I., Flachetto R., Mayr L. M., Filipuzzi I. (2004). SpeedScreen:
label-free liquid chromatography–mass spectrometry-based high-throughput
screening for the discovery of orphan protein ligands. Anal. Biochem..

[29] Nishiguchi G., Keramatnia F., Min J., Chang Y., Jonchere B., Das S., Actis M., Price J., Chepyala D., Young B., McGowan K., Slavish P. J., Mayasundari A., Jarusiewicz J. A., Yang L., Li Y., Fu X., Garrett S. H., Papizan J. B., Kodali K., Peng J., Pruett-Miller S. M., Roussel M. F., Mullighan C., Fischer M., Rankovic Z. (2021). Identification of potent, selective,
and orally bioavailable small-molecule GSPT1/2 degraders from a focused
library of cereblon modulators. J. Med. Chem..

[30] Matyskiela M. E., Zhang W., Man H.-W., Muller G., Khambatta G., Baculi F., Hickman M., LeBrun L., Pagarigan B., Carmel G., Lu C.-C., Lu G., Satoh Y., Schafer S., Daniel T. O., Carmichael J., Cathers D. E., Chamberlain P. P. (2018). A cereblon modulator (CC-220) with
improved degradation of Ikaros and Aiolos. J.
Med. Chem..

[31] Holderfield M., Lee B. J., Jiang J., Tomlinson A., Seamon K. J., Mira A., Patrucco E., Goodhart G., Dilly J., Gindin Y., Dinglasan N., Wang Y., Lai L. P., Cai S., Jiang L., Nasholm N., Shifrin N., Blaj C., Shah H., Evans J. W., Montazer N., Lai O., Shi J., Ahler E., Quintana E., Chang S., Salvador A., Marquez A., Cregg J., Liu Y., Milin A., Chen A., Ziv T. B., Parsons D., Knox J. E., Klomp J. E., Roth J., Rees M., Ronan M., Cuevas-Navarro A., Hu F., Lito P., Santamaria D., Aguirre A. J., Waters A. M., Der C. J., Ambrogio C., Wang Z., Gill A. K., Koltun E. S., Smith J. A. M., Wildes D., Singh M. (2024). Concurrent inhibition of oncogenic
and wild-type RAS-GTP for cancer therapy. Nature.

[32] Kim D., Herdeis L., Rudolph D., Zhao Y., Böttcher J., Vies A., Ayala-Santos C. I., Pourfarjam Y., Cuevas-Navarro A., Xue J. Y., Mantoulidis A., Bröker J., Wunberg T., Schaaf O., Popow J., Wolkerstorfer B., Kropatsch K. G., Qu R., de Stanchina E., Sang B., Li C., McConnell D. B., Kraut N., Lito P. (2023). Pan-KRAS inhibitor disables oncogenic
signaling and tumour growth. Nature.

[33] Santanilla A. B., Regalado E. L., Pereira T., Shevlin M., Bateman K., Campeau L.-C., Schneeweis J., Berritt S., Shi Z.-C., Nantermet P., Lui Y., Helmy R., Welch C. J., Vachal P., Davies I. W., Cernak T., Dreher S. D. (2014). Nanomole-scale
high-throughput chemistry for the synthesis of complex molecules. Science.

[34] Hu M., Yang L., Twarog N., Ochoada J., Li Y., Vrettos E. I., Torres-Hernandez A. X., Martinez J. B., Bhatia J., Young B. M., Price J., McGowan K., Nguyen T. H., Shi Z., Anyanwu M., Rimmer M. A., Mercer S., Rankovic Z., Shelat A. A., Blair D. J. (2024). Continuous collective analysis of
chemical reactions. Nature.

[35] Fischer E. S., Böhm K., Lydeard J. R., Yang H., Stadler M. B., Cavadini S., Nagel J., Serluca F., Acker V., Lingaraju G. M., Tichkule R. B., Schebesta M., Forrester W. C., Schirle M., Hassiepen U., Ottl J., Hild M., Beckwith R. E. J., Harper J. W., Jenkins J. L., Thomä N. H. (2014). Structure
of the DDB1-CRBN E3 ubiquitin
ligase in complex with thalidomide. Nature.

[36] Hu J., Jarusiewicz J., Du G., Nishiguchi G., Yoshimura S., Panetta J. C., Li J., Min J., Yang L., Chepyala D., Actis M., Reyes N., Smart B., Pui C.-H., Teachey D. T., Rankovic Z., Yang J. J. (2022). Preclinical evaluation of proteolytic targeting of
LCK as a therapeutic approach in T cell acute lyphoblastic leukemia. Sci. Trans. Med..

[37] Jarusiewicz J. A., Yoshimura S., Actis M., Li Y., Fu X., Yang L., Narina S., Pruett-Miller S. M., Zhou S., Wang X., High A. A., Nishiguchi G., Yang J. J. (2024). Development of an orally bioavailable LCK PROTAC degrader
as a potential therapeutic approach to T-cell acute lymphoblastic
leukemia. J. Med. Chem..

[38] Yoshimura S., Actis M., Seffernick J. T., Jarusiewicz J. A., Aggarwal A., Li A., Li Y., Lee D., Yang L., Maysundari A., Rankovic Z., Fischer M., Nishiguchi G., Yang J. J. (2025). LCK-targeting molecular glues overcome
resistance to inhibitor-based therapy in T-cell acute lymphoblastic
leukemia. BioRxiv.

