# Supplementary material for: Direct-to-Biology Enabled Molecular Glue Discovery
Source: J Am Chem Soc. 2025 Dec 24;148(1):20–7. doi: 10.1021/jacs.5c13496 (PMC12814336; doi:10.1021/jacs.5c13496)
Supplement: Supplementary file 1 [file ja5c13496_si_001.pdf]

Supplementary Materials for

Direct-to-Biology Enabled Molecular Glue Discovery

Maowei Hu [a], Jason Ochoada [b], Marisa Actis [c], Kevin McGowan [d], Jamie A. Jarusiewicz [c], Satoshi Yoshimura [e], Logan McGrath [e], Uma Neelakantan [a,f], Anup Aggarwal [c], Anand Mayasundari [c], Sarah M. Young [a], Meng Zhang [f], Lei Yang [g], Yong Li [g], Shea Mercer [h], M. Madan Babu [f], Marcus Fischer [a], Brandon M. Young [d], Jun J. Yang [e], Gisele Nishiguchi [c], Anang A. Shelat [b], Daniel J. Blair [a*]

[a] Department of Chemical Biology and Therapeutics, St Jude Children's Research Hospital, Memphis, TN 38105, USA.

[b] Lead Discovery Informatics Center, Department of Chemical Biology and Therapeutics, St Jude Children's Research Hospital, Memphis, TN 38105, USA.

[c] Targeted Protein Degradation Center, Department of Chemical Biology and Therapeutics, St Jude Children's Research Hospital, Memphis, TN 38015, USA.

[d] Medicinal Chemistry Center, Department of Chemical Biology and Therapeutics, St Jude Children's Research Hospital, Memphis, TN 38105, USA.

[e] Department of Pharmacy and Pharmaceutical Sciences, St Jude Children's Research Hospital, Memphis TN 38105, USA.

[f] Center of Excellence for Data Driven Discovery, Department of Structural Biology, St Jude Children's Research Hospital, Memphis, TN 38105, USA.

[g] Analytical Technologies Center, Department of Chemical Biology and Therapeutics, St Jude Children's Research Hospital, Memphis, TN 38105, USA.

[h] Program Management, Department of Chemical Biology and Therapeutics, St Jude Children's Research Hospital, Memphis, TN 38015, USA.

* Email: daniel.blair@stjude.org

Contents

Supplementary Figures

Supplementary Figure 1. Additional validation of ASMS methodology for identifying molecular glues.......4

Supplementary Figure 2. Candidate cereblon binders for library generation. 5

Supplementary Figure 3. Amide bond formation reaction outcomes for amine cores....................................6

Supplementary Figure 4. Amide bond formation reaction outcomes for acid cores.......................................7

Supplementary Figure 5. Buchwald-Hartwig reactions for halogenated cores...8

Supplementary Figure 6. Affinity-selection mass spectrometry for CRBN-LCK glues. 9

Supplementary Figure 7. Changing pool identity does not alter hit-calling. 10

Supplementary Figure 8. Degradation of native LCK. 11

Supplementary Figure 9. Molecular glue SAR interrogation by AS-MS. 12

1. Materials and General Methods..13

1.1 Reagents and Solvents 13

1.2 Neutral Loss Method Development 13

1.3 Neutral Loss-Acoustic Droplet Ejection-Mass Spectrometry (NL-ADE-MS) 13

Table S1 Selected Neutral Loss Data from Product Ion Scanning MS/MS for Building Blocks 1-16.........14

1.4 Affinity Selection Mass Spectrometry (ASMS) System..16

Table S2 SEC Column Gradient Timetable..16

Table S3 RPC Column Gradient Timetable 17

1.5 High Throughput Reaction Coupling Partner Libraries...17

1.6 ASMS Pooling 17

1.7 Protein Purification 18

1.8 ASMS Assays...19

1.8.1 Enrichment Experiment using Neosubstrate GSPT1 (Figure 2a, b, c)...19

1.8.2 Titration Experiment using Neosubstrate GSPT1 (Supplementary Figure 1a)......................................19

1.8.3 Drug Like Compound Decoys vs CRBN-DDB1-GSPT1 Ternary Complex (Supplementary Figure 1b, c, d, e)..20

1.8.4 Enrichment Experiment using CypA and KRAS (Figure 2d, e) 20

1.8.5 Crude Reactions Screening against CRBN-DDB1-LCK-GST (Figure 4a, b, d and Supplementary Figure 7) 20

1.8.6 Purified compounds enrichment experiment using neosubstrate LCK-GST (Figure 4c and Figure 5c) 20

1.8.7 Competition Experiment using Neosubstrate LCK-GST (Figure 5d)...21

Table S4 Volume of Ligands 21

1.8.8 Ranking Experiment of Neosubstrate LCK-GST (Figure 5e)..21

Table S5 Ranking Experiment...21

1.9 Alphascreen Assay (Figure 4e, Figure 5a, b, e) 22
1.10 HiBiT Assay (Figure 4f) 22
1.11 Immunoblotting Experiment (Supplementary Figure 8) 23
1.12 ASMS Hit Identification 23
Table S6 Hits Identification Parameters 23
2. Synthesis 24
2.1 Synthesis of Building Blocks 24
2.2 High Throughput Miniaturized Reaction (Figure 3) 32
2.1.1 Amidation Reaction 33
Supplementary Figure 10. Amidation Reaction Source Plates Layouts 33
2.1.2 Buchwald-Hartwig C-N coupling Reaction 34
Supplementary Figure 11. Buchwald-Hartwig Reaction Source Plates Layouts. 34
2.1.3 Reaction Analysis (Supplementary Figures 3, 4, and 5) 35
2.3 Scale-up Synthesis 35
Supplementary Figure 12. Physiochemical Parameters of Carboxylic Acid Coupling Partners for Amidation Reactions. 50
Supplementary Figure 13. Physiochemical Parameters of Amine Coupling Partners for Amidation Reactions. 51
Supplementary Figure 14. Physiochemical Parameters of Amine Coupling Partners for Buchwald-Hartwig Reactions. 52
Supplementary Figure 15. Histogram Graph of ASMS Pools. 53
3. CID Spectra of Building Blocks 56
4. References 63
5. NMR Spectra 63

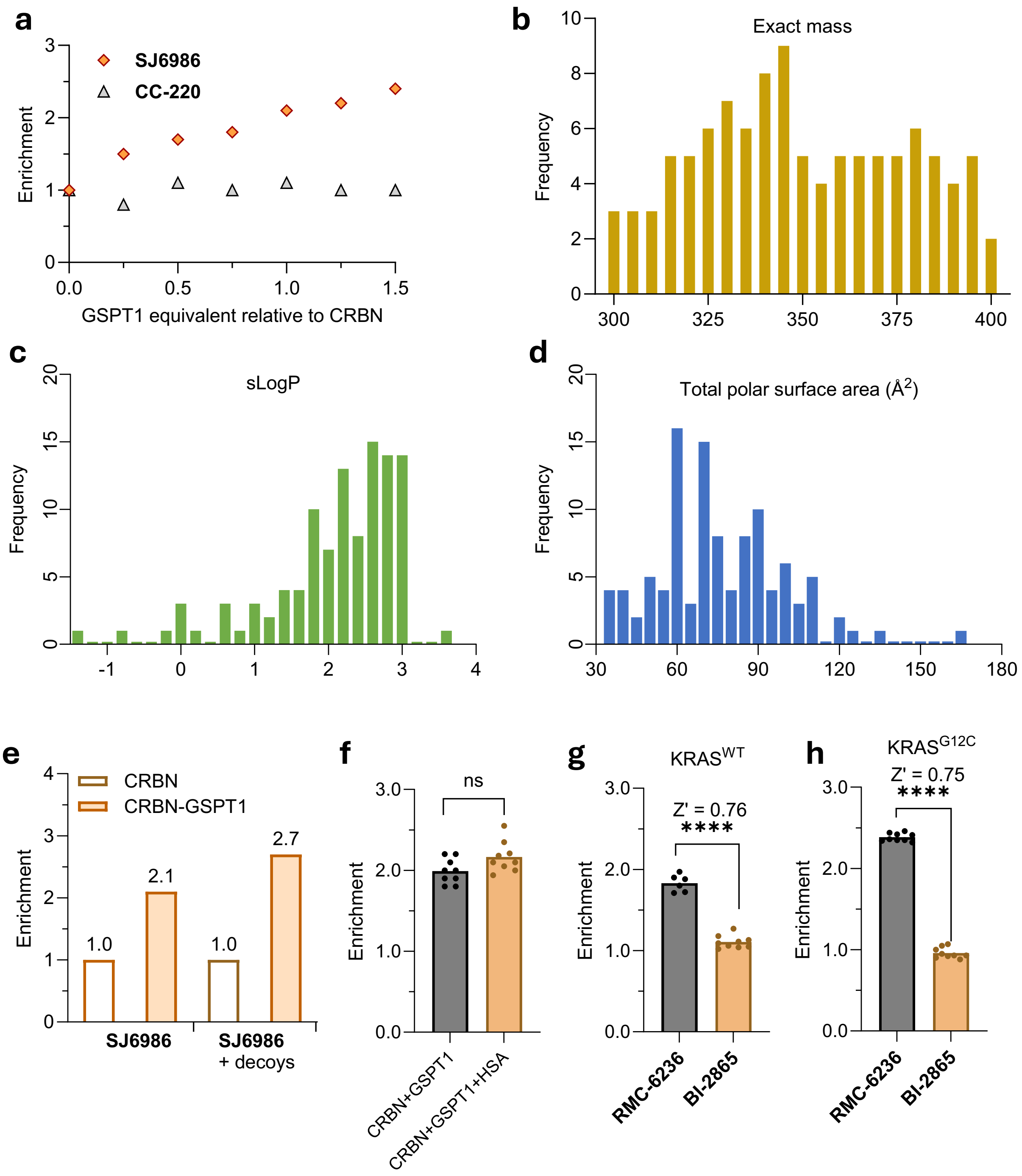

Supplementary Figure 1. Additional validation of ASMS methodology for identifying molecular glues. (a) Titration of GSPT1 to CRBN bound SJ6986 shows concentration-dependent enrichment, whereas CRBN bound CC-220 does not, validating ternary complex stabilization as a driver of enrichment. (b-d) A set of 106 decoy small molecules were selected from the St Jude compound collection spanning a range of (b) exact mass, (c) lipophilicity, and (d) polar surface area. (e) These 106 molecules were mixed with **SJ6986** and ASMS data were collected against the CRBN-GSPT1 protein pair. Affinity enrichment of **SJ6986** by ASMS was observed both with and without decoys. (f) Addition of a decoy protein to the detection of **SJ6986** did not significantly change enrichment. (g) The reproducibility of ASMS enrichment was measured for **RMC-6236** and **BI-2865** against CypA/KRAS^{WT}. (h) ASMS reproducibility were also determined for CypA/KRAS^{G12C}.

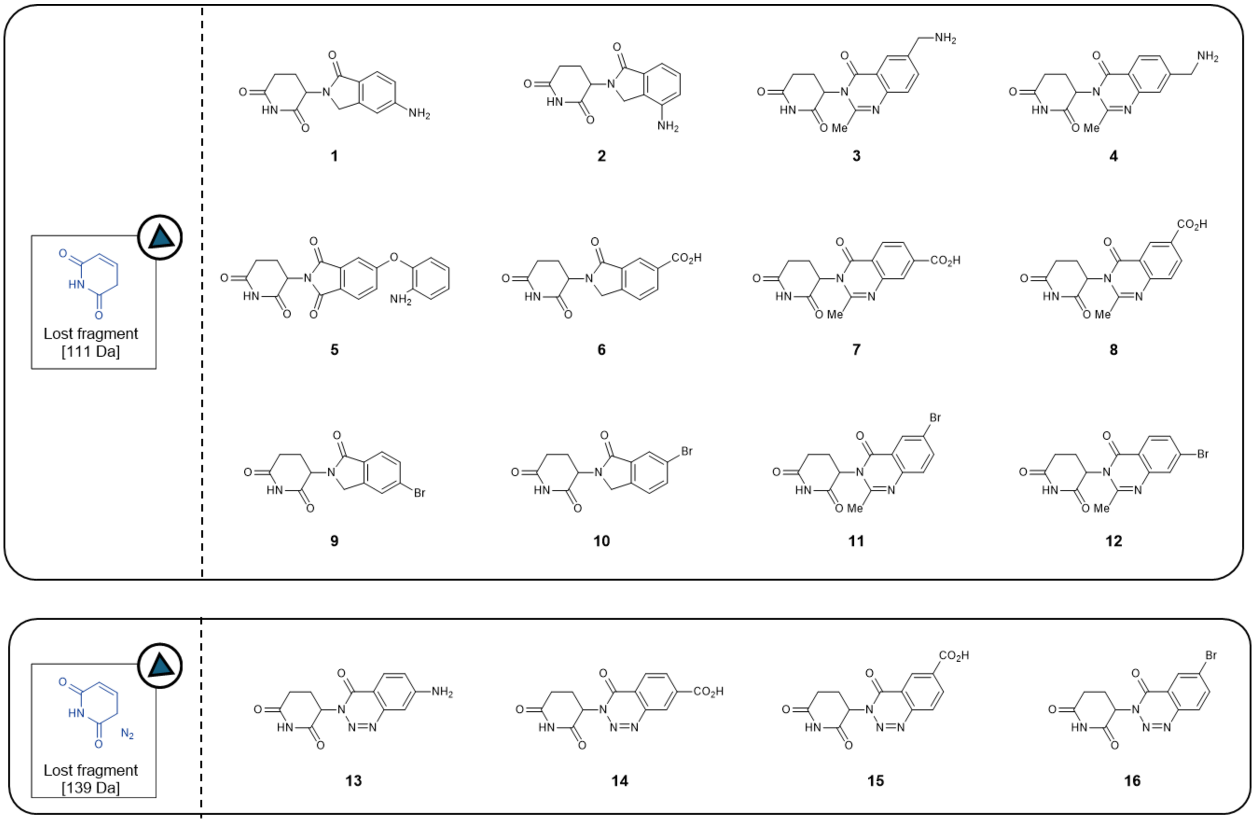

Supplementary Figure 2. Candidate cereblon binders for library generation. A series of glutarimide containing building block were assembled with each featuring common lost chemical fragments enabling of downstream product analysis by MS/MS.

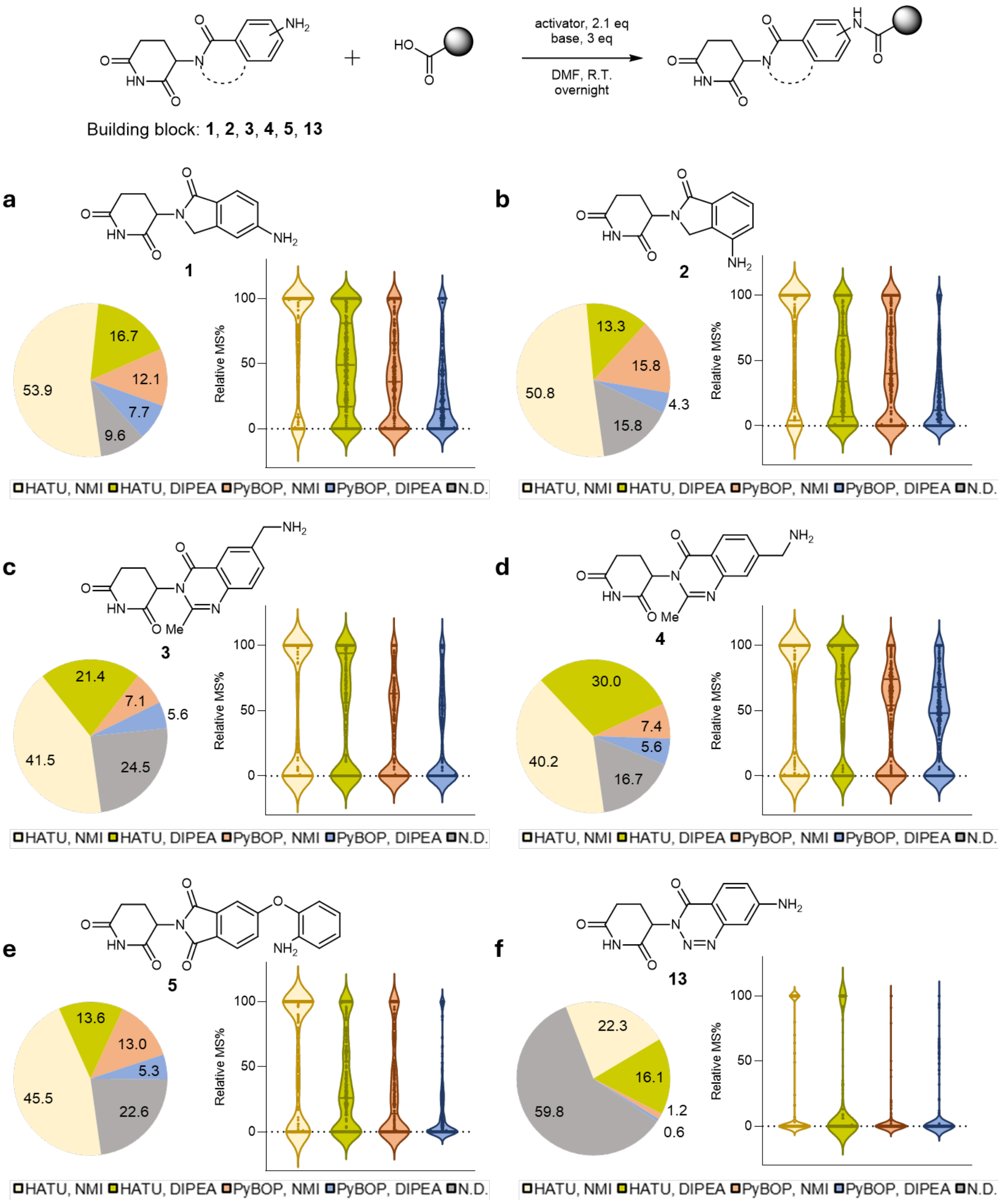

Supplementary Figure 3. Amide bond formation reaction outcomes for amine cores. High-throughput synthesis data representing the fractional selected reaction conditions as pie charts and the distribution of relative MS% as violin plots for building blocks (a) **1**, (b) **2**, (c) **3**, (d) **4**, (e) **5**, and (f) **13**. N.D. = not detected.

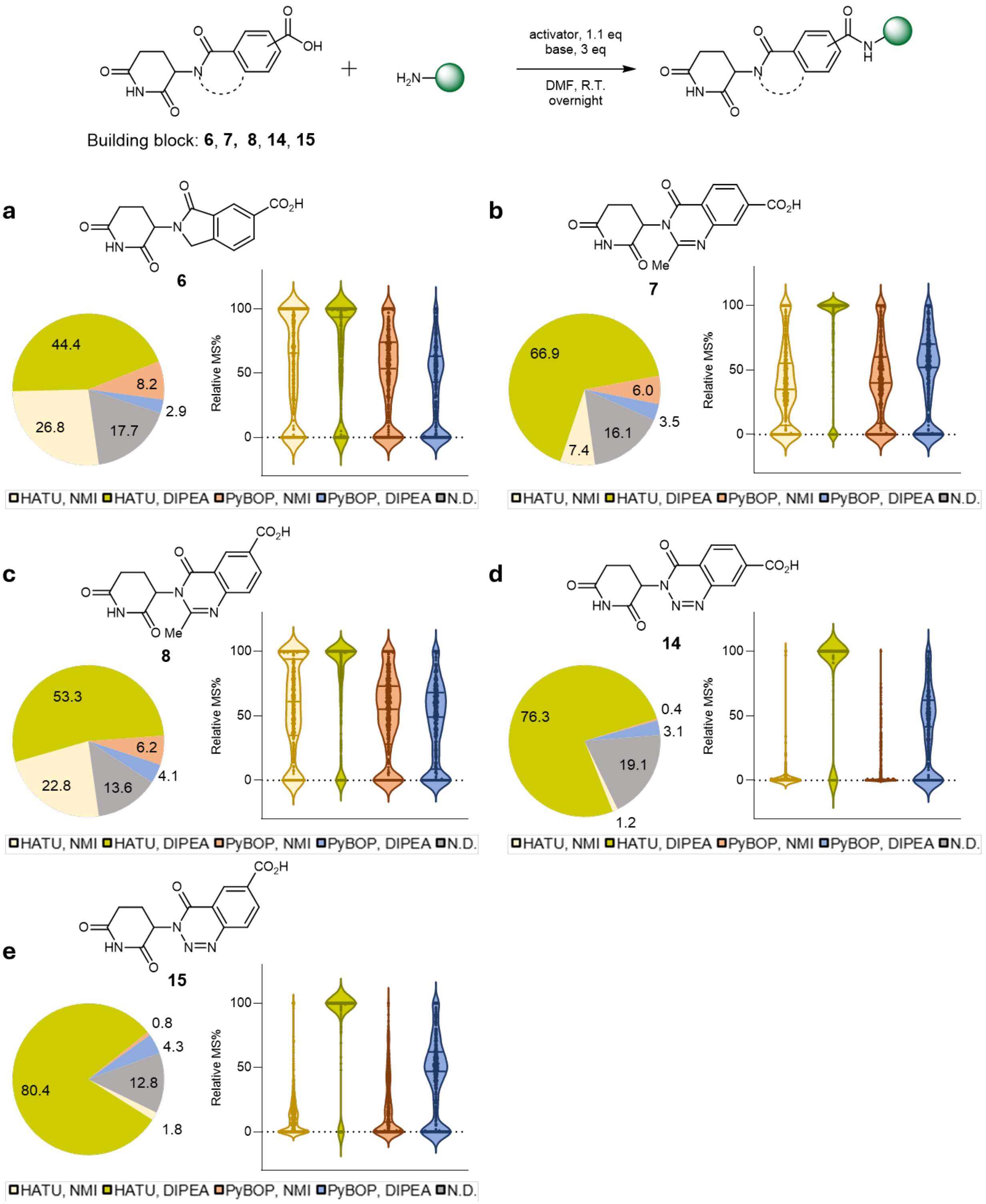

Supplementary Figure 4. Amide bond formation reaction outcomes for acid cores**.** High-throughput synthesis data representing the fractional selected reaction conditions as pie charts and the distribution of relative MS% as violin plots for building blocks (a) **6**, (b) **7**, (c) **8**, (d) **14**, and (e) **15**. N.D. = not detected.

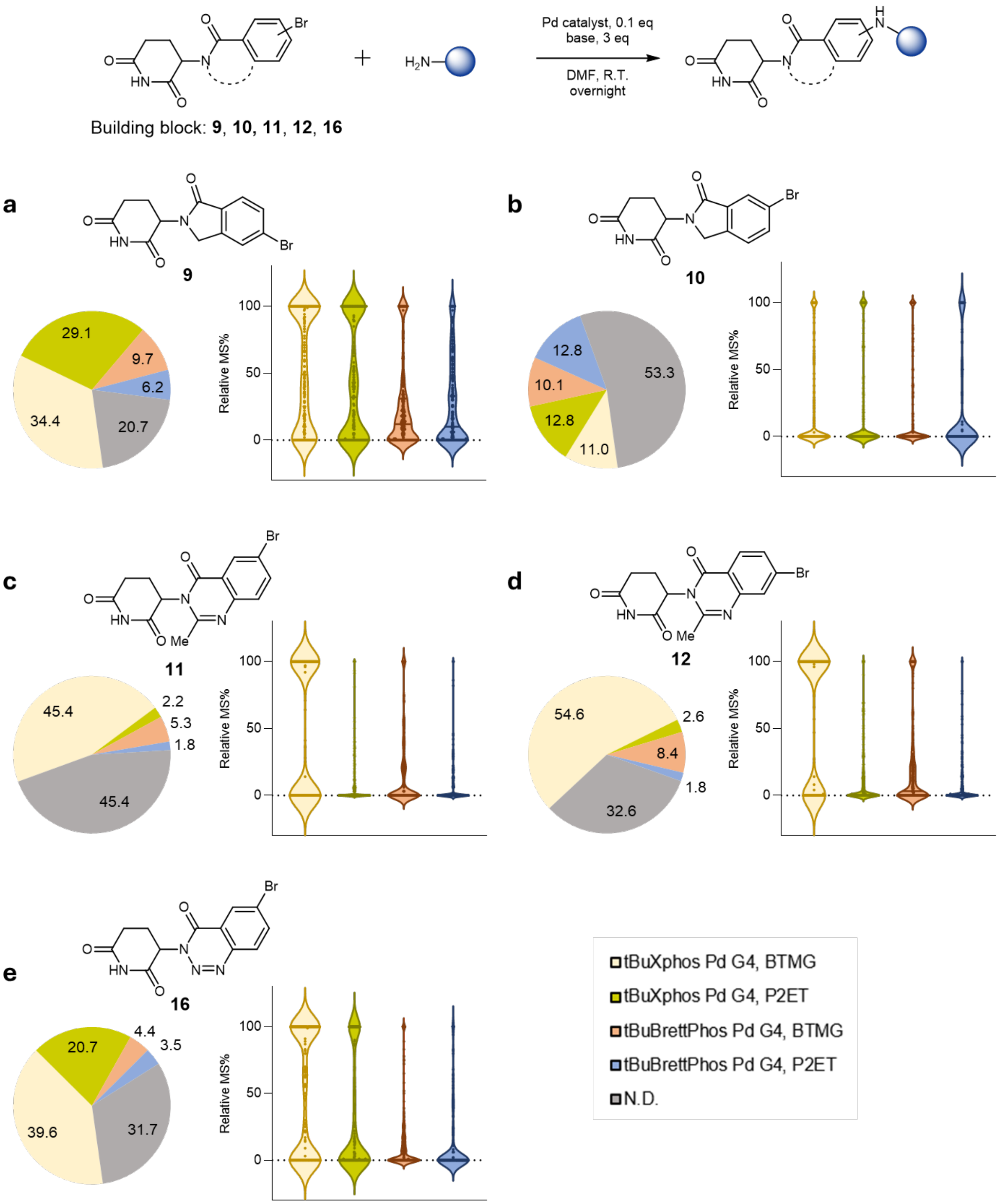

Supplementary Figure 5. Buchwald-Hartwig reactions for halogenated cores. High-throughput synthesis data representing the fractional selected reaction conditions as pie charts and the distribution of relative MS% as violin plots for building blocks (a) **9**, (b) **10**, (c) **11**, (d) **12**, and (e) **16**. N.D. = not detected. N.D. = not detected.

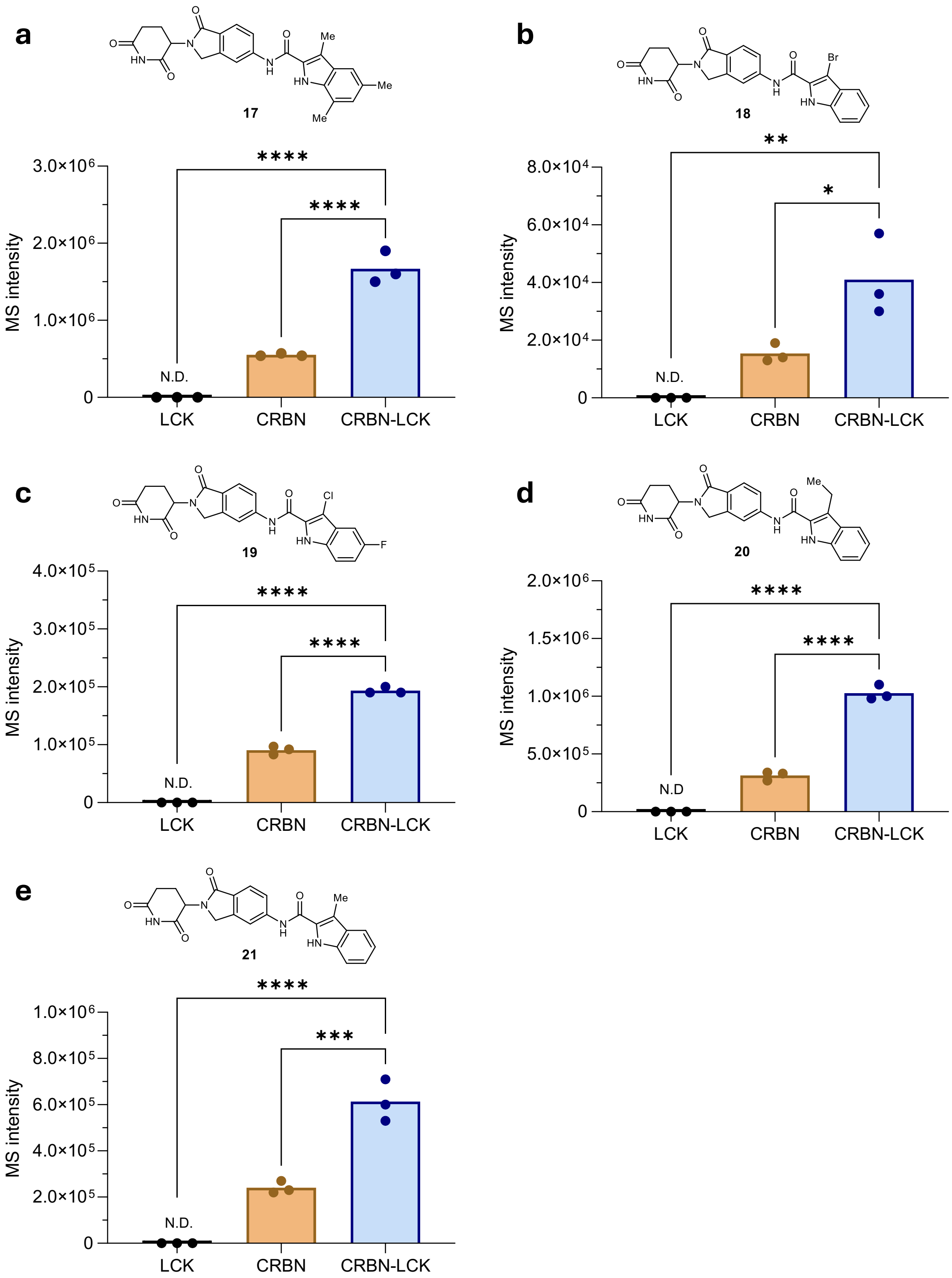

Supplementary Figure 6. Affinity-selection mass spectrometry for CRBN-LCK glues. Lead compounds **17-21** from our iterative ASMS screen were repeated in a purified format, data were consistent and of reasonable MS intensity. Figures show triplicates for (a) **17**, (b) **18**, (c) **19**, (d) **20**, and (e) **21**.

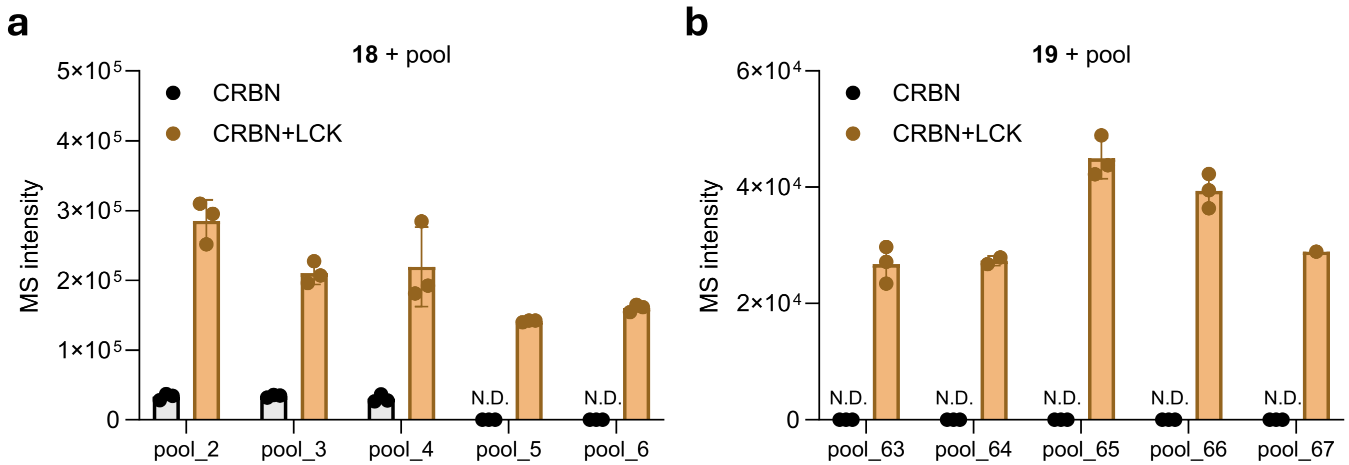

Supplementary Figure 7. Changing pool identity does not alter hit-calling. ASMS experiments using (a) **18** and (b) **19** were performed using alternative pools against CRBN-LCK. Enriched ligand signal for **18** and **19** in the presence of CRBN-LCK was minimally influenced by the identity of the pool.

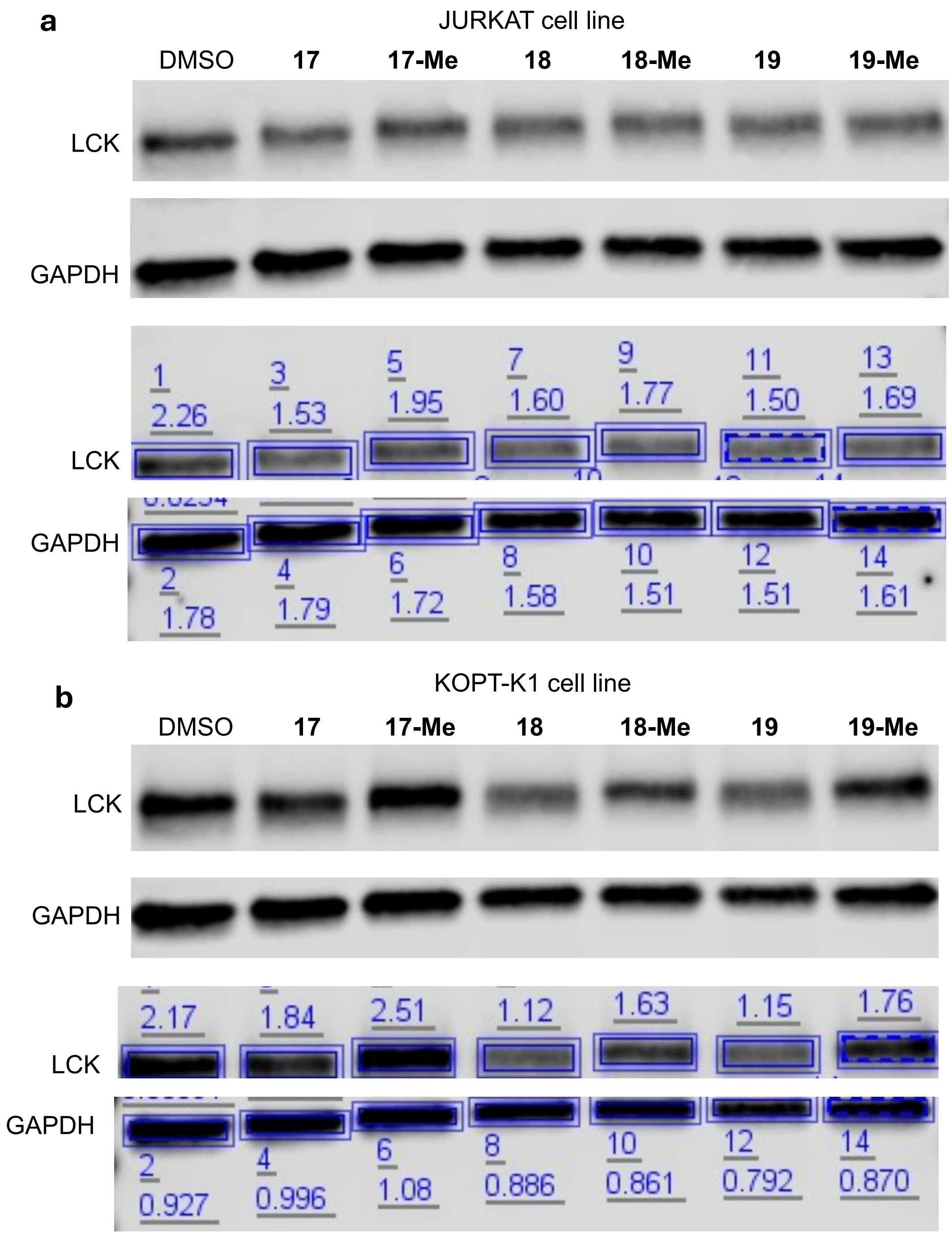

Supplementary Figure 8. Degradation of native LCK. Using compounds **17**, **18**, and **19** the degradation of native LCK was investigated by immunoblot after 24 h exposure to 1 uM ligand against (a) JURKAT and (b) KOPT-K1 cell lines. A series of *N*-methylated derivatives **17-Me**, **18-Me**, and **19-Me** were also employed to block CRBN and suppress degradation providing evidence of on-mechanism behavior for CRBN-mediated degradation.

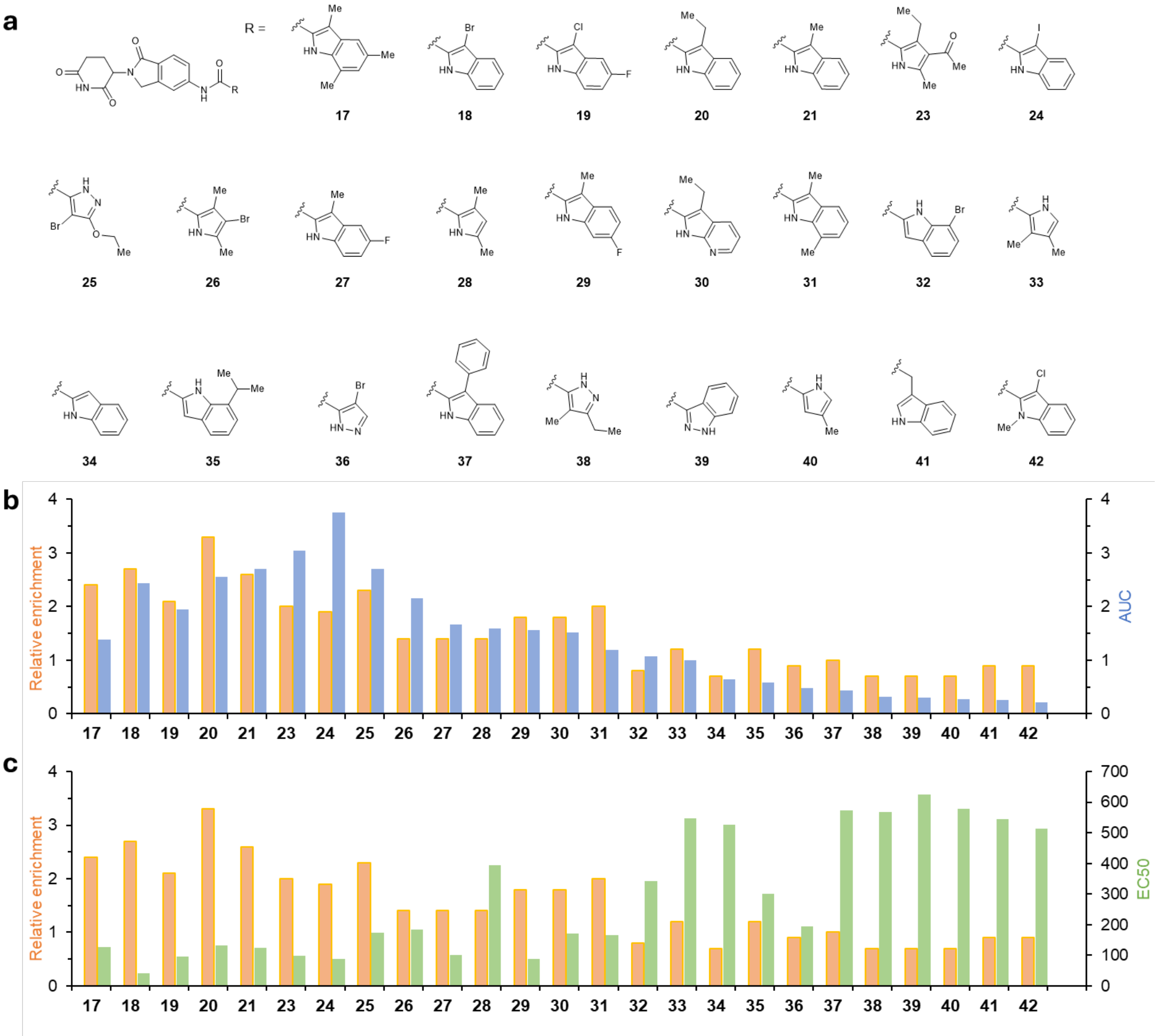

Supplementary Figure 9. Molecular glue SAR interrogation by AS-MS. (a) A range of analogs centered on structures **17-21** were tested by both ASMS and alphascreen. (b) Enrichment data against relative alphascreen AUC. (c) Enrichment data against alphascreen EC_{50} values. These data show excellent agreement for selection of the best performing molecular glues by using ternary complex mediated enrichment to identify glues.

1. Materials and General Methods

1.1 Reagents and Solvents

Air- and moisture-sensitive reactions were carried out in a nitrogen filled glovebox. Anhydrous Dimethylformamide (DMF) was purchased from Sigma Aldrich and stored in the glovebox. Dimethyl sulfoxide (DMSO) was purchased from Fisher Chemical in 4 L (ACS grade) bottles. Coupling partners were selected from St. Jude in house chemical collection and used directly as received from a variety of sources, Oakwood, Acros Organics, Alfa Aesar, Frontier Scientific, Combi-Blocks, Enamine, Matrix Scientific, and Sigma Aldrich. Ammonium acetate (ACS, Reag. Ph.Eur.) was purchased from EMSURE® (formerly GR) in 12 Kg bottle. Acetonitrile (LC-MS grade) were acquired from Fisher Scientific (Waltham, MA). Milli-Q water (Millipore, Molsheim Cedex, France) was used as produced. LC-MS grade formic acid (for mass spectrometry) was purchased from Honeywell. Building block **1** C5 Lenalidomide purchased from BroadPharm. Building block **2** Lenalidomide purchased from Combi-Blocks. Building block **6** 2-(2,6-dioxopiperidin-3-yl)-3-oxo-2,3-dihydro-1H-isoindole-5-carboxylic acid purchased from Enamine. Building block **9** 3-(5-bromo-1-oxoisoindolin-2-yl)piperidine-2,6-dione purchased from Ambeed Inc. Building block **10** 3-(6-Bromo-1-oxoisoindolin-2-yl)piperidine-2,6-dionepurchased from BroadPharm. Daraxonrasib and BI-2865 were purchased from MedChemExpress. Cyclophilin A protein (mouse) was purchased MedChemExpress. ^{1}H and ^{13}C Nuclear Magnetic Resonance (NMR) spectra were recorded in DMSO-d6 solvent on Bruker Ascend 500 with Avance III HD Console (at 500 MHz and 126 MHz). Chemical shifts (δ_H , δ_C) are calibrated to the residual protio solvent signals of DMSO-d6 (2.50 ppm, 39.5 ppm). Coupling constants are quoted in parts per million (ppm) and refer to apparent multiplicities. Data are listed as follows: chemical shift, multiplicity (s = singlet, d = doublet, t = triplet, q = quartet, quin =quintet, sext = sextet, sept = septet, m = multiplet, br = broad peak, dd = doublet of doublet, etc.), coupling constant, integration, and assignment.

1.2 Neutral Loss Method Development

The multiple reaction monitoring (MRM) transitions of each building block was monitored using a SCIEX ExionLC/Qtrap 6500 plus triple-quadrupole mass spectrometer system from SCIEX (Forster City, CA), equipped with an electrospray ion (Turbo Ionspray) interface. The SCIEX ExionLC featured two pumps, an integrated degasser, column oven, autosampler, and sample organizer. Nitrogen served as curtain, nebulizer, and collision gas. Analytes were dissolved individually in 50% acetonitrile aqueous solution and were introduced into the ion source of the mass spectrometer by an autosampler. The following solvent series of was used to introduce the sample and wash the system: The isocratic program had a run time of 2 minutes at 25% A (0.1% formic acid in Milli-Q H_2O) and 75% B (0.1% formic acid in acetonitrile). The flow rate started at 0.4 mL/min, held for 0.3 minutes, transitioned to 0.05 mL/min over 0.01 minutes, changed to 0.03 mL/min over 1.39 minutes, then returned to 0.5 mL/min over 0.01 minutes, and held for 0.29 minutes. MRM transitions and compound-dependent parameters (including declustering potential (DP), entrance potential (EP), collision energy (CE), and collision cell exit potential (CXP)) for each tested compound were selected using Discovery Quant Software (version 3.0.2). The temperature was set at 300 °C, employing high collision gas, and using a 30 µL injection volume. The two most abundant fragments of the parent ion were documented. Scan step size was 0.1 amu, with Q3 scan at 200 amu/s. Compounds were acquired in positive mode, and negative mode if required. Data acquisition was performed with Analyst 1.7.3 (SCIEX).

1.3 Neutral Loss-Acoustic Droplet Ejection-Mass Spectrometry (NL-ADE-MS)

Samples were prepared in 384-well PP plates (001-14615) with 50 µL of DMSO/H_2O (*v:v* = 70:30). Acoustic transfer of solutions from 384-well LDV echo plates (001-12782) was the typical method used to generate these samples from quenched reaction mixtures (12.5 mM). Prior to loading into the NL-ADE-MS system, the source microplates containing the sample cosolvent underwent centrifugation (5 minutes at 2000 rpm) to eliminate gas bubbles and ensure a uniform fluid meniscus shape. Sample collection was facilitated using the Sciex OS-MQ

Analytics Software (version 2.1.6.59781) from Sciex (Forster City, CA), to control an AE-MS system operating in neutral loss mode. This system comprised an externalized transducer assembly from an AE-MS autosampler, an open port interface (OPI) connected to a carrier solvent pump, and a transfer capillary leading to a standard IonDrive Turbo V ESI source of an AB Sciex Triple Quad 6500+ system. The carrier liquid utilized was methanol supplemented with 1 mM ammonium fluoride, flowing at a rate of 350 µL/min, generating a stable vortex at the OPI inlet for optimized signal performance. Contactless sampling involved ejecting of 2.5 nL directly from the microtiter plate wells into the carrier liquid vortex of the OPI, at a frequency of 1.2 seconds per well. The electrospray ionization (ESI) source of the triple quadrupole MS instrument operated in positive ionization mode, with nebulizer gas (GS1) set to 90 psi, heater gas (GS2) to 70 psi, curtain gas and collision-activated dissociation (CAD) gas to 35 psi and 9 units, respectively. The mass scan range was set according to samples being studied. For analyte measurements, the following MS parameters were employed: ion source temperature: 500 ˚C; spray voltage: 5500 V; pause time: 5 ms; Q1 operated at unit resolution. To simplify the test, NL-AE-MS methods were set up based on the average results from multiple reaction monitoring of the starting materials as shown in **Table S1,** included declustering potential (DP), entrance potential (EP), collision energy (CE), and collision cell exit potential (CXP). Specifically, NL of 111 Da, with CE, 31 V, DP, 89 V, EP, 10 V, CXP, 13 V was used for products that generated from building blocks **1-12**. NL of 139 Da, with CE, 24 V, DP, 93 V, EP, 10 V, CXP, 13 V was used for products that generated from building blocks **13-16**. After acquiring sample batches, data processing was conducted using an in-house-built R script we previously described[1].

Table S1 Selected Neutral Loss Data from Product Ion Scanning MS/MS for Building Blocks 1-16

Molecule	NL (Da)	CE (V)	DP (V)	EP (V)	CXP	Ref
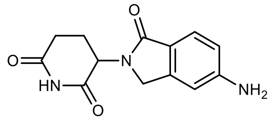 **1**	111	25	90	10	10	[1]
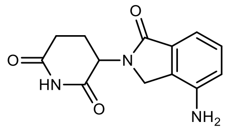 **2**	111	23	90	10	9	This work
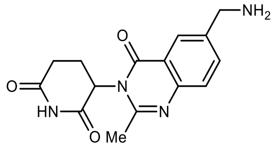 **3**	111	34	100	10	12	This work
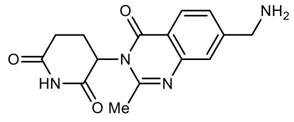 **4**	111	34	50	10	12	This work
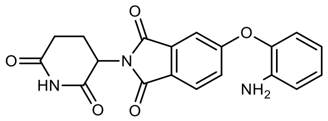 **5**	111	26	110	10	16	This work

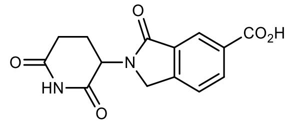 **6**	111	23	100	10	10	This work
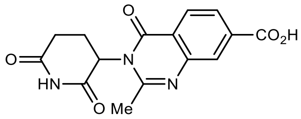 **7**	111	37	120	10	12	This work
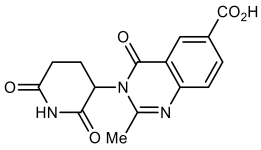 **8**	111	36	120	10	13	This work
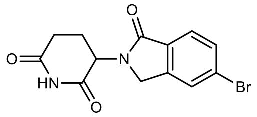 **9**	111	26	90	10	14	[1]
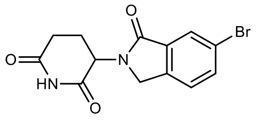 **10**	111	26	100	10	12	This work
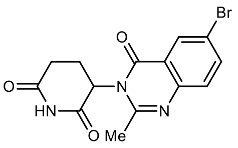 **11**	111	38	50	10	16	This work
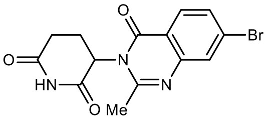 **12**	111	38	50	10	13	This work
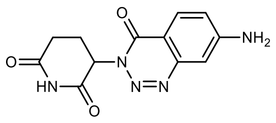 **13**	139	27	100	10	14	This work
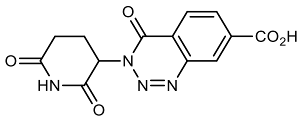 **14**	139	27	110	10	11	This work

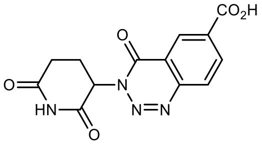 **15**	139	25	100	10	10	This work
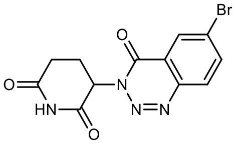 **16**	139	27	60	10	18	This work

1.4 Affinity Selection Mass Spectrometry (ASMS) System

The automated ligand identification system (ALIS) or affinity selection mass spectrometry (ASMS) instrument setup was adapted from the Annis et al.[2]. More specifically, an Agilent 2D-LC-qToF system controlled by Agilent 2D-LC software, consisting of a 1260 Multisampler with integrated sample chiller, 1260 Binary Pump (SEC solvent pump), 1290 High Speed Pump (Reverse phase solvent pump), 1290 multiple heart cutting valve with a 50 µL sample loop, two of 1260 Multicolumn Thermostat (one for the SEC column, one for the reverse phase column), 1260 Variable Wavelength Detector (VWD) with a high-pressure flow cell, 1290 Diode Array Detector (DAD), and 6530 qToF from Agilent Technologies (Santa Clara, CA, USA). Outflow from the size exclusion column was routed through the variable wavelength detector to trigger the heart cutting valve fractionating a single peak via the Agilent 2D-LC software, the diode array detector was placed in the waste flow line to validate successful peak capture. Note: Use of a high-pressure flow cell for the VWD upstream of the heart cutting valve prevents failure of the flow cell from repeated heart-cutting events. Samples were prepared in 384-well Greiner plates (Part 784201) in 10 or 20 µL volumes and placed in the multisampler at 4 °C. 3 µL of sample was injected to SEC column (Polyhydroxyethyl A, 60 Å, 5 µM, 50 x 2.1 mm) at 4 °C. 1.0 M ammonium acetate (NH_4OAc, A solvent) at pH 7.5 was used as eluent at flow rate of 1 mL/min. The protein peak was detected by variable wavelength detector at 280 nM. About 50 µL (~ 3s wide) of protein/ligand complex was captured in the sample loop triggered by detection of the eluting protein peak. Immediately after the SEC column was washed using B solvent containing acetonitrile/water (*v:v* = 50:50). Concurrently, the protein-ligand complex captured by the sample loop was directed to RPC column (Higgins Analytical, TARGA C_{18}, 5 µM, 50 x 0.5 mm) for separation and detection by high resolution MS. The RPC column was run at 60 °C with flow rate 0.1 mL/min using 0.1% FA in water (A solvent) and 0.1 % FA in acetonitrile (B solvent). Both the SEC and RPC columns were reconditioned prior to the next injection. The tables below show the specific gradients and timings.

Table S2 SEC Column Gradient Timetable

Time (min)	A (%)	B (%)	Flow rate (mL/min)	Max pressure limit (Bar)
0.00	100.0	0.0	1.00	600.0
0.50	100.0	0.0	1.00	600.0
0.60	0.0	100.0	0.50	600.0
2.60	0.0	100.0	0.50	600.0
2.70	100.0	0.0	1.00	600.0
6.00	100.0	0.0	1.00	600.0

7.50	100.0	0.0	1.00	600.0

Table S3 RPC Column Gradient Timetable

Time (min)	A (%)	B (%)	Flow rate (mL/min)	Max pressure limit (Bar)
0.00	100.0	0.0	0.10	600.0
0.50	100.0	0.0	0.10	600.0
2.50	90.0	10.0	0.10	600.0
3.50	90.0	10.0	0.10	600.0
4.00	0.0	100	0.10	600.0
7.50	0.0	100	0.10	600.0

1.5 High Throughput Reaction Coupling Partner Libraries

Carboxylic acid and amine coupling partners were selected from our St. Jude in house chemical collection. Chemicals were prepared as 1 mmol samples in 7 mL glass vials with screw caps, and transferred to an N_2 filled glovebox to make solutions. DMF (5 mL) was added in the glovebox to make a final 0.2 M stock. A thirty second to one minute vortex was used to dissolve them. The amidation reaction carboxylic acid library contained 323 acids. The amidation reaction amine library contained 514 amines. The Buchwald-Hartwig reaction amine library contains 227 aromatic amines. 50 µL of each building block was loaded into 384-well Greiner plates (Part 784201) and sealed in the glovebox that is ready for high throughput synthesis. Physiochemical parameters including molecular weight, hydrogen bond acceptors, hydrogen bond donors, quantitative estimate of drug-likeness, topological polar surface area, fraction of sp^3 carbons, partition coefficient, rotatable bonds, and number of rings of each library are summarized in **Supplementary Figure 12-14**.

1.6 ASMS Pooling

Pooling was based on the analysis from high throughput miniaturized reactions. The reactions with highest relative output by tandem mass spectrometry were selected this gave 4434 products from an expected 5643. Each selected compound was identified by its unique compound ID, plate barcode, and well location. Should two wells have equivalent maximum relative signal by tandem mass spectrometry then one of them was removed manually by checking the unique compound ID. Pools were made using a round-robin strategy to avoid exact mass overlap where the minimum difference between two small molecules had to be greater than or equal to 0.05 amu. This gave about 60 compounds per pool again indicated by their unique compound ID, plate barcode, and well locations. From these data an acoustic transfer list was generated to pool the crude mixtures. Alongside this transfer list an ASMS input list file also generated with unique compound ID and formula corresponding to contents of each well. 100 nL of crude reaction mixture of each compound was transferred from the original quenched reaction plate to make a pooled 384-well LDV echo compatible source plate for a total volume of about 6 µL per well across a total of 73 pools and which was sealed and stored at 4 °C. This plate became the source plate for ASMS experiments and was thawed before ASMS assays see **Section 1.8.5.** The mass distribution of each pool was shown in **Supplementary Figure 15**.

Custom code to provide custom compound pooling, diagnostic files, direction files for echo transfers and MS analysis files can be found at: https://github.com/J-M-Ochoada/cpd_pooling. This format was designed to work with the Agilent Mass Hunter PCDL and Quantitative Analysis modules.

1.7 Protein Purification

GSPT1 (domains 2 and 3: amino acids 437-633) was expressed and purified as previously described[3]. Briefly, MBP-TEV-GSPT1 was expressed in OneShot BL21 (DE3) Escherichia coli cells (Life Technologies) using 2XYT media (PR1MA). Cells were grown to an optical density OD600 of 0.7 and induced with 0.4 mM IPTG. Induced cells were grown overnight at 16 °C for 18 hours, shaking at 180 rpm. After overnight induction, cells were harvested by centrifugation at 5000xg for 20 min at 4 °C and pellets were flash frozen in liquid nitrogen. Cell pellets were resuspended in 50 mM Tris-HCl pH 7.5, 200 mM NaCl, 1 mM TCEP, 10% glycerol, and SigmaFast protease inhibitor cocktail (Millipore Sigma). MBP-TEV-GSPT1 cell pellets were lysed by sonication (2 second ON/2 second OFF, 10 minutes), clarified by centrifugation (136,000xg) for 1.5 hours at 4 °C, and loaded onto an MBP Trap HP column (Cytiva) equilibrated in 50 mM Tris-HCl pH 7.5, 200 mM NaCl, 1 mM TCEP and 10% glycerol. The MBP Trap column was washed with equilibration buffer, and MBP-TEV-GSPT1 was eluted with 50 mM Tris-HCl pH 7.5, 200 mM NaCl, 1 mM TCEP, 10% glycerol, and 10 mM maltose (Sigma). TEV protease (made in-house) was added to MBP-TEV-GSP1 fractions at a ratio of 1:25 (*v:v*) and incubated overnight at 4 °C. Cleaved mixture containing GSPT1, MBP, and TEV was loaded onto nickel resin (Goldbio) to capture TEV, and flow-through was collected and diluted to 90 mM NaCl for a final buffer of 50 mM Tris-HCl pH 7.5, 90 mM NaCl, 1 mM TCEP, and 10% glycerol. Diluted GSPT1 and MBP mixture was loaded onto a heparin column (Cytiva) equilibrated in 50 mM Tris-HCl pH 7.5, 90 mM NaCl, 1 mM TCEP, and 10% glycerol, and eluted using a linear salt gradient by mixing buffer A (50 mM Tris-HCl pH 7.5, 1 mM TCEP, 10% glycerol) and buffer B (50 mM Tris-HCl pH 7.5, 200 mM NaCl, 1 mM TCEP, 10% glycerol). GSPT1 fractions were concentrated using a 3-kDa cutoff concentrator (Millipore) and loaded onto a Superdex HiLoad 16/600 S75 gel filtration column (Cytiva) equilibrated in 50 mM HEPES pH 7.4, 200 mM NaCl, and 0.25 mM TCEP. GSPT1 fractions were concentrated using a 3-kDa cutoff concentrator (Millipore) and add 5% glycerol for storage.

CRBN-DDB1 was prepared following the reported procedure[3]. The purified protein was stored at – 80 °C with the final buffer containing 50 mM Tris-HCl, 500 mM NaCl, 10% glycerol with 500 mM imidazole, pH 7.5.

The GST-tagged human LCK construct (residues Gly2–Pro509, UniProt: P06239) was designed with the GST tag added to the N-terminus of LCK using a 'SDGGGGS' linker. The codon-optimized LCK gene fragment was synthesized and cloned into the pFastBac1 vector for expression in Sf9 insect cells. The protein was subsequently produced by Genscript. The purified protein was stored at – 80 °C in a final buffer contains 50 mM Tris-HCl, 500 mM NaCl, 5% glycerol, pH 8.0.

Human KRAS variants ($KRAS^{G12C}$ and $KRAS^{WT}$) was expressed and purified as previously described[4]. Briefly, human KRAS variants were cloned into a pET-28a(+) vector containing an N-terminal His_6-tag followed by a TEV protease cleavage site and expressed in E. coli BL21-CodonPlus (DE3)-RIPL cells. Transformed cells were cultured in modified LB medium (10 g Trptone, 10 g Yeast Extract, 10 g NaCl) supplemented with 50 µg mL^{-1} kanamycin, 34 µg mL^{-1} chloramphenicol, and 0.5% glucose at 37 °C, 200 rpm. Protein expression was induced at OD_{600} of 0.8–1.0 with 1 mM IPTG, followed by incubation overnight at 20 °C, 140 rpm. Harvested cells were lysed in 20 mM Tris-HCl pH 8.0, 500 mM NaCl, 2 mM β-mercaptoethanol, 5 mM $MgCl_2$, 5% glycerol, and protease inhibitor cocktail (Roche) with sonication. Lysate supernatant was applied to Ni^{2+}-NTA resin equilibrated in lysis buffer. The resin was washed sequentially with 10 mM imidazole and eluted with 300 mM imidazole. The eluate was incubated with 20-fold molar excess of GDP. TEV protease (1:25, w/w) was then added to the mixture for His_6-tag cleavage during overnight dialysis against 20 mM Tris-HCl pH 8.0, 300 mM NaCl, 1 mM DTT, 5 mM imidazole, and 0.5 mM EDTA at 4 °C. Cleaved KRAS was separated from uncleaved protein and TEV protease by reverse Ni^{2+}-affinity chromatography. For $KRAS^{WT}$, an additional purification step was performed using Mono S cation-exchange chromatography (Cytiva) with a linear 0.1–0.4 M NaCl gradient in 20 mM HEPES pH 6.5, 1 mM DTT. All protein variants were further purified by size-exclusion chromatography on a Superdex 75 Increase 10/300 column (Cytiva) equilibrated in 20 mM HEPES

pH 7.5, 150 mM NaCl, 1 mM DTT. Purified KRAS fractions were concentrated to 10 mg mL^{-1}, flash-frozen in liquid nitrogen, and stored at –80 °C. Before the ASMS assay, a nucleotide exchange experiment was performed to have a GTP form KRAS protein[5]. Protein concentrations were determined by Bradford assay, $KRAS^{G12C}$ and $KRAS^{WT}$ were diluted to approximately 150 µM in base buffer (20 mM HEPES pH 7.5, 150 mM NaCl, 1 mM DTT, 1 mM $MgCl_2$). Nucleotide exchange was initiated by transiently chelating Mg^{2+} with 10 mM EDTA (0.5 M stock), and a 10-fold molar excess of GTP (100 mM stock) was then added, and the samples were incubated for 1 hour at room temperature to allow nucleotide loading. Mg^{2+} was restored to a concentration of 20 mM using 1 M $MgCl_2$, resulting in around 10 mM free Mg^{2+} after EDTA chelation to support efficient nucleotide rebinding. $KRAS^{G12C}$ and $KRAS^{WT}$ samples were further buffer-exchanged by dialysis in base buffer (typically 1:1500–1:1700 overnight) to remove excess nucleotide and potential remaining EDTA. After dialysis, protein was quantified using the Bradford assay. This protocol resulted in GTP-loaded forms of both KRAS protein forms.

All proteins were aliquoted and stored in freezer at −80 °C in microtubes and thawed on the ice right before use.

1.8 ASMS Assays

In general, ligand(s) were transferred from a 384-well LDV Echo source plate by an Echo liquid handler into a 384-well Greiner plates (Part 784201) immediately before adding the desired protein samples. Pure ligands were stored in DMSO at 4 °C in 384-well LDV Echo plate and thawed in room temperature before transfer. Quenched crude reaction mixtures were stored at 4 °C in 384-well LDV Echo plate and thawed in room temperature before Echo transfers. Proteins were transferred by electronic multi-channel pipettes. After adding the compound(s), buffer, and protein together, the plate was sealed and mixed by centrifuge for 1 minutes at 1000 rpm. The plate was then incubated at room temperature in an orbital plate shaker for 30 minutes at 600 rpm. After centrifuging again for 1 minute at 1000 rpm, the plate was cooled down to 4 °C in the autosampler of the ASMS system for about 10 minutes before testing.

The SEC primary flow solvent (A solvent) 1.0 M NH_4OAc pH 7.5 was prepared and filtered freshly through 0.2 µM filter before use. HEPES pH 7.4 buffer was used to dilute protein, which contains 20 mM HEPES and 100 mM NaCl, was filtered through a 0.2 µM filter, and aliquoted to 1 mL per tube stored at −20 °C. Aliquots were thawed at 4 °C one day before use.

1.8.1 Enrichment Experiment using Neosubstrate GSPT1 (Figure 2a, b, c)

Three separate samples were prepared and the ligand signal by ASMS compared. To test the reproducibility of the sample preparation, each sample was made in triplicate.

CRBN-DDB1: **SJ6986** 20 µM, 0.002% Tween 20, 10 µM of CRBN-DDB1 in HEPES buffer.

GSPT1: **SJ6986** 20 µM ligand, 0.002% Tween 20, 10 µM of GSPT1 in HEPES buffer.

CRBN-DDB1/GSPT1: **SJ6986** 20 µM ligand, 0.002% Tween 20, 10 µM of CRBN-DDB1 and GSPT1 in HEPES buffer.

The same sample was repeated using **CC-220** in place of **SJ6986**.

1.8.2 Titration Experiment using Neosubstrate GSPT1 (Supplementary Figure 1a)

Individual samples were prepared for ASMS containing GSPT1 (0, 2.5, 5.0, 7.5, 10.0, 12.5, and 15 µM) and 20 µM **SJ6986**, 0.002% Tween 20, and 10 µM of CRBN-DDB1, in HEPES buffer. A control containing only neosubstrate, GSPT1 15 µM, 20 µM **SJ6986**, and 0.002% Tween 20, established no binding of **SJ6986** to the neosubstrate at the highest concentration tested in this experiment. The same workflow was repeated using **CC-220** in place of **SJ6986**.

1.8.3 Drug Like Compound Decoys vs CRBN-DDB1-GSPT1 Ternary Complex (Supplementary Figure 1b, c, d, e)

A pool containing 106 drug like compounds (see physiochemical properties in **Supplementary Figure 1b, c, d**) was pre-mixed in 384-well LDV Echo plate. 312 nL of this mixture was transferred by an Echo liquid handler, to give a 2 µM final concentration of each compound in the assay samples. Binary complex, CRBN-DDB1 sample contains 2 µM of 106 drug like compounds, 20 µM of **SJ6986**, 10 µM of CRBN-DDB1 in HEPES buffer. Ternary complex, CRBN-GSPT1 samples, contains 2 µM of 106 drug like compounds, 20 µM of **SJ6986**, 0.002% Tween 20, 10 µM of CRBN-DDB1 and GSPT1 in HEPES buffer.

1.8.4 Enrichment Experiment using CypA and KRAS (Figure 2d, e)

Three separate samples were prepared and the ligand signal by ASMS compared. To test the reproducibility of the sample preparation, each sample was made in triplicate.

CypA: **RMC-6236** (Daraxonrasib) 2.5 µM, 5 µM of CypA in HEPES buffer.

KRAS: **RMC-6236** (Daraxonrasib) 2.5 µM ligand, 5 µM of KRAS in HEPES buffer.

CypA/KRAS: **RMC-6236** (Daraxonrasib) 2.5 µM ligand, 5 µM of CypA and KRAS in HEPES buffer.

The same sample was repeated using **BI-2865** in place of **RMC-6236** (Daraxonrasib).

1.8.5 Crude Reactions Screening against CRBN-DDB1-LCK-GST (Figure 4a, b, d and Supplementary Figure 7)

Crude reaction screening was performed in three phase strategy. The first-round screening was carried out using ternary complex CRBN-DDB1-LCK-GST at 5 µM. Hits from first round were pooled and tested against CRBN-DDB1 (5 µM), LCK-GST (5 µM), and CRBN-LCK (5 µM). Enrichment of ligand signal in the presence of ternary complex (1.5 fold) was used to define hits for final follow up.-To generate the pools and singletons for rounds 2 and 3 the quenched reaction plates were stored at 4 °C in 384-well LDV Echo plate, thawed, and transferred to assay plates by Echo liquid handler (DMSO mode was used) right before making the ASMS samples. Hits from screening were retested by adding it in a different pool where there was no mass overlap for the target compound. Triplicate data were collected against CRBN-DDB1 (5 µM), and CRBN-LCK (5 µM).

Round 1: CRBN-DDB1-LCK-GST samples, containing 150 nL of pooled compounds, 0.002% Tween 20, 5 µM of CRBN-DDB1 and LCK-GST in HEPES buffer.

Round 2: Hits from round one were grouped into 22-23 compound pools using 2.5 nL per compound directly from the quenched reaction plates. Three samples were made and tested one after another for each pool:

CRBN-DDB1: 2.5 nL per ligand, 0.002% Tween 20, 5 µM of CRBN-DDB1 in HEPES buffer.

LCK-GST: 2.5 nL per ligand, 0.002% Tween 20, 5 µM of LCK-GST in HEPES buffer.

CRBN-DDB1-LCK-GST: 2.5 nL per ligand, 0.002% Tween 20, 5 µM of CRBN-DDB1 and LCK-GST in HEPES buffer.

1.8.6 Purified compounds enrichment experiment using neosubstrate LCK-GST (Figure 4c and Figure 5c)

Purified compounds were tested using an identical method to individual crude compounds.

CRBN-DDB1: 2.5 nL ligand, 0.002% Tween 20, 5 µM of CRBN-DDB1 in HEPES buffer.

LCK-GST: 2.5 nL ligand, 0.002% Tween 20, 5 µM of LCK-GST in HEPES buffer.

CRBN-DDB1/LCK-GST: 2.5 nL ligand, 0.002% Tween 20, 5 µM of CRBN-DDB1 and LCK-GST in HEPES buffer.

For **Figure 5c** LCK was replaced with GSPT1 in HEPES buffer.

1.8.7 Competition Experiment using Neosubstrate LCK-GST (Figure 5d)

Two sets of samples were prepared using either binary complex CRBN-DDB1 (5 µM) or ternary complex CRBN-DDB1/LCK-GST (5 µM), with 0.002% Tween 20 in HEPES buffer. Compound **23** (AUC_{rel} = 3.0, EC_{50} = 97 nM) was added to all the samples at 5 µM. A competing compound **42** (AUC_{rel} = 0.2, EC_{50} = 513 nM) was added in different amounts 5 µM (1 eq), 50 µM (10 eq), 125 µM (25 eq), 250 µM (50 eq), and 500 µM (100 eq) to interrogate change in ternary complex mediated affinity enrichment of **23**. Comparative experiments were performed with both 5 µM of CRBN-DDB1 in HEPES buffer and ternary complex and 5 µM of CRBN-DDB1/LCK-GST with 0.002% Tween 20 in HEPES buffer. Compounds and make-up DMSO volume were added by Echo liquid handler as below. Samples were run using a longer ASMS method, SEC: 0-1 min 100% A, 1-6 min gradient from 100% A to 100% B, hold at 100% B for 5 min, then, 11-16 min gradient from 100% B to 100% A. For RPC, 0-1.5 min 99% A, 1.5-3.5 min gradient to 99% B and hold for 1 min, then gradient to 1% B over 1.5 min.

Table S4 Volume of Ligands

**23** volume (nL)	**42** eq.	**42** volume (nL)	DMSO volume (nL)	Total DMSO volume (nL)
7.5	1	7.5	742.5	757.5
7.5	10	75	675	757.5
7.5	25	187.5	562.5	757.5
7.5	50	375	375	757.5
7.5	100	750	0.0	757.5

1.8.8 Ranking Experiment of Neosubstrate LCK-GST (Figure 5e)

The ranking experiment was conducted with 8 compounds (**18**, **21**, **25**, **32**, **37**, **38**, **39**, and **41**) which have distinguishable exact masses. A 9 µM protein sample with 5 µM of each ligand was prepared as a 40 µL volume to create a stock for serial dilution as shown in the **Table S5 Stock Concentration**. Protein was added last after additional compound transfers see below. The target wells for serial dilution were loaded with 10 nL of each ligand by Echo liquid transfer as well as HEPES buffer as shown in the **Table S5 Serial Dilution** table. Proteins were added and mixed by electronic pipette in A1 and A2, which was centrifuged for 1 minute at 1000 rpm. A 20 µL aliquot was transferred from A1 and A2 to B1 and B2 respectively and mixed by electronic pipette. Samples were centrifuged for 1 minute at 1000 rpm before next transfer. A 20 µL aliquot was transferred from B1 and B2 to C1 and C2 respectively, repeating the mixing and centrifuge steps. Then, a 20 µL aliquot was transferred from C1 and C2 to D1 and D2 respectively, repeating the mixing and centrifuge steps. A final serial dilution step was performed from wells D1 and D2 to E1 and E2 respectively. Here compounds concentration was maintained at 5 µM, but the protein concentration was serial diluted to obtain a series of concentrations 9, 4.5, 2.25, 1.125, 0.5625 µM. The final plate was then sealed and then incubated for test. The samples were tested by ASMS from lowest to highest protein concentration.

Table S5 Ranking Experiment

Stock Concentration (highest concentration) and wells

Well position	Per compound	HEPES buffer	CRBN	LCK	Protein concentration
A1	20 nL	31.3 µL	8.7 µL	0	9 µM
A2	20 nL	18.2 µL	8.7 µL	13.1 µL	9 µM

Serial Dilution concentration and wells

Well position	Ligand	HEPES buffer	CRBN	LCK	Expected protein concentration
B1	10 nL	20 µL	0	0	4.5 µM
B2	10 nL	20 µL	0	0	4.5 µM
C1	10 nL	20 µL	0	0	2.25 µM
C2	10 nL	20 µL	0	0	2.25 µM
D1	10 nL	20 µL	0	0	1.125 µM
D2	10 nL	20 µL	0	0	1.125 µM
E1	10 nL	20 µL	0	0	0.5625 µM
E2	10 nL	20 µL	0	0	0.5625 µM

1.9 Alphascreen Assay (Figure 4e, Figure 5a, b, e)

All reagents were diluted in assay buffer comprising 25 mM HEPES, pH 7.4, 100 mM NaCl, 0.1% BSA, and 0.05% Tween20. An ECHO 650 (Labcyte Inc.) acoustic dispenser was used to generate a 10-point dilution curve (0.51–10,000 nM final concentration) from DMSO stocks (0.51–10,000 µM) of the compounds directly into a 384-well OptiPlate (PerkinElmer, cat# 6007290) giving a final DMSO concentration of 0.1%. The assay mixture contained 100 nM His-tagged CRBN-DDB1, and 75 nM GST-tagged LCK. AlphaScreen glutathione coated donor and AlphaScreen nickel chelate acceptor beads were purchased from PerkinElmer (cat# 6765300 and 6760141 respectively). Briefly, to a 384-well OptiPlate containing 5× compound in triplicate was added 5 µL of a 5× solution of His-CRBN-DDB1 and GST-LCK and then incubated at rt for 1 h. After incubation, 10 µL nickel chelate acceptor (20 µg/mL final concentration) and 10 µL glutathione donor beads (20 µg/mL final concentration) were added under subdued lighting. The plate was sealed and mixed on a MixMate (eppendorf) for 1 h at room temperature and then luminescence detection was collected on an Envision plate reader (PerkinElmer). GraphPad Prism software (v10.3.1) was used to fit a four-parameter dose-response model and calculate the AUC.

1.10 HiBiT Assay (Figure 4f)

The compounds were prepared in 384-well plate in a dose-response format. 100 nL of each compound at 10 serial concentrations between 0.5081-10,000 µM (final concentrations being 2.03 - 40,000 nM) were transferred to the plates using acoustic liquid handler. KOPT-K1_LCK_HiBiT cells [PMID: 38973320] were used to quantify LCK degradation in vitro. The cells were suspended at the density of 300,000 cells/mL, and then 25µl (7,500 cells) of the suspension was plated into each well by a VIAFLO 384 electronic pipette (Integra). After 24 hours of incubation, the level of HiBiT tagged-LCK protein was evaluated by the Nano-Glo HiBiT lytic detection system (Promega, #N3030) according to the manufacturer’s instructions. The luminescence signal was

measured using a Synergy H4 Hybrid Microplate reader (BioTek). % LCK was determined by comparing the luminescence of cells treated with compounds with those treated with DMSO, subtracting the luminescence of KOPT-K1 parental cells treated with DMSO. Each compound was tested independently in triplicates. Both lines were cultured in RPMI-1640 (ThermoFisher, #11875093) containing 10% fetal bovine serum (FBS) at 37 °C with 5% CO_2.

1.11 Immunoblotting Experiment (Supplementary Figure 8)

Immunoblotting T-ALL cell lines (KOPT-K1 and Jurkat, 10% FBS RPMI1640) were incubated for one hour and then DMSO or each compound diluted in DMSO was added. After being treated for 24 hrs, cells were harvested, washed once with ice-cold PBS and lysed with RIPA Lysis and Extraction Buffer (Thermo Scientific, #89901) supplemented with protease and phosphatase inhibitor cocktail (Thermo Scientific, #78440). Protein lysates were incubated on ice with gentle shaking for 15 minutes before being centrifuged at 4°C, 14,000 rpm for 15 minutes. Supernatants were added to an equal volume of 2 × Laemmli sample buffer (BioRad, #1610737) supplemented with 2-mercaptoethanol (Bio-Rad, #1610710). Protein samples were heated at 98°C for 10 minutes before western blotting. Equal amounts of protein samples were separated by precast 4–15% Tris-glycine Mini-PROTEAN TGX gels (Bio-Rad, #4561086). Resolved proteins were transferred onto Immobilon-FL PVDF membranes (Millipore, #IPFL00010) for detection of LCK. The membranes were blocked with Intercept® (TBS) blocking buffer (LI-COR, #927-60001) for two hours at room temperature and then probed with primary antibodies at an optimal concentration in the same blocking buffer supplemented with 0.2% Tween 20 (Fisher BioReagents, #BP337-500) overnight at 4°C: anti-LCK (Cell Signaling Technology, #2657; 1:2000) and anti-GAPDH (Cell Signaling Technology, #2118; 1:5000) for loading controls. The membranes were then washed with TBS-T three times and incubated with the IRDye® 800CW goat anti-rabbit (LI-COR, #926-32211; 1:5000) and 680CW goat anti-mouse IgG secondary antibodies (LI-COR, #926-68070; 1:5000) in the blocking buffer with 0.2% Tween 20 and 0.02% sodium dodecyl sulfate at room temperature for two hours. Excessive antibodies were washed out with TBS-T, and the membranes were exposed in LI-COR Odyssey imaging system. Fluorescent intensity was quantified and analyzed with Image Studio software (lite version 5.2).

1.12 ASMS Hit Identification

Hits were identified use Mass Hunter by matching formula from the ASMS input list (with unique ID) via the parameters in **Table S6**. 547 hits were found in the first round, then, 5 hits were found in the second round. Enrichment fold was calculated by normalizing the MS signal to its corresponding CRBN samples.

Table S6 Hits Identification Parameters

Parameter	Value
Absolute peak area (counts)>=	10000
Formula matching match tolerance (ppm)	+/−10
Peak spectrum average scans (% of peak height)>	10
TOF spectra exclude if above (% of saturation)	10
Mass score	100
Isotope abundance score	60
Isotope spacing score	50
Retention time score	100
Expected data variation MS mass	2.0 mDa + 5.6 ppm

Expected data MS isotope abundance	7.5%
Ion species positive	+H
Ion species negative	+electron,−H

2. Synthesis

2.1 Synthesis of Building Blocks

Synthesis of building block (3)

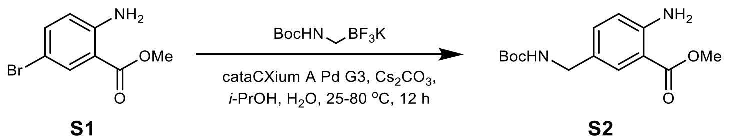

S1 **S2**

To a solution of methyl 2-amino-5-bromobenzoate **S1** (25.0 g, 108 mmol, 1.0 eq) in H_2O (80 mL) and *i*-PrOH (240 mL) were added Cs_2CO_3 (71.0 g, 216 mmol, 2.0 eq), potassium (tert-butoxycarbonylamino)methyl-trifluoro-boranuide (31.0 g, 130 mmol, 1.2 eq) and cataCXium A Pd G3 (8.0 g, 10.8 mmol, 0.1 eq) at 25 °C under N_2, then the mixture was stirred at 80 °C for 12 h. Reaction was monitored by LC-MS and product was confirmed. The reaction mixture was cooled to 0°C and quenched by addition saturated NH_4Cl aqueous solution (200 mL), then diluted with H_2O (200 mL) and extracted with EtOAc (200 mL × 3). The combined organic layers were washed with brine (200 mL x 3), dried over Na_2SO_4, filtered and concentrated under reduced pressure. The residue was purified by column chromatography (petroleum ether: EtOAc = 1:0 to 1:1) to give methyl methyl 2-amino-5-(((tert-butoxycarbonyl)amino)methyl)benzoate **S2** (26.0 g, 85% yield) as a white solid. ^{1}H NMR (400 MHz, DMSO-*d₆*) δ 7.60 (s, 1H), 7.24 (br s, 1H), 7.12-7.18 (m, 1H), 6.74 (d, *J* = 8.8 Hz, 1H), 6.58 (s, 2H), 3.96 (br d, *J* = 6.0 Hz, 2H), 3.81 (s, 3H), 1.39 (s, 9H).

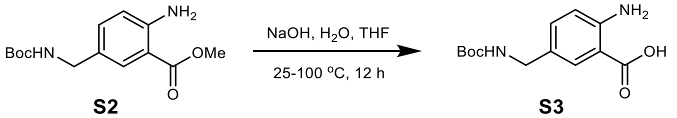

S2 **S3**

To a solution of methyl 2-amino-5-(((tert-butoxycarbonyl)amino)methyl)benzoate **S2**(13.0 g, 46.4 mmol, 1.0 eq) in THF (80 mL) and H_2O (20 mL) was added NaOH (5.6 g, 139 mmol, 3.0 eq) at 25 °C, then the mixture was stirred at 100 °C for 12 h. Reaction was monitored by LC-MS and product was confirmed. The mixture was concentrated to remove THF and H_2O (100 mL) was added to the solution. The mixture was extracted with EtOAc (70 mL × 3) then the aqueous phase was adjust pH to 5 by HCl (1 M). The aqueous phase was filtered and the filter cake was dried under reduced pressure to give 2-amino-5-(((tert-butoxycarbonyl)amino)methyl)benzoic acid **S3** (20 g, crude) as a white solid. ^{1}H NMR (400 MHz, DMSO-*d₆*) δ 7.87-9.11 (m, 2H), 7.59 (br s, 1H), 7.24 (br s, 1H), 7.13 (br d, *J* = 7.6 Hz, 1H), 6.70 (br d, *J* =8.4 Hz, 1H), 3.96 (br d, J = 5.6 Hz, 2H), 1.39 (s, 9H).

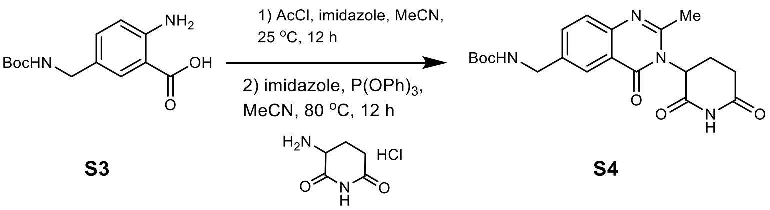

S3 **S4**

To a solution of 2-amino-5-(((tert-butoxycarbonyl)amino)methyl)benzoic acid **S3** (18.0 g, 67.6 mmol, 1.0 eq) in MeCN (50 mL) were added imidazole (5.5 g, 81.1 mmol, 1.2 eq) and acetyl chloride (5.8 g, 74.4 mmol, 5.3 mL, 1.1 eq) at 25 °C. The mixture was stirred at 25 °C for 12 h. Then to the reaction mixture were added 3-

aminopiperidine-2,6-dione hydrochloride (13.4 g, 81.2 mmol, 1.2 eq), imidazole (9.7 g, 142 mmol, 2.1 eq) and triphenyl phosphite (25.2 g, 81.1 mmol, 21.3 mL, 1.2 eq) at 25 °C. The mixture was stirred at 80 °C for 12 h. Reaction was monitored by LC-MS and product was confirmed. The mixture was concentrated to remove MeCN and H_2O (80 ml) was added to the solution. The reaction mixture was filtered and the filter cake was dried under reduced pressure. The crude product was triturated with EtOAc (100 mL × 3), then the solid was dried under reduced pressure to give tert-butyl ((3-(2,6-dioxopiperidin-3-yl)-2-methyl-4-oxo-3,4-dihydroquinazolin-6-yl)methyl)carbamate **S4** (11.3 g, 61% yield, 2 steps) as a yellow solid. ^{1}H NMR (400 MHz, DMSO-d_6) δ 11.04 (s, 1H), 7.88 (s, 1H), 7.70 (m, 1H), 7.64 (s, 2H), 5.29 (m, 1H), 4.14-4.30 (m, 2H), 2.87 (m, 1H), 2.56-2.71 (m, 5H), 2.09-2.27 (m, 1H), 1.40 (s, 9H).

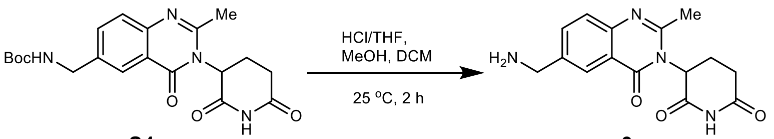

S4 **3**

To a solution of tert-butyl ((3-(2,6-dioxopiperidin-3-yl)-2-methyl-4-oxo-3,4-dihydroquinazolin-6-yl)methyl)carbamate **S4** (5.3 g, 13.2 mmol, 1.0 eq) in DCM (50 mL) and MeOH (50 mL) was added HCl/THF (2.0 M, 100 mL, 15.1 eq) at 25 °C, then the mixture was stirred at 25 °C for 2 h. Reaction was monitored by LC-MS and product was confirmed. The mixture was filtered and the filter cake was dried under reduced pressure. The crude product was recrystallized by methanol (3 L) at 70 °C, then the reaction mixture was filtered and the filter cake was dried under reduced pressure to give 3-(6-(aminomethyl)-2-methyl-4-oxoquinazolin-3(4H)-yl)piperidine-2,6-dione **3** (2.5 g, 54% yield, HCl) was obtained as a white solid. ^{1}H NMR (400 MHz, DMSO-d_6) δ 11.07 (s, 1H), 8.37 (br s, 2H), 8.18 (d, J= 2.1 Hz, 1H), 7.91 (m, 1H), 7.60 (d, J = 8.3 Hz, 1H), 5.31 (m, 1H), 4.17 (q, J = 5.9 Hz, 2H), 2.87 (m, 1H), 2.67 (s, 3H), 2.62-2.69 (m, 2H), 2.21 (m, 1H). ^{13}C NMR (126 MHz, DMSO-d_6) δ 173.1, 169.9, 160.7, 156.5, 147.0, 136.1, 132.9, 127.2, 127.1, 120.6, 57.2, 42.2, 31.1, 23.9, 21.4.

Synthesis of building block (4)

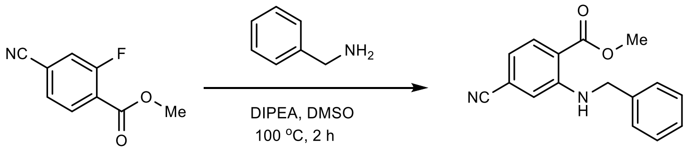

S5 **S6**

To a solution of methyl 4-cyano-2-fluorobenzoate **S5** (10.0 g, 55.8 mmol, 1.0 eq) in DMSO (100 mL) were added benzylamine (8.9 g, 83.7 mmol, 1.5 eq) and DIPEA (21.6 g, 167.5 mmol, 3.0 eq). The resulting mixture was stirred at 100 °C for 2 h. Reaction was monitored by LC-MS and product was confirmed. The mixture was cooled to room temp and diluted with H_2O (300 mL). The aqueous layer was extracted with EtOAc (100 mL × 3). The organics were dried over Na_2SO_4, concentrated and purified by column chromatography (petroleum ether: EtOAc = 1: 0 to 1: 1) to give methyl 2-(benzylamino)-4-cyanobenzoate **S6** (18.0 g, crude) as a yellow green solid.

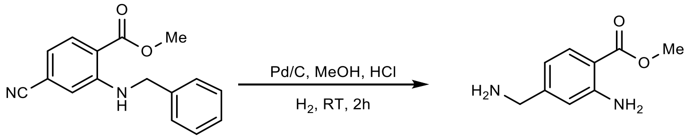

S6 **S7**

To a solution of methyl 2-(benzylamino)-4-cyanobenzoate **S6** (18.0 g, 67.6 mmol, 1.0 eq) in MeOH (100 mL) was added Pd/C (2.2 g, 20.3 mmol, 0.3 eq). The resulting mixture was stirred for 2 h at room temperature under H_2 atmosphere. To the solution was added HCl (30 mL) dropwise over 10 min at room temperature and the solution was stirred for additional 2 days at room temperature. Reaction was monitored by LC-MS and product was confirmed. The resulting mixture was filtered, the filter cake was washed with MeOH (100 mL × 3). The

filtrate was concentrated under reduced pressure. The residue was purified by column chromatography (DCM: MeOH = 1: 0 to 9: 1) to give methyl 2-amino-4-(aminomethyl)benzoate **S7** (9.1 g, 74%) as a white solid.

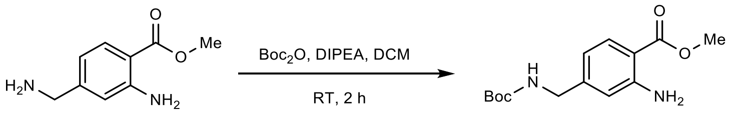

S7 **S8**

To a solution of methyl 2-amino-4-(aminomethyl)benzoate **S7** (9.0 g, 49.4 mmol, 1.0 eq) in DCM (100 mL) was added Boc_2O (10.9 g, 49.9 mmol, 1.0 eq) and DIPEA (6.4 g, 49.9 mmol, 1.0 eq). The reaction mixture stirred for 2 h at room temperature. Reaction was monitored by LC-MS and product was confirmed. The reaction was concentrated then purified by column chromatography (petroleum ether: EtOAc = 1:0 to 4:1) to give methyl 2-amino-4-(((tert-butoxycarbonyl)amino)methyl)benzoate **S8** (6.3 g, 45%) as a white solid.

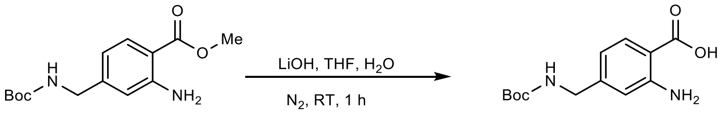

S8 **S9**

To a solution of methyl 2-amino-4-(((tert-butoxycarbonyl)amino)methyl)benzoate **S8** (6.3 g, 22.5 mmol, 1.0 eq) in THF/H_2O (4:1, 80 mL) was added LiOH (2.7 g, 112.4 mmol, 5.0 eq). The resulting mixture was stirred for 1 h at room temperature under nitrogen atmosphere. Reaction was monitored by LC-MS and product was confirmed.The mixture was adjusted pH value 5 with conc. HCl then the aqueous layer was extracted with EtOAc (100 mL × 3). The organic layers were dried over anhydrous Na_2SO_4, filtered, and concentrated to give 2-amino-4-(((tert-butoxycarbonyl)amino)methyl)benzoic acid **S9** (4.6 g, 77%) as a red solid.

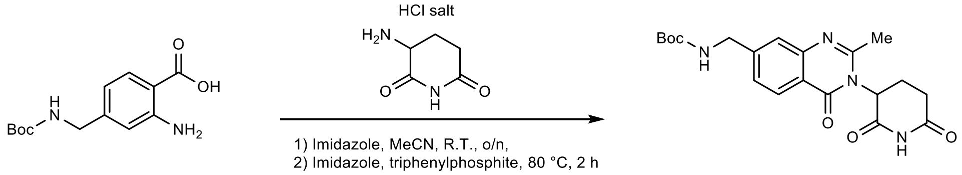

S9 **S10**

To a solution of 2-amino-4-(((tert-butoxycarbonyl)amino)methyl)benzoic acid **S9** (4.6 g, 17.3 mmol, 1.0 eq) in acetonitrile (20 mL) was added imidazole (1.1 g, 20.7 mmol, 1.2 eq) followed by acetyl chloride (1.6 g, 20.7 mmol, 1.2 eq) and the mixture was stirred at room temperature overnight. Reaction was monitored by LC-MS and the intermediate was confirmed. To the mixture was added 3-aminopiperidine-2,6-dione (2.2 g, 17.3 mmol, 1.0 eq), imidazole (2.4 g, 34.5 mmol, 2.0 eq) and triphenyl phosphite (6.4 g, 20.7 mmol, 1.2 eq) and the mixture was heated to reflux for 6 h. Reaction was monitored by LC-MS and product was confirmed. The mixture was concentrated then purified by column chromatography (DCM: MeOH = 1: 0 to 9: 1) to give tert-butyl ((3-(2,6-dioxopiperidin-3-yl)-2-methyl-4-oxo-3,4-dihydroquinazolin-7-yl)methyl)carbamate **S10** (2.1 g, 30%) as a white solid.

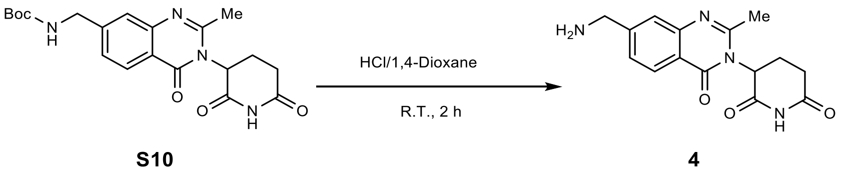

S10 **4**

To a vial containing tert-butyl ((3-(2,6-dioxopiperidin-3-yl)-2-methyl-4-oxo-3,4-dihydroquinazolin-7-yl)methyl)carbamate **S10** (2.1 g, 5.2 mmol, 1.0 eq) was added 4 M HCl in Dioxane (20 mL). The resulting mixture was stirred for 2 h at room temperature. Reaction was monitored by LC-MS and product was confirmed. The resulting mixture was concentrated under vacuum The filtrate was concentrated then taken up in DMSO and purified by preparative liquid chromatography Waters purification/analytical LC/UV/ELSD system

(0.1% formic acid in MilliQ H_2O: 0.1% formic acid in acetonitrile = 95:5 to 10:90). Desired fractions were concentrated to give 3-(7-(aminomethyl)-2-methyl-4-oxoquinazolin-3(4H)-yl)piperidine-2,6-dione **4** (1.5 g, 92%) as an off-white solid. ^{1}H NMR (600 MHz, DMSO-d_6) δ 11.06 (s, 1H), 8.44 (s, 2H), 8.07 (d, J = 8.2 Hz, 1H), 7.75 (s, 1H), 7.58 (dd, J = 8.5, 2.8 Hz, 1H), 5.30 (dd, J = 11.7, 5.7 Hz, 1H), 4.22 (q, J = 5.9 Hz, 2H), 2.86 (m, 1H), 2.66 (s, 3H), 2.57-2.72 (m, 2H), 2.19 (m, 1H). ^{13}C NMR (150 MHz, DMSO-d_6) δ 173.1, 170.0, 160.7, 156.3, 147.3, 141.4, 127.2, 126.9, 126.7, 120.4, 57.1, 42.3, 31.1, 24.0, 21.4.

Synthesis of building block (5)

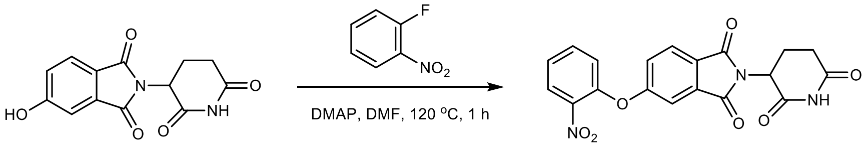

S11 S12

To a solution of 2-(2,6-dioxopiperidin-3-yl)-5-hydroxyisoindoline-1,3-dione **S11** (2.0 g, 7.3 mmol, 1.0 eq) in DMF (20 mL) were added o-fluoronitrobenzene (1.2 g, 8.8 mmol, 1.2 eq) and DMAP (1.1 g, 8.8 mmol, 1.2 eq) at room temperature. The resulting mixture was stirred for 1 h at 120 °C under nitrogen atmosphere. Reaction was monitored by LC-MS and product was confirmed. The reaction was quenched by the addition of H_2O (50 mL). The aqueous layer was extracted with DCM (50 mL × 3). The organics were concentrated and purified by column chromatography (Petroleum ether: EtOAc = 1:0 to 1:1) to give 2-(2,6-dioxopiperidin-3-yl)-5-(2-nitrophenoxy)isoindoline-1,3-dione **S12** (1.7 g, 59%) as a brown solid.

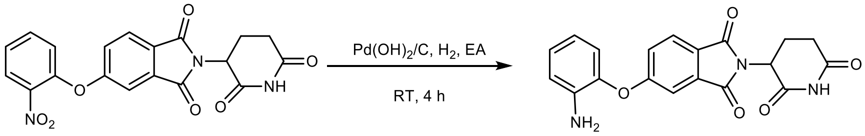

S12 5

To a solution of 2-(2,6-dioxopiperidin-3-yl)-5-(2-nitrophenoxy)isoindoline-1,3-dione **S12** (1.7 g, 4.3 mmol, 1.0 eq) in EtOAc (40 mL) was added $Pd(OH)_2$/C (0.17 g, 1.2 mmol, 0.28 eq) at room temperature. The resulting mixture was stirred for 4 h at room temperature under H_2 atmosphere. Reaction was monitored by LC-MS and product was confirmed. The mixture was filtered and the filtrate was concentrated under vacuum to give 5-(2-aminophenoxy)-2-(2,6-dioxopiperidin-3-yl)isoindoline-1,3-dione **5** (1.5 g, 96%) as a yellow solid. ^{1}H NMR (600 MHz, DMSO-d_6) δ 11.12 (s, 1H), 7.90 (d, J = 8.3 Hz, 1H), 7.29 (dd, J = 8.3, 2.3 Hz, 1H), 7.17 (d, J = 2.3 Hz, 1H), 7.05 (td, J = 7.6, 1.5 Hz, 1H), 6.97 (dd, J = 7.9, 1.5 Hz, 1H), 6.87 (dd, J = 8.0, 1.5 Hz, 1H), 6.63 (td, J = 7.6, 1.6 Hz, 1H), 5.13 (m, 1H), 5.11 (s, 2H), 2.88 (m, 1H), 2.51-2.64 (m, 2H), 2.00-2.08 (m, 1H). ^{13}C NMR (150 MHz, DMSO-d_6) δ 173.2, 170.3, 167.2, 167.1, 163.8, 141.4, 139.9, 134.2, 127.2, 126.1, 124.7, 122.2, 122.0, 117.0, 116.8, 110.7, 49.5, 31.4, 22.5.

Synthesis of building block (7)

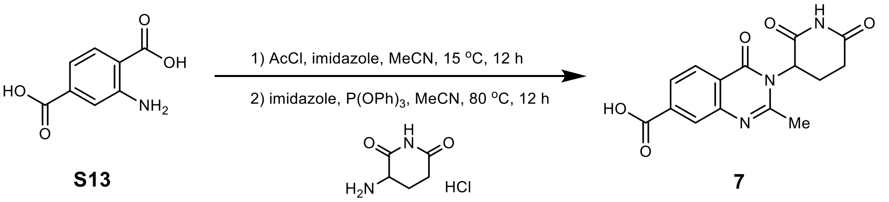

S13 7

To a solution of 2-aminoterephthalic acid **S13** (10.0 g, 55.2 mmol, 1.0 eq) in acetonitrile (100 mL) were added imidazole (4.5 g, 66.2 mmol, 1.2 eq) and acetyl chloride (4.7 mL, 66.2 mmol, 1.2 eq) at 15 °C. The mixture was

stirred at 15 °C for 12 h. Reaction was monitored by LC-MS and intermediate was confirmed. To the reaction mixture were added 3-aminopiperidine-2,6-dione (10.9 g, 66.2 mmol, 1.2 eq, HCl), imidazole (8.3 g, 121.4 mmol, 2.2 eq) and triphenyl phosphite (14.5 mL, 55.2 mmol, 1.0 eq) at 15 °C. The mixture was stirred at 80 °C for 12 h. Reaction was monitored by LC-MS and was confirmed. The reaction mixture was quenched by H_2O (200 mL) at 15 °C, and then extracted with EtOAc (200 mL× 3). The organics was concentrated then taken up in DMSO and purified by the Waters purification/analytical LC/UV/ELSD system (0.1% formic acid in MilliQ H_2O: 0.1% formic acid in acetonitrile = 95:5 to 10:90). Desired fractions were concentrated to give 3-(2,6-dioxopiperidin-3-yl)-2-methyl-4-oxo-3,4-dihydroquinazoline-7-carboxylic acid **7** (2.2 g, 12% yield) as a white solid. ^{1}H NMR (600 MHz, DMSO-d_6) δ 13.53 (s, 1H), 11.07 (s, 1H), 8.14 (d, J = 8.2 Hz, 1H), 8.10 (d, J = 1.5 Hz, 1H), 7.98 (dd, J = 8.3, 1.6 Hz, 1H), 5.32 (dd, J = 11.7, 5.6 Hz, 1H), 2.86 (m, 1H), 2.67 (s, 3H), 2.57-2.72 (m, 2H), 2.19 (m, 1H). ^{13}C NMR (150 MHz, DMSO-d_6) δ 173.1, 169.9, 166.9, 160.6, 156.6, 147.2, 136.8, 128.2, 127.1, 126.8, 123.6, 57.2, 31.1, 24.0, 21.3.

Synthesis of building block (8)

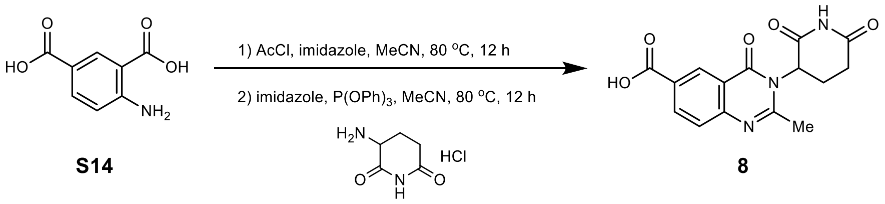

To a solution of 4-aminoisophthalic acid **S14** (5.0 g, 27.6 mmol, 1.0 eq) in acetonitrile (50 mL) were added imidazole (3.8 g, 55.2 mmol, 2.0 eq) and acetyl chloride (3.9 mL, 55.2 mmol, 2 eq) at 15 °C. The mixture was stirred at 15 °C for 12 h. Reaction was monitored by LC-MS and intermediate was confirmed. To the reaction mixture were added 3-aminopiperidine-2,6-dione (9.1 g, 55.2 mmol, 2.0 eq, HCl), imidazole (5.6 g, 82.8 mmol, 3.0 eq) and triphenyl phosphite (14.5 mL, 55.2 mmol, 2.0 eq) at 15 °C. The mixture was stirred at 80 °C for 12 h. Reaction was monitored by LC-MS and was confirmed. The reaction mixture was filtered and the solid was washed with acetonitrile (50 mL × 3), then was dried under reduced pressure to give a residue. The crude product was triturated with 1 N HCl (50 mL) 15 ºC for 30 min to give 3-(2,6-dioxopiperidin-3-yl)-2-methyl-4-oxo-3,4-dihydroquinazoline-6-carboxylic acid **8** (3.0 g, 33% yield) as a white solid. ^{1}H NMR (600 MHz, DMSO-d_6) δ 13.30 (s, 1H), 11.08 (s, 1H), 8.58 (d, J = 2.0 Hz, 1H), 8.29 (dd, J = 8.5, 2.1 Hz, 1H), 7.71 (d, J = 8.5 Hz, 1H), 5.32 (dd, J = 11.7, 5.7 Hz, 1H), 2.86 (m, 1H), 2.68 (s, 3H), 2.68 (m, 1H), 2.58-2.61 (m, 1H), 2.17-2.25 (m, 1H). ^{13}C NMR (150 MHz, DMSO-d_6) δ 173.1, 169.9, 166.7, 160.7, 157.9, 150.2, 135.1, 129.0, 128.4, 127.5, 120.5, 57.2, 31.1, 24.2, 21.3.

Synthesis of building block (11)

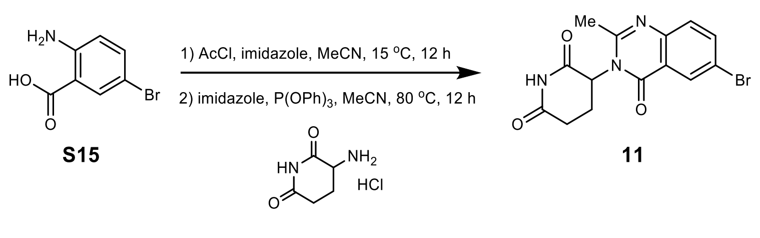

To a solution of 2-amino-5-bromobenzoic acid **S15** (5.0 g, 23.2 mmol, 1.0 eq) in acetonitrile (200 mL) were added imidazole (1.9 g, 27.8 mmol, 1.209 eq) and AcCl (2.0 mL, 27.8 mmol, 1.2 eq) at 15 °C. The mixture was stirred at 15 °C for 12 h. Reaction was monitored by LC-MS and intermediate was confirmed. Then to the reaction mixture were added 3-aminopiperidine-2,6-dione (4.6 g, 27.8 mmol, 1.2 eq, HCl), imidazole (3.5 g, 51.1 mmol, 2.2 eq) and triphenyl phosphite (6.1 mL, 23.2 mmol, 1.0 eq) at 15 °C. The mixture was stirred at 80 °C for 12 h. Reaction was monitored by LC-MS and product was confirmed. To the reaction mixture was added H_2O (100 mL) was added into the reaction mixture, the mixture was filtered and the filter cake was dried in vacuum. The

crude product was triturated with EtOAc (100 mL), MeOH (100 mL) and THF (40 mL) at 15 °C for 1 h to give 3-(6-bromo-2-methyl-4-oxoquinazolin-3(4H)-yl)piperidine-2,6-dione **11** (2.8 g, 35% yield) as a white solid. ^{1}H NMR (600 MHz, DMSO-d_6) δ 11.07 (s, 1H), 8.13 (d, *J* = 2.3 Hz, 1H), 7.98 (dd, *J* = 8.7, 2.3 Hz, 1H), 7.59 (d, *J* = 8.7 Hz, 1H), 5.30 (dd, *J* = 11.4, 5.6 Hz, 1H), 2.78-2.90 (m, 1H), 2.64 (s, 3H), 2.57-2.70 (m, 2H), 2.16-2.21 (m, 1H). ^{13}C NMR (150 MHz, DMSO-d_6) δ 173.1, 169.8, 159.9, 156.3, 146.3, 138.1, 129.5, 128.5, 122.3, 119.4, 57.2, 31.1, 24.0, 21.3.

Synthesis of building block (12)

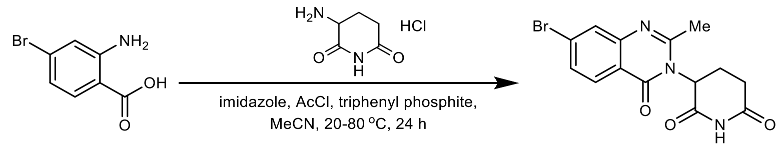

S16 **12**

To a solution of 2-amino-4-bromobenzoic acid **S16** (15.0 g, 69.4 mmol, 1.0 eq) in acetonitrile (150 mL) were added imidazole (5.7 g, 83.3 mmol, 1.2 eq) and acetyl chloride (5.9 mL, 83.3 mmol, 1.2 eq) at 20 °C. The mixture was stirred at 20 °C for 12 h. Reaction was monitored by LC-MS and intermediate was confirmed. Then to the reaction mixture were added 3-aminopiperidine-2,6-dione;hydrochloride (16.8 g, 83.3 mmol, 1.2 eq, HCl), imidazole (10.4 g, 153 mmol, 2.2 eq) and triphenyl phosphite (18.2 mL, 69.4 mmol, 1.0 eq) at 20 °C. The mixture was stirred at 80 °C for 12 h. Reaction was monitored by LC-MS and product was confirmed. The reaction mixture was concentrated under reduced pressure. The crude product was triturated with MeOH:THF (1:1, 1 L) at 80 °C for 30 min. The mixture was filtered and dried under vacuum to give 3-(7-bromo-2-methyl-4-oxoquinazolin-3(4H)-yl)piperidine-2,6-dione **12** (11.5 g, 46% yield) as a white solid. ^{1}H NMR (600 MHz, DMSO-d_6) δ 11.06 (s, 1H), 7.96 (d, *J* = 8.5 Hz, 1H), 7.86 (d, *J* = 1.8 Hz, 1H), 7.68 (dd, *J* = 8.5, 1.9 Hz, 1H), 5.29 (dd, *J* = 11.5 5.6 Hz, 1H), 2.76-2.90 (m, 1H), 2.65 (s, 3H), 2.56-2.64 (m, 2H), 2.14-2.23 (m, 1H). ^{13}C NMR (150 MHz, DMSO-d_6) δ 173.1, 169.9, 160.5, 157.2, 148.4, 130.2, 129.3, 128.8, 128.6, 119.9, 57.2, 31.1, 24.0, 21.3.

Synthesis of building block (13)

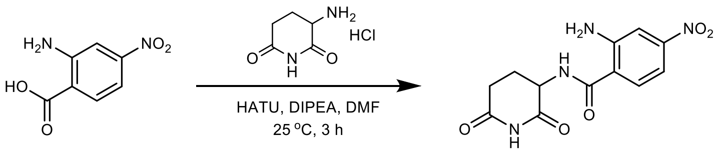

S17 **S18**

To a solution of 2-amino-4-nitrobenzoic acid **S17** (24.3 g, 133.6 mmol, 1.1 eq) in DMF (200 mL) was added HATU (50.8 g, 133.6 mmol 1.1 eq) and DIPEA (63.5 mL, 364.3 mmol, 3.0 eq),3-aminopiperidine-2,6-dione;hydrochloride (20 g, 121.4 mmol, 1.0 eq). The mixture was stirred at 25 °C for 3 h. Reaction was monitored by LC-MS and product was confirmed. The reaction was poured to ice water (400 mL), then the mixture was filtered and the filter cake was washed with H_2O (30 mL × 3). The cake was dried under vacuum to give 2-amino-N-(2,6-dioxopiperidin-3-yl)-4-nitrobenzamide **S18** (35.0 g, 90% yield) as a yellow solid. ^{1}H NMR (400 MHz, DMSO-d_6) δ 10.9 (s, 1 H), 8.8 (br d, *J* = 7.4 Hz, 1 H), 7.7 (d, *J* = 8.2 Hz, 1 H), 7.3 (d, *J* = 7.6 Hz, 1 H), 7.6 (s, 1 H), 6.8 (s, 2 H), 4.8 (s, 1 H), 2.7 - 2.8 (m, 1 H), 2.6 (s, 1 H), 2.1 (d, *J* = 10.8 Hz, 1 H), 2.0 (br s, 1 H).

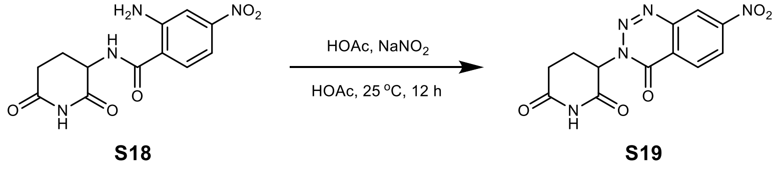

S18 **S19**

To a solution of 2-amino-N-(2,6-dioxopiperidin-3-yl)-4-nitrobenzamide **S18** (32.0 g, 109.5 mmol, 1.0 eq) in AcOH (300 mL) was added $NaNO_2$ (7.6 g, 109.5 mmol, 1.0 eq) and the mixture was stirred at 25 °C for 12 h. Reaction was monitored by LC-MS and product was confirmed. To the mixture was added H_2O (300 ml), the mixture was filtered and the filter cake was washed with of H_2O (30 mL × 3), The solid was dried under vacuum to give 3-(7-nitro-4-oxobenzo[d][1,2,3]triazin-3(4H)-yl)piperidine-2,6-dione **S19** (25.0 g, 75% yield) as a yellow solid. ^{1}H NMR (400 MHz, DMSO-d_6) δ 11.1 (s, 1 H), 9.0 (s, 1 H), 8.4 - 8.8 (m, 2 H), 6.0 (d, J = 7.2 Hz, 1 H), 3.0 (d, J = 13.8 Hz, 1 H), 2.7 (d, J = 13.6 Hz, 2 H), 2.3 (s, 1 H).

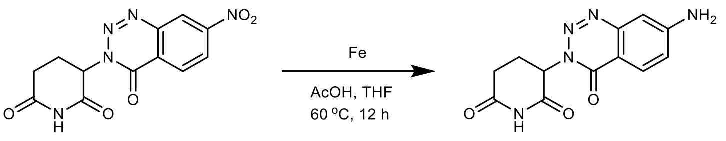

To a solution of 3-(7-nitro-4-oxobenzo[d][1,2,3]triazin-3(4H)-yl)piperidine-2,6-dione **S19** (10.0 g, 33.0 mmol, 1.0 eq) in AcOH (250 mL) and THF (250 mL) was added Fe (10.0 g, 179.0 mmol, 5.4 eq). The mixture was stirred at 60 °C for 12 h. Reaction was monitored by LC-MS and product was confirmed. The reaction mixture was filtered and the filter cake was washed with EtOAc (100 mL × 3) then dried under vacuum to give a solid. The residue was taken up in DMSO and purified by the Waters purification/analytical LC/UV/ELSD system (0.1% formic acid in MilliQ H_2O: 0.1% formic acid in acetonitrile = 95:5 to 10:90). Desired fractions were concentrated to give 3-(7-amino-4-oxobenzo[d][1,2,3]triazin-3(4H)-yl)piperidine-2,6-dione **13** (5.0 g, 55% yield) was obtained as a yellow solid. ^{1}H NMR (600 MHz, DMSO-d_6) δ 7.90 (d, J = 8.5 Hz, 1H), 7.10 (dd, J = 8.6, 2.2 Hz, 1H), 7.07 (d, J = 2.2 Hz, 1H), 6.65 (s, 2H), 5.86 (dd, J = 12.1, 5.3 Hz, 1H), 2.90-2.99 (m, 1H), 2.59-2.71 (m, 2H), 2.18-2.24 (m, 1H). ^{13}C NMR (150 MHz, DMSO-d_6) δ 173.2, 170.5, 155.9, 154.6, 146.4, 126.6, 120.7, 107.7, 107.3, 58.4, 31.2, 23.3.

Synthesis of building block (14)

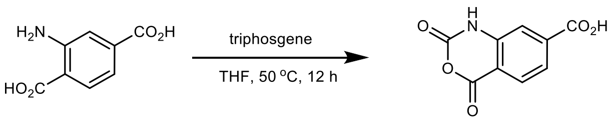

To a solution of 2-aminoterephthalic acid **S20** (10 g, 55.2 mmol, 1.0 eq) in THF (150 mL) was added triphosgene (26.5 g, 89.3 mmol, 1.6 eq). The mixture was stirred at 50 °C for 12 hr. Reaction was monitored by LC-MS and product was confirmed. The reaction mixture was poured into iced-water (100 mL) and stirred for 5 min. The organic layer was isolated and the aqueous layer was extracted with DCM (100 mL × 3). The combined organic layers were washed with 1 N HCl (100 mL) and brine (100 mL), dried over Na_2SO_4, filtered and concentrated under reduced pressure to give 2,4-dioxo-1,4-dihydro-2H-benzo[d][1,3]oxazine-7-carboxylic acid **S21** (11.0 g, 97 % yield) was obtained as a brown solid. ^{1}H NMR (400 MHz, DMSO-d_6) δ 11.89 (s, 1 H), 8.00 (d, J = 8.2 Hz, 1 H), 7.62 - 7.76 (m, 2 H).

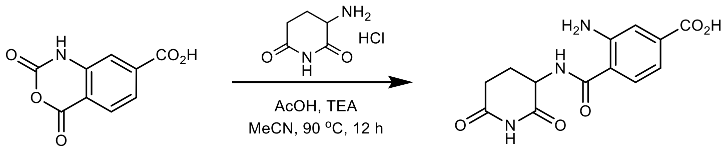

To a solution of 2,4-dioxo-1,4-dihydro-2H-benzo[d][1,3]oxazine-7-carboxylic acid **S21** (5.0 g, 24.1 mmol, 1.0 eq), in ACN (100 mL) was added 3-aminopiperidine-2,6-dione;hydrochloride (4.0 g, 24.1 mmol, 1.0 eq, HCl),TEA (16.8 mL, 120.7 mmol, 5.0 eq) and AcOH (13.8 mL, 241.4 mmol, 10.0 eq). The mixture was stirred at 90 °C for 12 h. Reaction was monitored by LC-MS and product was confirmed. The mixture was filtered, the filtrate was concentrated to give a crude 3-amino-4-((2,6-dioxopiperidin-3-yl)carbamoyl)benzoic acid **S22** (7.0 g, 99% yield) was obtained as a yellow oil. ^{1}H NMR (400 MHz, DMSO-d_6) δ 10.92 (br s, 1 H), 8.70 (d, *J* = 8.2 Hz, 1 H), 7.63 (d, *J* = 8.2 Hz, 1 H), 7.13 (dd, *J* = 8.2, 1.4 Hz, 1 H), 3.07 (q, *J* = 7.3 Hz, 1 H), 2.76 - 2.93 (m, 1 H), 2.57 - 2.66 (m, 2 H), 2.19 (qd, *J* = 12.8, 4.3 Hz, 1 H), 1.97 - 2.09 (m, 1 H).

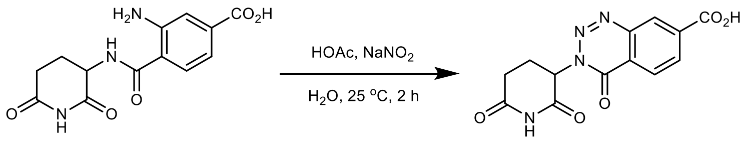

S22 **14**

To a solution of 3-amino-4-((2,6-dioxopiperidin-3-yl)carbamoyl)benzoic acid **S22** (5.0 g, 17.2 mmol, 1.0 eq) in AcOH (1 mL) was added $NaNO_2$ (1.9 g, 25.8 mmol, 1.5 eq). The mixture was stirred at 25 °C for 12 hr. Reaction was monitored by LC-MS and product was confirmed. The mixture was filtered and the filtrate was concentrated to give 3-(2,6-dioxopiperidin-3-yl)-4-oxo-3,4-dihydrobenzo[d][1,2,3]triazine-7-carboxylic acid **14** (2.6 g, 49% yield) as a yellow solid. ^{1}H NMR (600 MHz, DMSO-d_6) δ 11.22 (s, 1H), 8.61 (d, *J* = 1.5 Hz, 1H), 8.41 (dd, *J* = 8.2, 1.5 Hz, 1H), 8.33 (d, *J* = 8.2, 1H), 6.01 (dd, *J* = 12.4, 5.4 Hz, 1H), 2.91-3.03 (m, 1H), 2.65-2.77 (m, 2H), 2.27-2.33 (m, 1H). ^{13}C NMR (150 MHz, DMSO-d_6) δ 173.1, 172.5, 170.1, 166.3, 154.8, 143.8, 133.6, 129.1, 125.5, 121.4, 59.1, 31.2, 23.1.

Synthesis of building block (15)

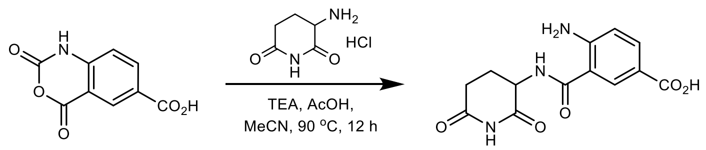

S23 **S24**

To a solution of 2,4-dioxo-1,4-dihydro-2H-benzo[d][1,3]oxazine-6-carboxylic acid **S23** (4.3 g, 20.8 mmol, 1.0 eq) in acetonitrile (60 mL) was added 3- aminopiperidine-2, 6-dione; hydrochloride (3.4 g, 20.8 mmol, 1.0 eq), TEA (14.5 mL, 103.8 mmol, 5.0 eq)and AcOH (11.9 mL, 207.6 mmol, 10.0 eq) and the reaction was stirred at 90 °C for 12 h. Reaction was monitored by LC-MS and product was confirmed. The reaction was concentrated and the residue was taken up in DMSO and purified by the Waters purification/analytical LC/UV/ELSD system (0.1% formic acid in MilliQ H_2O: 0.1% formic acid in acetonitrile = 95:5 to 10:90) to give 4-amino-3-((2,6-dioxopiperidin-3-yl)carbamoyl)benzoic acid **S24** (4.1 g, 64% yield) as a yellow solid. ^{1}H NMR (400 MHz, DMSO-d_6) δ 12.05 - 12.55 (m, 1 H), 10.85 (s, 1 H), 8.76 (d, *J* = 8.4 Hz, 1 H), 8.20 (d, *J* = 1.8 Hz, 1 H), 7.71 (dd, *J* = 8.8, 1.9 Hz, 1 H), 7.14 (s, 2 H), 6.74 (d, *J* = 8.6 Hz, 1 H), 4.65 - 4.86 (m, 1 H), 2.71 - 2.87 (m, 1 H), 2.55 (d, *J* = 3.5 Hz, 1 H), 2.13 (qd, *J* = 12.9, 4.4 Hz, 1 H), 1.87 - 2.01 (m, 1 H).

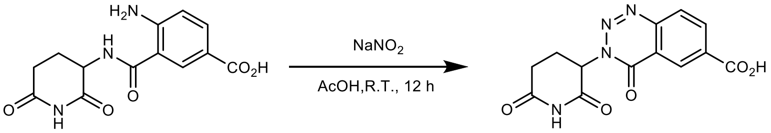

S24 **15**

To a solution of 4-amino-3-((2,6-dioxopiperidin-3-yl)carbamoyl)benzoic acid **S24** (2.2 g, 7.6 mmol, 1.0 eq) in AcOH (40 mL) was added $NaNO_2$ (1.3 g, 18.9 mmol, 2.5 eq) and was stirred at 25 °C for 12 h. Reaction was monitored by LC-MS and product was confirmed. To the reaction mixture was added H_2O (50 mL), then the mixture was filtered and the filter cake was washed with DCM (50 mL) and dried under vacuum to give 3-(2,6-dioxopiperidin-3-yl)-4-oxo-3,4-dihydrobenzo[d][1,2,3]triazine-6-carboxylic acid **15** (1.3 g, 57% yield) as a yellow solid. ^1H NMR (600 MHz, DMSO-d_6) δ 13.86 (bs, 1H), 11.24 (s, 1H), 8.72 (d, J = 1.8 Hz, 1H), 8.57 (dd, J = 8.4, 1.9 Hz, 1H),8.37 (d, J = 8.5 Hz, 1H), 6.05 (dd, J = 12.5, 5.4 Hz, 1H), 2.93-3.02 (m, 1H), 2.65-2.76 (m, 2H), 2.27-2.33 (m, 1H). ^{13}C NMR (150 MHz, DMSO-d_6) δ 173.1, 170.1, 166.0, 154.8, 145.5, 136.1, 135.1, 129.4, 126.5, 119.8, 59.1, 31.2, 23.1.

Synthesis of building block (16)

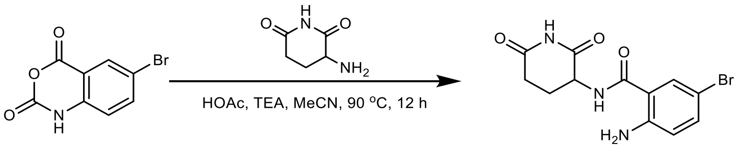

S25 **S26**

To a solution of 6-bromo-2H-benzo[d][1,3]oxazine-2,4(1H)-dione **S25** (5.0 g, 20.7 mmol, 1.0 eq) in acetonitrile (50 mL) were added 3-aminopiperidine-2,6-dione (3.4 g, 20.7 mmol, 1.0 eq), AcOH (12.5 mL, 218.0 mmol, 10.6 eq) and TEA (15.0 mL, 108.0 mmol, 5.2 eq) at 25 °C. The mixture was stirred at 90 °C for 12 h. Reaction was monitored by LC-MS and product was confirmed. The mixture was filtered and the filtrate cake was dried in vacuum to give 2-amino-5-bromo-N-(2,6-dioxopiperidin-3-yl)benzamide **S26** (5.0 g, crude) as a gray solid.

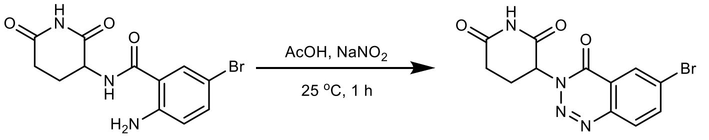

S26 **16**

To a solution of 2-amino-5-bromo-N-(2,6-dioxopiperidin-3-yl)benzamide **S26** (4.5 g, 13.8 mmol, 1.0 eq) in AcOH (50 mL) was added $NaNO_2$ (1.9 g, 27.6 mmol, 2.0 eq) at 25 °C. The mixture was stirred at 25 °C for 1 h. Reaction was monitored by LC-MS and product was confirmed. Ice water (20 mL) was added in the mixture, the mixture was filtered and the filtrate cake was dried in vacuum. The crude product was triturated with EtOAc (50 mL), MeOH (10 mL) and THF (30 mL) at 25 °C for 1 h to give 3-(6-bromo-4-oxobenzo[d][1,2,3]triazin-3(4H)-yl)piperidine-2,6-dione **16** (3.1 g, 65% yield) as a white solid. ^1H NMR (600 MHz, DMSO-d_6) δ 11.23 (s, 1H), 8.41 (d, J = 2.1 Hz, 1H), 8.33 (dd, J = 8.6, 2.2 Hz, 1H), 8.22 (d, J = 8.6 Hz, 1H), 6.01 (dd, J = 12.2, 5.3 Hz, 1H), 2.91-3.03 (m, 1H), 2.64-2.75 (m, 2H), 2.24-2.33 (m, 1H). ^{13}C NMR (150 MHz, DMSO-d_6) δ 173.1, 170.0, 153.9, 142.7, 139.4, 131.0, 127.6, 127.2, 121.2, 59.1, 31.2, 23.1.

2.2 High Throughput Miniaturized Reaction (Figure 3)

In this experiment, four conditions were selected to synthesize each product. The layout of reaction plates are the same in all cases, where each 384-well plate is split into four parts by columns, columns 1-6 are condition 1, columns 7-12 are condition 2, columns 13-18 are condition 3, and columns 19-24 are condition 4. To simplify the protocol, every 384-well plate contains only one glutarimide scaffold using one type of reaction. 16 glutarimide scaffolds were selected as starting materials with pre-established fragmentation patterns by MS/MS. Glutaramide building blocks (**1**-**16**) were the limiting reagents and were dissolved in DMF at 0.1 M. Building blocks which were HCl salts were stirred with 0.5 eq of Cs_2CO_3 in DMF for 4 h before load into the plates. Coupling partners in groups of 96 were pre-formatted into 384-well Greiner plate (Part 784201) as four copies

at 0.2 M in DMF. Automated liquid handling was performed using an apricot PP5 liquid handler using either 384-tip or 16-tip heads for whole plate and column-by-column transfers. All reactions were assembled into 384-well echo LDV plates to enable downstream acoustic liquid transfer events. These experiments were carried out in a N_2 filled glovebox including prepare solutions, liquid transfer, and overnight reaction.

2.1.1 Amidation Reaction

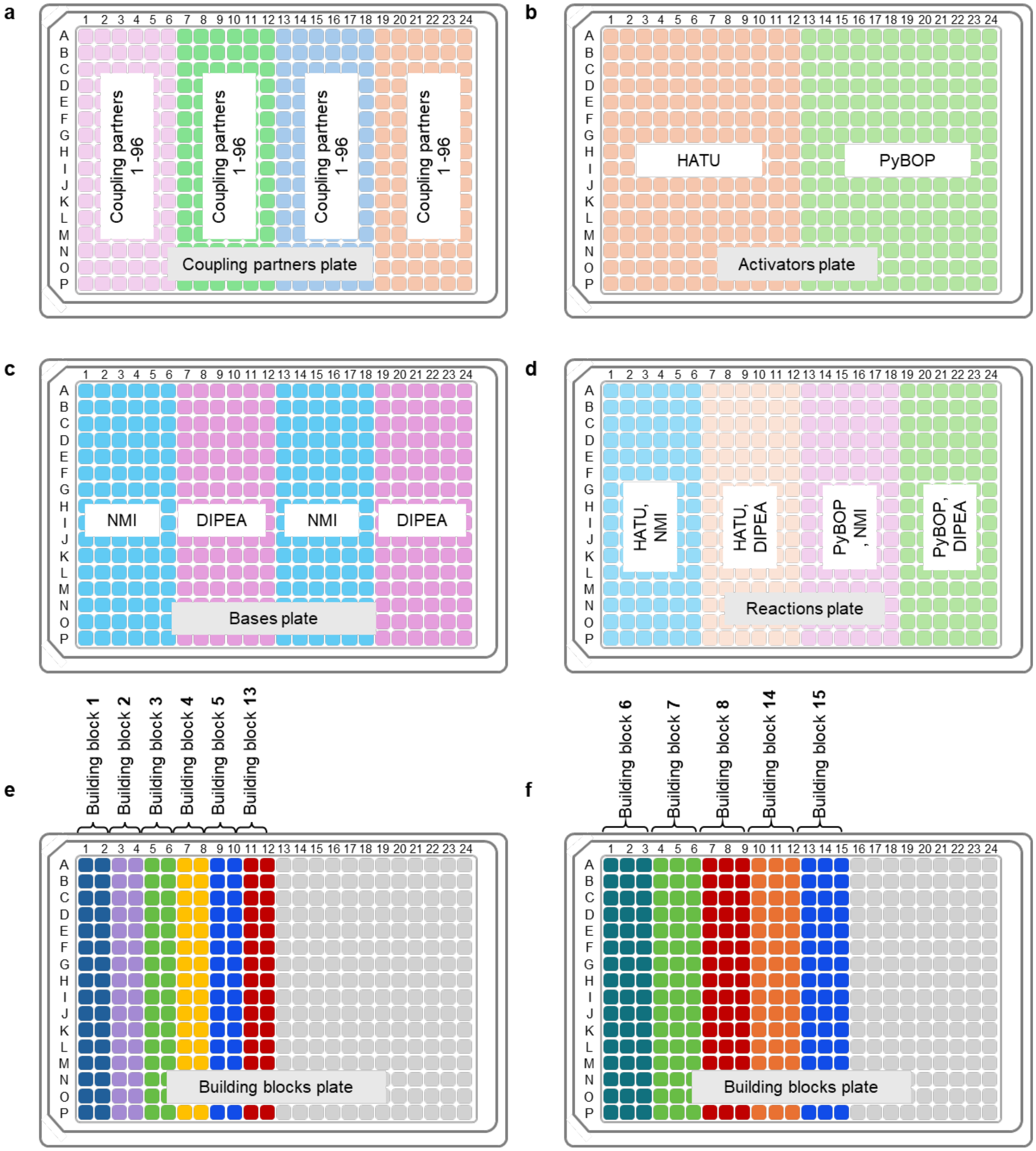

Supplementary Figure 10. Amidation Reaction Source Plates Layouts. Groups of 96 coupling partner were arranged as shown in (a), with four copies of each reagent. Two activators (hexafluorophosphate azabenzotriazole tetramethyl uranium (HATU) and benzotriazol-1-yloxytripyrrolidinophosphonium hexafluorophosphate (PyBOP), and two bases (N-methylimidazole (NMI) and N,N-diisopropylethylamine (DIPEA) were selected to give four reaction conditions combinations. The layout of activators and bases is shown in (b) and (c)**.** Building blocks only filled two or three columns per scaffold to save materials as shown in **e** and **f.** Once assembled the reagents will occupy distinct quarters of the plate as shown in (d). To make a

reaction plate, 1 µL solution was transferred from each source plate (a, b and c) followed by a single building block from plate (e) or (f) to make a final 4 µL reaction solution. Coupling partners, activators, and bases were transferred by whole plate transfer using a 384-tip head into 384-well echo LDV plates. Building blocks were transferred last 1 µL * 24 times column-by-column. One source column would have 60 µL solution per well to enable creation of two whole reaction plates. Where coupling partners were carboxylic acids they were incubated with activator and base for 2-4 hours at room temperature prior to addition of building blocks. Similarly, where building blocks were carboxylic acids they were incubated with activators and bases for 2-4 h at room temperature prior to addition of coupling partners.

2.1.2 Buchwald-Hartwig C-N coupling Reaction

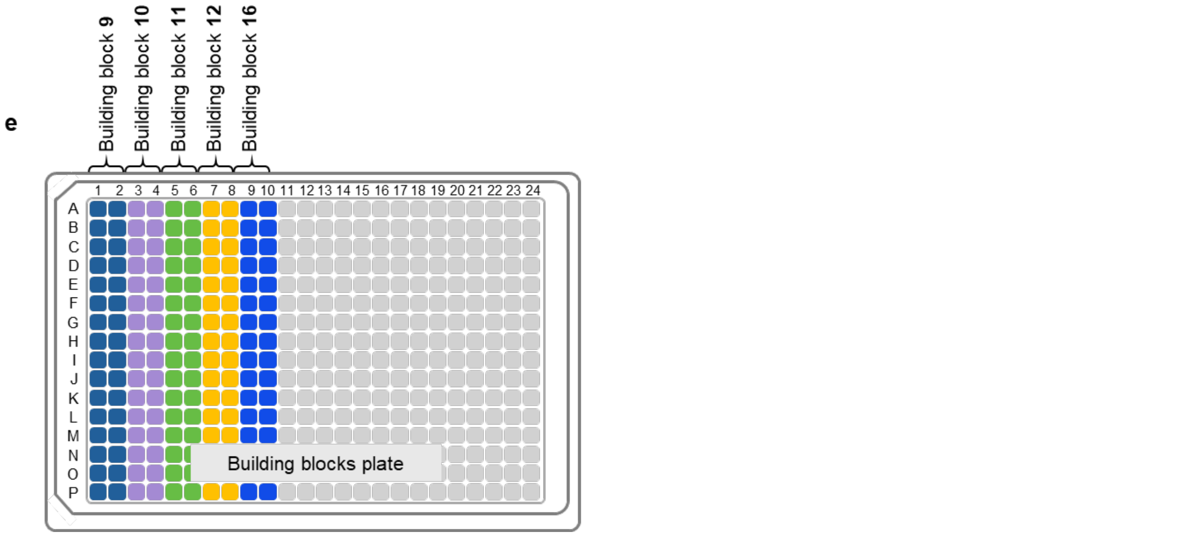

Supplementary Figure 11. Buchwald-Hartwig Reaction Source Plates Layouts**.** Groups of 96 coupling partners were arranged as shown in (a), coupling partners were arrayed as 4 copies as described above. Two palladium

catalysts (tBuXPhos Pd G4 and tBuBrettPhos Pd G4), and two bases (Barton's base (BTMG) and phosphazene base P2Et) were selected to give four reaction conditions. The layout of Pd catalysts and bases is shown in (b) and (c). The final reaction plate is shown in (d). Building blocks only filled two columns per scaffold as shown in **e.** To make the reaction plate, 1 µL solution was assembled from each plate (a,b,c,e) to make a final 4 µL reaction. Coupling partners, Pd catalysts, and bases were transferred by whole plate transfer using a 384-tip head. Then, building blocks were transferred 1 µL * 24 times using column-by-column transfers. One column has 60 µL solution per well which can make two whole reaction plates.

2.1.3 Reaction Analysis (Supplementary Figures 3, 4, and 5)

After overnight reaction, plates were removed from the glovebox and quenched through addition of DMSO/H_2O (*v:v* = 3:1, 4 µL) for amidation or acetic acid (AcOH) in DMSO (4 µL, 0.75 M) for Buchwald-Hartwig reaction using multidrop combi nL. After centrifuging the plates for 5 min at 1000 rpm, 100 nL of quenched reaction solution was Echo transferred to 384-well PP plate making a whole plate copy to create analysis plates. 50 µL of DMSO/H_2O (*v:v* = 7:3) solution was then added to every well of the analysis plates using a multidrop combi nL. Analysis plates were centrifuged for 5 min at 2000 rpm, and subject to orbital shaking for 5 mins at 1350 rpm (BioShake 5000 elm) right before NL-ADE -MS tests. Data collection was separated across two days, about half of the plates was stored at 4 °C and thawed to room temperature a few hours before test. Plates were centrifuged and orbitally shaken as described above. Each data was collected in duplicate and averaged. A total of about 18 hours analysis time was used to collect 69 plates. Reactions were selected based on the highest MS peak intensity on a per product basis. Selected best reactions were plotted as pie charts. Violin plots were generated to show the relative MS% distribution of each reaction after normalizing its MS peak intensity to the highest outcome. These results are shown in **Supplementary Figures 3**, **4**, and **5** separated by building block. Data processing was conducted using an in-house-built R script we previously described.

2.3 Scale-up Synthesis

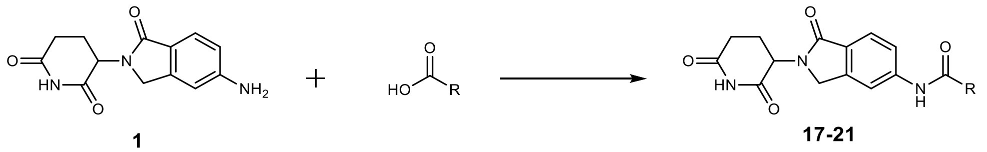

Hits (**17**-**21**) from ASMS screening was synthesized follow the selected conditions from high throughput synthesis. HATU was selected as activator and DIPEA as base in DMF at room temperature. Additional compounds used for defining the operational characteristics of glue identification by ASMS were also prepared according to this scheme.

N-(2-(2,6-dioxopiperidin-3-yl)-1-oxoisoindolin-5-yl)-3,5,7-trimethyl-1H-indole-2-carboxamide (**17**)

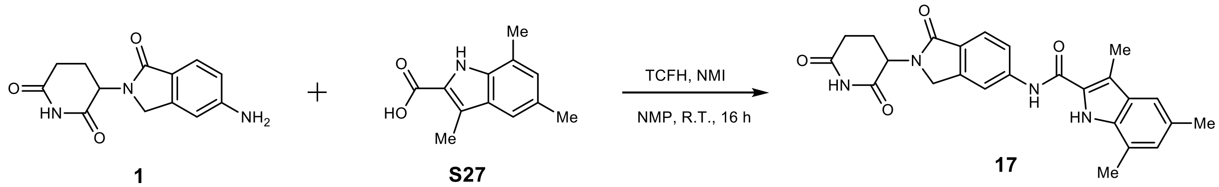

A mixture of C5 Lenalidomide **1** (19.0 mg, 0.074 mmol, 1.0 eq), 3,5,7-trimethyl-1H-indole-2-carboxylic acid **S27** (15.0 mg, 0.074 mmol, 1.0 eq), TCFH (0.023 g, 0.081 mmol, 1.1 eq) and NMI (0.018 mL, 0.22 mmol, 3.0 eq) was stirred at room temperature in NMP (0.5 mL) for 16 h. The reaction mixture was then diluted with ethyl acetate (3 mL) and washed with brine (3 mL × 3). The organic phase was dried over anhydrous Na_2SO_4, filtered and concentrated *in vacuo*. The crude was purified using automated flash chromatography (Biotage Sfar Amino

D 11g column, methanol/dichloromethane gradient mobile phase). Product-containing fractions were evaporated *in vacuo* to obtain a white solid (12.0 mg, 37% yield). ^{1}H NMR (500 MHz, DMSO-d_6) δ 11.05 (s, 1H), 10.99 (s, 1H), 10.21 (s, 1H), 8.14 (s, 1H), 7.62 - 7.85 (m, 2H), 7.24 (s, 1H), 6.91 (s, 1H), 5.11 (dd, *J* = 13.2, 4.9 Hz, 1H), 4.48 (d, *J* = 17.1 Hz, 1H), 4.34 (d, *J* = 17.2 Hz, 1H), 2.84 – 2.99 (m, 1H), 2.61 (d, *J* = 16.7 Hz, 1H), 2.53 (s, 3H), 2.50 (s, 3H, overlaps with residual DMSO peak), 2.37 (s, 4H), 1.93 - 2.08 (m, 1H). ^{13}C NMR (126 MHz, DMSO-d_6) δ 173.4, 171.6, 168.3, 161.3, 143.8, 142.8, 134.4, 128.7, 128.4, 127.5, 127.2, 127.0, 124.1, 121.5, 120.0, 117.3, 117.2, 114.5, 52.1, 47.7, 31.7, 23.0, 21.6, 17.3, 10.5.

3-Bromo-*N*-(2-(2,6-dioxopiperidin-3-yl)-1-oxoisoindolin-5-yl)-1*H*-indole-2-carboxamide (**18**)

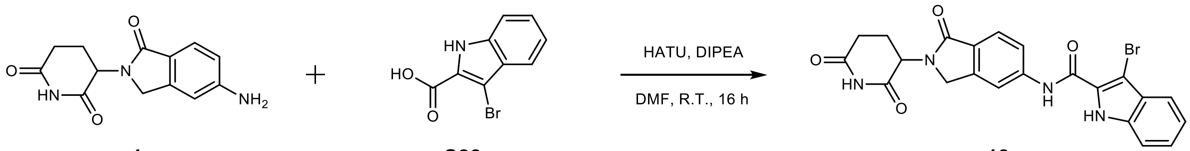

C5 Lenalidomide **1** (30.0 mg, 0.116 mmol, 1.0 eq) was added to a stirring solution of 3-bromo-1*H*-indole-2-carboxylic acid **S28** (27.8 mg, 0.116 mmol, 1.0 eq), HATU (66.0 mg, 0.174 mmol, 1.5 eq), and DIPEA (40.4 µL, 0.231 mmol, 2.0 eq) in DMF (0.5 mL). The reaction mixture was stirred for 16 h and then purified using automated reversed-phase flash chromatography (Biotage Sfar C18 30g column, acetonitrile/water gradient mobile phase with 0.1% formic acid additive). Product-containing fractions were lyophilized to obtain a white solid (3.0 mg, 5% yield). ^{1}H NMR (500 MHz, DMSO-d_6) δ 12.27 (s, 1H), 11.00 (s, 1H), 10.47 (s, 1H), 8.11 (d, *J* = 1.7 Hz, 1H), 7.79 (dd, *J* = 8.2, 1.8 Hz, 1H), 7.75 (d, *J* = 8.3 Hz, 1H), 7.56 (d, *J* = 8.1 Hz, 1H), 7.52 (d, *J* = 8.3 Hz, 1H), 7.35 (ddd, *J* = 8.2, 7.0, 1.2 Hz, 1H), 7.22 (t, *J* = 7.5 Hz, 1H), 5.11 (dd, *J* = 13.3, 5.1 Hz, 1H), 4.49 (d, *J* = 17.3 Hz, 1H), 4.35 (d, *J* = 17.2 Hz, 1H), 2.98 – 2.84 (m, 1H), 2.65 – 2.58 (m, 1H), 2.40 (qd, *J* = 13.2, 4.3 Hz, 1H), 2.02 (ddd, *J* = 13.4, 5.9, 3.7 Hz, 1H). ^{13}C NMR (126 MHz, DMSO-d_6) δ 173.0, 171.2, 167.9, 159.1, 143.4, 141.8, 135.3, 128.8, 127.1, 126.7, 125.3, 123.9, 121.2, 119.7, 119.7, 114.1, 112.9, 51.7, 47.3, 31.2, 22.6.

3-Chloro-*N*-(2-(2,6-dioxopiperidin-3-yl)-1-oxoisoindolin-5-yl)-5-fluoro-1*H*-indole-2-carboxamide (**19**)

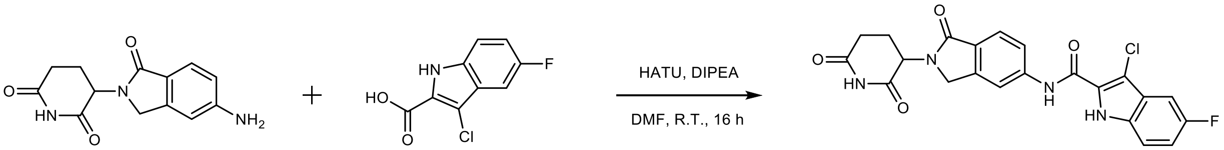

C5 Lenalidomide **1** (30.0 mg, 0.116 mmol, 1.0 eq) was added to a stirring solution of 3-chloro-5-fluoro-1*H*-indole-2-carboxylic acid **S29** (24.7 mg, 0.116 mmol, 1.0 eq), HATU (66.0 mg, 0.174 mmol, 1.5 eq), and DIPEA (40.4 µL, 0.231 mmol, 2.0 eq) in DMF (0.5 mL). The reaction mixture was stirred for 16 h and then purified using automated reversed-phase flash chromatography (Biotage Sfar C18 30g column, acetonitrile/water gradient mobile phase with 0.1% formic acid additive). Product-containing fractions were lyophilized to obtain a white solid (6.0 mg, 11% yield). ^{1}H NMR (500 MHz, DMSO-d_6) δ 12.31 (s, 1H), 11.00 (s, 1H), 10.46 (s, 1H), 8.09 (d, *J* = 1.7 Hz, 1H), 7.79 (dd, *J* = 8.2, 1.8 Hz, 1H), 7.75 (d, *J* = 8.3 Hz, 1H), 7.54 (dd, *J* = 9.0, 4.3 Hz, 1H), 7.38 (dd, *J* = 9.1, 2.5 Hz, 1H), 7.23 (td, *J* = 9.2, 2.6 Hz, 1H), 5.11 (dd, *J* = 13.3, 5.1 Hz, 1H), 4.49 (d, *J* = 17.3 Hz, 1H), 4.35 (d, *J* = 17.3 Hz, 1H), 2.92 (ddd, *J* = 17.3, 13.6, 5.4 Hz, 1H), 2.61 (dt, *J* = 17.2, 3.7 Hz, 1H), 2.40 (qd, *J* = 13.2, 4.4 Hz, 1H), 2.02 (dtd, *J* = 11.7, 6.0, 3.3 Hz, 1H). ^{13}C NMR (126 MHz, DMSO-d_6) δ 173.0, 171.2, 167.8, 158.2, 158.6, 156.9, 142.5 (d, J = 217.5 Hz), 131.3, 128.8, 127.2, 125.1 (d, J = 10.6 Hz), 123.9, 119.8,

114.7 (d, J = 9.6 Hz), 114.3 (d, J = 26.9 Hz), 114.3, 105.8 (d, J = 5.3 Hz), 103.2 (d, J = 24.6 Hz), 51.7, 47.3, 31.3, 22.6.

N-(2-(2,6-dioxopiperidin-3-yl)-1-oxoisoindolin-5-yl)-3-ethyl-1H-indole-2-carboxamide (**20**)

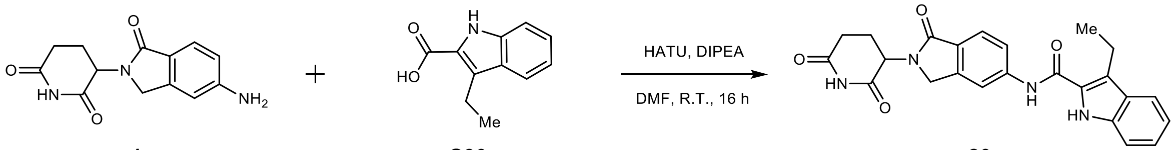

1 **S30** **20**

To a solution of 3-ethyl-1H-indole-2-carboxylic acid **S30** (22.0 mg, 0.120 mmol, 1.0 eq) in DMF (0.5 mL) at room temperature was added DIPEA (0.060 mL, 0.35 mmol, 3.0 eq), HATU (48.0 mg, 0.13 mmol, 1.1 eq) and C5 Lenalidomide**1** (30.0 mg, 0.12 mmol, 1.0 eq). The reaction mixture was stirred at room temperature for 16 h then was diluted with ethyl acetate (3 mL) and washed sequentially with brine (3 mL × 3) followed by 5% aqueous LiCl solution (3 mL). The organic phase was dried over anhydrous Na_2SO_4, filtered and concentrated *in vacuo*. The crude was purified using automated flash chromatography (Biotage Sfar Amino D 11g column, methanol/dicholoromethane gradient mobile phase). Product-containing fractions were evaporated *in vacuo* to obtain a tan solid (8.3 mg, 17% yield). ^{1}H NMR (500 MHz, DMSO-d_6) δ 11.44 (s, 1H),10.99 (s, 1H), 10.29 (s, 1H), 8.15 (s, 1H), 7.70 - 7.79 (m, 2H), 7.68 (d, J = 8.1 Hz, 1H), 7.46 (d, J = 8.2 Hz, 1H), 7.23 - 7.31 (m, 1H), 7.03 - 7.13 (m, 1H), 5.11 (dd, J = 13.3, 5.1 Hz, 1H), 4.26 - 4.54 (m, 2H), 3.09 (q, J = 7.4 Hz, 2H), 2.86 - 2.97 (m, 1H), 2.57 - 2.66 (m, 1H), 2.34 - 2.46 (m, 1H), 1.97 - 2.06 (m, 1H), 1.22 (t, J = 7.5 Hz, 3H). ^{13}C NMR (126 MHz, DMSO-d_6) δ 173.4, 171.6, 168.3, 161.2, 143.8, 142.8, 136.1, 127.5, 127.0, 124.8, 124.1, 123.8, 120.4, 119.9, 114.4, 112.7, 52.0, 47.7, 31.7, 23.0, 17.9, 16.2.

N-(2-(2,6-Dioxopiperidin-3-yl)-1-oxoisoindolin-5-yl)-3-methyl-1*H*-indole-2-carboxamide (**21**)

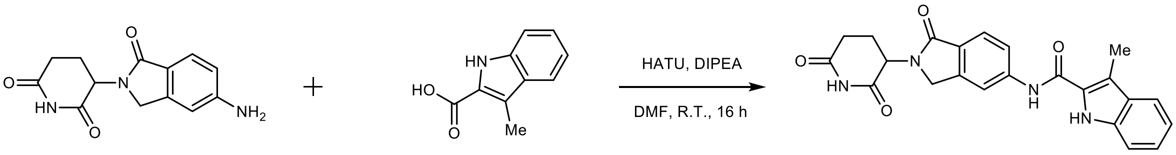

1 **S31** **21**

C5 Lenalidomide **1** (30.0 mg, 0.116 mmol, 1.0 eq) was added to a stirring solution of 3-methyl-1*H*-indole-2-carboxylic acid **S31** (20.3 mg, 0.116 mmol, 1.0 eq), HATU (66.0 mg, 0.174 mmol, 1.5 eq), and DIPEA (40.4 µL, 0.231 mmol, 2.0 eq) in DMF (0.5 mL). The reaction mixture was stirred for 16 h and then diluted with water (5 mL) and extracted with EtOAc (5 mL × 3). The combined organic layers were washed with brine, dried with anhydrous Na_2SO_4, filtered, and concentrated. The crude product was purified using automated flash chromatography (Sfar Silica HC D 10 g column, methanol/dichloromethane gradient mobile phase). Product-containing fractions were concentrated to obtain a white solid (11.0 mg, 22% yield). ^{1}H NMR (500 MHz, DMSO-d_6) δ 11.45 (s, 1H), 11.00 (s, 1H), 10.24 (s, 1H), 8.13 (d, J = 1.8 Hz, 1H), 7.78 (dd, J = 8.3, 1.7 Hz, 1H), 7.73 (d, J = 8.2 Hz, 1H), 7.66 (d, J = 8.0 Hz, 1H), 7.45 (d, J = 8.2 Hz, 1H), 7.26 (t, J = 7.6 Hz, 1H), 7.09 (t, J = 7.5 Hz, 1H), 5.10 (dd, J = 13.3, 5.1 Hz, 1H), 4.48 (d, J = 17.2 Hz, 1H), 4.34 (d, J = 17.2 Hz, 1H), 2.92 (ddd, J = 18.0, 13.6, 5.4 Hz, 1H), 2.67 – 2.58 (m, 1H), 2.56 (s, 3H), 2.40 (qd, J = 13.2, 4.5 Hz, 1H), 2.04 – 1.94 (m, 1H). ^{13}C NMR (126 MHz, DMSO-d_6) δ 173.0, 171.2, 168.0, 161.0, 143.3, 142.4, 135.7, 128.0, 127.4, 126.6, 124.4, 123.8, 120.0, 119.6, 119.5, 116.0, 114.0, 112.1, 51.7, 47.3, 31.3, 22.6, 9.9.

3,5,7-trimethyl-N-(2-(1-methyl-2,6-dioxopiperidin-3-yl)-1-oxoisoindolin-5-yl)-1H-indole-2-carboxamide (**17-Me**)

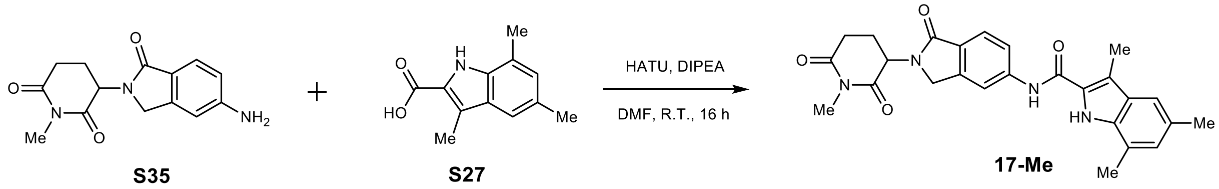

A solution of 3-(5-amino-1-oxoisoindolin-2-yl)-1-methylpiperidine-2,6-dione **S35** (15.0 mg, 0.055 mmol, 1.0 eq), 3,5,7-trimethyl-1H-indole-2-carboxylic acid **S27** (13.4 mg, 0.066 mmol, 1.2 eq), HATU (31.3 mg, 0.082 mmol, 1.5 eq), and DIPEA (19 µL, 0.109 mmol, 2.0 eq) was stirred for 24 h in DMF (600 µL). The reaction mixture was purified using automated reversed-phase flash chromatography (Biotage Sfar C18 30 g column, acetonitrile/water gradient mobile phase with 0.1% formic acid additive). Product-containing fractions were lyophilized to obtain a white solid (3.8 mg, 15% yield). ^{1}H NMR (500 MHz, DMSO) δ 11.10 (s, 1H), 10.27 (s, 1H), 8.14 (d, *J* = 1.8 Hz, 1H), 7.79 (dd, *J* = 1.8, 8.2 Hz, 1H), 7.74 (d, *J* = 8.2 Hz, 1H), 7.24 (s, 1H), 6.91 (s, 1H), 5.18 (dd, *J* = 5.1, 13.5 Hz, 1H), 4.48 (d, *J* = 17.1 Hz, 1H), 4.34 (d, *J* = 17.1 Hz, 1H), 3.01 (s, 3H), 3.05 – 2.96 (m, 1H), 2.77 (ddd, *J* = 2.4, 4.5, 17.3 Hz, 1H), 2.53 (s, 3H), 2.44 – 2.38 (m, 1H), 2.37 (s, 3H), 2.07 – 1.99 (m, 1H). ^{13}C NMR (126 MHz, DMSO) δ 172.0, 170.8, 167.9, 160.8, 143.3, 142.4, 133.9, 128.2, 127.9, 127.0, 126.8, 126.5, 123.7, 121.0, 119.6, 116.8, 116.7, 114.0, 52.1, 47.2, 31.4, 30.7, 26.6, 21.8, 21.2, 16.8, 10.0.

3-bromo-N-(2-(1-methyl-2,6-dioxopiperidin-3-yl)-1-oxoisoindolin-5-yl)-1H-indole-2-carboxamide (**18-Me**)

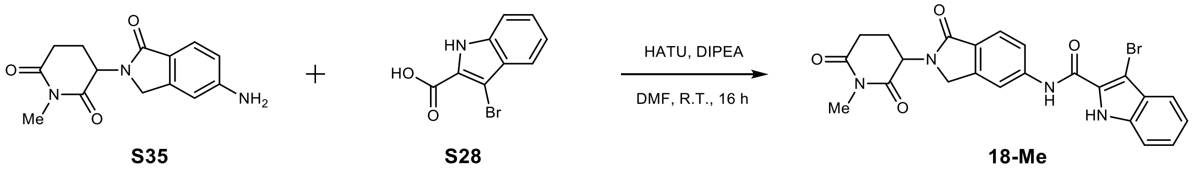

A solution of 3-(5-amino-1-oxoisoindolin-2-yl)-1-methylpiperidine-2,6-dione **S35** (15.0 mg, 0.055 mmol, 1.0 eq), 3-bromo-1*H*-indole-2-carboxylic acid **S28** (15.8 mg, 0.066 mmol, 1.2 eq), HATU (31.1 mg, 0.082 mmol, 1.5 eq), and DIPEA (19 µL, 0.109 mmol, 2.0 eq) was stirred for 24 h in DMF (600 µL). The reaction mixture was purified using automated reversed-phase flash chromatography (Biotage Sfar C18 30 g column, acetonitrile/water gradient mobile phase with 0.1% formic acid additive). Product-containing fractions were lyophilized to obtain a white solid (7.2 mg, 27% yield). ^{1}H NMR (500 MHz, DMSO) δ 12.27 (s, 1H), 10.48 (s, 1H), 8.12 (d, *J* = 1.8 Hz, 1H), 7.82 – 7.73 (m, 2H), 7.54 (dd, *J* = 8.2, 19.1 Hz, 2H), 7.35 (ddd, *J* = 1.1, 7.0, 8.2 Hz, 1H), 7.23 (ddd, *J* = 0.9, 7.0, 7.9 Hz, 1H), 5.18 (dd, *J* = 5.1, 13.4 Hz, 1H), 4.49 (d, *J* = 17.2 Hz, 1H), 4.35 (d, *J* = 17.2 Hz, 1H), 3.01 (s, 3H), 3.05 – 2.94 (m, 1H), 2.77 (ddd, *J* = 2.4, 4.5, 17.2 Hz, 1H), 2.54 (s, 1H), 2.42 (td, *J* = 4.6, 13.1 Hz, 1H). ^{13}C NMR (126 MHz, DMSO) δ 172.0, 170.8, 167.8, 159.1, 143.4, 141.8, 135.3, 128.8, 127.0, 126.6, 125.2, 123.8, 121.1, 119.6, 119.6, 114.1, 112.8, 52.1, 47.2, 40.4, 31.4, 26.6, 21.8.

3-chloro-5-fluoro-N-(2-(1-methyl-2,6-dioxopiperidin-3-yl)-1-oxoisoindolin-5-yl)-1H-indole-2-carboxamide (**19-Me**)

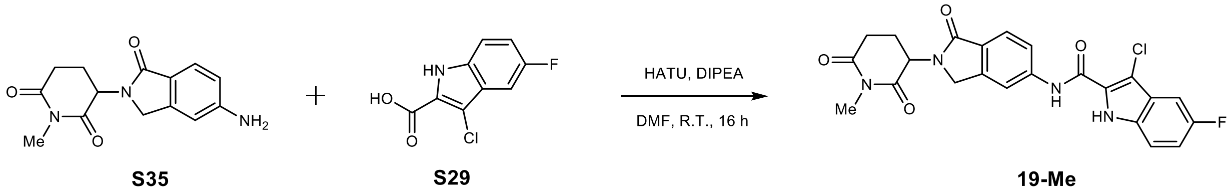

A solution of 3-(5-amino-1-oxoisoindolin-2-yl)-1-methylpiperidine-2,6-dione **S35** (15.0 mg, 0.055 mmol, 1.0 eq), 3-chloro-5-fluoro-1*H*-indole-2-carboxylic acid **S29** (14.1 mg, 0.066 mmol, 1.2 eq), HATU (31.1 mg, 0.082 mmol, 1.5 eq), and DIPEA (19 µL, 0.109 mmol, 2.0 eq) was stirred for 24 h in DMF (600 µL). The reaction mixture was purified using automated reversed-phase flash chromatography (Biotage Sfar C18 30 g column, acetonitrile/water gradient mobile phase with 0.1% formic acid additive). Product-containing fractions were lyophilized to obtain a white solid (4.6 mg, 18% yield). ^{1}H NMR (500 MHz, DMSO) δ 12.30 (s, 1H), 10.47 (s, 1H), 8.10 (d, *J* = 1.7 Hz, 1H), 7.82 – 7.73 (m, 2H), 7.54 (dd, *J* = 4.4, 9.0 Hz, 1H), 7.38 (dd, *J* = 2.5, 9.0 Hz, 1H), 7.23 (td, *J* = 2.5, 9.2 Hz, 1H), 5.18 (dd, *J* = 5.0, 13.4 Hz, 1H), 4.49 (d, *J* = 17.2 Hz, 1H), 4.34 (d, *J* = 17.2 Hz, 1H), 301 (s, 3H), 3.03 – 2.96 (m, 1H), 2.77 (d, *J* = 15.4 Hz, 1H), 2.44 – 2.37 (m, 1H), 2.03 (dt, *J* = 6.3, 12.6 Hz, 1H). ^{13}C NMR (126 MHz, DMSO) δ 171.9, 170.8, 167.8, 158.5, 156.8, 142.5 (d, *J* = 214.3 Hz), 131.3, 128.8, 127.1, 125.0 (d, *J* = 10.7 Hz), 123.8, 119.8, 114.7 (d, *J* = 9.5 Hz), 114.2, 114.2 (d, *J* = 26.4 Hz), 105.6 (d, *J* = 5.1 Hz), 103.1 (d, *J* = 24.6 Hz), 52.1, 47.2, 31.4, 26.6, 21.8.

7-bromo-3-ethyl-N-(2-(1-methyl-2,6-dioxopiperidin-3-yl)-1-oxoisoindolin-5-yl)-1H-indole-2-carboxamide (**22**).

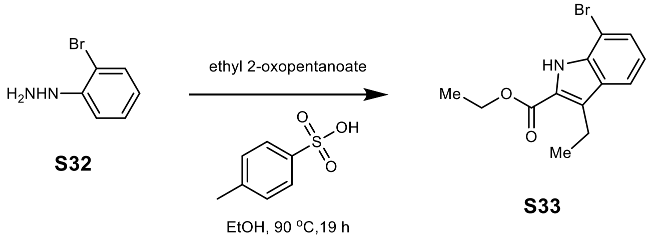

To a solution of (2-bromophenyl)hydrazine hydrochloride **S32** (244 mg, 1.09 mmol, 1.0 eq), and ethyl 2-oxopentanoate (157 mg, 1.09 mmol, 1.0 eq), in EtOH (10 mL), was added *p*-toluenesulfonic acid monohydrate (623 mg, 3.28 mmol, 3.0 eq) and stirred at 90 °C. After 3 h, additional *p*-toluenesulfonic acid monohydrate (623 mg, 3.28 mmol, 3.0 eq) was added and the reaction mixture stirred for an additional 16 h. The solution was cooled to rt, diluted with saturated $NaHCO_3$ (50 mL), and washed with EtOAc (50 mL). The organic layer was washed with brine, dried with anhydrous Na_2SO_4, filtered, and concentrated. The crude mixture was purified by flash column chromatography (Biotage Isolera, 25 g Sfar column, 0–50% hexanes:EtOAc) to give the desired product **S33** (101 mg, 31% yield). ^{1}H NMR (500 MHz, $CDCl_3$) δ 8.74 (s, 1H), 7.64 (d, *J* = 8.1 Hz, 1H), 7.47 (d, *J* = 7.5 Hz, 1H), 7.02 (t, *J* = 7.8 Hz, 1H), 4.45 (q, *J* = 7.1 Hz, 2H), 3.10 (q, *J* = 7.5 Hz, 2H), 1.44 (t, *J* = 7.2 Hz, 3H), 1.27 (t, *J* = 7.5 Hz, 3H).

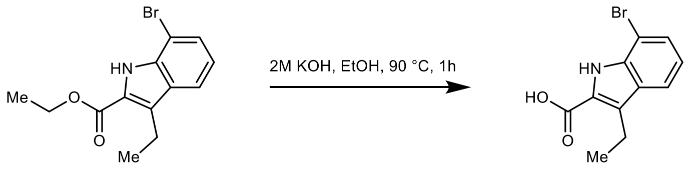

S33 **S34**

A solution of ethyl 7-bromo-3-ethyl-1*H*-indole-2-carboxylate **S33** (60 mg, 0.20 mmol, 1.0 eq) in 2M KOH (0.8 mL, 8.0 eq) and EtOH (2 mL) was stirred at 90 °C. After 1 h, the reaction mixture was cooled to room temperature and then directly purified using automated reversed-phase flash chromatography (Biotage Sfar C18 30 g column, acetonitrile/water gradient mobile phase with 0.1% formic acid additive). Product-containing fractions were lyophilized to obtain a white solid **S34** (50 mg, 92%). ^{1}H NMR (400 MHz, DMSO-*d*$_6$) δ 13.18 (s,

1H), 11.03 (s, 1H), 7.69 (d, *J* = 8.0 Hz, 1H), 7.48 (d, *J* = 7.5 Hz, 1H), 7.01 (t, *J* = 7.7 Hz, 1H), 3.05 (q, *J* = 7.4 Hz, 2H), 1.18 (t, *J* = 7.4 Hz, 3H).

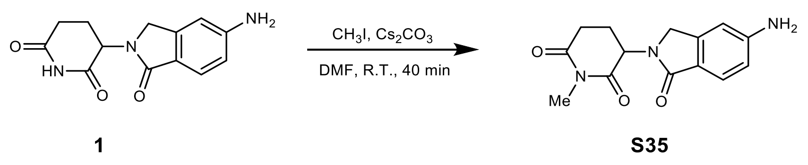

To a solution of 3-(5-amino-1-oxoisoindolin-2-yl)piperidine-2,6-dione **1** (100 mg, 0.39 mmol, 1.0 eq) and Cs_2CO_3 (151 mg, 0.46 mmol, 1.2 eq) in anhydrous DMF (2 mL) was added methyl iodide (0.036 mL, 0.58 mmol, 1.5 eq). The reaction mixture was stirred for 40 min at room temperature and then diluted with water (15 mL) and extracted with EtOAc (15 mL × 3). The combined organic layers were washed with brine, dried over anhydrous Na_2SO_4, filtered, and concentrated. The crude product was purified using automated flash chromatography (Sfar Silica HC D 25 g column, methanol/dichloromethane gradient mobile phase). Product-containing fractions were concentrated to obtain the desired product **S35** (66 mg, 63% yield). ^{1}H NMR (400 MHz, DMSO-d_6) δ 7.35 (d, *J* = 8.7 Hz, 1H), 6.66 – 6.57 (m, 2H), 5.80 (s, 2H), 5.07 (dd, *J* = 13.4, 5.0 Hz, 1H), 4.24 (d, *J* = 16.5 Hz, 1H), 4.10 (d, *J* = 16.6 Hz, 1H), 3.04 – 2.92 (m, 4H), 2.73 (s, 1H), 2.33 (qd, *J* = 13.2, 4.6 Hz, 1H), 1.99 – 1.91 (m, 1H).

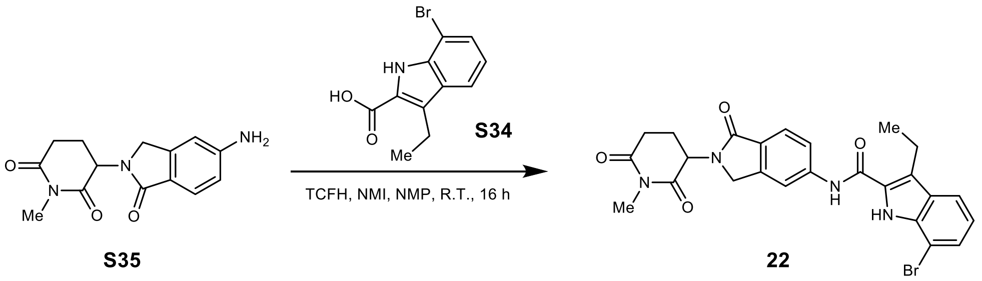

A solution of 3-(5-amino-1-oxoisoindolin-2-yl)-1-methylpiperidine-2,6-dione **S35** (20 mg, 0.073 mmol, 1.0 eq), 7-bromo-3-ethyl-1*H*-indole-2-carboxylic acid **S34** (19.6 mg, 0.073 mmol, 1.0 eq), TCFH (41.1 mg, 0.146 mmol, 2.0 eq), and *N*-methylimidazole (17.5 µL, 0.220 mmol, 3.0 eq) was stirred for 2.5 h in NMP (500 µL). The reaction mixture was purified using automated reversed-phase flash chromatography (Biotage Sfar C18 30 g column, acetonitrile/water gradient mobile phase with 0.1% formic acid additive). Product-containing fractions were lyophilized to obtain a white solid (12 mg, 31% yield). ^{1}H NMR (500 MHz, DMSO-d_6) δ 11.52 (s, 1H), 10.61 (s, 1H), 8.18 (s, 1H), 7.81 (dd, *J* = 8.2, 1.8 Hz, 1H), 7.77 – 7.71 (m, 2H), 7.52 (dd, *J* = 7.5, 0.9 Hz, 1H), 7.05 (t, *J* = 7.7 Hz, 1H), 5.18 (dd, *J* = 13.4, 5.1 Hz, 1H), 4.49 (d, *J* = 17.2 Hz, 1H), 4.34 (d, *J* = 17.2 Hz, 1H), 3.11 (q, *J* = 7.5 Hz, 2H), 3.02 (s, 4H), 2.77 (ddd, *J* = 17.2, 4.5, 2.4 Hz, 1H), 2.41 (qd, *J* = 13.2, 4.5 Hz, 1H), 2.03 (dtd, *J* = 12.5, 5.2, 2.3 Hz, 1H), 1.21 (t, *J* = 7.4 Hz, 3H). ^{13}C NMR (126 MHz, DMSO-d_6) δ 171.9, 170.8, 167.9, 159.8, 143.3, 142.2, 134.2, 128.6, 127.3, 127.0, 126.7, 125.6, 123.7, 120.9, 119.8, 119.4, 114.2, 104.6, 52.1, 47.2, 31.4, 26.6, 21.8, 17.7, 15.5.

4-Acetyl-*N*-(2-(2,6-dioxopiperidin-3-yl)-1-oxoisoindolin-5-yl)-3-ethyl-5-methyl-1*H*-pyrrole-2-carboxamide (**23**)

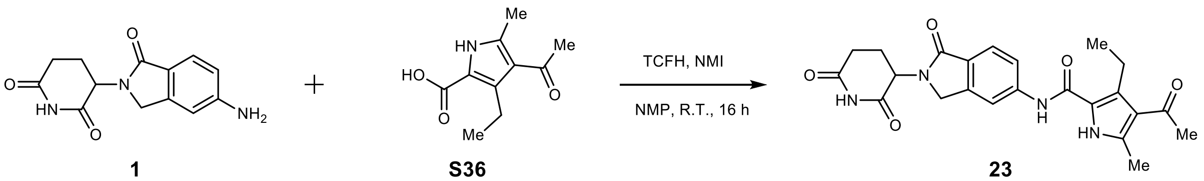

1 **S36** **23**

A solution of C5 Lenalidomide **1** (10.0 mg, 0.039 mmol, 1.0 eq), 4-acetyl-3-ethyl-5-methyl-1*H*-pyrrole-2-carboxylic acid **S36** (7.5 mg, 0.039 mmol, 1.0 eq), TCFH (11.9 mg, 0.042 mmol, 1.1 eq), and NMI (9.2 µL, 0.116 mmol, 3.0 eq) was stirred in NMP (0.5 mL) for 16 h. The reaction mixture was purified using automated reversed-phase flash chromatography (Biotage Sfar C18 30g column, acetonitrile/water gradient mobile phase with 0.1% formic acid additive). Product-containing fractions were lyophilized to obtain the desired product. ^{1}H NMR (500 MHz, DMSO-d_6) δ 11.69 (s, 1H), 10.98 (s, 1H), 9.92 (s, 1H), 8.06 (d, J = 1.7 Hz, 1H), 7.69 (d, J = 8.2 Hz, 1H), 7.65 (dd, J = 8.3, 1.8 Hz, 1H), 5.09 (dd, J = 13.3, 5.1 Hz, 1H), 4.45 (d, J = 17.2 Hz, 1H), 4.31 (d, J = 17.2 Hz, 1H), 3.02 (q, J = 7.3 Hz, 2H), 2.92 (ddd, J = 17.3, 13.7, 5.4 Hz, 1H), 2.65 – 2.56 (m, 1H), 2.51 (s, 3H), 2.43 – 2.34 (m, 4H), 2.01 (dtd, J = 11.0, 5.5, 3.0 Hz, 1H), 1.07 (t, J = 7.3 Hz, 3H). ^{13}C NMR (126 MHz, DMSO-d_6) δ 194.2, 172.9, 171.1, 167.9, 159.6, 143.3, 142.5, 136.7, 133.9, 126.2, 123.6, 121.5, 120.6, 119.2, 113.6, 51.5, 47.2, 31.2, 31.0, 22.5, 18.3, 15.9, 14.9.

N-(2-(2,6-Dioxopiperidin-3-yl)-1-oxoisoindolin-5-yl)-3-iodo-1*H*-indole-2-carboxamide (**24**)

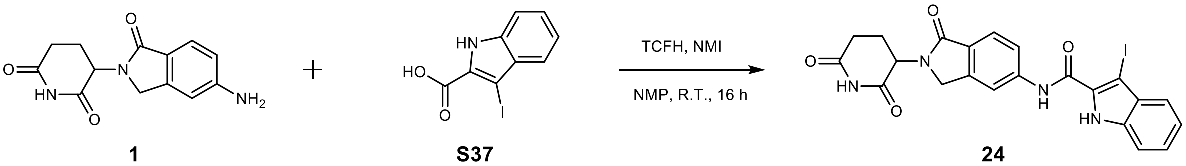

1 **S37** **24**

A solution of C5 Lenalidomide**1** (10.0 mg, 0.039 mmol, 1.0 eq), 3-iodo-1*H*-indole-2-carboxylic acid **S37** (11.1 mg, 0.039 mmol, 1.0 eq), TCFH (11.9 mg, 0.042 mmol, 1.1 eq), and NMI (9.2 µL, 0.116 mmol, 3.0 eq) was stirred in NMP (0.5 mL) for 16 h. The reaction mixture was purified using automated reversed-phase flash chromatography (Biotage Sfar C18 30g column, acetonitrile/water gradient mobile phase with 0.1% formic acid additive). Product-containing fractions were lyophilized to obtain the desired product. ^{1}H NMR (500 MHz, DMSO-d_6) δ 12.28 (s, 1H), 11.00 (s, 1H), 10.49 (s, 1H), 8.13 (d, J = 1.6 Hz, 1H), 7.79 – 7.72 (m, 2H), 7.49 (d, J = 8.1 Hz, 1H), 7.46 – 7.42 (m, 1H), 7.34 (ddd, J = 8.2, 6.9, 1.2 Hz, 1H), 7.21 (ddd, J = 8.1, 6.9, 1.0 Hz, 1H), 5.11 (dd, J = 13.3, 5.1 Hz, 1H), 4.50 (d, J = 17.3 Hz, 1H), 4.36 (d, J = 17.3 Hz, 1H), 2.93 (ddd, J = 17.2, 13.6, 5.3 Hz, 1H), 2.65 – 2.58 (m, 1H), 2.40 (qd, J = 13.3, 4.4 Hz, 1H), 2.02 (dtd, J = 11.2, 5.8, 3.2 Hz, 1H). ^{13}C NMR (126 MHz, DMSO-d_6) δ 172.9, 171.1, 167.8, 159.8, 143.4, 141.9, 136.3, 132.1, 130.2, 127.0, 125.1, 123.8, 121.8, 121.1, 119.5, 113.9, 112.7, 62.5, 51.6, 47.2, 31.2, 22.5.

4-Bromo-*N*-(2-(2,6-dioxopiperidin-3-yl)-1-oxoisoindolin-5-yl)-3-ethoxy-1*H*-pyrazole-5-carboxamide (**25**)

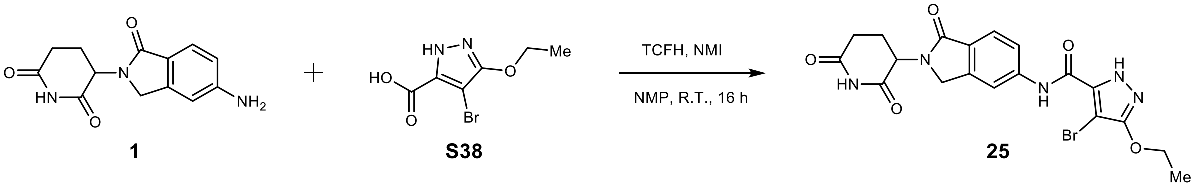

1 **S38** **25**

A solution of C5 Lenalidomide**1** (10.0 mg, 0.039 mmol, 1.0 eq), 4-bromo-3-ethoxy-1*H*-pyrazole-5-carboxylic acid **S38** (9.1 mg, 0.039 mmol, 1.0 eq), TCFH (11.9 mg, 0.042 mmol, 1.1 eq), and NMI (9.2 µL, 0.116 mmol, 3.0 eq) was stirred in NMP (0.5 mL) for 16 h. The reaction mixture was purified using automated reversed-phase flash chromatography (Biotage Sfar C18 30g column, acetonitrile/water gradient mobile phase with 0.1% formic acid additive). Product-containing fractions were lyophilized to obtain the desired product. ^{1}H NMR (500 MHz, DMSO-d_6) δ 10.99 (s, 1H), 10.51 (s, 1H), 8.06 (s, 1H), 7.73 (s, 2H), 5.10 (dd, *J* = 13.3, 5.1 Hz, 1H), 4.47 (d, *J* = 17.3 Hz, 1H), 4.33 (d, *J* = 17.3 Hz, 1H), 4.27 (q, *J* = 7.0 Hz, 2H), 2.92 (ddd, *J* = 17.3, 13.6, 5.4 Hz, 1H), 2.64 – 2.56 (m, 1H), 2.39 (qd, *J* = 13.2, 4.4 Hz, 1H), 2.01 (dtd, *J* = 12.7, 5.3, 2.3 Hz, 1H), 1.34 (t, *J* = 7.0 Hz, 3H). ^{13}C NMR (126 MHz, DMSO-d_6) δ 172.9, 171.09, 167.7, 143.3, 141.4, 127.2, 123.8, 119.6, 114.1, 51.6, 47.2, 31.2, 22.5, 14.7.

4-Bromo-*N*-(2-(2,6-dioxopiperidin-3-yl)-1-oxoisoindolin-5-yl)-3,5-dimethyl-1*H*-pyrrole-2-carboxamide (**26**)

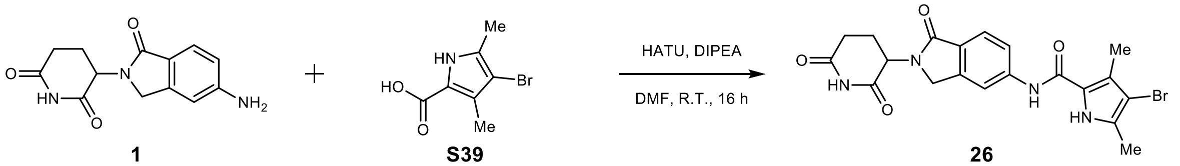

C5 Lenalidomide**1** (29.7 mg, 0.115 mmol, 1.0 eq) was added to a stirring solution of 4-bromo-3,5-dimethyl-1H-pyrrole-2-carboxylic acid **S39** (25.1 mg, 0.115 mmol, 1.0 eq), HATU (65.4 mg, 0.172 mmol, 1.5 eq), and DIPEA (60.1 µL, 0.344 mmol, 3.0 eq) in DMF (0.5 mL). The reaction mixture was stirred for 16 h and then purified using automated reversed-phase flash chromatography (Biotage Sfar C18 30g column, acetonitrile/water gradient mobile phase with 0.1% formic acid additive). Product-containing fractions were lyophilized to obtain a white solid (8.0 mg, 15% yield). ^{1}H NMR (500 MHz, DMSO-d_6) δ 11.82 (s, 1H), 10.98 (s, 1H), 9.84 (s, 1H), 8.03 (s, 1H), 7.68 (s, 2H), 5.09 (dd, *J* = 13.3, 5.1 Hz, 1H), 4.44 (d, *J* = 17.2 Hz, 1H), 4.31 (d, *J* = 17.2 Hz, 1H), 2.96 – 2.86 (m, 1H), 2.60 (ddd, *J* = 17.3, 4.5, 2.3 Hz, 1H), 2.38 (tt, *J* = 13.2, 6.5 Hz, 1H), 2.25 (s, 3H), 2.22 (s, 3H), 2.00 (dtd, *J* = 12.7, 5.4, 2.3 Hz, 1H). ^{13}C NMR (126 MHz, DMSO-d_6) δ 173.4, 171.6, 168.4, 159.7, 143.7, 143.1, 129.5, 126.6, 124.1, 121.1, 119.7, 114.1, 99.7, 52.0, 47.6, 31.7, 23.0, 12.2.

N-(2-(2,6-Dioxopiperidin-3-yl)-1-oxoisoindolin-5-yl)-5-fluoro-3-methyl-1*H*-indole-2-carboxamide (**27**)

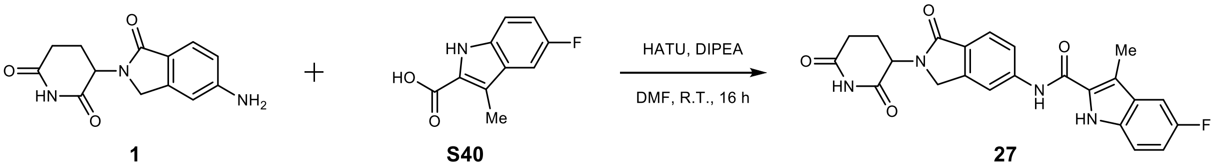

C5 Lenalidomide**1** (30.0 mg, 0.116 mmol, 1.0 eq) was added to a stirring solution of 5-fluoro-3-methyl-1*H*-indole-2-carboxylic acid **S40** (22.4 mg, 0.116 mmol, 1.0 eq), HATU (66.0 mg, 0.174 mmol, 1.5 eq), and DIPEA (60.6 µL, 0.347 mmol, 3.0 eq) in DMF (0.5 mL). The reaction mixture was stirred for 16 h and then purified using automated reversed-phase flash chromatography (Biotage Sfar C18 30g column, acetonitrile/water gradient mobile phase with 0.1% formic acid additive). Product-containing fractions were lyophilized to obtain a white solid (15.0 mg, 30% yield). ^{1}H NMR (500 MHz, DMSO-d_6) δ 11.61 (s, 1H), 10.99 (s, 1H), 10.32 (s, 1H), 8.12 (d, *J* = 1.7 Hz, 1H), 7.78 (dd, *J* = 8.3, 1.8 Hz, 1H), 7.73 (d, *J* = 8.2 Hz, 1H), 7.44 (ddd, *J* = 12.2, 9.3, 3.5 Hz, 2H), 7.12 (td, *J* = 9.2, 2.6 Hz, 1H), 5.10 (dd, *J* = 13.3, 5.1 Hz, 1H), 4.48 (d, *J* = 17.3 Hz, 1H), 4.34 (d, *J* = 17.3 Hz, 1H), 2.91 (ddd, *J* = 17.3, 13.7, 5.4 Hz, 1H), 2.67 – 2.58 (m, 1H), 2.52 (s, 3H), 2.45 – 2.35 (m, 1H), 2.02 (ddq, *J* = 10.4, 5.3, 2.7 Hz, 1H). ^{13}C NMR (126 MHz, DMSO-d_6) δ 173.0, 171.2, 168.0, 160.8, 158.0,

156.1, 142.8 (d, J = 135.8 Hz), 132.4, 129.4, 128.2 (d, J = 9.7 Hz), 126.8, 123.8, 119.7, 115.7 (d, J = 5.3 Hz), 114.1, 113.5 (d, J = 9.5 Hz), 113.0 (d, J = 26.6 Hz), 104.4 (d, J = 23.1 Hz), 51.7, 47.3, 31.3, 22.6, 9.8.

N-(2-(2,6-Dioxopiperidin-3-yl)-1-oxoisoindolin-5-yl)-3,5-dimethyl-1*H*-pyrrole-2-carboxamide (**28**)

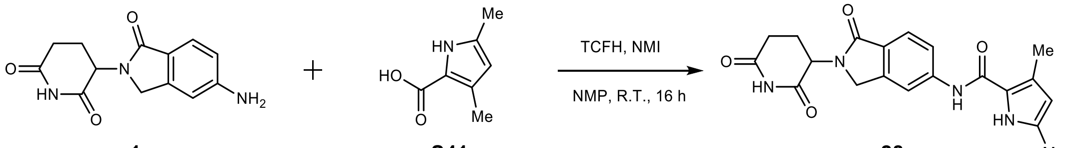

A solution of C5 Lenalidomide**1** (30.0 mg, 0.116 mmol, 1.0 eq), 3,5-dimethyl-1*H*-pyrrole-2-carboxylic acid **S41** (16.1 mg, 0.116 mmol, 1.0 eq), TCFH (48.7 mg, 0.174 mmol, 1.5 eq), and NMI (27.7 µL, 0.347 mmol, 3.0 eq) was stirred in NMP (0.5 mL) for 16 h. The reaction mixture was purified using automated reversed-phase flash chromatography (Biotage Sfar C18 30g column, acetonitrile/water gradient mobile phase with 0.1% formic acid additive). Product-containing fractions were lyophilized to obtain a white solid (4.0 mg, 10% yield). ^{1}H NMR (500 MHz, DMSO-d_6) δ 11.19 – 11.15 (m, 1H), 10.98 (s, 1H), 9.58 (s, 1H), 8.04 (s, 1H), 7.66 (d, J = 1.2 Hz, 2H), 5.77 (d, J = 2.4 Hz, 1H), 5.09 (dd, J = 13.3, 5.1 Hz, 1H), 4.44 (d, J = 17.2 Hz, 1H), 4.30 (d, J = 17.1 Hz, 1H), 2.91 (ddd, J = 17.3, 13.6, 5.4 Hz, 1H), 2.64 – 2.56 (m, 1H), 2.43 – 2.32 (m, 1H), 2.28 (s, 3H), 2.21 (s, 3H), 2.04 – 1.96 (m, 1H). ^{13}C NMR (126 MHz, DMSO-d_6) δ 172.9, 171.2, 168.0, 159.7, 143.2, 143.1, 131.0, 126.3, 125.7, 123.6, 120.3, 119.0, 113.4, 110.8, 51.5, 47.1, 31.2, 22.6, 13.0, 12.8.

N-(2-(2,6-Dioxopiperidin-3-yl)-1-oxoisoindolin-5-yl)-6-fluoro-3-methyl-1*H*-indole-2-carboxamide (**29**)

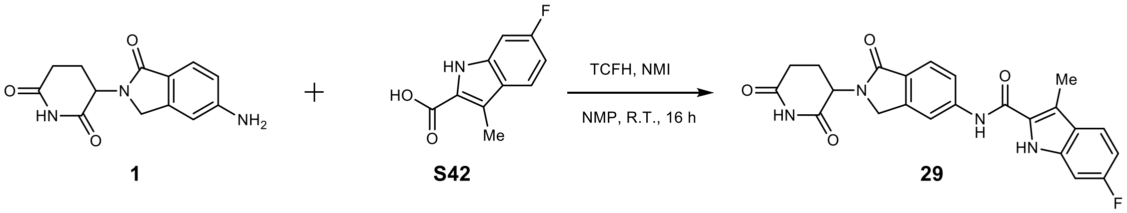

A solution of C5 Lenalidomide**1** (30.0 mg, 0.116 mmol, 1.0 eq), 6-fluoro-3-methyl-1*H*-indole-2-carboxylic acid **S42** (24.6 mg, 0.127 mmol, 1.0 eq), TCFH (64.9 mg, 0.231 mmol, 2.0 eq), and NMI (27.7 µL, 0.347 mmol, 3.0 eq) was stirred in NMP (0.5 mL) for 16 h. The reaction mixture was purified using automated reversed-phase flash chromatography (Biotage Sfar C18 30g column, acetonitrile/water gradient mobile phase with 0.1% formic acid additive). Product-containing fractions were lyophilized to obtain a white solid (26.0 mg, 52% yield). ^{1}H NMR (500 MHz, DMSO-d_6) δ 11.57 (s, 1H), 10.99 (s, 1H), 10.24 (s, 1H), 8.11 (d, J = 1.7 Hz, 1H), 7.82 – 7.71 (m, 2H), 7.68 (dd, J = 8.8, 5.5 Hz, 1H), 7.21 (dd, J = 9.9, 2.4 Hz, 1H), 6.96 (ddd, J = 9.7, 8.7, 2.3 Hz, 1H), 5.10 (dd, J = 13.3, 5.2 Hz, 1H), 4.48 (d, J = 17.2 Hz, 1H), 4.34 (d, J = 17.2 Hz, 1H), 2.91 (ddd, J = 17.3, 13.7, 5.5 Hz, 1H), 2.61 (ddd, J = 17.4, 4.6, 2.4 Hz, 1H), 2.55 (s, 3H), 2.40 (qd, J = 13.2, 4.4 Hz, 1H), 2.01 (dtd, J = 12.6, 5.3, 2.3 Hz, 1H). ^{13}C NMR (126 MHz, DMSO-d_6) δ 173.0, 171.2, 168.0, 161.7, 160.7, 159.8, 142.8 (d, J = 130.0 Hz), 135.7 (d, J = 13.0 Hz), 128.2 (d, J = 3.6 Hz), 126.7, 125.0, 123.8, 121.7 (d, J = 10.5 Hz), 119.6, 116.3, 114.1, 108.6 (d, J = 24.9 Hz), 97.8 (d, J = 25.6 Hz), 51.7, 47.3, 31.3, 22.6, 9.9.

N-(2-(2,6-Dioxopiperidin-3-yl)-1-oxoisoindolin-5-yl)-3-ethyl-1*H*-pyrrolo[2,3-*b*]pyridine-2-carboxamide (**30**)

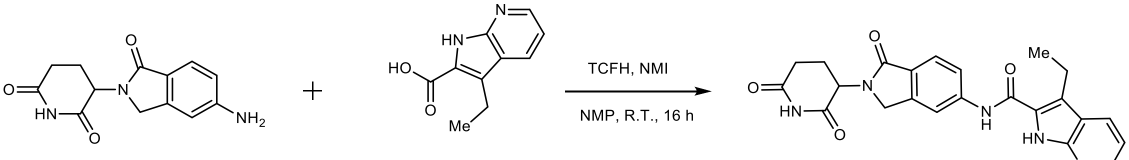

1 **S43** **30**

A solution of C5 Lenalidomide**1** (30.0 mg, 0.116 mmol, 1.0 eq), 3-ethyl-1*H*-pyrrolo[2,3-*b*]pyridine-2-carboxylic acid **S43** (22.0 mg, 0.116 mmol, 1.0 eq), TCFH (35.7 mg, 0.127 mmol, 1.1 eq), and NMI (27.7 µL, 0.347 mmol, 3.0 eq) was stirred for 16 h in NMP (0.5 mL). The reaction mixture was purified using automated reversed-phase flash chromatography (Biotage Sfar C18 30g column, acetonitrile/water gradient mobile phase with 0.1% formic acid additive). Product-containing fractions were lyophilized to obtain a white solid (9.0 mg, 18% yield). ^{1}H NMR (500 MHz, DMSO-d_6) δ 12.05 (s, 1H), 11.00 (s, 1H), 10.37 (s, 1H), 8.40 (dd, *J* = 4.6, 1.6 Hz, 1H), 8.19 – 8.12 (m, 2H), 7.78 (dd, *J* = 8.3, 1.8 Hz, 1H), 7.73 (d, *J* = 8.3 Hz, 1H), 7.15 (dd, *J* = 7.9, 4.6 Hz, 1H), 5.11 (dd, *J* = 13.3, 5.2 Hz, 1H), 4.48 (d, *J* = 17.2 Hz, 1H), 4.35 (d, *J* = 17.2 Hz, 1H), 3.10 (q, *J* = 7.5 Hz, 2H), 2.98 – 2.87 (m, 1H), 2.61 (ddd, *J* = 17.3, 4.5, 2.3 Hz, 1H), 2.40 (qd, *J* = 13.2, 4.4 Hz, 1H), 2.02 (dtd, *J* = 12.7, 5.4, 2.3 Hz, 1H), 1.23 (t, *J* = 7.5 Hz, 3H). ^{13}C NMR (126 MHz, DMSO-d_6) δ 172.9, 171.2, 167.9, 160.2, 147.6, 146.3, 143.3, 142.2, 128.7, 126.7, 126.3, 123.7, 122.5, 119.5, 119.3, 116.1, 113.9, 51.6, 47.2, 31.2, 22.6, 17.4, 15.6.

N-(2-(2,6-dioxopiperidin-3-yl)-1-oxoisoindolin-5-yl)-3,7-dimethyl-1H-indole-2-carboxamide (**31**)

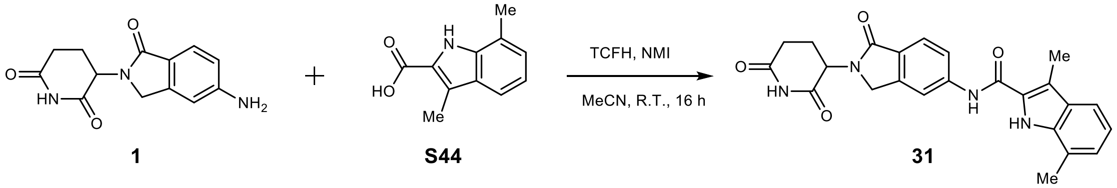

1 **S44** **31**

A mixture of C5 Lenalidomide**1** (30.0 mg, 0.110 mmol, 1.0 eq), 3,7-dimethyl-1H-indole-2-carboxylic acid **S44** (21.0 mg, 0.110 mmol, 1.0 eq) and 1-methylimidiazole (26 µL, 0.33 mmol, 3.0 eq) was stirred at room temperature in MeCN (0.5 mL) for 1 min. then TCFH (62.0 Mg) was added. The reaction mixture was stirred at room temperature for 16 h, then was diluted with ethyl acetate (5 mL) and washed with brine (5 Ml x 3). The organic phase was separated and filtered to obtain a gray solid which was then triturated with diethyl ether to obtain a gray solid (24.0 mg, 48%) ^{1}H NMR (500 MHz, DMSO-d_6) δ 1.99 - 2.06 (m, 1H), 2.43 (td, *J* = 13.2, 4.4 Hz, 1H), 2.59 (s, 6H), 2.62 - 2.64 (m, 1H), 2.87 - 3.00 (m, 1H), 4.35 (d, *J* = 17.2 Hz, 1H), 4.49 (d, *J* = 17.2 Hz, 1H), 5.12 (dd, *J* = 13.3, 5.1 Hz, 1H), 6.95 - 7.03 (m, 1H), 7.07 (d, *J* = 7.0 Hz, 1H), 7.47 (d, *J* = 7.9 Hz, 1H), 7.72 (d, *J* = 8.3 Hz, 1H), 8.01 (s, 1H), 8.29 (s, 1H), 10.93 (s, 1H), 11.00 (s, 1H), 11.85 (s, 1H). ^{13}C NMR (126 MHz, DMSO-d_6) δ 173.4, 171.6, 168.4, 161.2, 143.6, 143.2, 136.0, 128.1, 127.0, 126.9, 125.2, 123.9, 122.1, 120.1, 120.0, 118.6, 117.8, 114.5, 52.0, 47.7, 31.7, 23.0, 17.8, 10.7.

7-Bromo-*N*-(2-(2,6-dioxopiperidin-3-yl)-1-oxoisoindolin-5-yl)-1*H*-indole-2-carboxamide (**32**)

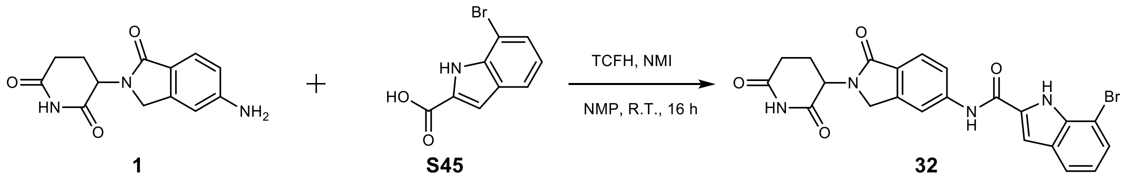

1 **S45** **32**

A solution of C5 Lenalidomide**1** (30.0 mg, 0.116 mmol, 1.0 eq), 7-bromo-1*H*-indole-2-carboxylic acid **S45** (27.8 mg, 0.116 mmol, 1.0 eq), TCFH (35.7 mg, 0.127 mmol, 1.1 eq), and NMI (27.7 µL, 0.347 mmol, 3.0 eq) was stirred in NMP (0.5 mL) for 16 h. The reaction mixture was purified using automated reversed-phase flash chromatography (Biotage Sfar C18 30g column, acetonitrile/water gradient mobile phase with 0.1% formic acid additive). Product-containing fractions were lyophilized to obtain a white solid (10.0 mg, 18% yield). ^{1}H NMR (500 MHz, DMSO-d_6) δ 11.71 (s, 1H), 11.00 (s, 1H), 10.66 (s, 1H), 8.15 (d, *J* = 1.8 Hz, 1H), 7.86 (dd, *J* = 8.3, 1.8 Hz, 1H), 7.74 (t, *J* = 8.0 Hz, 2H), 7.53 – 7.48 (m, 2H), 7.06 (t, *J* = 7.7 Hz, 1H), 5.11 (dd, *J* = 13.3, 5.1 Hz, 1H), 4.50 (d, *J* = 17.2 Hz, 1H), 4.35 (d, *J* = 17.1 Hz, 1H), 2.93 (ddd, *J* = 17.3, 13.7, 5.4 Hz, 1H), 2.61 (ddd, *J* = 17.4, 4.5, 2.2 Hz, 1H), 2.40 (qd, *J* = 13.2, 4.5 Hz, 1H), 2.02 (ddq, *J* = 10.7, 5.5, 3.1 Hz, 1H). ^{13}C NMR (126 MHz, DMSO-d_6) δ 172.9, 171.1, 167.8, 159.1, 143.3, 142.1, 135.5, 132.8, 128.4, 127.0, 126.9, 123.7, 121.6, 121.4, 119.8, 114.3, 107.4, 104.7, 51.6, 47.2, 31.3, 22.6.

N-(2-(2,6-Dioxopiperidin-3-yl)-1-oxoisoindolin-5-yl)-3,4-dimethyl-1*H*-pyrrole-2-carboxamide (**33**)

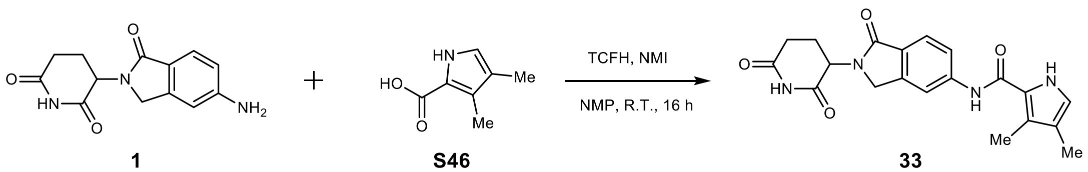

A solution of C5 Lenalidomide**1** (30.0 mg, 0.116 mmol, 1.0 eq), 3,4-dimethyl-1*H*-pyrrole-2-carboxylic acid **S46** (16.1 mg, 0.116 mmol, 1.0 eq), TCFH (48.7 mg, 0.174 mmol, 1.5 eq), and NMI (27.7 µL, 0.347 mmol, 3.0 eq) was stirred in NMP (0.5 mL) for 16 h. The reaction mixture was purified using automated reversed-phase flash chromatography (Biotage Sfar C18 30g column, acetonitrile/water gradient mobile phase with 0.1% formic acid additive). Product-containing fractions were lyophilized to obtain a white solid (9.0 mg, 20% yield). ^{1}H NMR (500 MHz, DMSO-d_6) δ 11.09 (d, *J* = 3.1 Hz, 1H), 10.97 (s, 1H), 9.97 (d, *J* = 311.3 Hz, 1H), 8.08 – 7.93 (m, 1H), 7.76 – 7.62 (m, 2H), 6.80 – 6.56 (m, 1H), 5.13 – 5.04 (m, 1H), 4.43 (dd, *J* = 17.2, 9.9 Hz, 1H), 4.29 (dd, *J* = 17.2, 11.9 Hz, 1H), 2.91 (ddd, *J* = 16.8, 13.4, 5.1 Hz, 1H), 2.60 (ddd, *J* = 17.2, 4.4, 2.5 Hz, 1H), 2.38 (ddq, *J* = 18.0, 8.1, 4.4 Hz, 1H), 2.17 (d, *J* = 72.9 Hz, 3H), 2.04 – 1.92 (m, 4H). ^{13}C NMR (126 MHz, DMSO-d_6) δ 172.9, 171.2, 168.0, 160.0, 143.3, 142.9, 125.9, 124.1, 123.6, 121.9, 119.6, 119.0, 118.9, 113.4, 51.5, 47.2, 42.5, 31.2, 22.6, 10.4, 9.8.

N-(2-(2,6-Dioxopiperidin-3-yl)-1-oxoisoindolin-5-yl)-1*H*-indole-2-carboxamide (**34**)

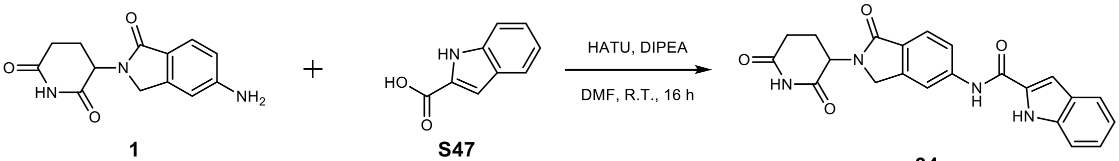

C5 Lenalidomide**1** (30.0 mg, 0.116 mmol, 1.0 eq) was added to a stirring solution of 1*H*-indole-2-carboxylic acid **S47** (18.7 mg, 0.116 mmol, 1.0 eq), HATU (66.0 mg, 0.174 mmol, 1.5 eq), and DIPEA (40.4 µL, 0.231 mmol, 2.0 eq) in DMF (0.5 mL). The reaction mixture was stirred for 16 h and then diluted with water (5 mL) and extracted with EtOAc (5 mL ×3). The combined organic layers were washed with brine, dried over anhydrous Na_2SO_4, filtered, and concentrated. The crude product was purified using automated flash chromatography (Sfar Silica HC D 10 g column, methanol/dichloromethane gradient mobile phase). Product-containing fractions were concentrated to obtain a white solid (27.0 mg, 58% yield). ^{1}H NMR (500 MHz,

DMSO-d_6) δ 11.79 (s, 1H), 11.00 (s, 1H), 10.51 (s, 1H), 8.15 (d, J = 1.7 Hz, 1H), 7.89 (dd, J = 8.3, 1.8 Hz, 1H), 7.74 (d, J = 8.3 Hz, 1H), 7.70 (d, J = 8.0 Hz, 1H), 7.51 – 7.46 (m, 2H), 7.24 (t, J = 7.7 Hz, 1H), 7.08 (t, J = 7.5 Hz, 1H), 5.10 (dd, J = 13.3, 5.1 Hz, 1H), 4.49 (d, J = 17.1 Hz, 1H), 4.35 (d, J = 17.1 Hz, 1H), 2.92 (ddd, J = 18.0, 13.6, 5.4 Hz, 1H), 2.65 – 2.58 (m, 1H), 2.40 (qd, J = 13.3, 4.5 Hz, 1H), 2.02 (ddd, J = 10.9, 5.6, 3.4 Hz, 1H). ^{13}C NMR (126 MHz, DMSO-d_6) δ 173.0, 171.2, 168.0, 160.0, 143.3, 142.3, 137.1, 131.1, 127.0, 126.7, 124.1, 123.7, 122.0, 120.1, 119.8, 114.3, 112.5, 104.6, 51.6, 47.3, 31.3, 22.6.

N-(2-(2,6-dioxopiperidin-3-yl)-1-oxoisoindolin-5-yl)-1H-indole-2-carboxamide (**35**)

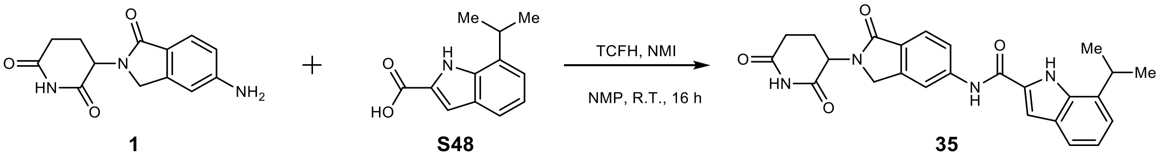

A mixture of C5 Lenalidomide**1** (19.0 mg, 0.074 mmol, 1.0 eq), 7-isopropyl-1H-indole-2-carboxylic acid **S48** (15.0 mg, 0.074 mmol, 1.0 eq), TCFH (23.0 m g, 0.081 mmol, 1.1 eq) and NMI (0.018 mL, 0.22 mmol, 3.0 eq) was stirred at room temperature in NMP (0.5 mL) for 16 h. The reaction mixture was then diluted with ethyl acetate (3 mL) and washed with brine (3 mL ×3). The organic phase was dried over sodium sulfate, filtered and concentrated *in vacuo*. The crude was purified using automated flash chromatography (Biotage Sfar Amino D 11g column, methanol/dicholoromethane gradient mobile phase). Product-containing fractions were evaporated *in vacuo* to obtain a white solid (8.5 mg, 26%). ^{1}H NMR (500 MHz, DMSO-d_6) δ 1.29 (d, J = 6.8 Hz, 6H), 1.99 - 2.03 (m, 1H), 2.28 - 2.45 (m, 1H), 2.61 (d, J = 17.4 Hz, 1H), 2.93 (ddd, J = 17.8, 13.8, 5.4 Hz, 1H), 3.69 (p, J = 7.7 Hz, 1H), 4.35 (d, J = 17.2 Hz, 1H), 4.50 (d, J = 17.1 Hz, 1H), 5.12 (dd, J = 13.3, 5.1 Hz, 1H), 7.07 (t, J = 7.6 Hz, 1H), 7.14 (d, J = 7.2 Hz, 1H), 7.46 (d, J = 1.5 Hz, 1H), 7.51 (d, J = 7.9 Hz, 1H), 7.74 (d, J = 8.3 Hz, 1H), 7.89 (d, J = 8.4 Hz, 1H), 8.15 (s, 1H), 10.47 (s, 1H), 10.99 (s, 1H), 11.55 (s, 1H). ^{13}C NMR (126 MHz, DMSO-d_6) δ 173.4, 171.6, 168.3, 160.5, 143.7, 142.8, 136.0, 133.5, 131.7, 127.5, 127.1, 124.1, 121.0, 120.2, 120.1, 119.7, 114.6, 106.3, 52.1, 47.7, 31.7, 27.7, 23.6, 23.0.

4-Bromo-*N*-(2-(2,6-dioxopiperidin-3-yl)-1-oxoisoindolin-5-yl)-1*H*-pyrazole-5-carboxamide (**36**)

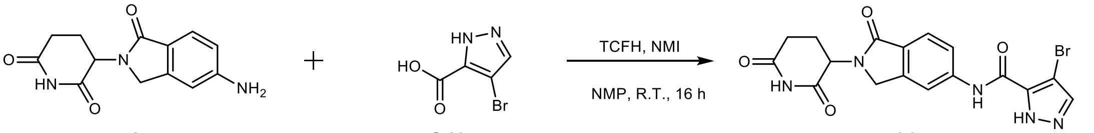

A solution of C5 Lenalidomide**1** (30.0 mg, 0.116 mmol, 1.0 eq), 4-bromo-1*H*-pyrazole-5-carboxylic acid **S49** (22.1 mg, 0.116 mmol, 1.0 eq), TCFH (35.7 mg, 0.127 mmol, 1.1 eq), and NMI (27.7 µL, 0.347 mmol, 3.0 eq) was stirred in NMP (0.5 mL) for 16 h. The reaction mixture was purified using automated reversed-phase flash chromatography (Biotage Sfar C18 30g column, acetonitrile/water gradient mobile phase with 0.1% formic acid additive). Product-containing fractions were lyophilized to obtain a white solid (9.0 mg, 17% yield). ^{1}H NMR (500 MHz, DMSO-d_6) δ 10.99 (s, 1H), 10.53 (s, 1H), 8.17 (s, 2H), 7.84 (dd, J = 8.4, 1.8 Hz, 1H), 7.68 (d, J = 8.3 Hz, 1H), 5.10 (dd, J = 13.3, 5.1 Hz, 1H), 4.45 (d, J = 17.2 Hz, 1H), 4.31 (d, J = 17.2 Hz, 1H), 2.91 (ddd, J = 17.2, 13.6, 5.5 Hz, 1H), 2.60 (ddd, J = 17.3, 4.5, 2.3 Hz, 1H), 2.40 (qd, J = 13.1, 4.5 Hz, 1H), 2.05 – 1.96 (m, 1H). ^{13}C NMR (126 MHz, DMSO-d_6) δ 172.9, 171.1, 167.9, 159.7, 143.1, 142.0, 126.7, 123.5, 119.8, 114.2, 93.3, 51.6, 47.2, 44.9, 31.2, 22.5.

N-(2-(2,6-dioxopiperidin-3-yl)-1-oxoisoindolin-5-yl)-3-phenyl-1H-indole-2-carboxamide (**37**)

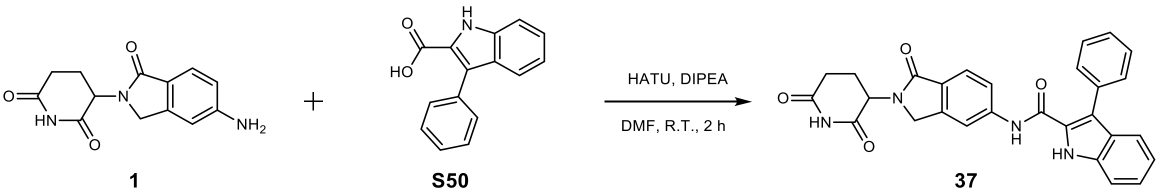

1 **S50** **37**

To 3-phenyl-1H-indole-2-carboxylic acid **S50** (27.0 mg, 0.12 mmol, 1.0 eq) in DMF (0.5 mL) at room temperature was added DIPEA (0.060 mL, 0.35 mmol, 3.0 eq), HATU (48.0 Mg, 0.13 mmol, 1.1 eq) and C5 Lenalidomide**1** (30.0 Mg, 0.12 mmol, 1.0 eq). The reaction mixture was stirred at room temperature for 2 h. The reaction mixture was diluted with ethyl acetate (3 mL) and washed sequentially with brine (3 mL ×3) and 5% LiCl (3 mL). The organic phase was dried over sodium sulfate, filtered and concentrated *in vacuo*. The crude was purified using automated flash chromatography (Biotage Sfar Amino D 11g column, methanol/dicholoromethane gradient mobile phase). Product-containing fractions were evaporated *in vacuo* obtain a white solid (4.3 mg, 8%). ^{1}H NMR (500 MHz, DMSO-d_6) δ 1.96 - 2.04 (m, 1H), 2.38 (qd, *J* = 13.4, 4.4 Hz, 1H), 2.56 - 2.67 (m, 1H), 2.91 (ddd, *J* = 17.5, 13.7, 5.4 Hz, 1H), 4.24 - 4.50 (m, 2H), 5.09 (dd, *J* = 13.3, 5.1 Hz, 1H), 7.14 (t, *J* = 7.5 Hz, 1H), 7.31 (t, *J* = 7.6 Hz, 1H), 7.35 (t, *J* = 7.4 Hz, 1H), 7.46 (t, *J* = 7.7 Hz, 2H), 7.50 - 7.57 (m, 4H), 7.62 (d, *J* = 8.2 Hz, 1H), 7.68 (d, *J* = 8.3 Hz, 1H), 7.97 (s, 1H), 10.10 (s, 1H), 10.98 (s, 1H), 12.00 (s, 1H). ^{13}C NMR (126 MHz, DMSO-d_6) δ 173.4, 171.6, 168.2, 161.30, 143.8, 142.4, 136.1, 134.1, 130.3, 128.9, 128.7, 127.2, 126.8, 124.6, 124.2, 121.0, 120.5, 119.8, 118.9, 114.2, 112.9, 52.0, 47.6, 31.7, 23.0.

N-(2-(2,6-Dioxopiperidin-3-yl)-1-oxoisoindolin-5-yl)-3-ethyl-4-methyl-1*H*-pyrazole-5-carboxamide (**38**)

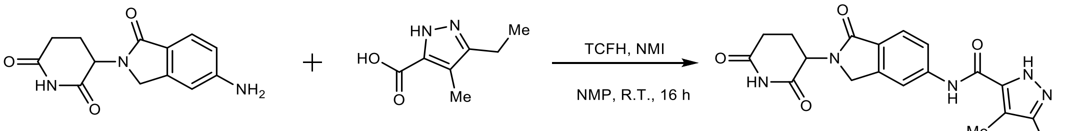

1 **S51** **38**

A solution of C5 Lenalidomide**1** (30.0 mg, 0.116 mmol, 1.0 eq), 3-ethyl-4-methyl-1*H*-pyrazole-5-carboxylic acid **S51** (17.8 mg, 0.116 mmol, 1.0 eq), TCFH (35.7 mg, 0.127 mmol, 1.1 eq), and NMI (27.7 µL, 0.347 mmol, 3.0 eq) was stirred in NMP (0.5 mL) for 16 h. The reaction mixture was purified using automated reversed-phase flash chromatography (Biotage Sfar C18 30g column, acetonitrile/water gradient mobile phase with 0.1% formic acid additive). Product-containing fractions were lyophilized to obtain a white solid (9.0 mg, 21% yield). ^{1}H NMR (500 MHz, DMSO-d_6) δ 13.01 (s, 1H), 10.98 (s, 1H), 10.21 (s, 1H), 8.19 (s, 1H), 7.86 (d, *J* = 8.3 Hz, 1H), 7.65 (d, *J* = 8.3 Hz, 1H), 5.09 (dd, *J* = 13.3, 5.1 Hz, 1H), 4.44 (d, *J* = 17.1 Hz, 1H), 4.30 (d, *J* = 17.1 Hz, 1H), 2.97 – 2.86 (m, 1H), 2.61 (q, *J* = 7.9 Hz, 3H), 2.39 (qd, *J* = 13.3, 4.5 Hz, 1H), 2.19 (s, 3H), 2.04 – 1.95 (m, 1H), 1.17 (t, *J* = 7.6 Hz, 3H). ^{13}C NMR (126 MHz, DMSO-d_6) δ 172.9, 171.2, 167.9, 162.2, 143.1, 142.4, 126.2, 119.6, 113.9, 113.3, 51.6, 47.2, 31.3, 22.5, 13.7, 8.3.

N-(2-(2,6-Dioxopiperidin-3-yl)-1-oxoisoindolin-5-yl)-1*H*-indazole-3-carboxamide (**39**)

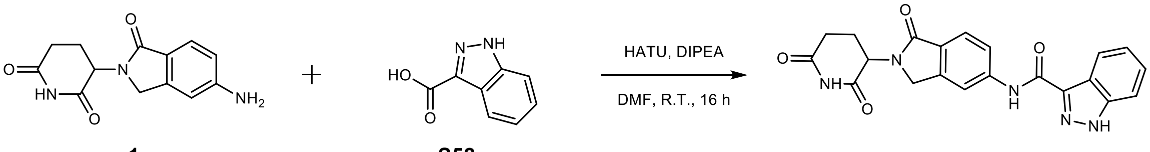

1 **S52** **39**

C5 Lenalidomide**1** (30.0 mg, 0.116 mmol, 1.0 eq) was added to a stirring solution of 1*H*-indazole-3-carboxylic acid **S52** (20.6 mg, 0.127 mmol, 1.1 eq), HATU (66.0 mg, 0.174 mmol, 1.5 eq), and DIPEA (60.6 µL, 0.347 mmol, 3.0 eq) in DMF (0.5 mL). The reaction mixture was stirred for 16 h and then purified using automated reversed-phase flash chromatography (Biotage Sfar C18 30g column, acetonitrile/water gradient mobile phase with 0.1% formic acid additive). Product-containing fractions were lyophilized to obtain a white solid (4.0 mg, 8% yield). ^{1}H NMR (500 MHz, DMSO-d_6) δ 13.87 (s, 1H), 10.99 (s, 1H), 10.71 (s, 1H), 8.29 (d, *J* = 1.8 Hz, 1H), 8.24 (d, *J* = 8.1 Hz, 1H), 7.97 (dd, *J* = 8.4, 1.8 Hz, 1H), 7.70 (t, *J* = 8.0 Hz, 2H), 7.48 (ddd, *J* = 8.2, 6.8, 1.1 Hz, 1H), 7.32 (t, *J* = 7.5 Hz, 1H), 5.11 (dd, *J* = 13.3, 5.1 Hz, 1H), 4.48 (d, *J* = 17.1 Hz, 1H), 4.33 (d, *J* = 17.1 Hz, 1H), 2.92 (ddd, *J* = 17.3, 13.6, 5.4 Hz, 1H), 2.65 – 2.55 (m, 1H), 2.41 (qd, *J* = 13.3, 4.5 Hz, 1H), 2.01 (dtd, *J* = 12.8, 5.4, 2.3 Hz, 1H). ^{13}C NMR (126 MHz, DMSO-d_6) δ 172.9, 171.2, 167.9, 161.3, 143.1, 142.2, 141.4, 138.0, 126.9, 126.5, 123.5, 122.6, 121.8, 121.4, 119.9, 114.3, 111.0, 51.6, 47.2, 31.3, 22.6.

N-(2-(2,6-Dioxopiperidin-3-yl)-1-oxoisoindolin-5-yl)-4-methyl-1*H*-pyrrole-2-carboxamide (**40**)

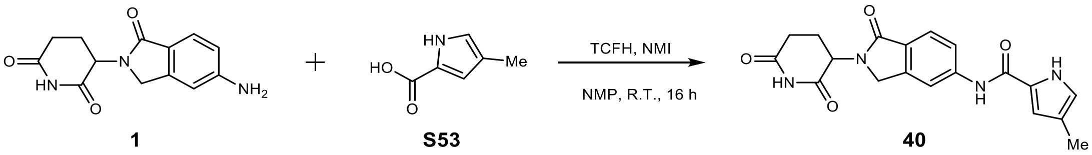

A solution of C5 Lenalidomide**1** (30.0 mg, 0.116 mmol, 1.0 eq), 4-methyl-1*H*-pyrrole-2-carboxylic acid **S53** (14.5 mg, 0.116 mmol, 1.0 eq), TCFH (48.7 mg, 0.174 mmol, 1.5 eq), and NMI (27.7 µL, 0.347 mmol, 3.0 eq) was stirred in NMP (0.5 mL) for 16 h. The reaction mixture was purified using automated reversed-phase flash chromatography (Biotage Sfar C18 30g column, acetonitrile/water gradient mobile phase with 0.1% formic acid additive). Product-containing fractions were lyophilized to obtain a white solid (5.0 mg, 12% yield). ^{1}H NMR (500 MHz, DMSO-d_6) δ 11.40 (t, *J* = 2.7 Hz, 1H), 10.98 (s, 1H), 9.93 (s, 1H), 8.08 (d, *J* = 1.7 Hz, 1H), 7.81 (dd, *J* = 8.3, 1.8 Hz, 1H), 7.70 – 7.60 (m, 1H), 6.94 (t, *J* = 2.1 Hz, 1H), 6.80 – 6.76 (m, 1H), 5.09 (dd, *J* = 13.3, 5.1 Hz, 1H), 4.45 (d, *J* = 17.1 Hz, 1H), 4.31 (d, *J* = 17.1 Hz, 1H), 2.92 (ddd, *J* = 17.3, 13.7, 5.4 Hz, 1H), 2.64 – 2.54 (m, 1H), 2.38 (qd, *J* = 13.2, 4.5 Hz, 1H), 2.07 (s, 3H), 2.00 (dtd, *J* = 12.5, 5.3, 2.2 Hz, 1H). ^{13}C NMR (126 MHz, DMSO-d_6) δ 172.9, 171.2, 168.0, 159.3, 143.1, 142.9, 125.9, 125.3, 123.5, 121.5, 119.3, 118.6, 113.7, 112.8, 51.5, 47.2, 31.3, 22.6, 11.7.

N-(2-(2,6-Dioxopiperidin-3-yl)-1-oxoisoindolin-5-yl)-2-(1*H*-indol-2-yl)acetamide (**41**)

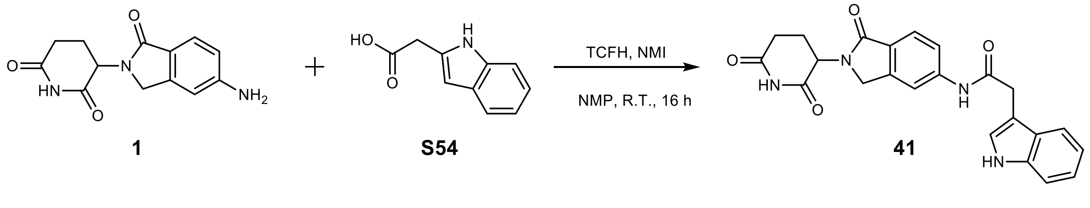

A solution of C5 Lenalidomide**1** (30.0 mg, 0.116 mmol, 1.0 eq), 2-(1*H*-indol-3-yl)acetic acid **S54** (20.3 mg, 0.116 mmol, 1.0 eq), TCFH (35.7 mg, 0.127 mmol, 1.1 eq), and NMI (27.7 µL, 0.347 mmol, 3.0 eq) was stirred in NMP (0.5 mL) for 16 h. The reaction mixture was purified using automated reversed-phase flash chromatography (Biotage Sfar C18 30g column, acetonitrile/water gradient mobile phase with 0.1% formic acid additive). Product-containing fractions were lyophilized to obtain a white solid (10.0 mg, 21% yield). ^{1}H NMR (500 MHz, DMSO-d_6) δ 10.97 (s, 1H), 10.94 (d, *J* = 2.4 Hz, 1H), 10.47 (s, 1H), 7.99 (d, *J* = 1.6 Hz, 1H), 7.63 (dt, *J* = 18.0, 8.1 Hz, 3H), 7.38 – 7.33 (m, 1H), 7.27 (d, *J* = 2.4 Hz, 1H), 7.07 (ddd, *J* = 8.1, 6.9, 1.2 Hz, 1H), 7.02 – 6.95 (m, 1H), 5.07 (dd, *J* = 13.3, 5.1 Hz, 1H), 4.41 (d, *J* = 17.3 Hz, 1H), 4.27 (d, *J* = 17.2 Hz, 1H), 3.78

(s, 2H), 2.90 (ddd, J = 17.3, 13.7, 5.4 Hz, 1H), 2.59 (ddd, J = 17.3, 4.5, 2.3 Hz, 1H), 2.36 (qd, J = 13.2, 4.5 Hz, 1H), 1.98 (dtd, J = 12.8, 5.4, 2.3 Hz, 1H). ^{13}C NMR (126 MHz, DMSO-d_6) δ 172.9, 171.1, 170.3, 167.9, 143.3, 142.6, 136.1, 127.2, 126.2, 124.0, 123.7, 121.0, 118.7, 118.7, 118.5, 113.2, 111.4, 108.3, 51.5, 47.1, 33.9, 31.2, 22.5.

3-Chloro-*N*-(2-(2,6-dioxopiperidin-3-yl)-1-oxoisoindolin-5-yl)-1-methyl-1*H*-indole-2-carboxamide (**42**)

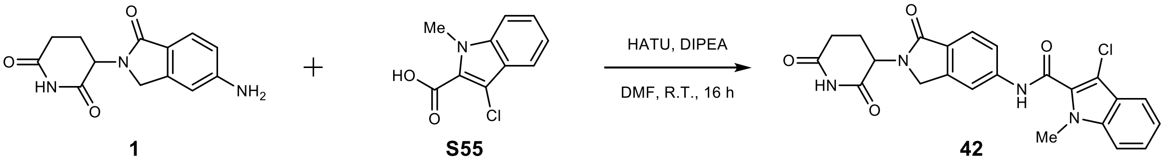

C5 Lenalidomide**1** (30.0 mg, 0.116 mmol, 1.0 eq) was added to a stirring solution of 3-chloro-1-methyl-1*H*-indole-2-carboxylic acid **S55** (24.3 mg, 0.116 mmol, 1.0 eq), HATU (66.0 mg, 0.174 mmol, 1.5 eq), and DIPEA (40.4 µL, 0.231 mmol, 2.0 eq) in DMF (0.5 mL). The reaction mixture was stirred for 16 h and then diluted with water (5 mL) and extracted with EtOAc (5 mL × 3). The combined organic layers were washed with brine, dried over anhydrous Na_2SO_4, filtered, and concentrated. The crude product was purified using automated flash chromatography (Sfar Silica HC D 10 g column, methanol/dichloromethane gradient mobile phase). Product-containing fractions were concentrated to obtain a white solid (12.0 mg, 23% yield). ^{1}H NMR (500 MHz, DMSO-d_6) δ 11.00 (s, 1H), 10.97 (s, 1H), 8.13 (s, 1H), 7.81 – 7.77 (m, 1H), 7.75 (d, J = 8.2 Hz, 1H), 7.69 – 7.61 (m, 2H), 7.41 (ddd, J = 8.4, 7.0, 1.1 Hz, 1H), 7.26 (t, J = 7.7 Hz, 1H), 5.11 (dd, J = 13.3, 5.2 Hz, 1H), 4.49 (d, J = 17.3 Hz, 1H), 4.35 (d, J = 17.3 Hz, 1H), 3.88 (s, 3H), 2.92 (ddd, J = 17.3, 13.6, 5.4 Hz, 1H), 2.61 (dt, J = 17.1, 3.5 Hz, 1H), 2.40 (qd, J = 13.2, 4.4 Hz, 1H), 2.05 – 1.98 (m, 1H). ^{13}C NMR (126 MHz, DMSO-d_6) δ 173.0, 171.1, 167.8, 161.9, 158.8, 143.5, 141.7, 137.0, 136.1, 130.1, 127.3, 126.0, 125.0, 124.7, 124.0, 123.9, 123.7, 121.3, 121.2, 119.6, 119.2, 118.6, 114.1, 111.4, 111.2, 110.2, 104.7, 51.7, 47.3, 32.5, 31.5, 31.3, 22.6.

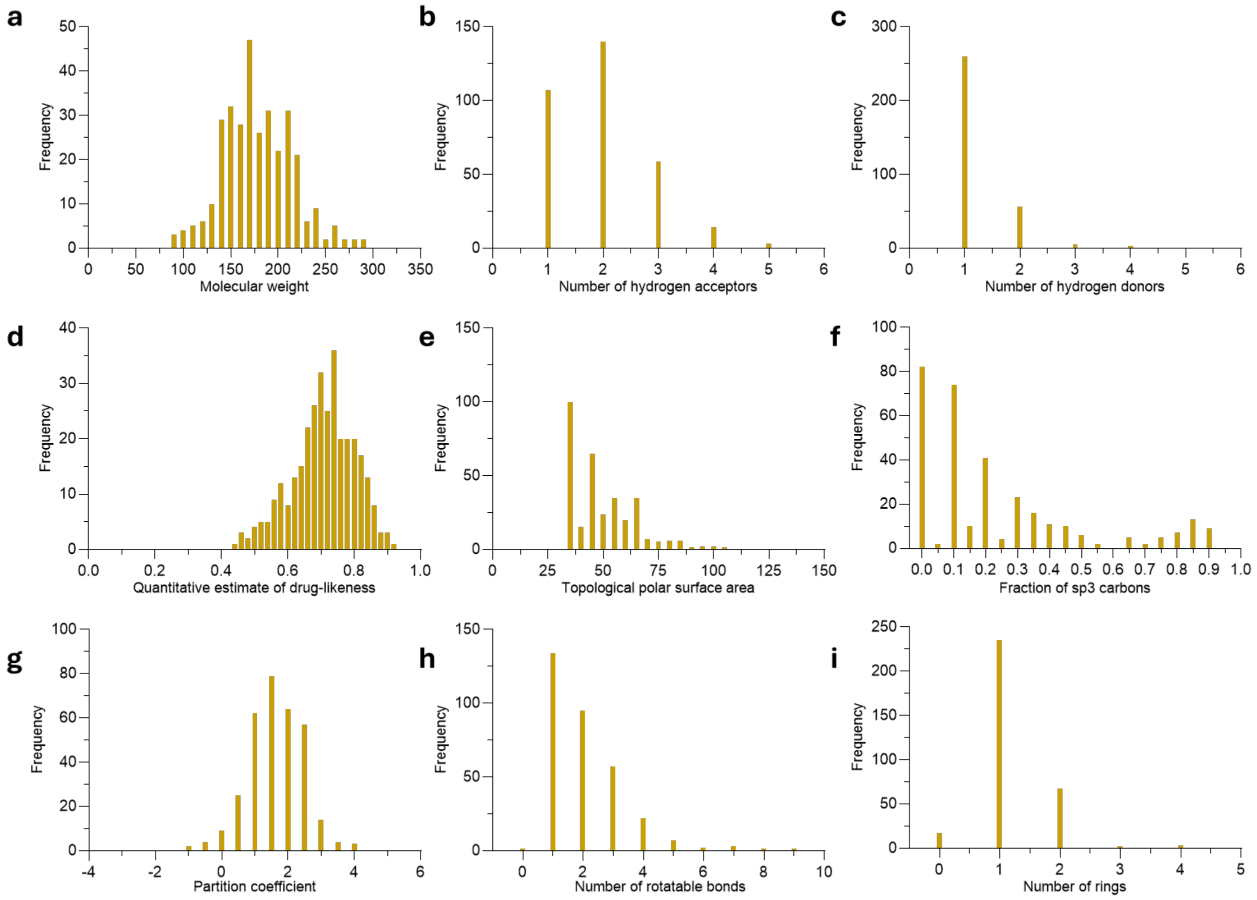

Supplementary Figure 12. Physiochemical Parameters of Carboxylic Acid Coupling Partners for Amidation Reactions. Histogram graph of (a) molecular weight, (b) hydrogen bond acceptors, (c) hydrogen bond donors, (d) quantitative estimate of drug-likeness, (e) topological polar surface area, (f) fraction of sp^3 carbons, (g) partition coefficient, (h) rotatable bonds, and (i) number of rings.

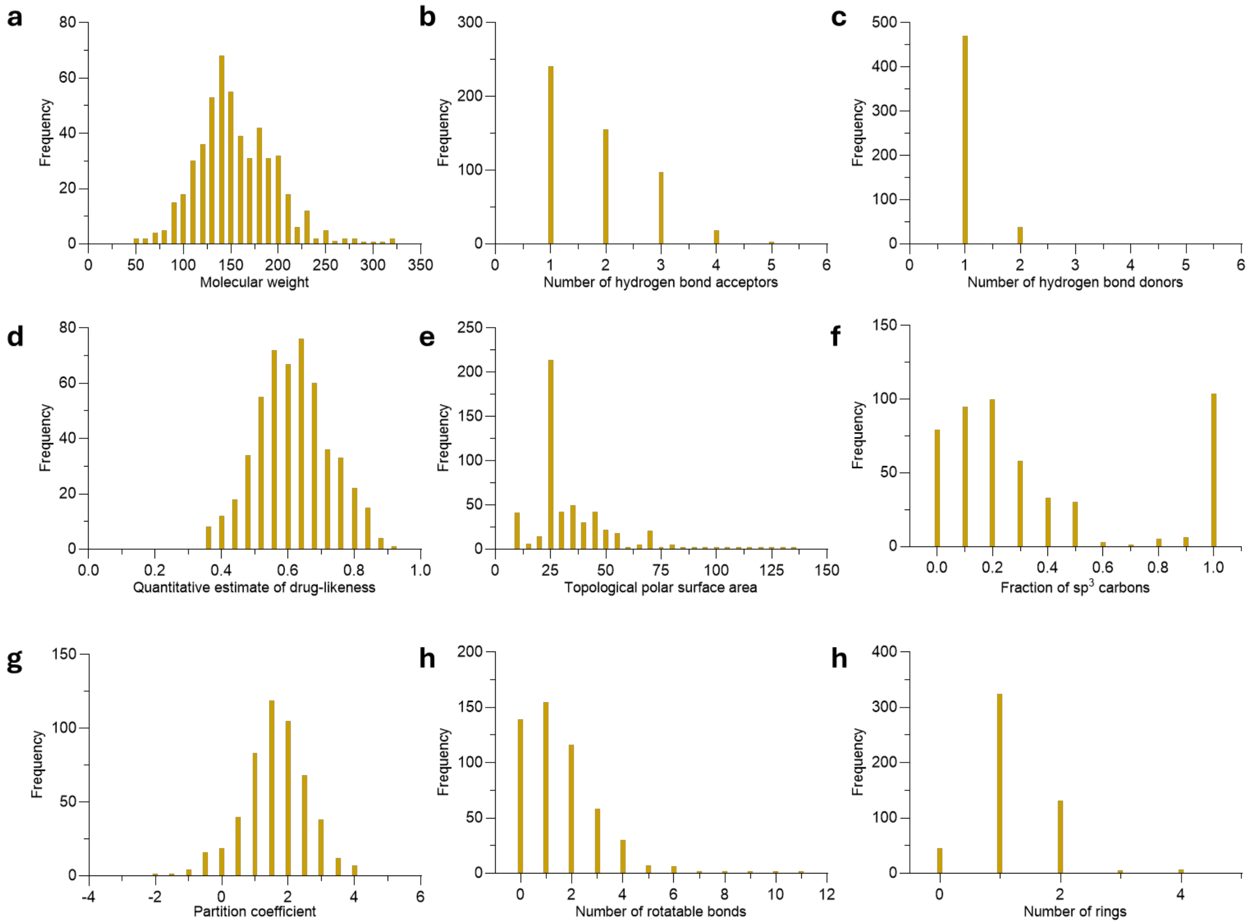

Supplementary Figure 13. Physiochemical Parameters of Amine Coupling Partners for Amidation Reactions. Histogram graph of (a) molecular weight, (b) hydrogen bond acceptors, (c) hydrogen bond donors, (d) quantitative estimate of drug-likeness, (e) topological polar surface area, (f) fraction of sp^3 carbons, (g) partition coefficient, (h) rotatable bonds, and (i) number of rings.

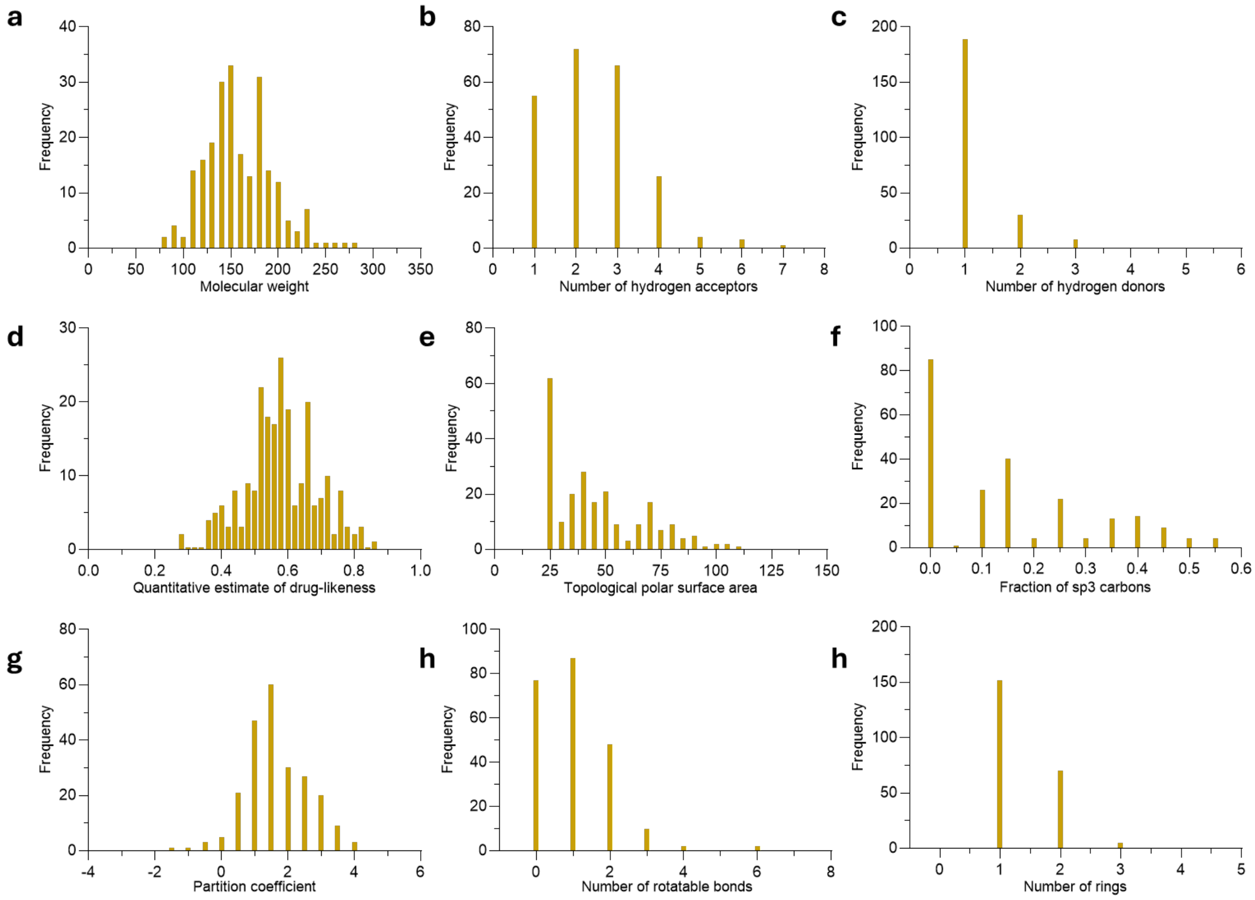

Supplementary Figure 14. Physiochemical Parameters of Amine Coupling Partners for Buchwald-Hartwig Reactions. Histogram graph of (a) molecular weight, (b) hydrogen bond acceptors, (c) hydrogen bond donors, (d) quantitative estimate of drug-likeness, (e) topological polar surface area, (f) fraction of sp^3 carbons, (g) partition coefficient, (h) rotatable bonds, and (i) number of rings.

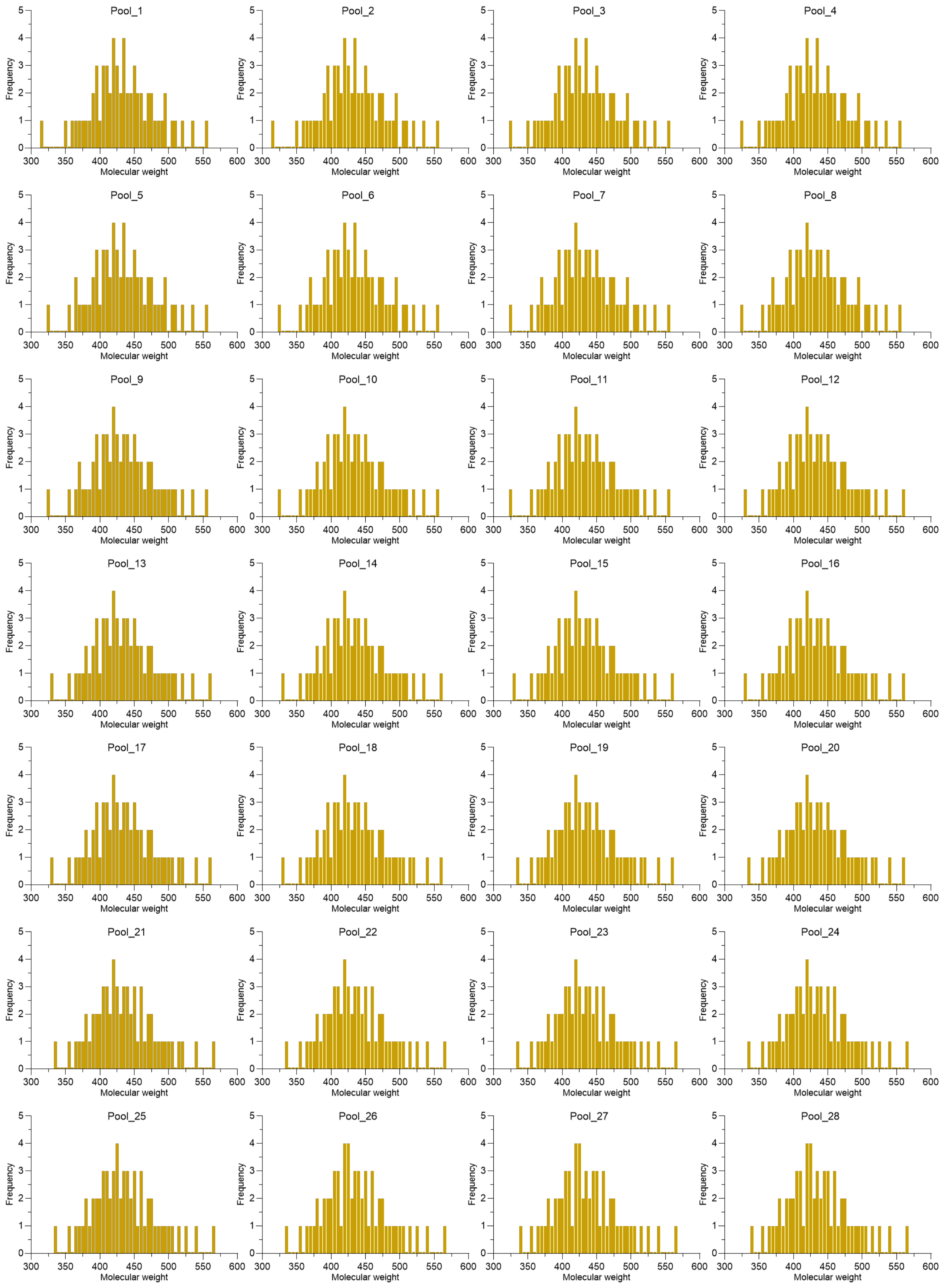

Supplementary Figure 15. Histogram Graph of ASMS Pools.

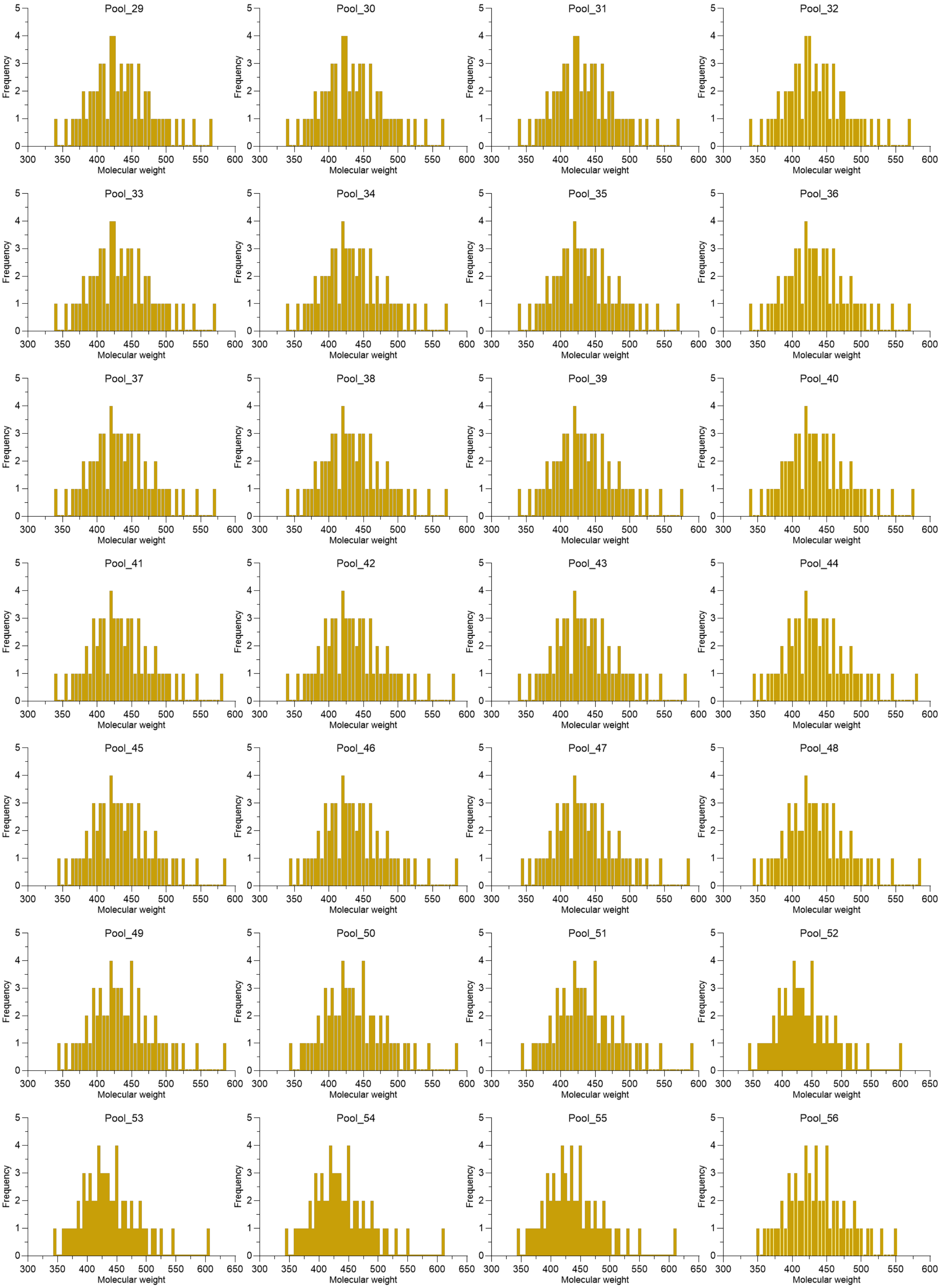

Supplementary Figure 15. Continued…

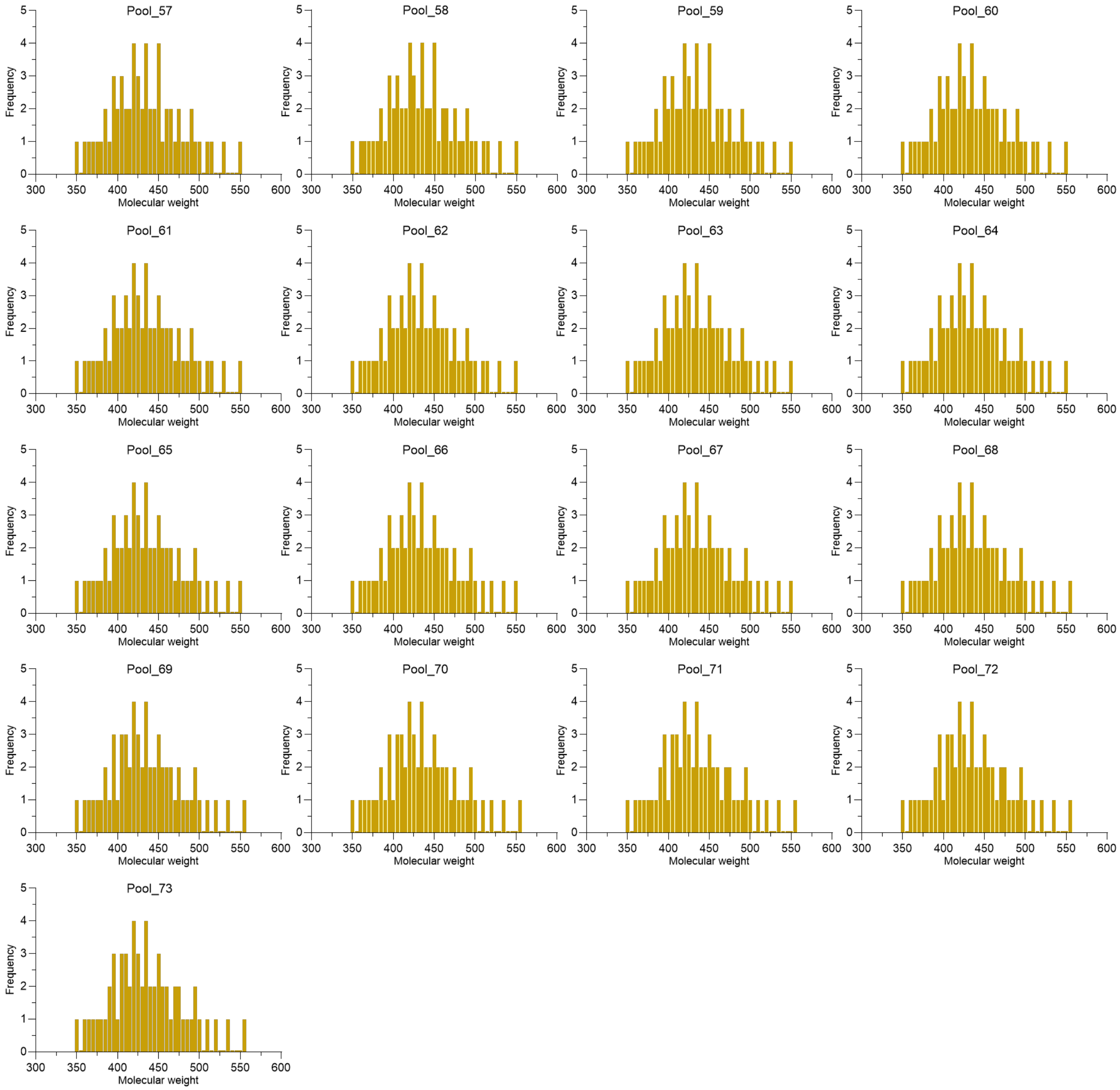

Supplementary Figure 15. Continued…

3. CID Spectra of Building Blocks

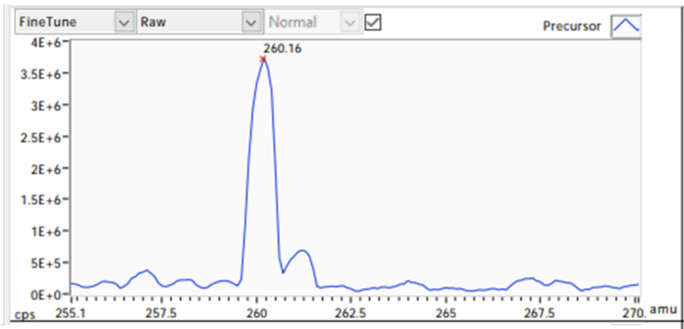

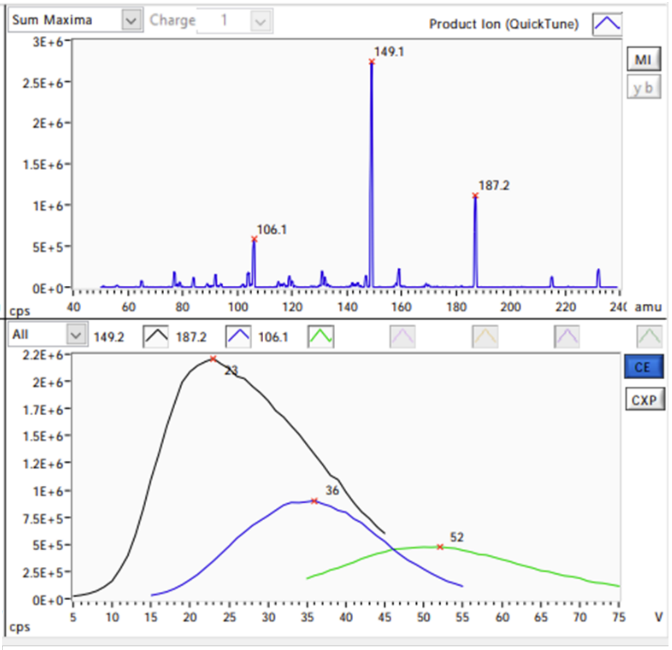

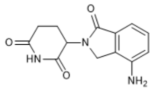

2

FineTune +

	Ion	Intensity	DP	EP	CE	CXP
Q1	260.158	3.7301E+6	90	10	-	-
Q3	149.147	2.2081E+6	90	10	23	9
Q3	187.163	9.0104E+5	90	10	36	11
Q3	106.090	4.7782E+5	90	10	52	12
Q3	-	-	-	-	-	-
Q3	-	-	-	-	-	-
Q3	-	-	-	-	-	-
Q3	-	-	-	-	-	-

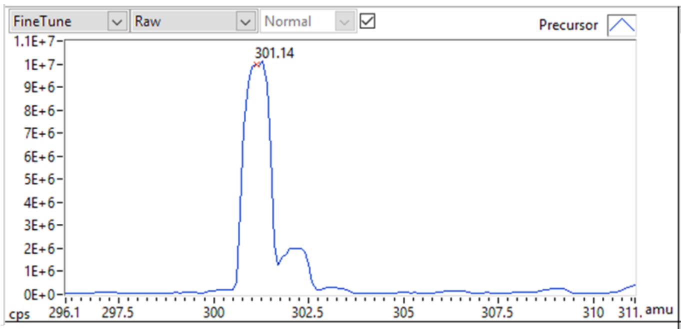

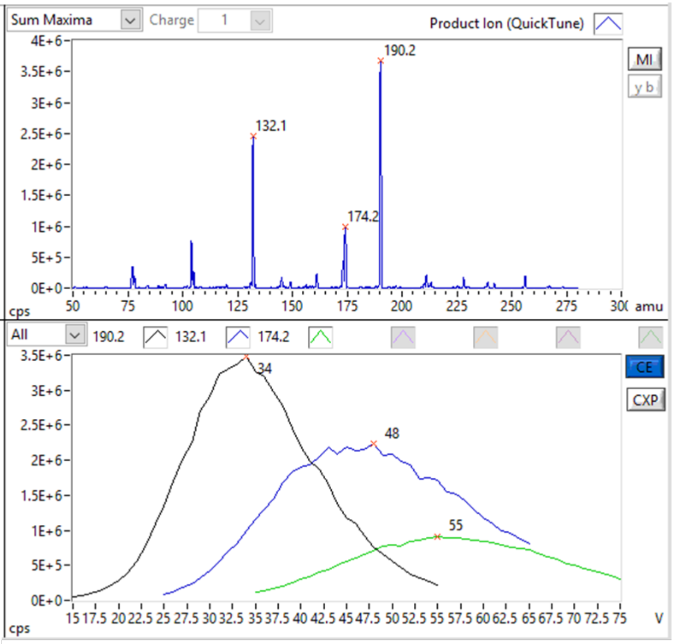

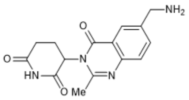

3

FineTune +

	Ion	Intensity	DP	EP	CE	CXP
Q1	301.138	9.9871E+6	100	10	-	-
Q3	190.169	3.4760E+6	100	10	34	12
Q3	132.112	2.2334E+6	100	10	48	8
Q3	174.161	9.1576E+5	100	10	55	12
Q3	-	-	-	-	-	-
Q3	-	-	-	-	-	-
Q3	-	-	-	-	-	-
Q3	-	-	-	-	-	-

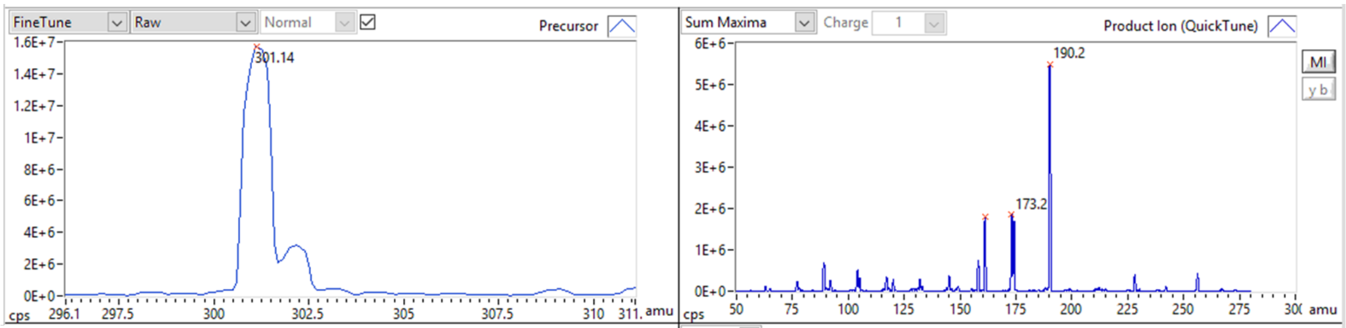

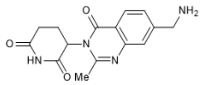

4

FineTune +

	Ion	Intensity	DP	EP	CE	CXP
Q1	301.143	1.5707E+7	50	10	-	-
Q3	190.157	4.8549E+6	50	10	34	12
Q3	173.151	1.6404E+6	50	10	42	11
Q3	161.146	1.5476E+6	50	10	51	10
Q3	-	-	-	-	-	-
Q3	-	-	-	-	-	-
Q3	-	-	-	-	-	-
Q3	-	-	-	-	-	-

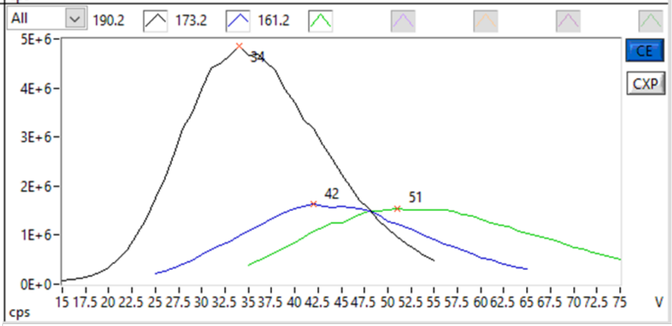

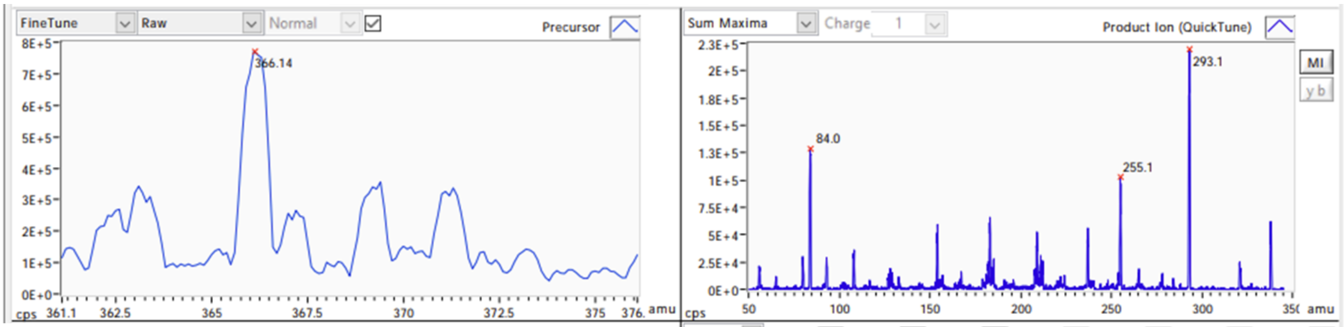

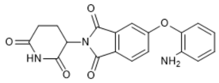

5

FineTune +

	Ion	Intensity	DP	EP	CE	CXP
Q1	366.138	7.7122E+5	110	10	-	-
Q3	293.119	1.9885E+5	110	10	34	16
Q3	84.043	1.3965E+5	110	10	19	10
Q3	255.134	8.0927E+4	110	10	26	13
Q3	-	-	-	-	-	-
Q3	-	-	-	-	-	-
Q3	-	-	-	-	-	-
Q3	-	-	-	-	-	-

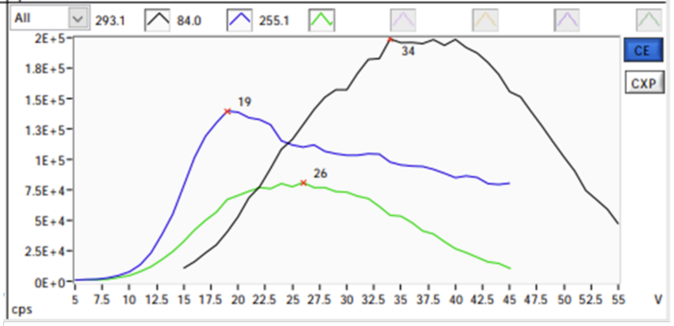

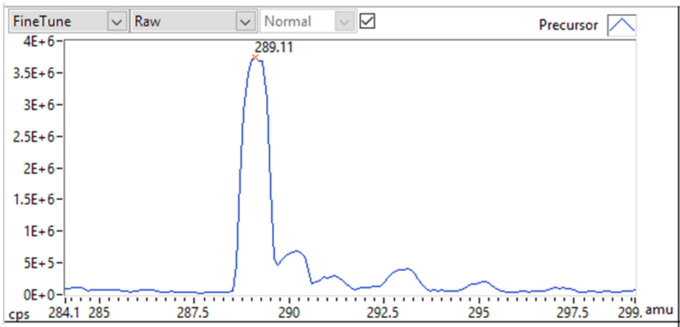

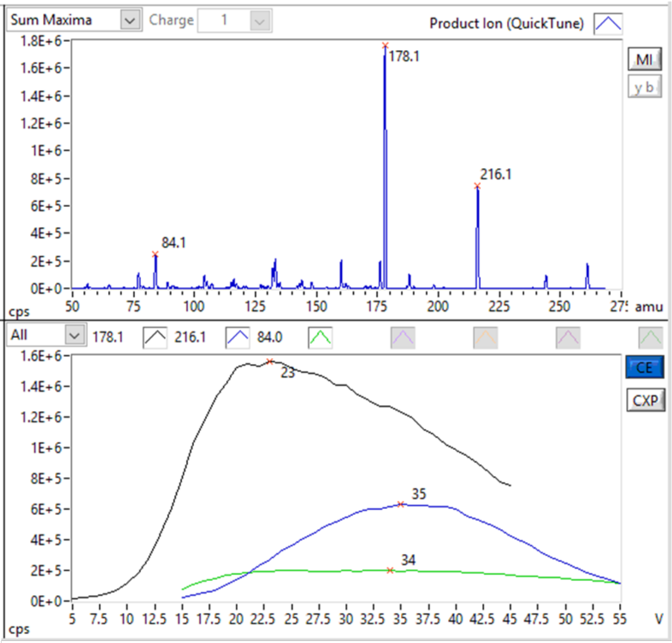

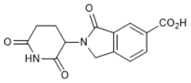

6

FineTune +

	Ion	Intensity	DP	EP	CE	CXP
Q1	289.109	3.7378E+6	100	10	-	-
Q3	178.109	1.5602E+6	100	10	23	10
Q3	216.122	6.2969E+5	100	10	35	14
Q3	84.051	2.0180E+5	100	10	34	10
Q3	-	-	-	-	-	-
Q3	-	-	-	-	-	-
Q3	-	-	-	-	-	-
Q3	-	-	-	-	-	-

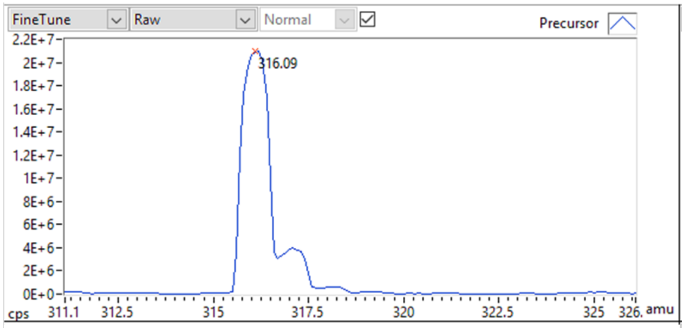

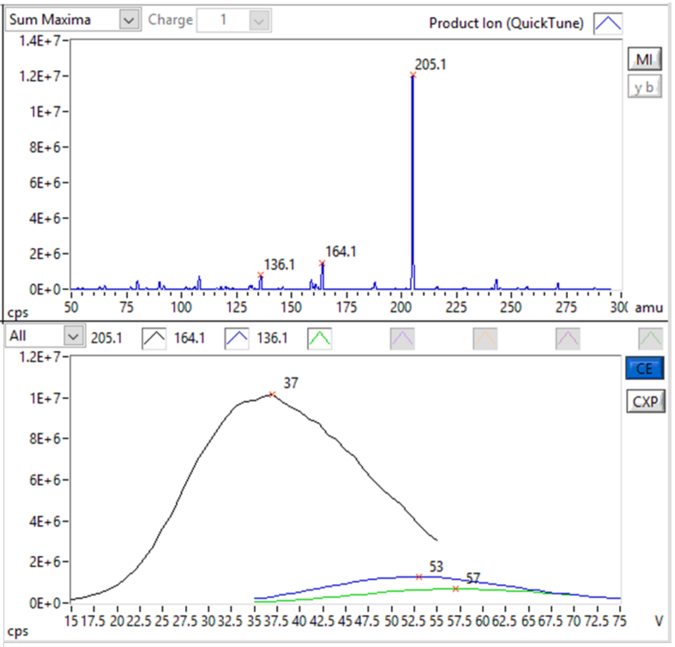

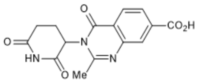

7

FineTune +

	Ion	Intensity	DP	EP	CE	CXP
Q1	316.092	2.0970E+7	120	10	-	-
Q3	205.131	1.0179E+7	120	10	37	12
Q3	164.125	1.2825E+6	120	10	53	15
Q3	136.120	7.0639E+5	120	10	57	12
Q3	-	-	-	-	-	-
Q3	-	-	-	-	-	-
Q3	-	-	-	-	-	-
Q3	-	-	-	-	-	-

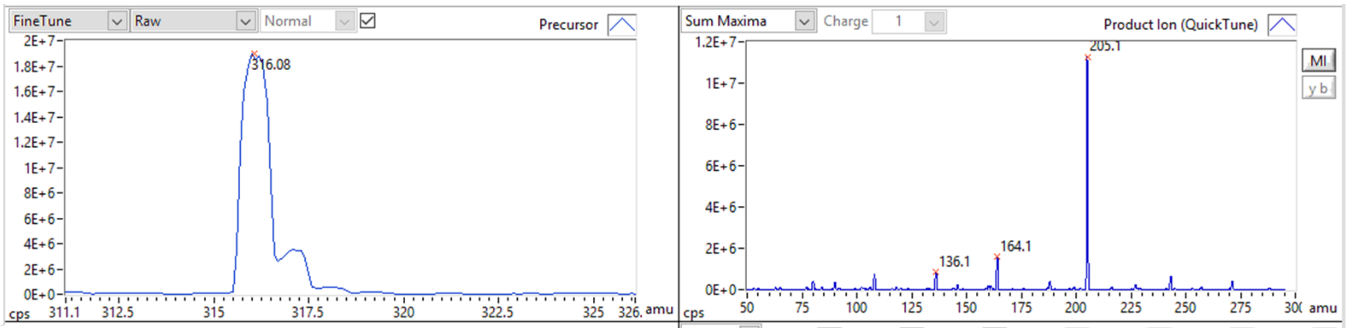

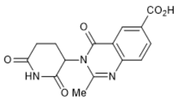

8

	Ion	Intensity	DP	EP	CE	CXP
Q1	316.083	1.9003E+7	120	10	-	-
Q3	205.117	8.9071E+6	120	10	36	13
Q3	164.109	1.4171E+6	120	10	52	11
Q3	136.105	7.3447E+5	120	10	58	12
Q3	-	-	-	-	-	-
Q3	-	-	-	-	-	-
Q3	-	-	-	-	-	-
Q3	-	-	-	-	-	-

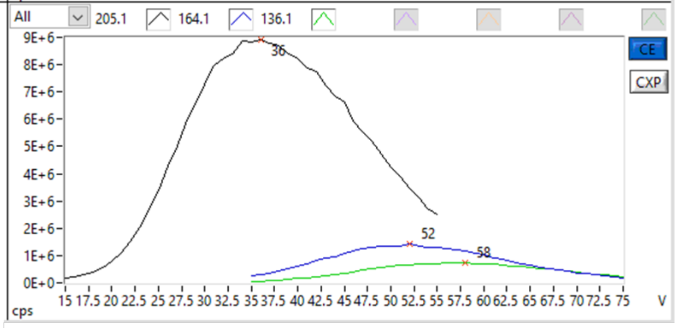

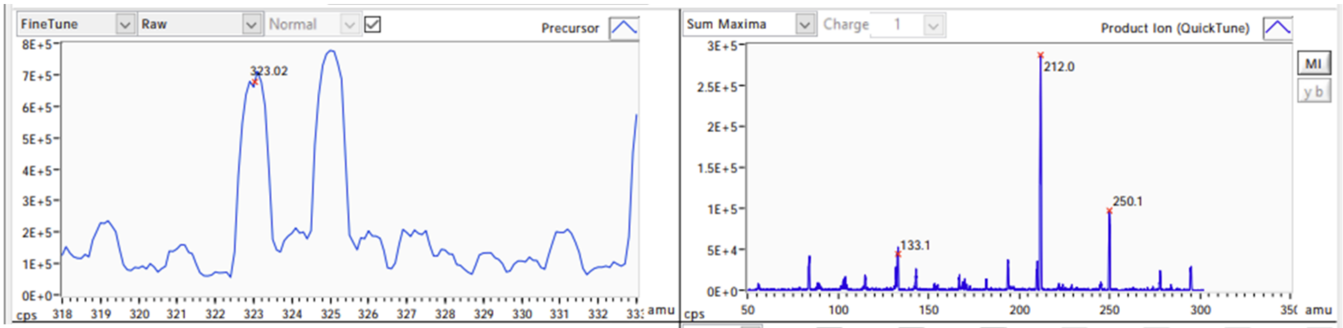

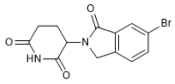

10

	Ion	Intensity	DP	EP	CE	CXP
Q1	323.017	6.7856E+5	100	10	-	-
Q3	212.013	2.4110E+5	100	10	26	12
Q3	250.080	6.9836E+4	100	10	38	15
Q3	133.105	4.0218E+4	100	10	57	8
Q3	-	-	-	-	-	-
Q3	-	-	-	-	-	-
Q3	-	-	-	-	-	-
Q3	-	-	-	-	-	-

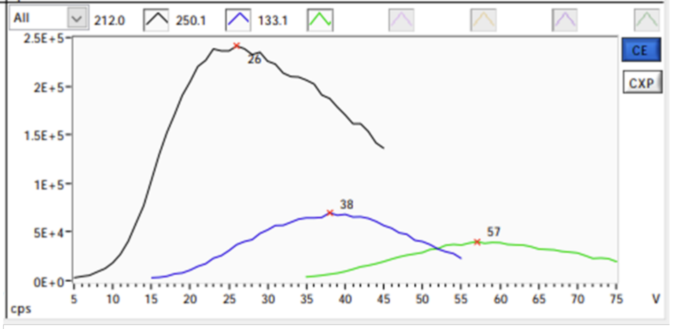

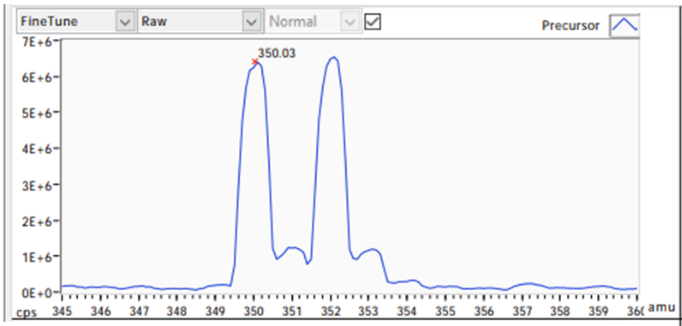

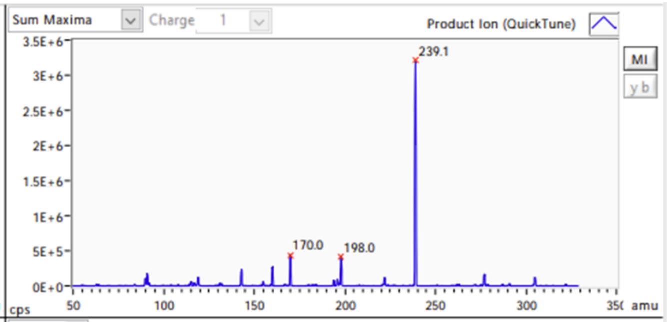

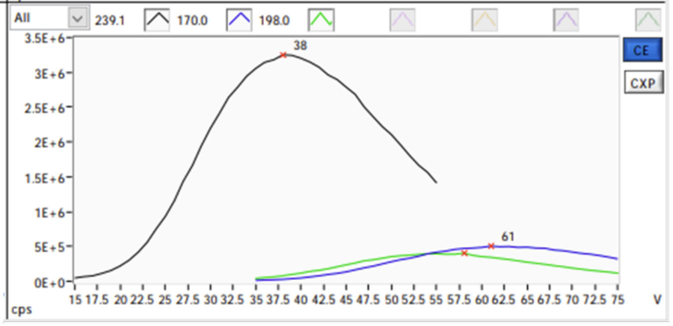

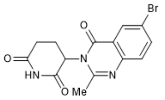

11

FineTune +

	Ion	Intensity	DP	EP	CE	CXP
Q1	350.029	6.3991E+6	50	10	-	-
Q3	239.054	3.2502E+6	50	10	38	16
Q3	170.039	5.0480E+5	50	10	61	10
Q3	198.026	4.0489E+5	50	10	58	12
Q3	-	-	-	-	-	-
Q3	-	-	-	-	-	-
Q3	-	-	-	-	-	-
Q3	-	-	-	-	-	-

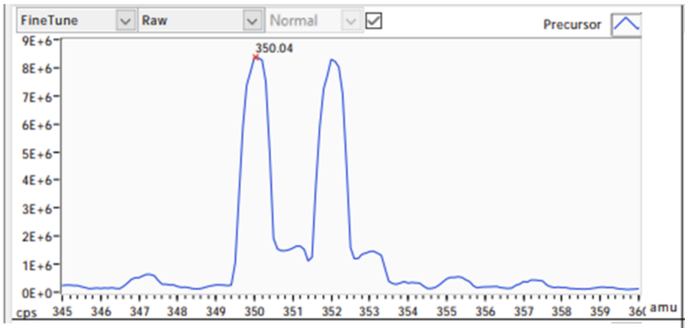

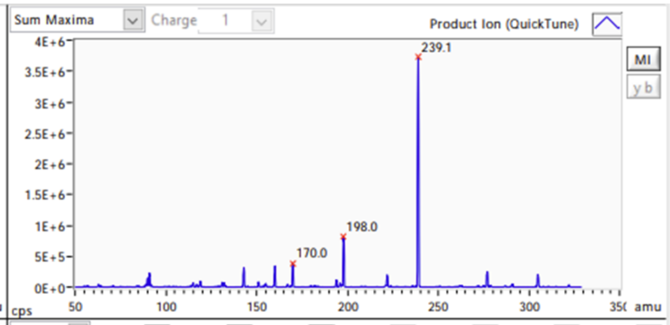

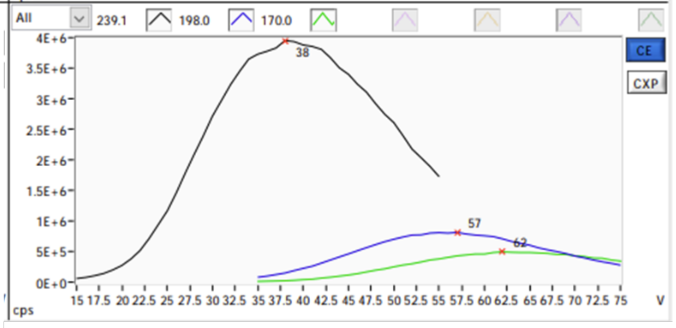

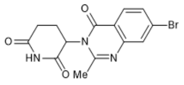

12

FineTune +

	Ion	Intensity	DP	EP	CE	CXP
Q1	350.040	8.3573E+6	50	10	-	-
Q3	239.068	3.9509E+6	50	10	38	13
Q3	198.014	8.1111E+5	50	10	57	11
Q3	170.026	4.9639E+5	50	10	62	10
Q3	-	-	-	-	-	-
Q3	-	-	-	-	-	-
Q3	-	-	-	-	-	-
Q3	-	-	-	-	-	-

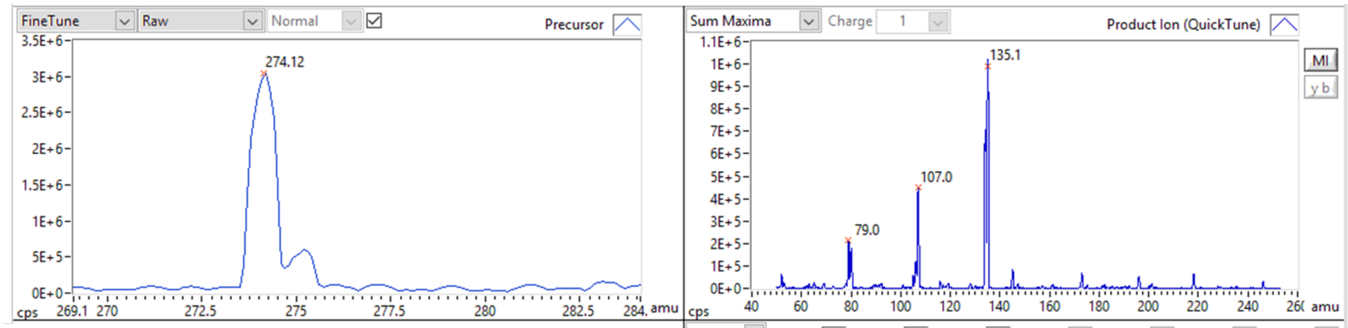

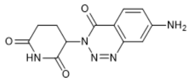

13

	Ion	Intensity	DP	EP	CE	CXP
Q1	274.120	3.0466E+6	100	10	-	-
Q3	135.107	8.3290E+5	100	10	27	14
Q3	107.046	3.4268E+5	100	10	47	13
Q3	79.031	2.1630E+5	100	10	65	9
Q3	-	-	-	-	-	-
Q3	-	-	-	-	-	-
Q3	-	-	-	-	-	-
Q3	-	-	-	-	-	-

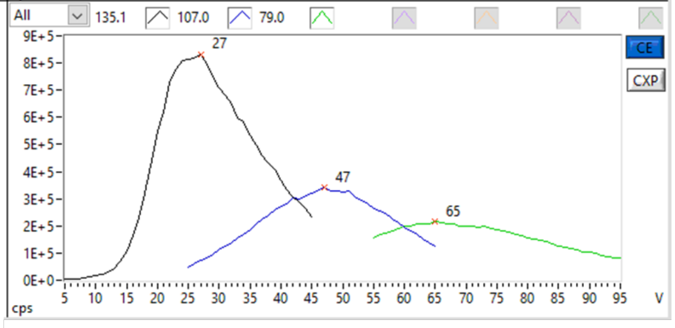

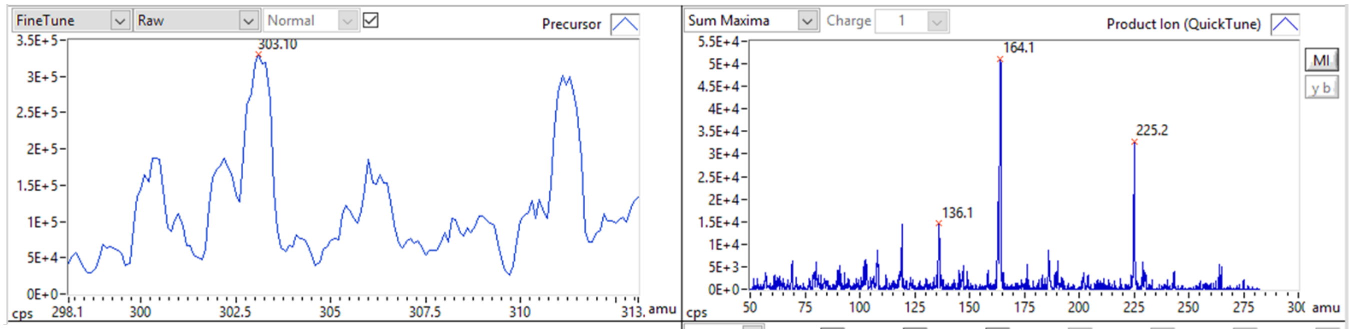

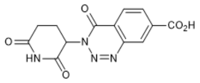

14

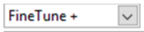

	Ion	Intensity	DP	EP	CE	CXP
Q1	303.100	3.2989E+5	110	10	-	-
Q3	164.114	3.7364E+4	110	10	27	11
Q3	136.108	1.0813E+4	110	10	46	15
Q3	-	-	-	-	-	-
Q3	-	-	-	-	-	-
Q3	-	-	-	-	-	-
Q3	-	-	-	-	-	-
Q3	-	-	-	-	-	-

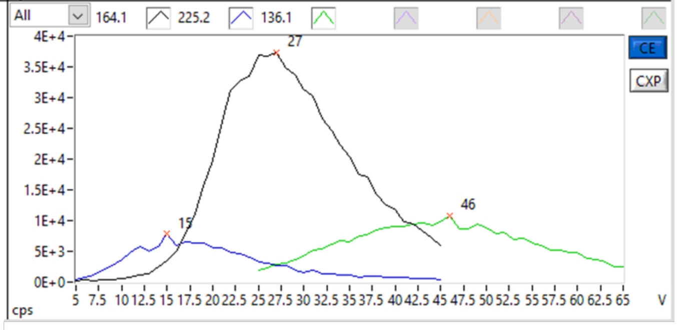

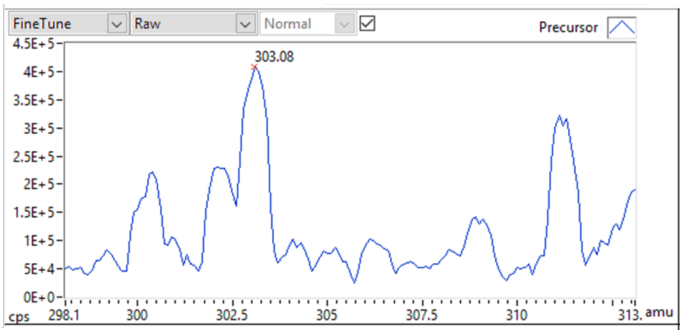

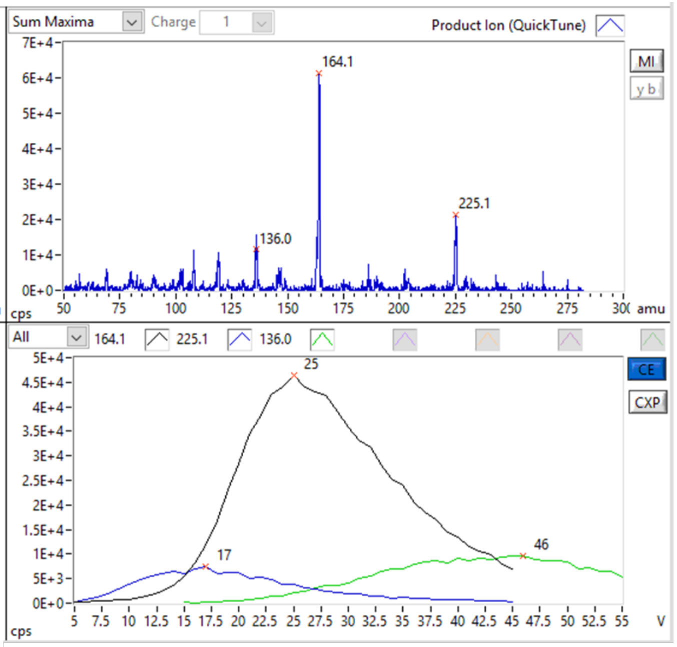

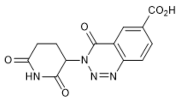

15

FineTune +

	Ion	Intensity	DP	EP	CE	CXP
Q1	303.084	4.0767E+5	100	10	-	-
Q3	164.102	4.6482E+4	100	10	25	10
Q3	-	-	-	-	-	-
Q3	-	-	-	-	-	-
Q3	-	-	-	-	-	-
Q3	-	-	-	-	-	-
Q3	-	-	-	-	-	-
Q3	-	-	-	-	-	-

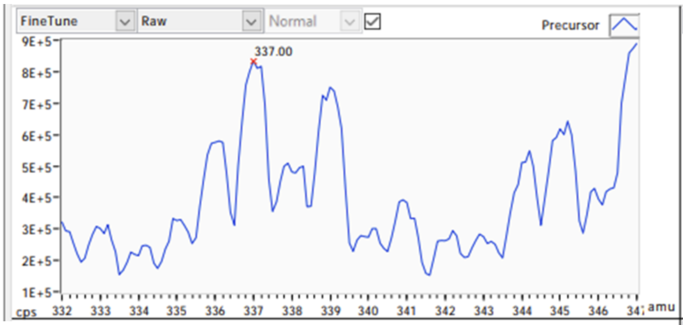

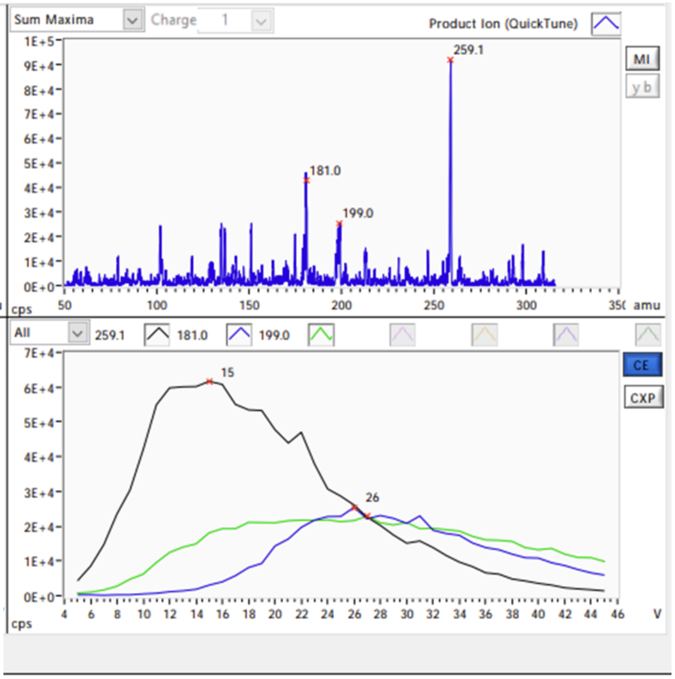

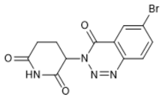

16

FineTune +

	Ion	Intensity	DP	EP	CE	CXP
Q1	337.004	8.3444E+5	60	10	-	-
Q3	259.061	6.1608E+4	60	10	15	18
Q3	180.988	2.5386E+4	60	10	26	10
Q3	198.999	2.2887E+4	60	10	27	14
Q3	-	-	-	-	-	-
Q3	-	-	-	-	-	-
Q3	-	-	-	-	-	-
Q3	-	-	-	-	-	-

4. References

[1] M. Hu, L. Yang, N. Twarog, J. Ochoada, Y. Li, E. I. Vrettos, A. X. Torres-Hernandez, J. B. Martinez, J. Bhatia, B. M. Young, J. Price, K. McGowan, T. H. Nguyen, Z. Shi, M. Anyanwu, M. A. Rimmer, S. Mercer, Z. Rankovic, A. A. Shelat, D. J. Blair, Continuous collective analysis of chemical reactions, *Nature* **636**, 374–379, (2024).

[2] D. A. Annis, J. Athanasopoulos, P. J. Curran, J. S. Felsch, K. Kalghatgi, W. H. Lee, H. M. Nash, J.-P. A. Orminati, K. E. Rosner, G. W. Shipps Jr., G. R. A. Thaddupathy, A. N. Tyler, L. Vilenchik, C. R. Wagner, E. A. Wintner, An agginity selection-mass spectrometry method for the identification of small molecule ligands from self-encoded combinatorial libraries: Discovery of a novel antagonist of E. coli dihydrofolate reductase, *Int. J. Mass. Spec.* **238**, 77–83, (2004).

[3] M. E. Matyskiela, G. Lu, T. Ito, B. Pagarigan, C.-C. Lu, K. Willer, W. Fang, N.-Y. Wang, D. Nguyen, J. Houston, G. Carmel, T. Tran, M. Riley, L. Nosaka, G. C. Lander, S. Gaidarova, S. Xu, A. L. Ruchelman, H. Handa, J. Carmichael, T. O. Daniel, B. E. Cathers, A. Lopez-Girona, P. P. Chamberlain, A novel cereblon modulator recruits GSPT1 to the CRL4CRBN ubiquitin ligase. *Nature* **535**, 252–257 (2016).

[4] J.M. Ostrem, U. Peters, M.L. Sos, J.A. Wells, K.M. Shokat, K-Ras(G12C) inhibitors allosterically control GTP affinity and effector interactions, *Nature*, **503**, 548–551 (2013).

[5] R. C. Killoran, M. J. Smith, Conformational resolution of nucleotide cycling and effector interactions for multiple small GTPases determined in parallel, *Journal of Biological Chemistry*, **294**, 9937–9948 (2019).

5. NMR Spectra

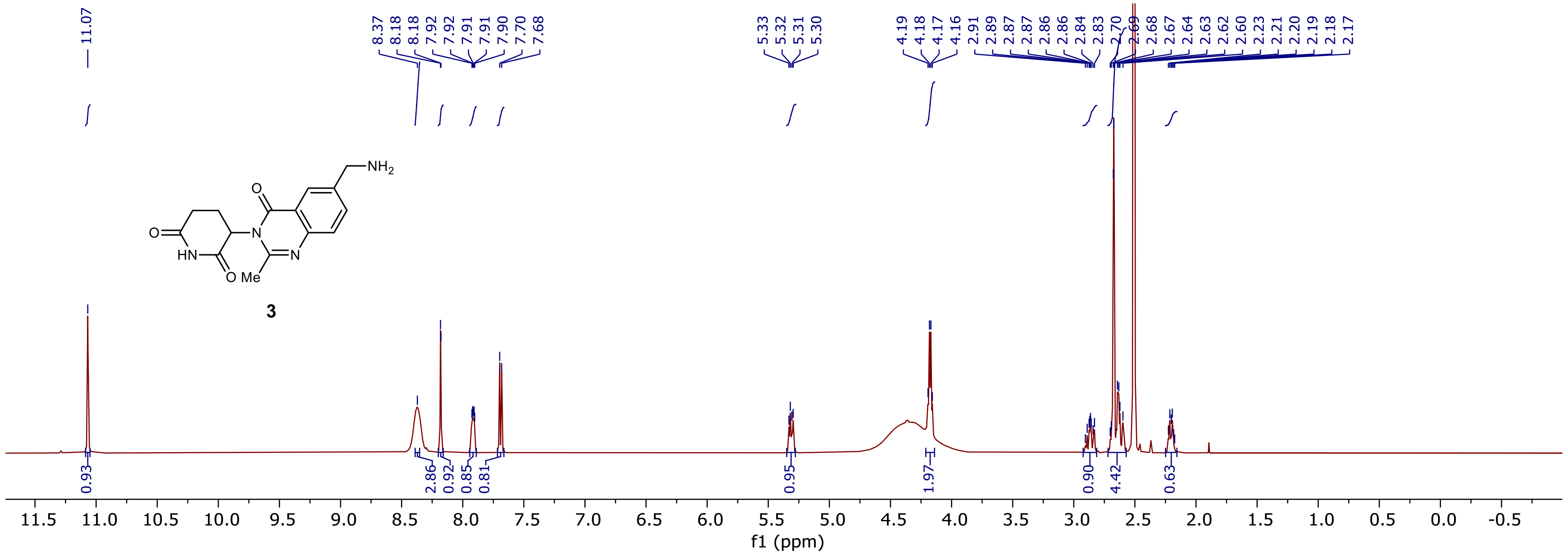
11.07
8.37 8.18 8.18 7.92 7.92 7.91 7.91 7.90 7.70 7.68
5.33 5.32 5.31 5.30
4.19 4.18 4.17 4.16 2.91 2.89 2.87 2.87 2.86 2.86 2.84 2.83 2.70 2.69 2.68 2.67 2.64 2.63 2.62 2.60 2.23 2.21 2.20 2.19 2.18 2.17
NH2
O
O
HN
O
N
N
Me
3
0.93
2.86 0.92 0.85 0.81
0.95
1.97
0.90 4.42 0.63
f1 (ppm)

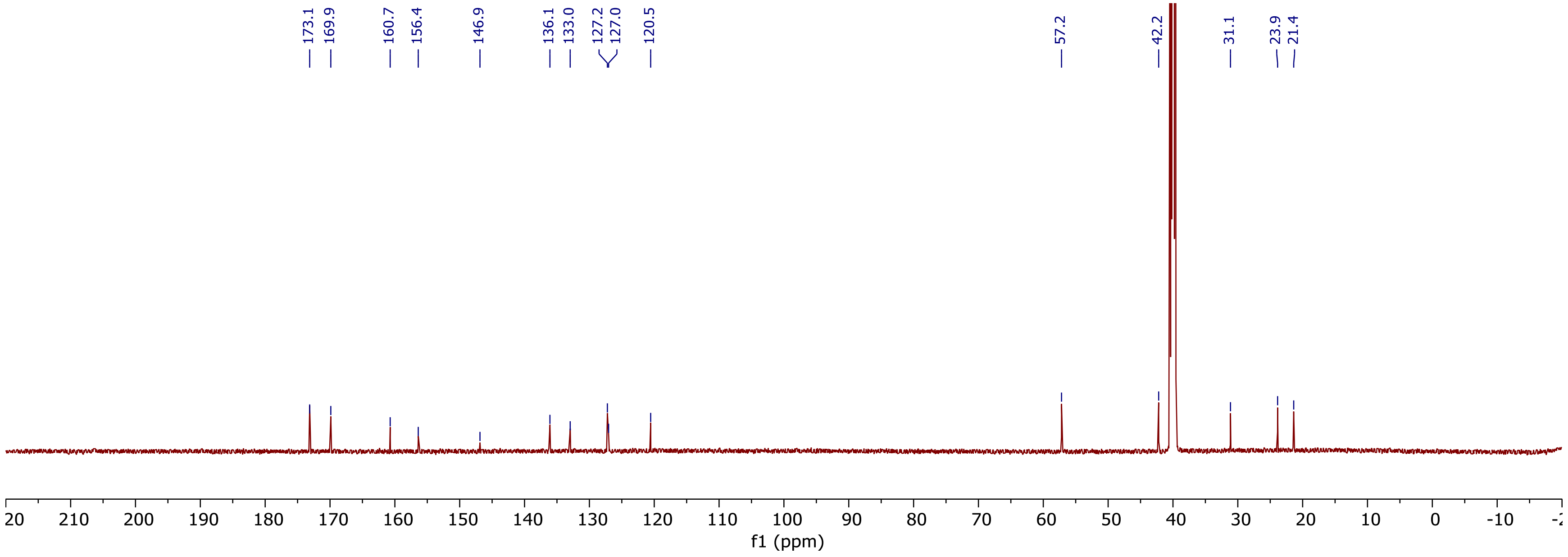
173.1 169.9 160.7 156.4 146.9 136.1 133.0 127.2 127.0 120.5
57.2 42.2 31.1 23.9 21.4
f1 (ppm)

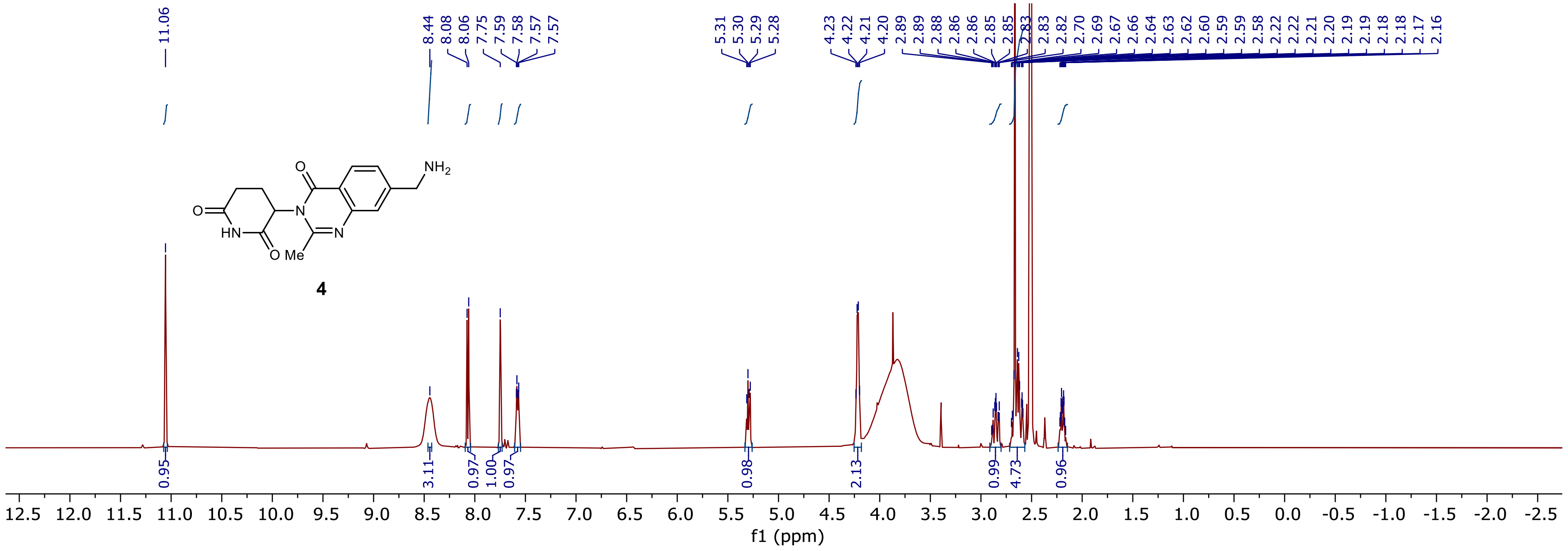
11.06
8.44
8.08
8.06
7.75
7.59
7.58
7.57
7.57
5.31
5.30
5.29
5.28
4.23
4.22
4.21
4.20
2.89
2.88
2.86
2.86
2.85
2.85
2.83
2.83
2.82
2.70
2.69
2.67
2.66
2.64
2.63
2.62
2.60
2.59
2.59
2.58
2.22
2.22
2.21
2.20
2.19
2.19
2.18
2.18
2.17
2.16
O
NH2
O
HN
N
N
O
Me
4
0.95
3.11
0.97
1.00
0.97
0.98
2.13
0.99
4.73
0.96
f1 (ppm)

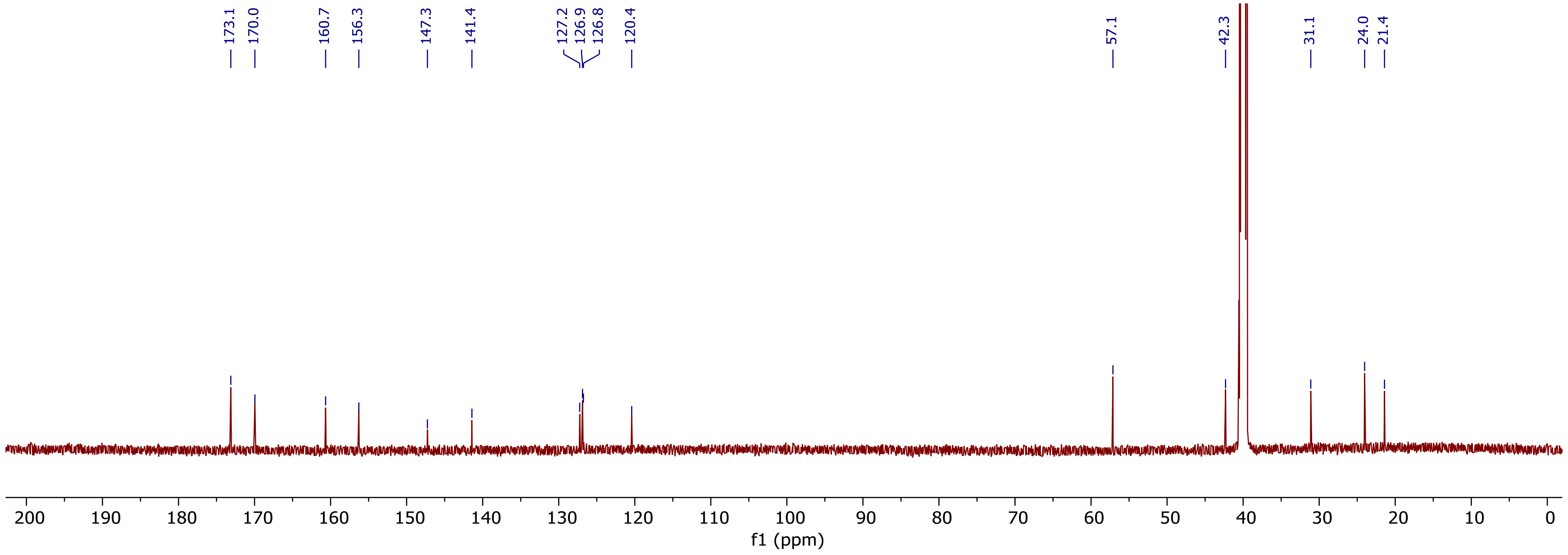
173.1
170.0
160.7
156.3
147.3
141.4
127.2
126.9
126.8
120.4
57.1
42.3
31.1
24.0
21.4
f1 (ppm)

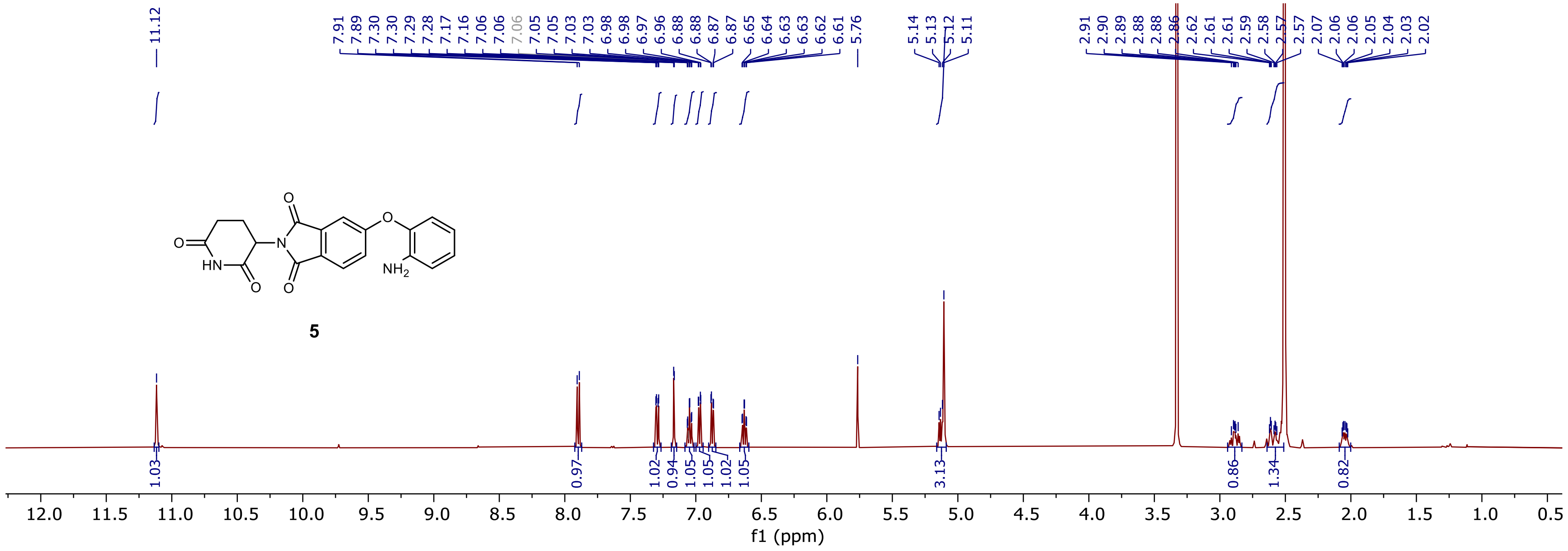
11.12
7.91 7.89 7.30 7.30 7.29 7.28 7.17 7.16 7.06 7.06 7.06 7.05 7.05 7.03 7.03 6.98 6.98 6.97 6.96 6.88 6.88 6.87 6.87 6.65 6.64 6.63 6.63 6.62 6.61 5.76
5.14 5.13 5.12 5.11
2.91 2.90 2.89 2.88 2.88 2.86 2.62 2.61 2.61 2.59 2.58 2.57 2.57 2.07 2.06 2.06 2.05 2.04 2.03 2.02
O
HN
N
NH2
5
1.03 0.97 1.02 0.94 1.05 1.05 1.02 1.05 3.13 0.86 1.34 0.82
12.0 11.5 11.0 10.5 10.0 9.5 9.0 8.5 8.0 7.5 7.0 6.5 6.0 5.5 5.0 4.5 4.0 3.5 3.0 2.5 2.0 1.5 1.0 0.5
f1 (ppm)

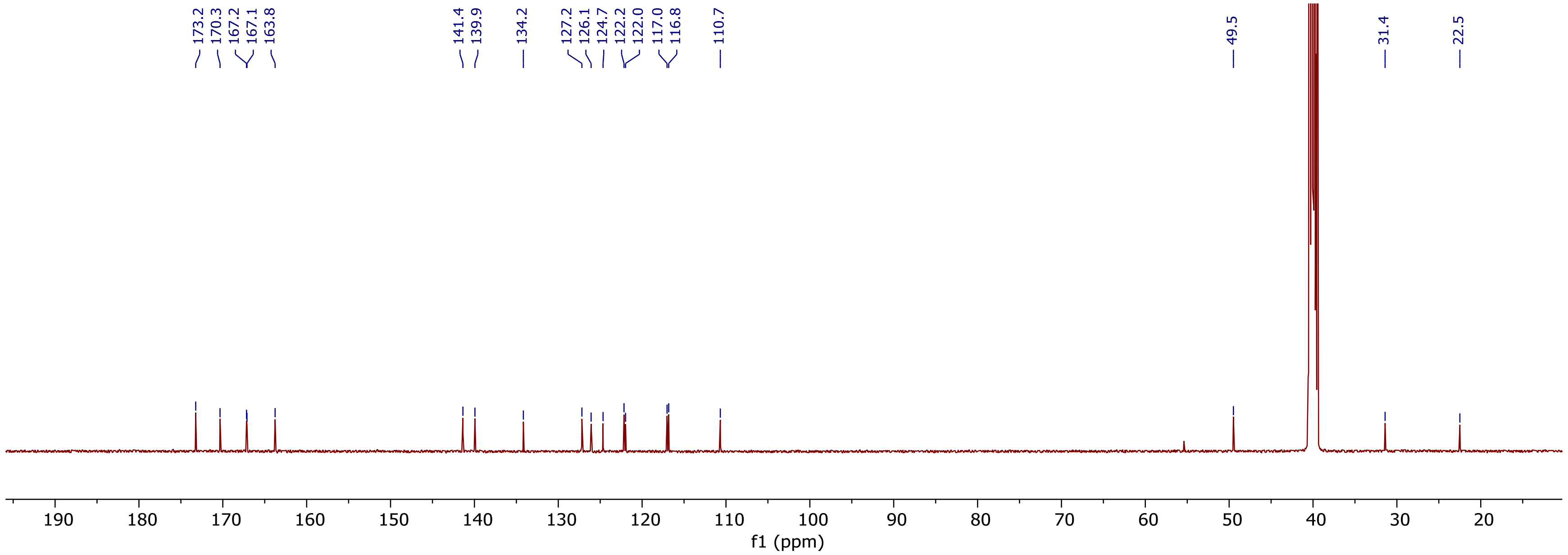
173.2 170.3 167.2 167.1 163.8 141.4 139.9 134.2 127.2 126.1 124.7 122.2 122.0 117.0 116.8 110.7 49.5 31.4 22.5
190 180 170 160 150 140 130 120 110 100 90 80 70 60 50 40 30 20
f1 (ppm)

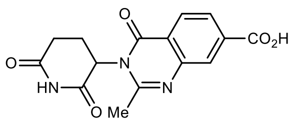
O
O
N
CO2H
HN
N
O Me

7

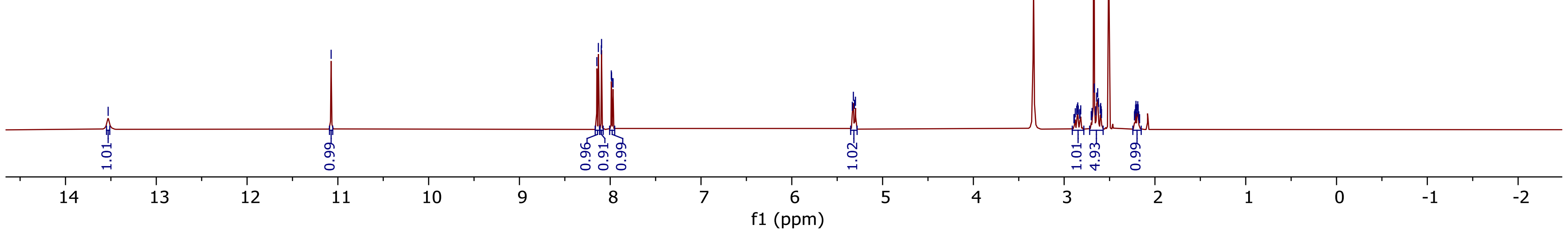
13.53
11.07
8.15
8.13
8.10
8.10
7.99
7.99
7.97
7.97
5.33
5.32
5.31
5.30
2.89
2.89
2.88
2.86
2.86
2.85
2.84
2.83
2.83
2.82
2.70
2.69
2.67
2.67
2.65
2.64
2.63
2.62
2.60
2.60
2.59
2.59
2.23
2.22
2.21
2.21
2.20
2.20
2.19
2.19
2.18
2.17
1.01
0.99
0.96
0.91
0.99
1.02
1.01
4.93
0.99
f1 (ppm)

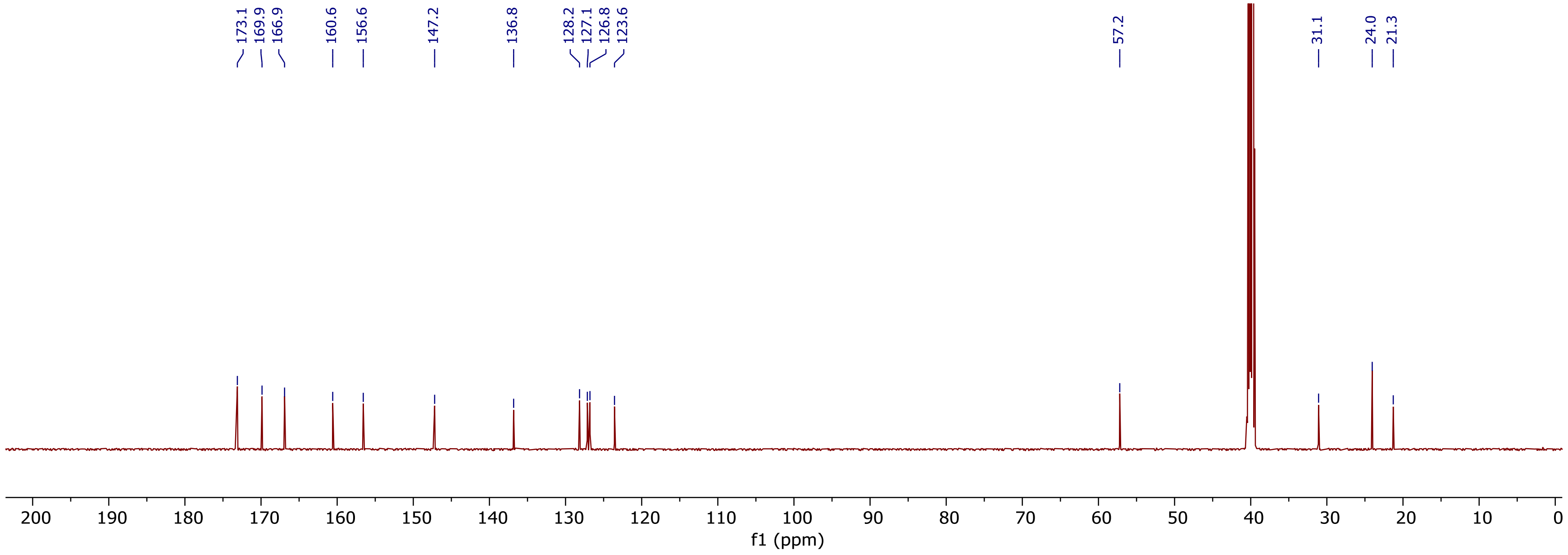
173.1
169.9
166.9
160.6
156.6
147.2
136.8
128.2
127.1
126.8
123.6
57.2
31.1
24.0
21.3
f1 (ppm)

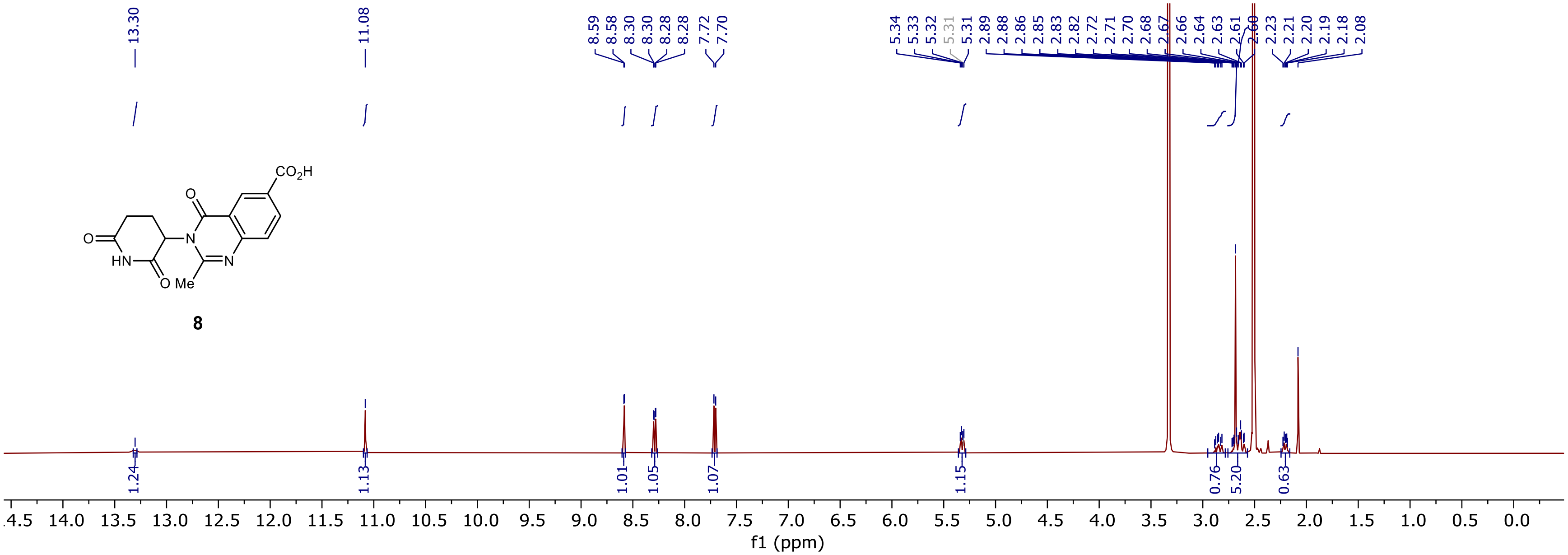
CO2H
O
O
N
HN
O
N
O Me
8
13.30
11.08
8.59 8.58 8.30 8.30 8.28 8.28 7.72 7.70
5.34 5.33 5.32 5.31 5.31 2.89 2.88 2.86 2.85 2.83 2.82 2.72 2.71 2.70 2.68 2.67 2.66 2.64 2.63 2.61 2.60 2.23 2.21 2.20 2.19 2.18 2.08
1.24
1.13
1.01
1.05
1.07
1.15
0.76
5.20
0.63
14.5 14.0 13.5 13.0 12.5 12.0 11.5 11.0 10.5 10.0 9.5 9.0 8.5 8.0 7.5 7.0 6.5 6.0 5.5 5.0 4.5 4.0 3.5 3.0 2.5 2.0 1.5 1.0 0.5 0.0
f1 (ppm)

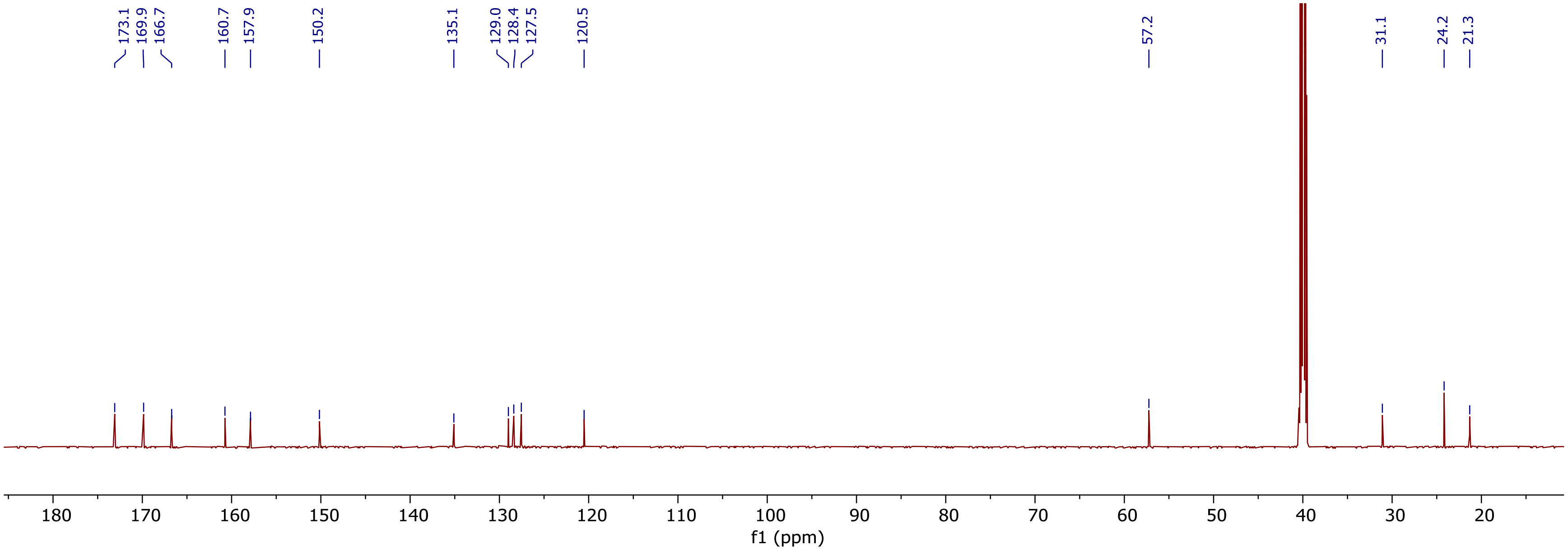
173.1 169.9 166.7
160.7 157.9
150.2
135.1
129.0 128.4 127.5
120.5
57.2
31.1
24.2 21.3
180 170 160 150 140 130 120 110 100 90 80 70 60 50 40 30 20
f1 (ppm)

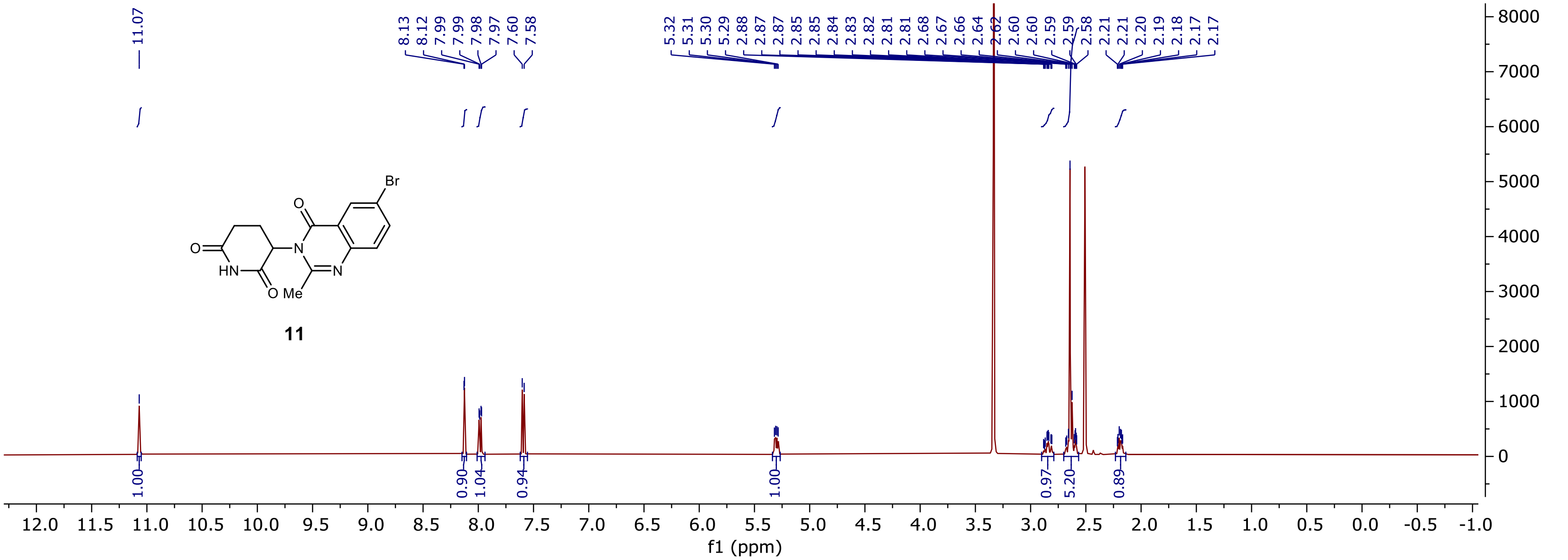
11.07
8.13 8.12 7.99 7.99 7.98 7.97 7.60 7.58
5.32 5.31 5.30 5.29 2.88 2.87 2.87 2.85 2.85 2.84 2.83 2.82 2.81 2.81 2.68 2.67 2.66 2.64 2.62 2.60 2.60 2.59 2.59 2.58 2.21 2.21 2.20 2.19 2.18 2.17 2.17
Br
O
O
HN
N
O
N
Me
11
1.00
0.90
1.04
0.94
1.00
0.97
5.20
0.89
f1 (ppm)

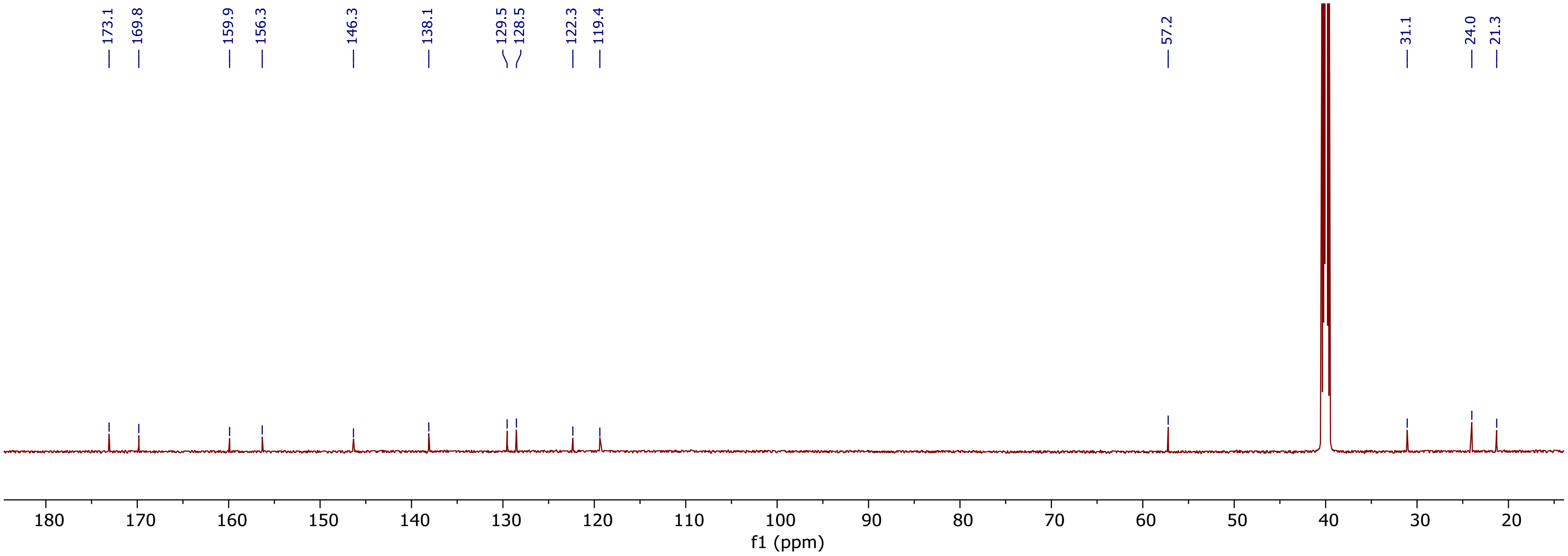
173.1
169.8
159.9
156.3
146.3
138.1
129.5
128.5
122.3
119.4
57.2
31.1
24.0
21.3
f1 (ppm)

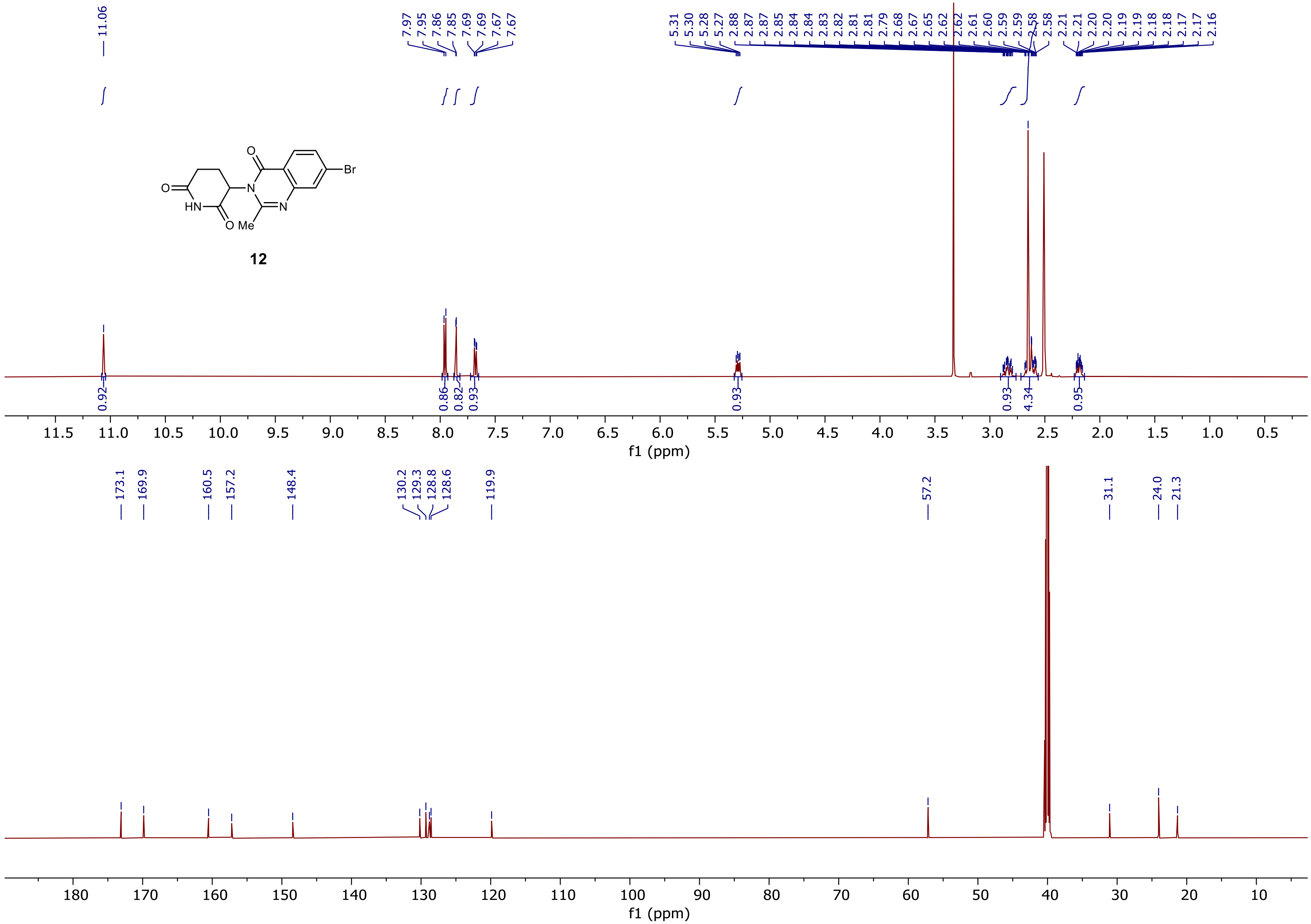
O
HN
O
N
N
Me
O
Br
11.06
7.97 7.95 7.86 7.85 7.69 7.69 7.67 7.67
5.31 5.30 5.28 5.27 2.88 2.87 2.87 2.85 2.84 2.84 2.83 2.82 2.81 2.81 2.79 2.68 2.67 2.65 2.62 2.62 2.61 2.60 2.59 2.59 2.58 2.58 2.21 2.21 2.20 2.20 2.19 2.19 2.18 2.18 2.17 2.17 2.16
0.92 0.86 0.82 0.93 0.93 0.93 4.34 0.95
11.5 11.0 10.5 10.0 9.5 9.0 8.5 8.0 7.5 7.0 6.5 6.0 5.5 5.0 4.5 4.0 3.5 3.0 2.5 2.0 1.5 1.0 0.5
f1 (ppm)
173.1 169.9 160.5 157.2 148.4 130.2 129.3 128.8 128.6 119.9 57.2 31.1 24.0 21.3
180 170 160 150 140 130 120 110 100 90 80 70 60 50 40 30 20 10
f1 (ppm)

12

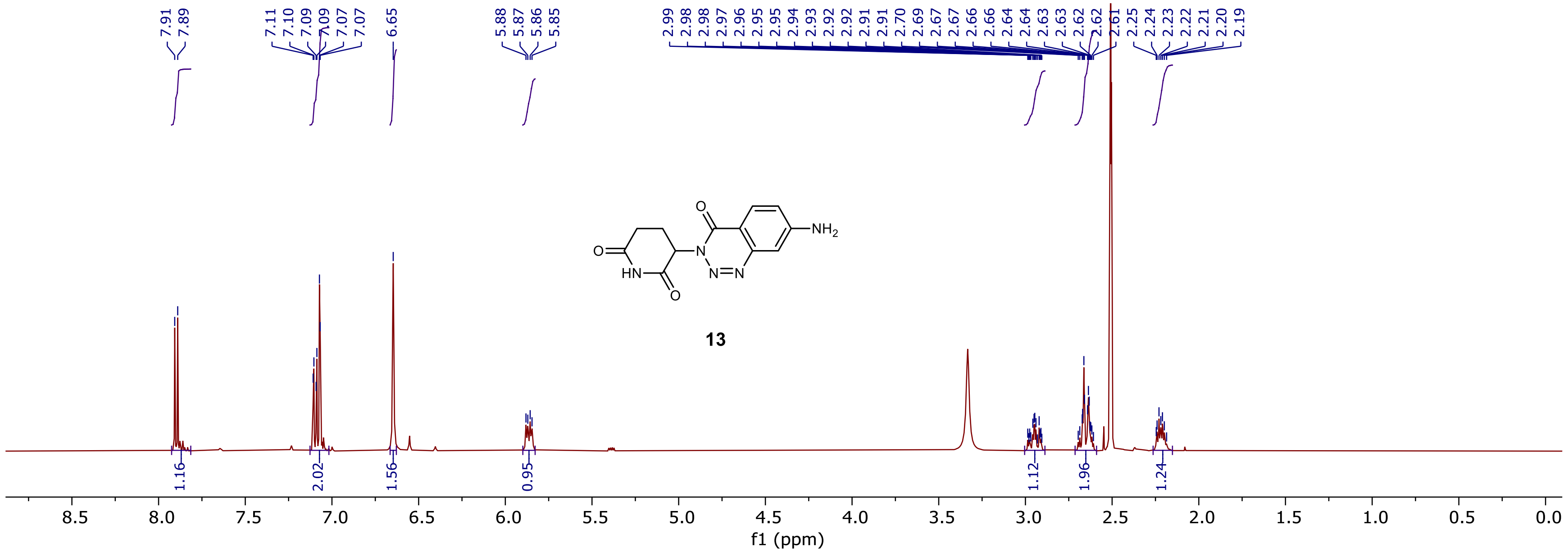
7.91
7.89
7.11
7.10
7.09
7.09
7.07
7.07
6.65
5.88
5.87
5.86
5.85
2.99
2.98
2.98
2.97
2.96
2.95
2.95
2.94
2.93
2.92
2.92
2.91
2.91
2.70
2.69
2.67
2.67
2.66
2.66
2.64
2.64
2.63
2.63
2.62
2.62
2.61
2.25
2.24
2.23
2.22
2.21
2.20
2.19
O
NH2
O
N
HN
N=N
O
13
1.16
2.02
1.56
0.95
1.12
1.96
1.24
8.5 8.0 7.5 7.0 6.5 6.0 5.5 5.0 4.5 4.0 3.5 3.0 2.5 2.0 1.5 1.0 0.5 0.0
f1 (ppm)
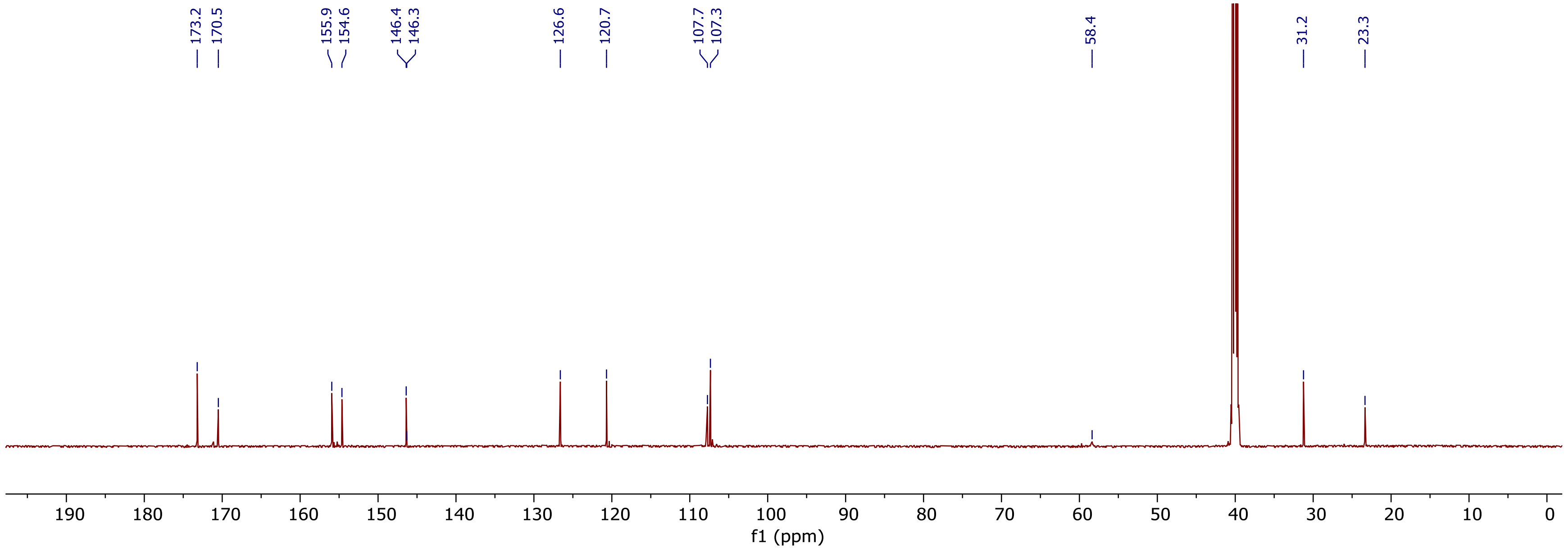
173.2
170.5
155.9
154.6
146.4
146.3
126.6
120.7
107.7
107.3
58.4
31.2
23.3
190 180 170 160 150 140 130 120 110 100 90 80 70 60 50 40 30 20 10 0
f1 (ppm)

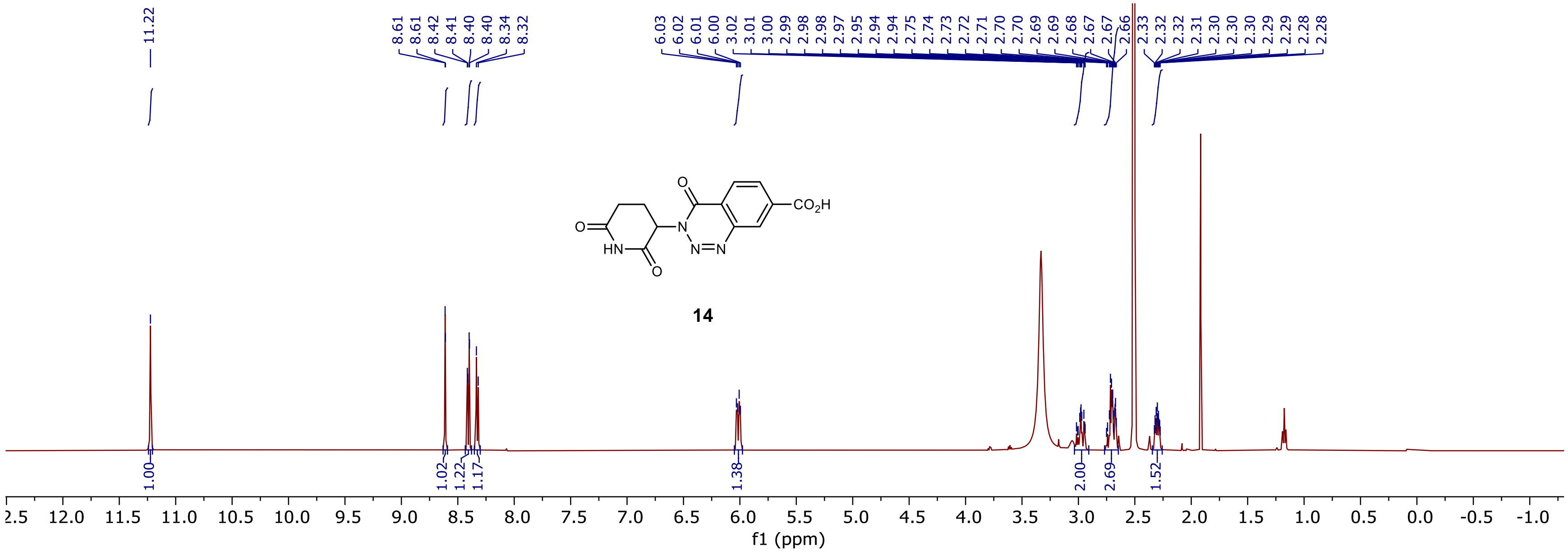
11.22
8.61 8.61 8.42 8.41 8.40 8.40 8.34 8.32
6.03 6.02 6.01 6.00 3.02 3.01 3.00 2.99 2.98 2.98 2.97 2.95 2.94 2.94 2.75 2.74 2.73 2.72 2.71 2.70 2.69 2.69 2.68 2.67 2.67 2.66
2.33 2.32 2.32 2.31 2.30 2.30 2.30 2.29 2.29 2.28 2.28
O
CO2H
O
N
HN
N
N
O
O
14
1.00
1.02
1.22
1.17
1.38
2.00
2.69
1.52
f1 (ppm)

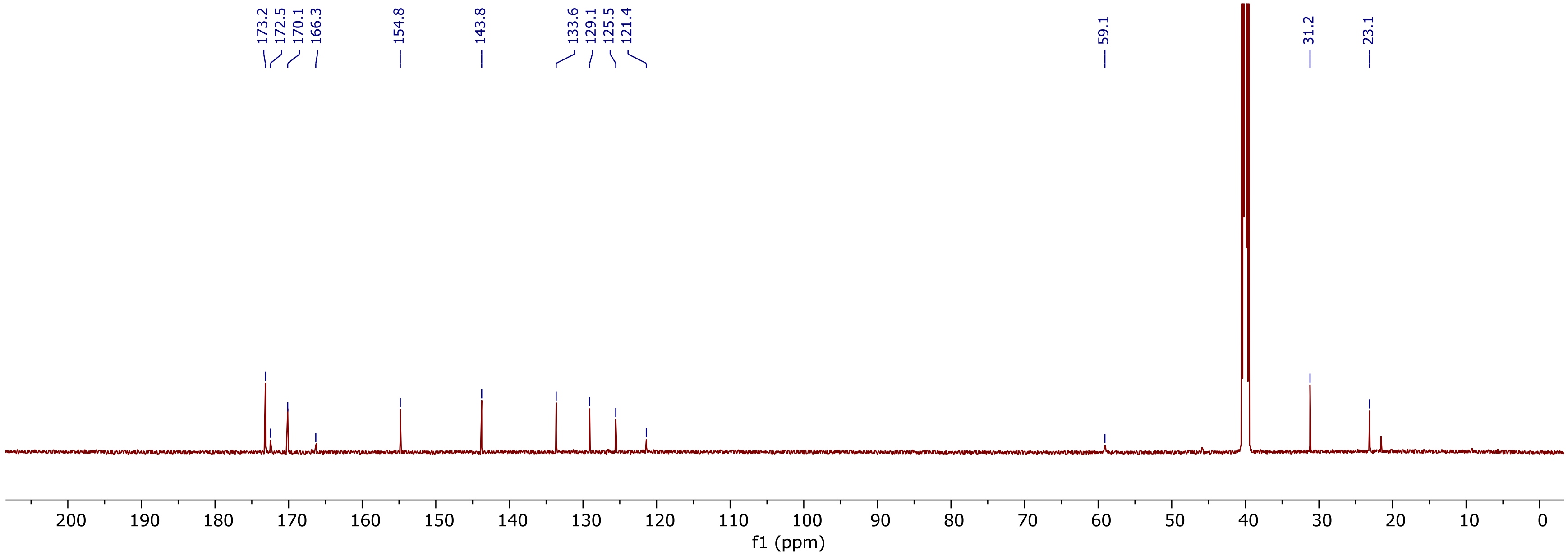
173.2 172.5 170.1 166.3
154.8
143.8
133.6 129.1 125.5 121.4
59.1
31.2
23.1
f1 (ppm)

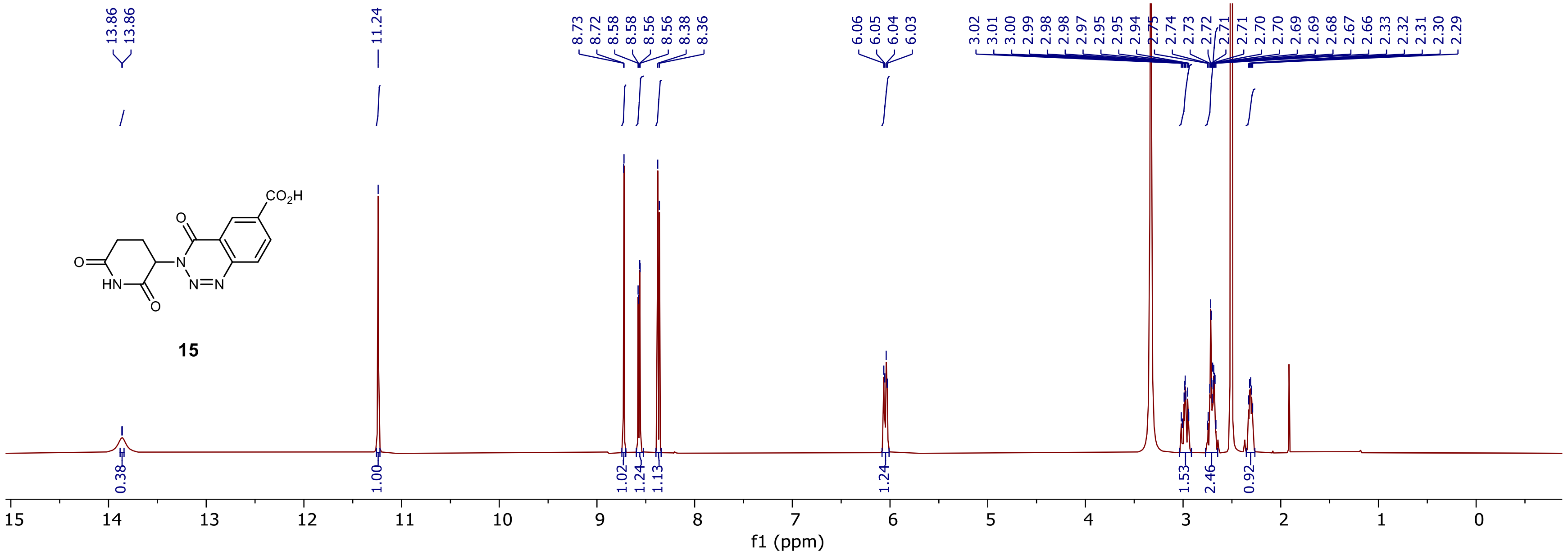
13.86
13.86
11.24
8.73
8.72
8.58
8.58
8.56
8.56
8.38
8.36
6.06
6.05
6.04
6.03
3.02
3.01
3.00
2.99
2.98
2.98
2.97
2.95
2.95
2.94
2.75
2.74
2.73
2.72
2.71
2.71
2.70
2.70
2.69
2.69
2.68
2.67
2.66
2.33
2.32
2.31
2.30
2.29
CO2H
O
O
N
HN
N=N
O
15
0.38
1.00
1.02
1.24
1.13
1.24
1.53
2.46
0.92
15
14
13
12
11
10
9
8
7
6
5
4
3
2
1
0
f1 (ppm)
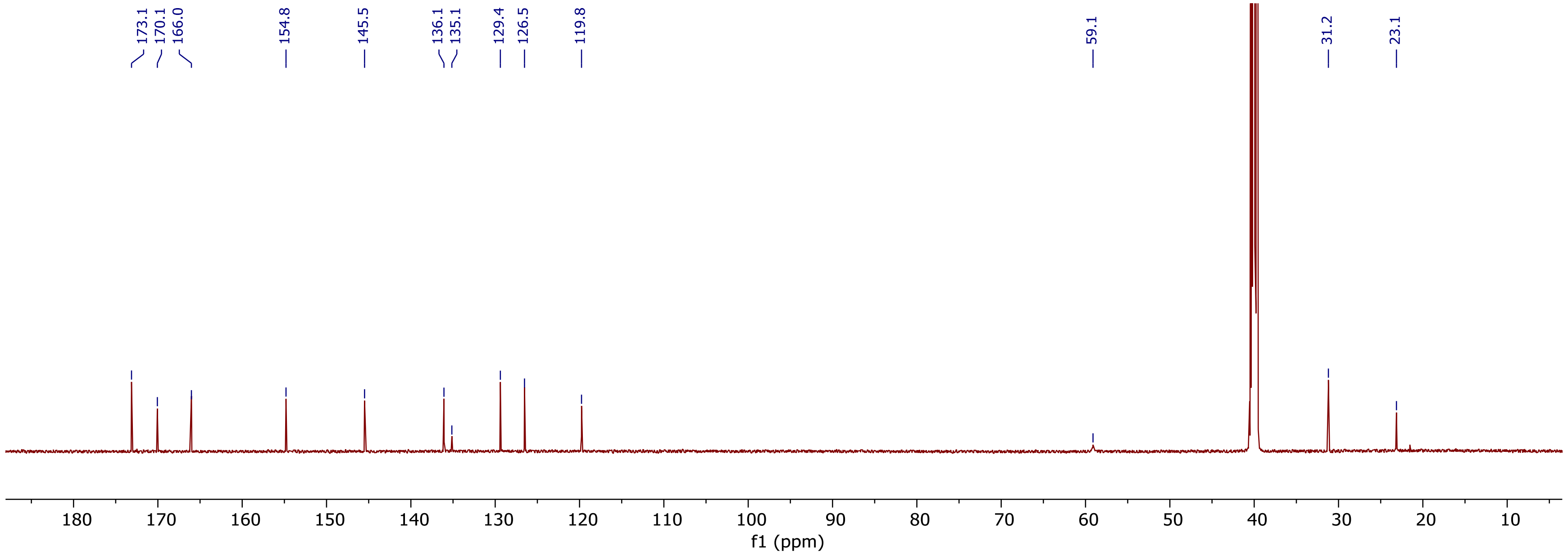
173.1
170.1
166.0
154.8
145.5
136.1
135.1
129.4
126.5
119.8
59.1
31.2
23.1
180
170
160
150
140
130
120
110
100
90
80
70
60
50
40
30
20
10
f1 (ppm)

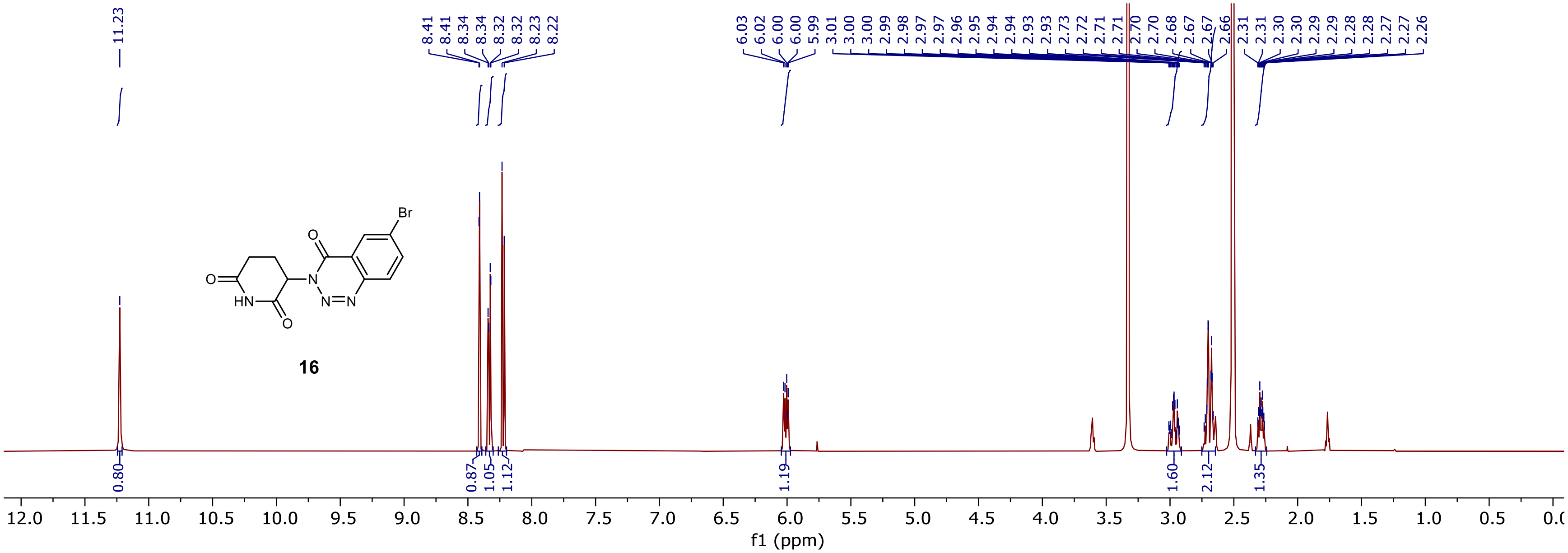
11.23
8.41
8.41
8.34
8.34
8.32
8.32
8.23
8.22
6.03
6.02
6.00
6.00
5.99
3.01
3.00
3.00
2.99
2.98
2.97
2.97
2.96
2.95
2.94
2.94
2.93
2.93
2.73
2.72
2.71
2.71
2.70
2.70
2.68
2.67
2.67
2.66
2.31
2.31
2.30
2.30
2.29
2.29
2.28
2.28
2.27
2.27
2.26
Br
O
O
N
HN
N=N
O
16
0.80
0.87
1.05
1.12
1.19
1.60
2.12
1.35
f1 (ppm)

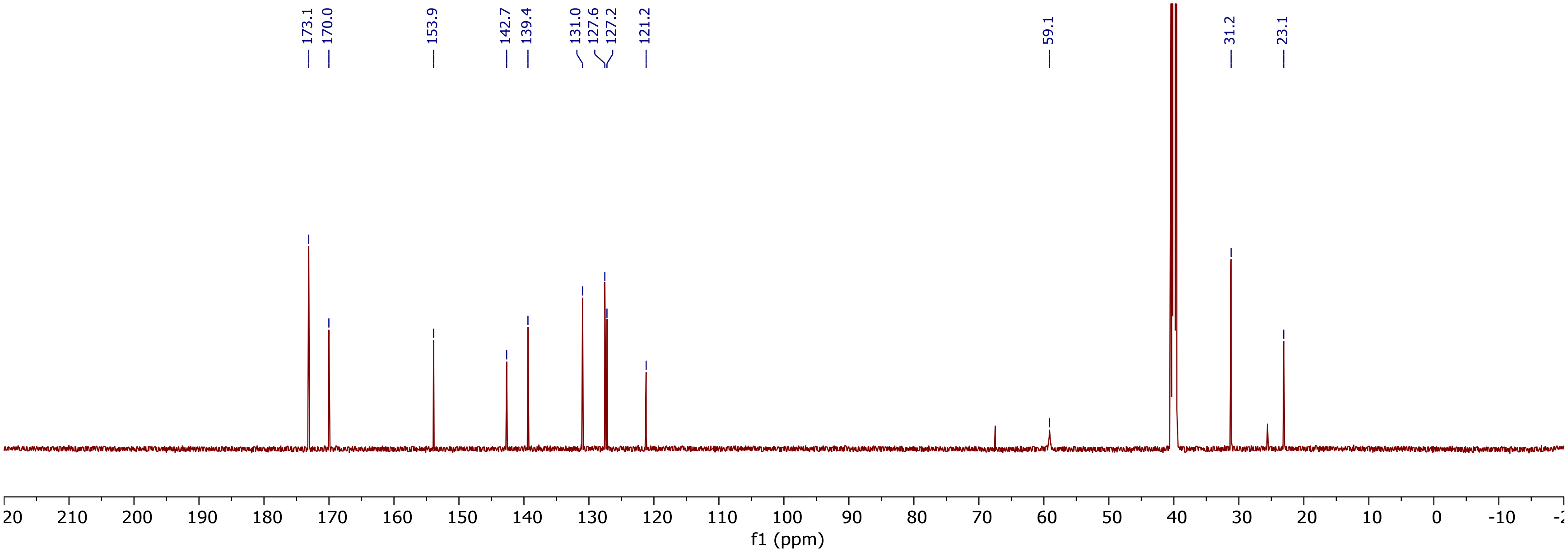
173.1
170.0
153.9
142.7
139.4
131.0
127.6
127.2
121.2
59.1
31.2
23.1
f1 (ppm)

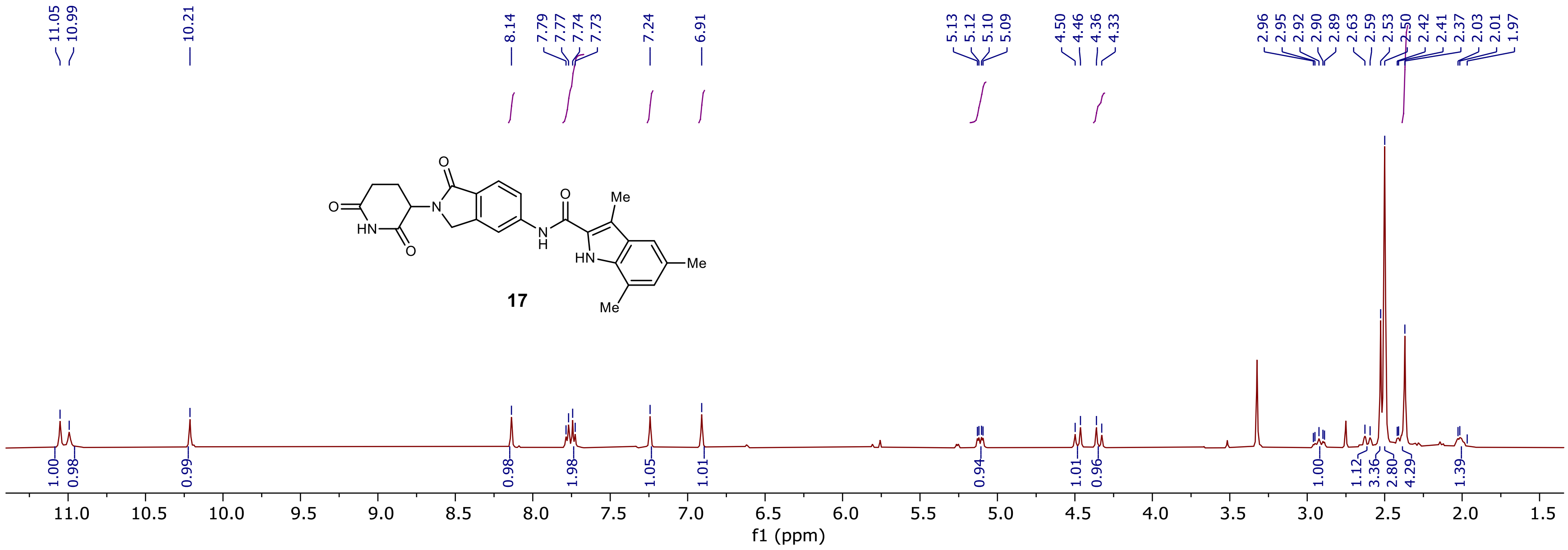
11.05
10.99
10.21
8.14
7.79
7.77
7.74
7.73
7.24
6.91
5.13
5.12
5.10
5.09
4.50
4.46
4.36
4.33
2.96
2.95
2.92
2.90
2.89
2.63
2.59
2.53
2.50
2.42
2.41
2.37
2.03
2.01
1.97
O
O
HN
N
O
N
H
O
Me
HN
Me
Me
17
1.00
0.98
0.99
0.98
1.98
1.05
1.01
0.94
1.01
0.96
1.00
1.12
3.36
2.80
4.29
1.39
11.0
10.5
10.0
9.5
9.0
8.5
8.0
7.5
7.0
6.5
6.0
5.5
5.0
4.5
4.0
3.5
3.0
2.5
2.0
1.5
f1 (ppm)

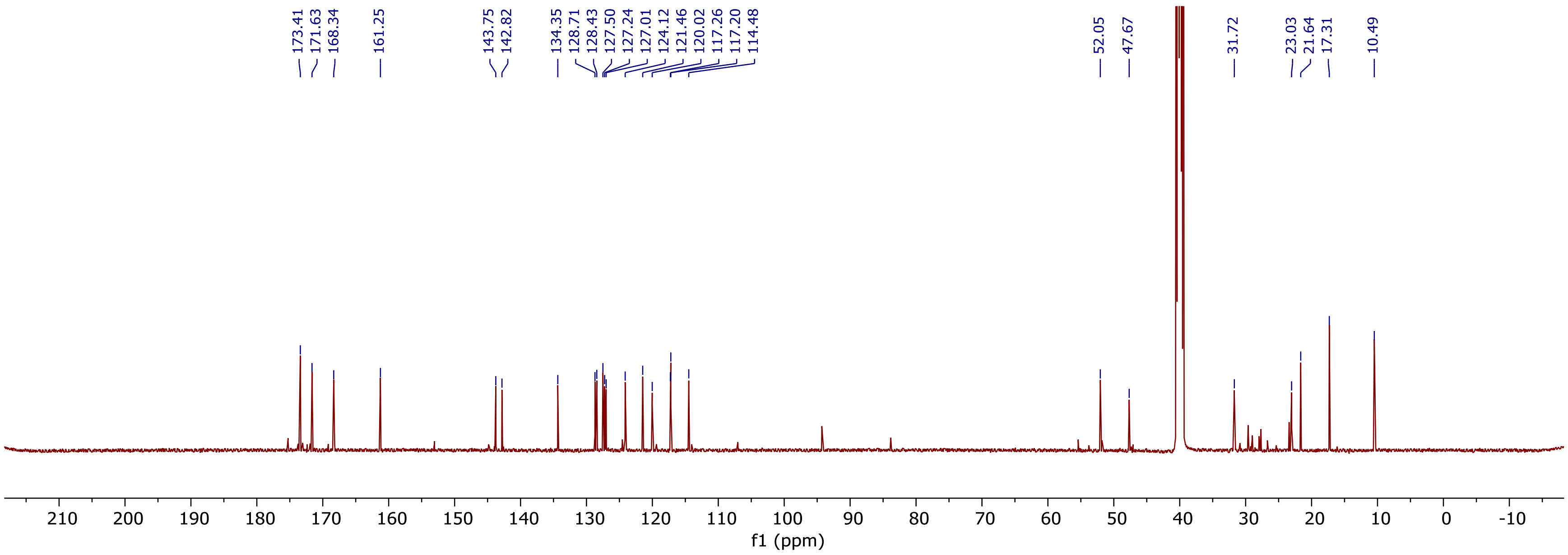
173.41
171.63
168.34
161.25
143.75
142.82
134.35
128.71
128.43
127.50
127.24
127.01
124.12
121.46
120.02
117.26
117.20
114.48
52.05
47.67
31.72
23.03
21.64
17.31
10.49
210
200
190
180
170
160
150
140
130
120
110
100
90
80
70
60
50
40
30
20
10
0
-10
f1 (ppm)

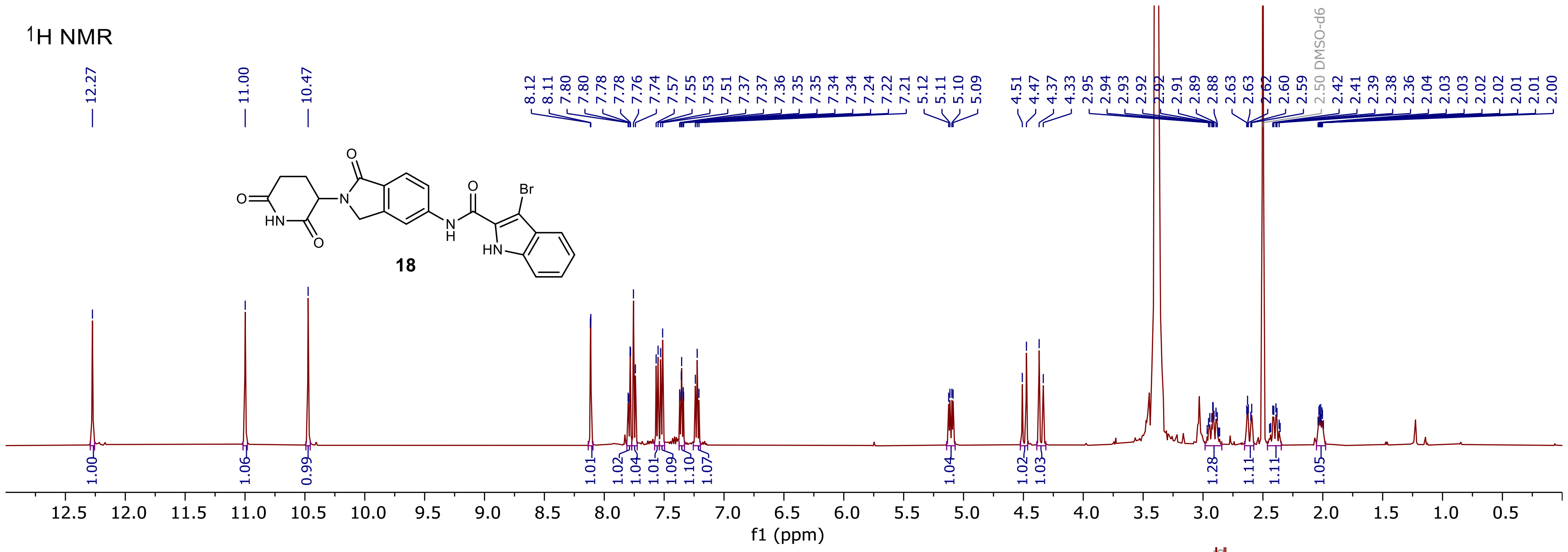
1H NMR
12.27
11.00
10.47
8.12 8.11 7.80 7.80 7.78 7.78 7.76 7.74 7.57 7.55 7.53 7.51 7.37 7.37 7.36 7.35 7.35 7.34 7.24 7.22 7.21 5.12 5.11 5.10 5.09 4.51 4.47 4.37 4.33 2.95 2.94 2.93 2.92 2.92 2.91 2.89 2.88 2.63 2.63 2.62 2.60 2.59
2.50 DMSO-d6
2.42 2.41 2.39 2.38 2.36 2.04 2.03 2.02 2.02 2.01 2.01 2.00
O
O
HN
O
N
O
N
H
Br
HN
18
1.00 1.06 0.99 1.01 1.02 1.04 1.01 1.09 1.10 1.07 1.04 1.02 1.03 1.28 1.11 1.11 1.05
12.5 12.0 11.5 11.0 10.5 10.0 9.5 9.0 8.5 8.0 7.5 7.0 6.5 6.0 5.5 5.0 4.5 4.0 3.5 3.0 2.5 2.0 1.5 1.0 0.5
f1 (ppm)

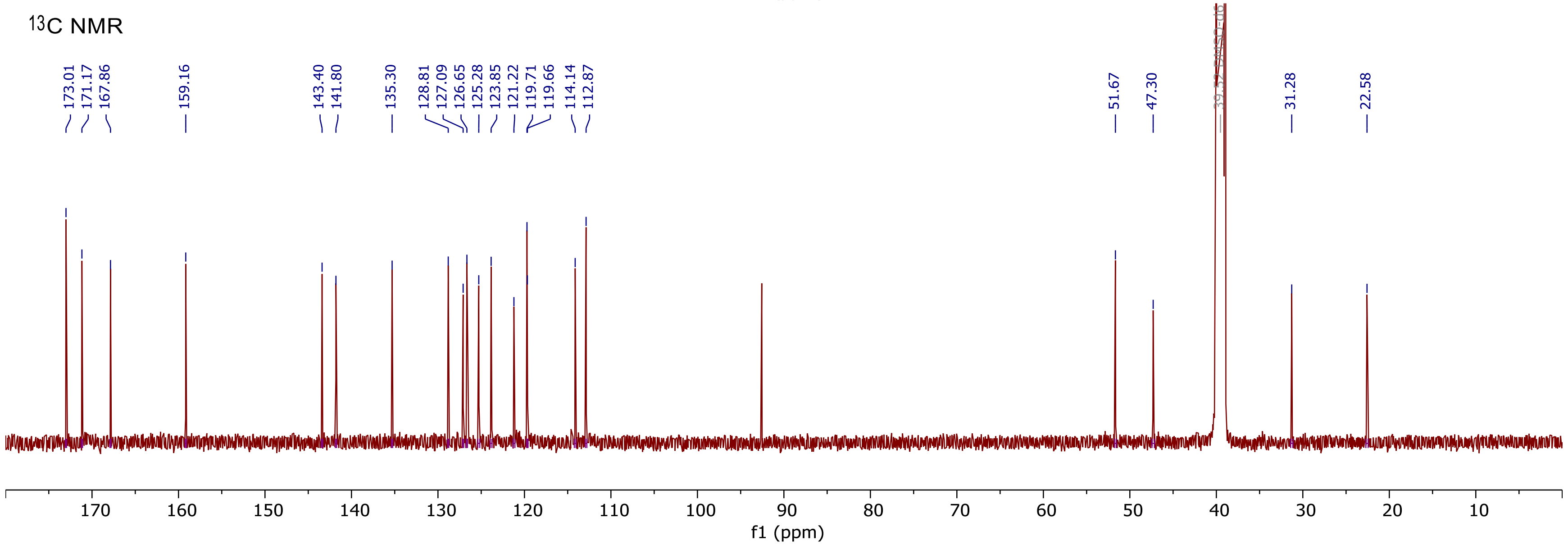
13C NMR
173.01 171.17 167.86
159.16
143.40 141.80
135.30
128.81 127.09 126.65 125.28 123.85 121.22 119.71 119.66 114.14 112.87
51.67
47.30
31.28
22.58
170 160 150 140 130 120 110 100 90 80 70 60 50 40 30 20 10
f1 (ppm)

^{1}H NMR

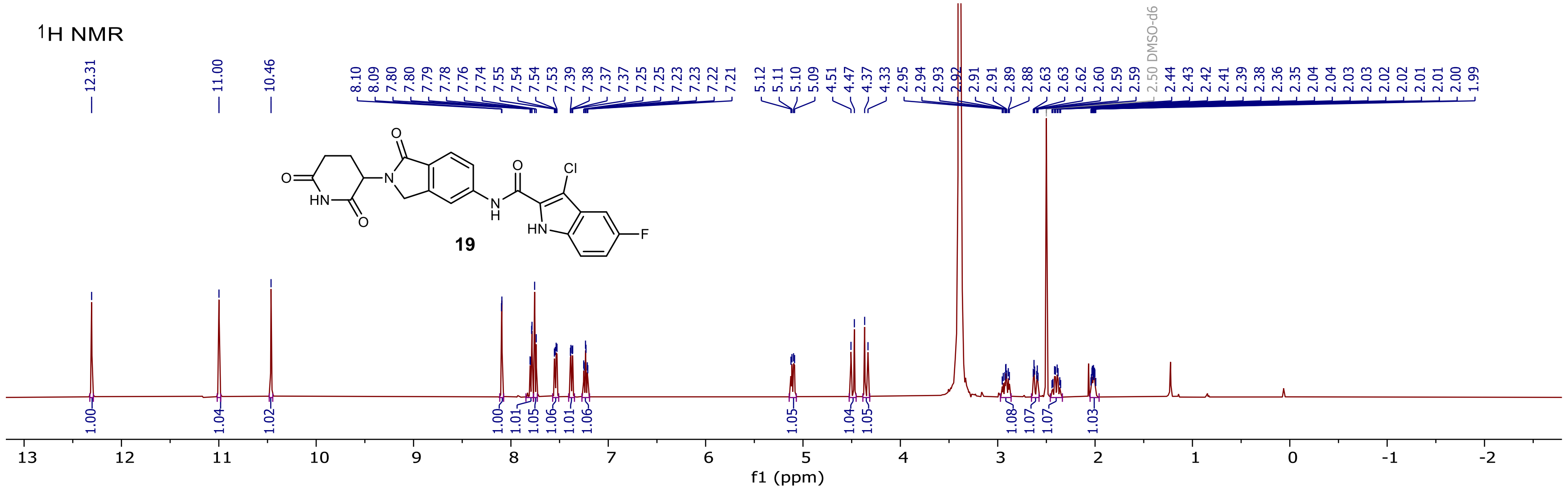

^{13}C NMR

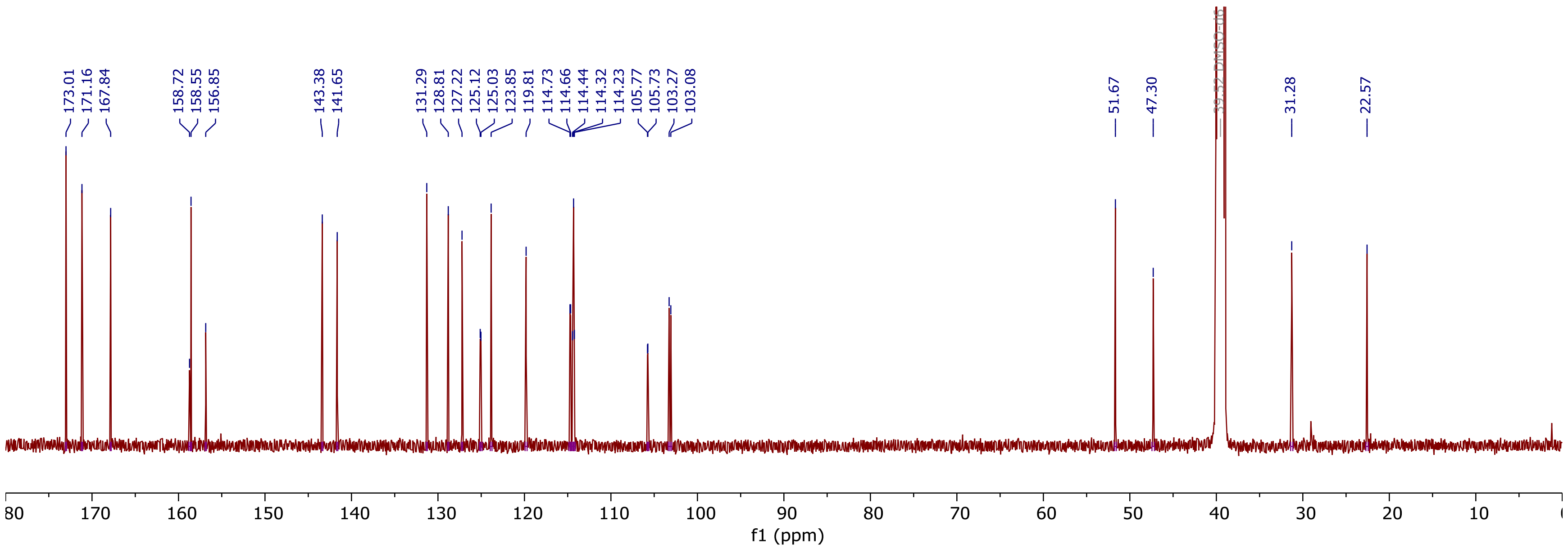

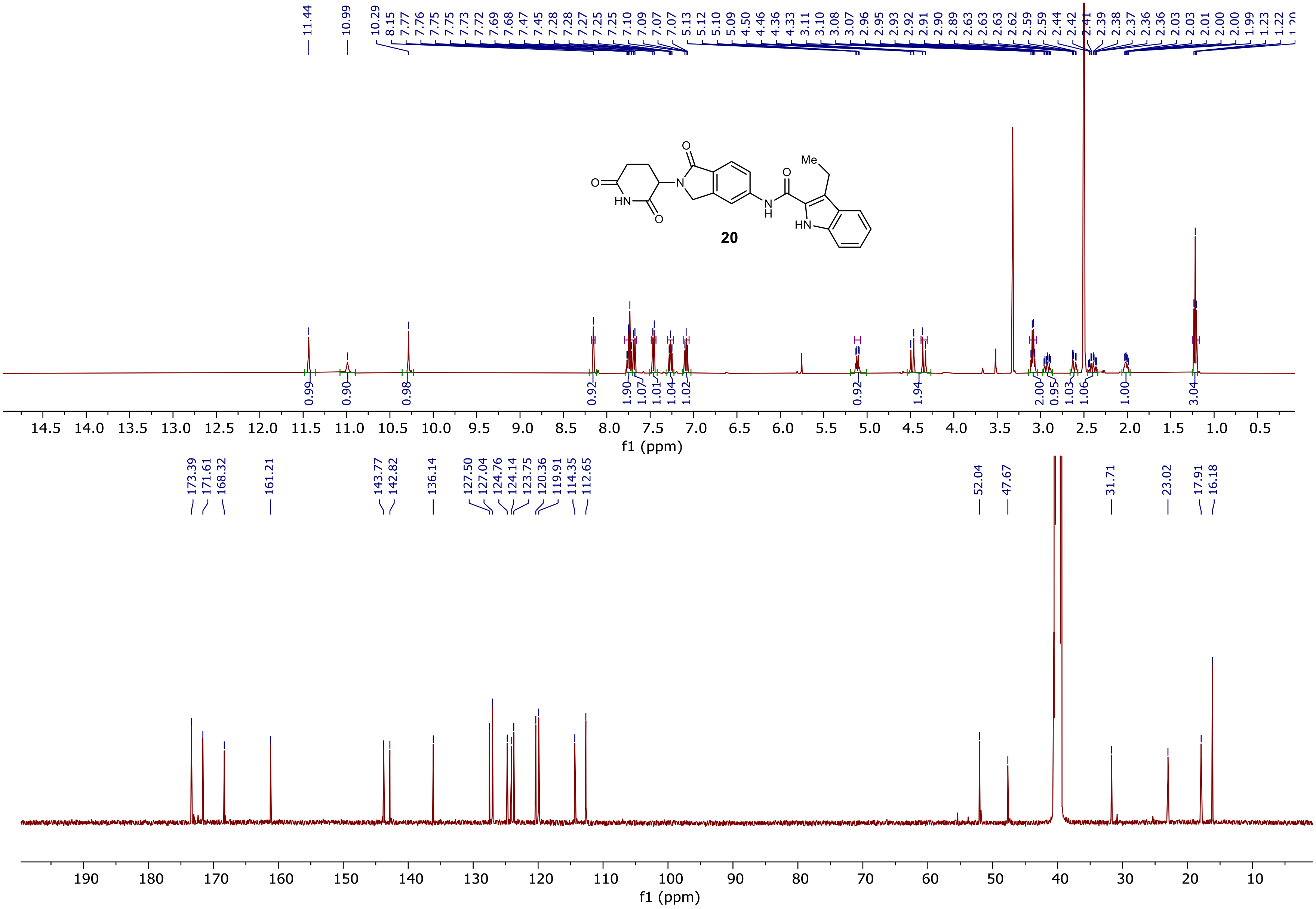
11.44
10.99
10.29 8.15 7.77 7.76 7.75 7.75 7.73 7.72 7.69 7.68 7.47 7.45 7.28 7.28 7.27 7.25 7.25 7.10 7.09 7.07 7.07
5.13 5.12 5.10 5.09 4.50 4.46 4.36 4.33 3.11 3.10 3.08 3.07 2.96 2.95 2.93 2.92 2.91 2.90 2.89 2.63 2.63 2.63 2.62 2.59 2.59 2.44 2.42 2.41 2.39 2.38 2.37 2.36 2.36 2.03 2.03 2.01 2.00 2.00 1.99 1.23 1.22
Me
O
HN
N
H
20
0.99 0.90 0.98 0.92 1.90 1.07 1.01 1.04 1.02 0.92 1.94 2.00 0.95 1.03 1.06 1.00 3.04
f1 (ppm)
173.39 171.61 168.32 161.21 143.77 142.82 136.14 127.50 127.04 124.76 124.14 123.75 120.36 119.91 114.35 112.65 52.04 47.67 31.71 23.02 17.91 16.18
f1 (ppm)

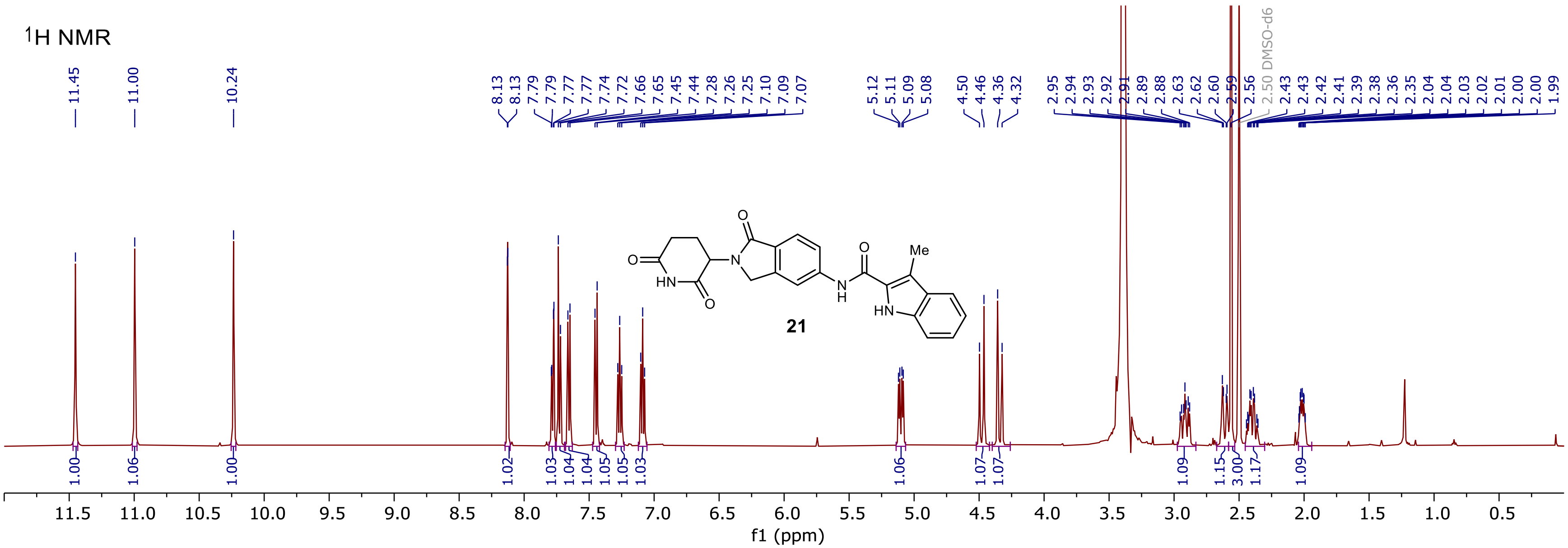

1H NMR
11.45
11.00
10.24
8.13 8.13 7.79 7.77 7.77 7.74 7.72 7.66 7.65 7.45 7.44 7.28 7.26 7.25 7.10 7.09 7.07
5.12 5.11 5.09 5.08
4.50 4.46 4.36 4.32
2.95 2.94 2.93 2.92 2.91 2.89 2.88 2.63 2.62 2.60 2.59 2.56 2.50 DMSO-d6 2.43 2.43 2.42 2.41 2.39 2.38 2.36 2.35 2.04 2.04 2.03 2.02 2.01 2.00 2.00 1.99
O
HN
N
O
O
NH
O
Me
HN
21
1.00 1.06 1.00 1.02 1.03 1.04 1.04 1.05 1.05 1.03 1.06 1.07 1.07 1.09 1.15 3.00 1.17 1.09
11.5 11.0 10.5 10.0 9.5 9.0 8.5 8.0 7.5 7.0 6.5 6.0 5.5 5.0 4.5 4.0 3.5 3.0 2.5 2.0 1.5 1.0 0.5
f1 (ppm)

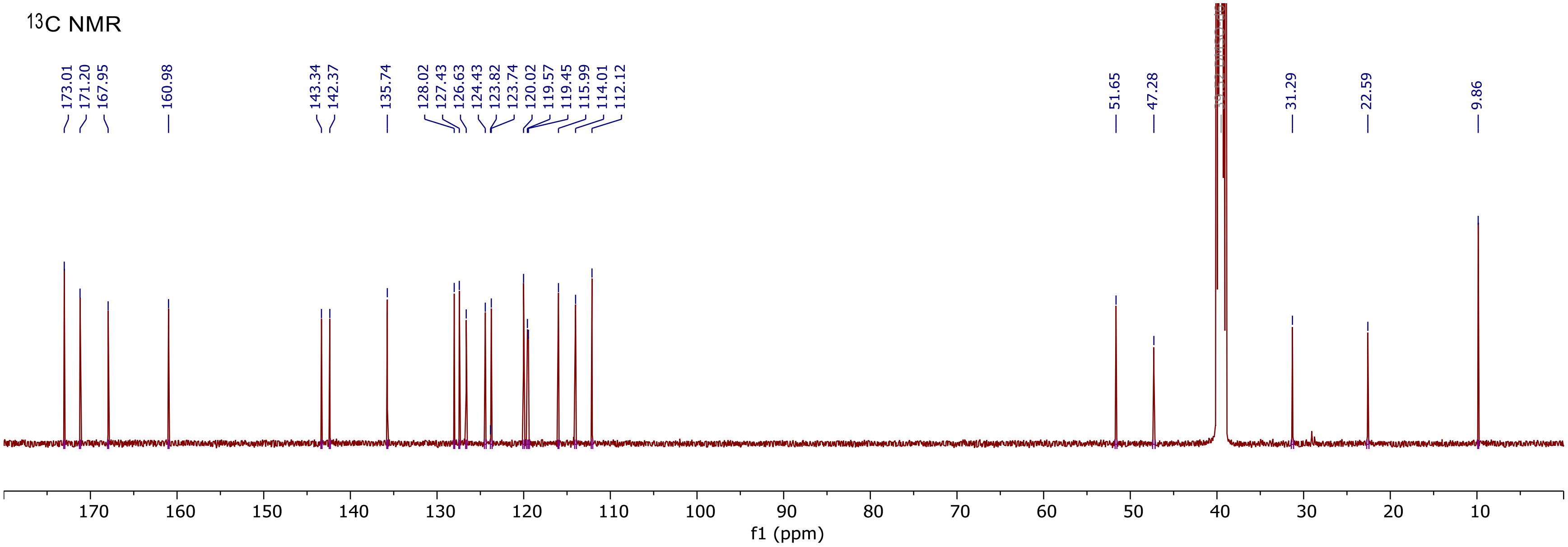

13C NMR
173.01 171.20 167.95
160.98
143.34 142.37
135.74
128.02 127.43 126.63 124.43 123.82 123.74 120.02 119.57 119.45 115.99 114.01 112.12
51.65
47.28
31.29
22.59
9.86
170 160 150 140 130 120 110 100 90 80 70 60 50 40 30 20 10
f1 (ppm)

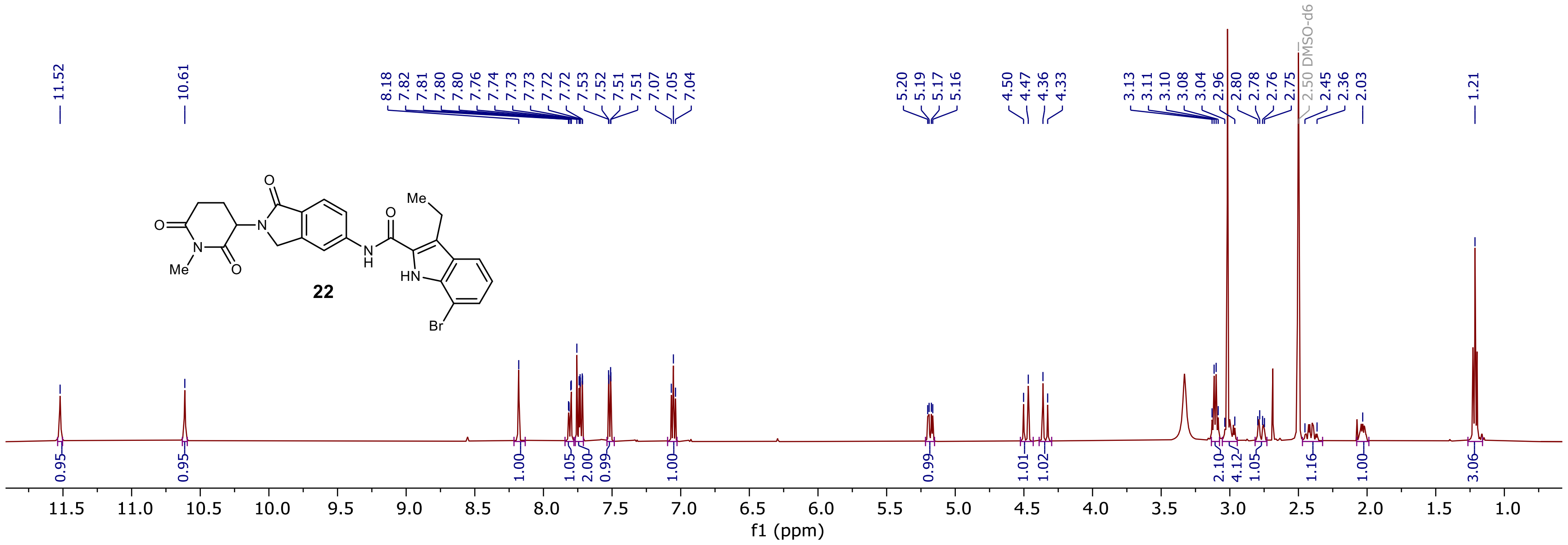
11.52
10.61
8.18
7.82
7.81
7.80
7.80
7.76
7.74
7.73
7.73
7.72
7.72
7.53
7.52
7.51
7.51
7.07
7.05
7.04
5.20
5.19
5.17
5.16
4.50
4.47
4.36
4.33
3.13
3.11
3.10
3.08
3.04
2.96
2.80
2.78
2.76
2.75
2.50 DMSO-d6
2.45
2.36
2.03
1.21
O
N
Me
O
O
N
H
Me
HN
Br
22
0.95
0.95
1.00
1.05
2.00
0.99
1.00
0.99
1.01
1.02
2.10
4.12
1.05
1.16
1.00
3.06
11.5 11.0 10.5 10.0 9.5 9.0 8.5 8.0 7.5 7.0 6.5 6.0 5.5 5.0 4.5 4.0 3.5 3.0 2.5 2.0 1.5 1.0
f1 (ppm)

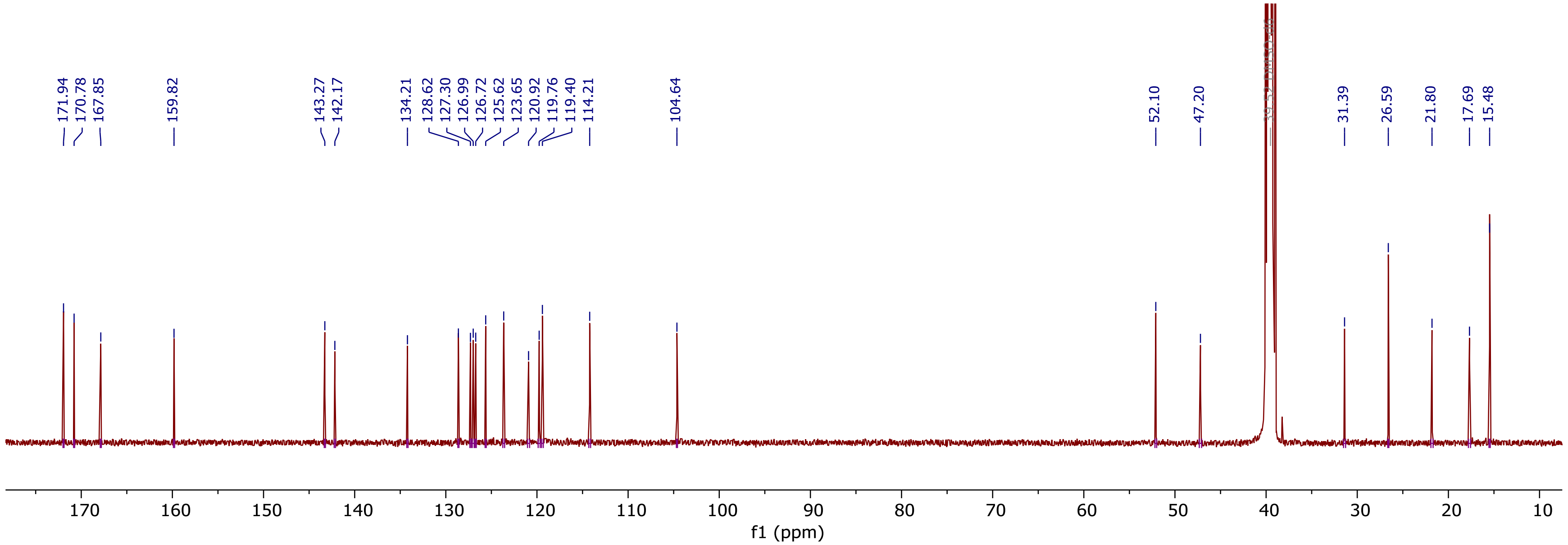
171.94
170.78
167.85
159.82
143.27
142.17
134.21
128.62
127.30
126.99
126.72
125.62
123.65
120.92
119.76
119.40
114.21
104.64
52.10
47.20
31.39
26.59
21.80
17.69
15.48
170 160 150 140 130 120 110 100 90 80 70 60 50 40 30 20 10
f1 (ppm)

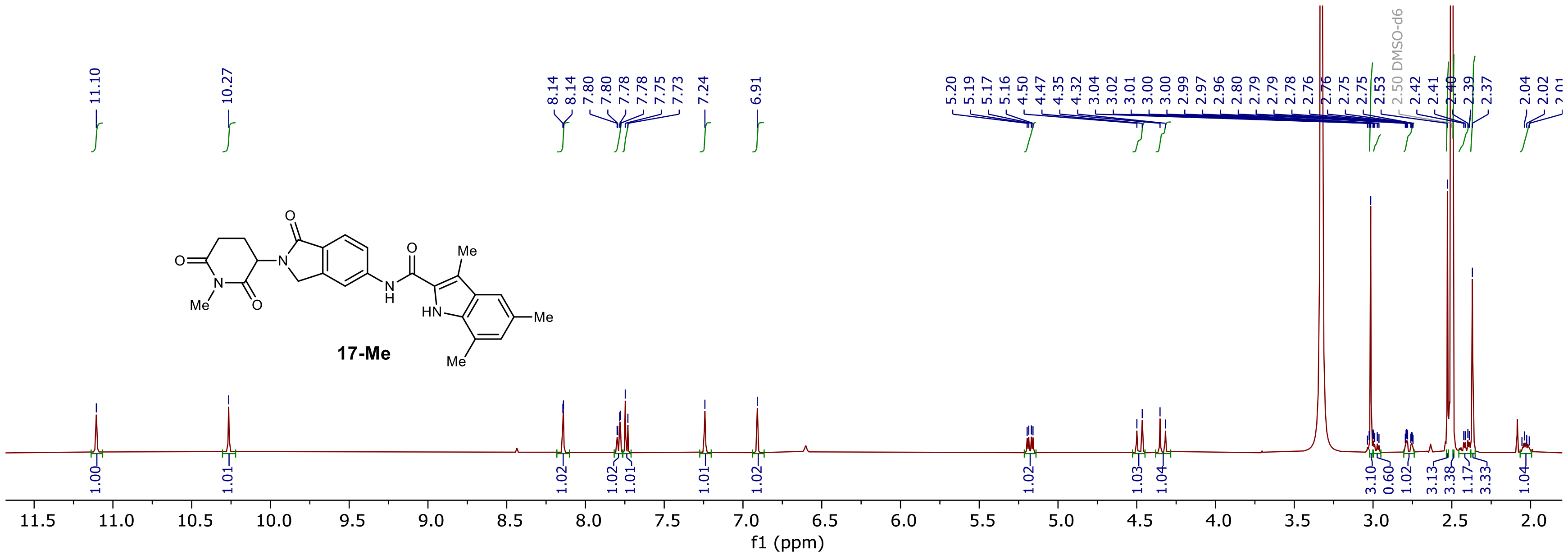

17-Me
O
N
H
HN
Me
11.10
10.27
8.14
8.14
7.80
7.80
7.78
7.78
7.75
7.73
7.24
6.91
5.20
5.19
5.17
5.16
4.50
4.47
4.35
4.32
3.04
3.02
3.01
3.00
2.99
2.97
2.96
2.80
2.79
2.79
2.78
2.76
2.76
2.75
2.75
2.53
2.50 DMSO-d6
2.42
2.41
2.40
2.39
2.37
2.04
2.02
1.00
1.01
1.02
1.02
1.01
1.01
1.02
1.02
1.03
1.04
3.10
0.60
1.02
3.13
3.38
1.17
3.33
1.04
11.5
11.0
10.5
10.0
9.5
9.0
8.5
8.0
7.5
7.0
6.5
6.0
5.5
5.0
4.5
4.0
3.5
3.0
2.5
2.0
f1 (ppm)

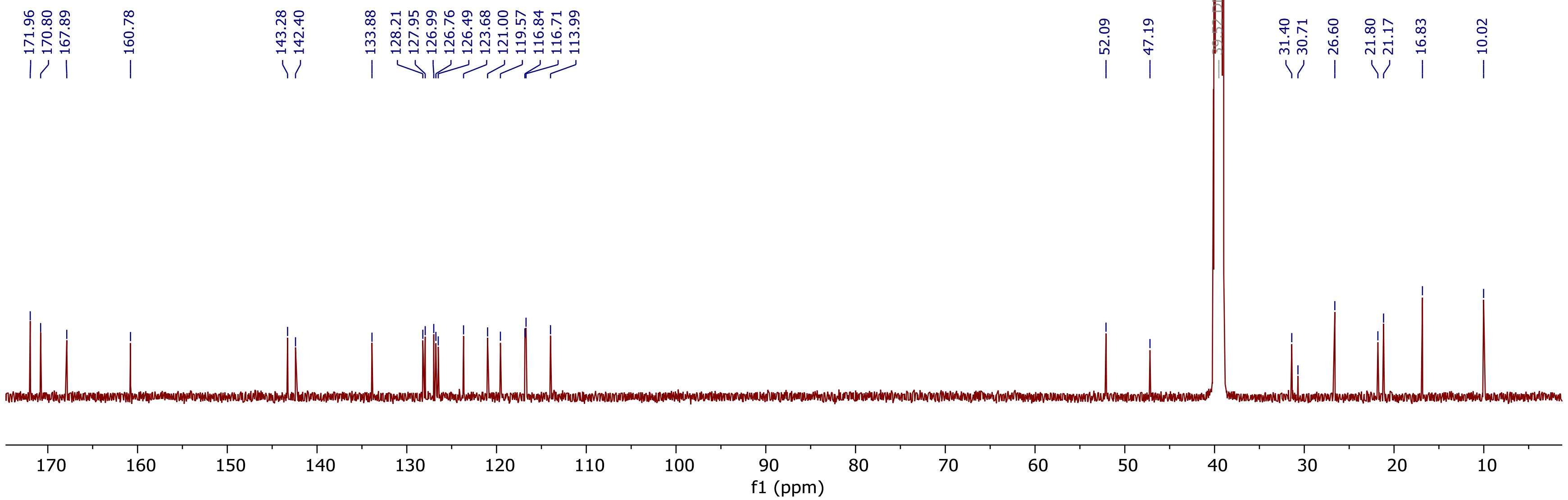

171.96
170.80
167.89
160.78
143.28
142.40
133.88
128.21
127.95
126.99
126.76
126.49
123.68
121.00
119.57
116.84
116.71
113.99
52.09
47.19
31.40
30.71
26.60
21.80
21.17
16.83
10.02
170
160
150
140
130
120
110
100
90
80
70
60
50
40
30
20
10
f1 (ppm)

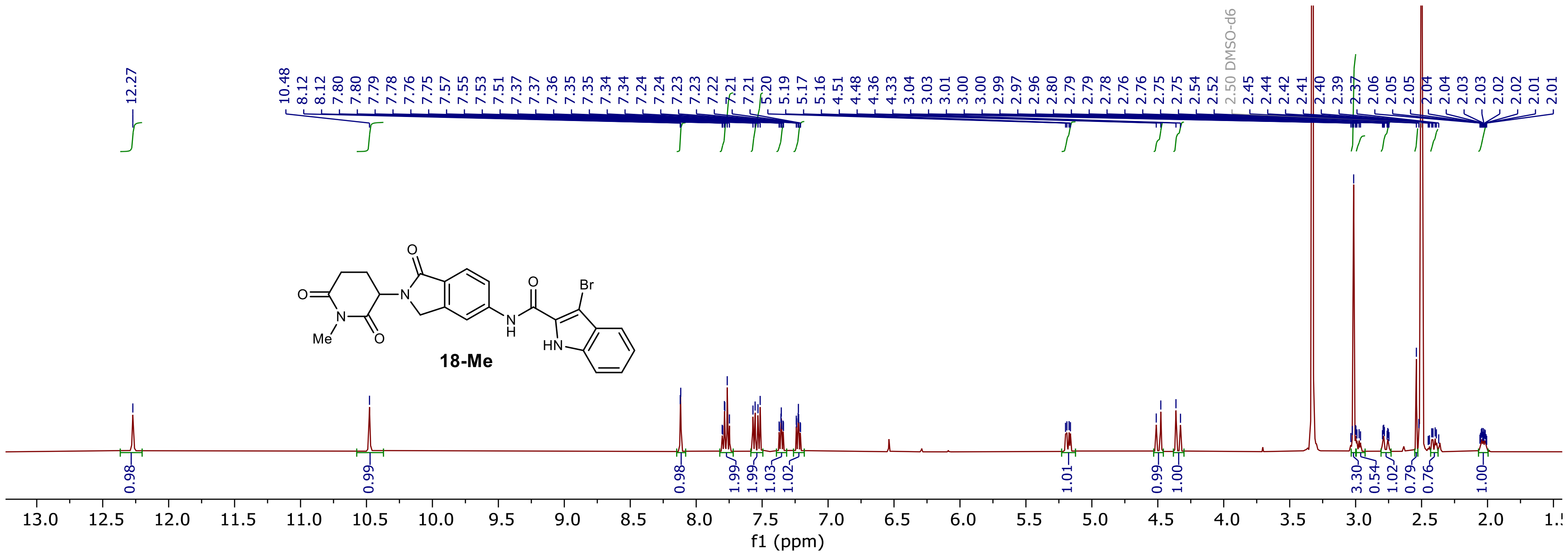
12.27
10.48 8.12 8.12 7.80 7.79 7.78 7.76 7.75 7.57 7.55 7.53 7.51 7.37 7.37 7.36 7.35 7.35 7.34 7.24 7.24 7.23 7.23 7.22 7.21 7.21 5.20 5.19 5.17 5.16 4.51 4.48 4.36 4.33 3.04 3.03 3.01 3.00 3.00 2.99 2.97 2.96 2.80 2.79 2.79 2.78 2.76 2.76 2.75 2.75 2.54 2.52 2.50 DMSO-d6 2.45 2.44 2.42 2.41 2.40 2.39 2.37 2.06 2.05 2.05 2.04 2.04 2.03 2.02 2.02 2.01
Br
N
H
HN
Me
18-Me
0.98 0.99 0.98 1.99 1.99 1.03 1.02 1.01 0.99 1.00 3.30 0.54 1.02 0.79 0.76 1.00
13.0 12.5 12.0 11.5 11.0 10.5 10.0 9.5 9.0 8.5 8.0 7.5 7.0 6.5 6.0 5.5 5.0 4.5 4.0 3.5 3.0 2.5 2.0
f1 (ppm)

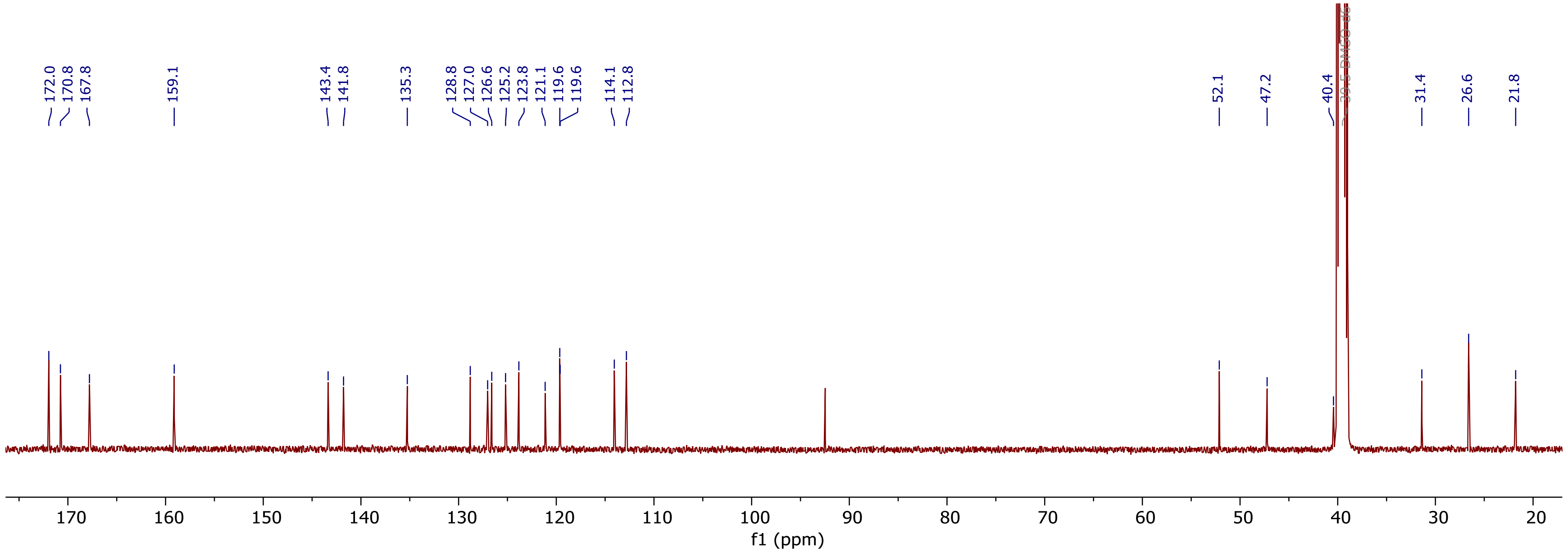
172.0 170.8 167.8 159.1 143.4 141.8 135.3 128.8 127.0 126.6 125.2 123.8 121.1 119.6 119.6 114.1 112.8 52.1 47.2 40.4 39.5 DMSO-d6 31.4 26.6 21.8
170 160 150 140 130 120 110 100 90 80 70 60 50 40 30 20
f1 (ppm)

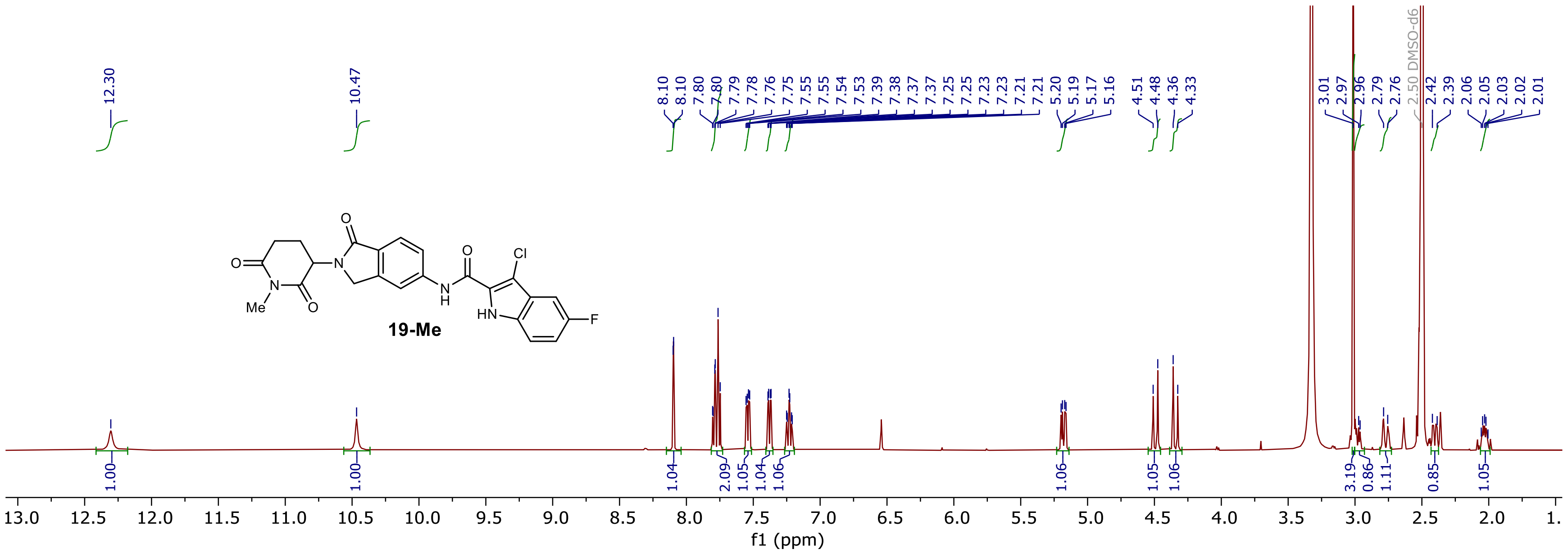

12.30
10.47
8.10 8.10 7.80 7.80 7.79 7.78 7.76 7.75 7.55 7.55 7.54 7.53 7.39 7.38 7.37 7.37 7.25 7.25 7.23 7.23 7.21 7.21
5.20 5.19 5.17 5.16
4.51 4.48 4.36 4.33
3.01 2.97 2.96 2.79 2.76
2.50 DMSO-d6
2.42 2.39 2.06 2.05 2.03 2.02 2.01
O
O
N
N
Me
O
O
N
H
O
Cl
HN
F
19-Me
1.00 1.00 1.04 2.09 1.05 1.04 1.06 1.06 1.05 1.06 3.19 0.86 1.11 0.85 1.05
13.0 12.5 12.0 11.5 11.0 10.5 10.0 9.5 9.0 8.5 8.0 7.5 7.0 6.5 6.0 5.5 5.0 4.5 4.0 3.5 3.0 2.5 2.0 1.
f1 (ppm)

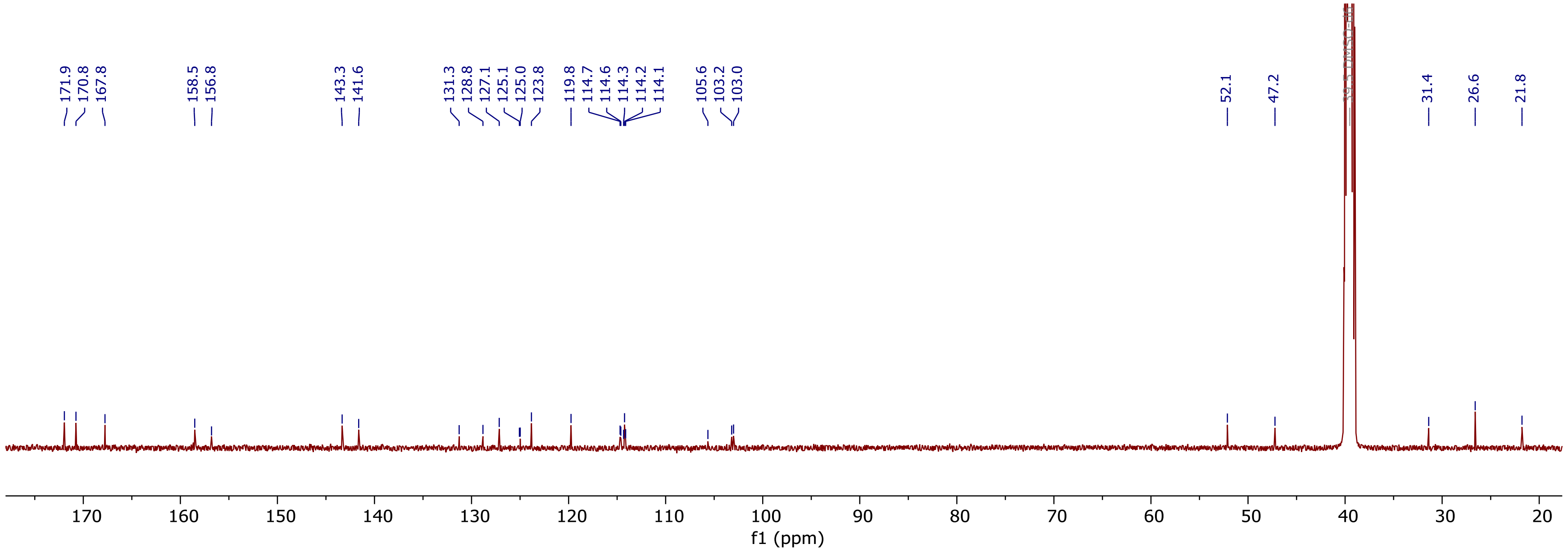

171.9 170.8 167.8
158.5 156.8
143.3 141.6
131.3 128.8 127.1 125.1 125.0 123.8
119.8 114.7 114.6 114.3 114.2 114.1
105.6 103.2 103.0
52.1 47.2
31.4 26.6 21.8
170 160 150 140 130 120 110 100 90 80 70 60 50 40 30 20
f1 (ppm)

^{1}H NMR

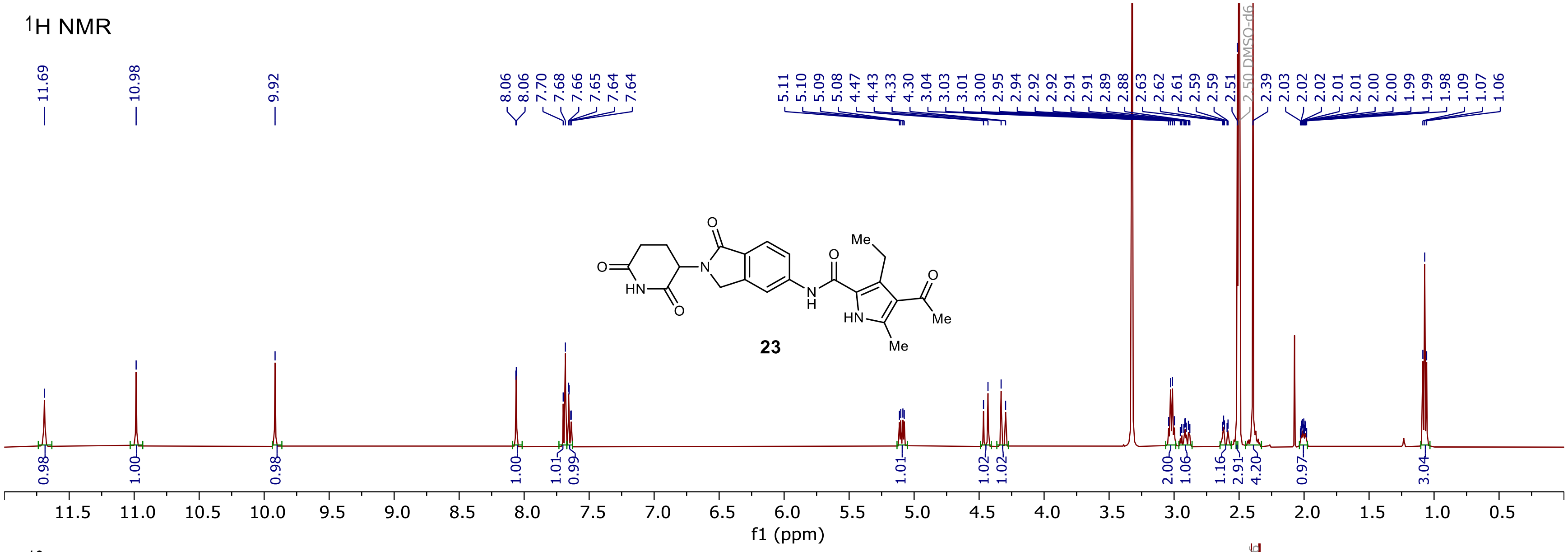

^{13}C NMR

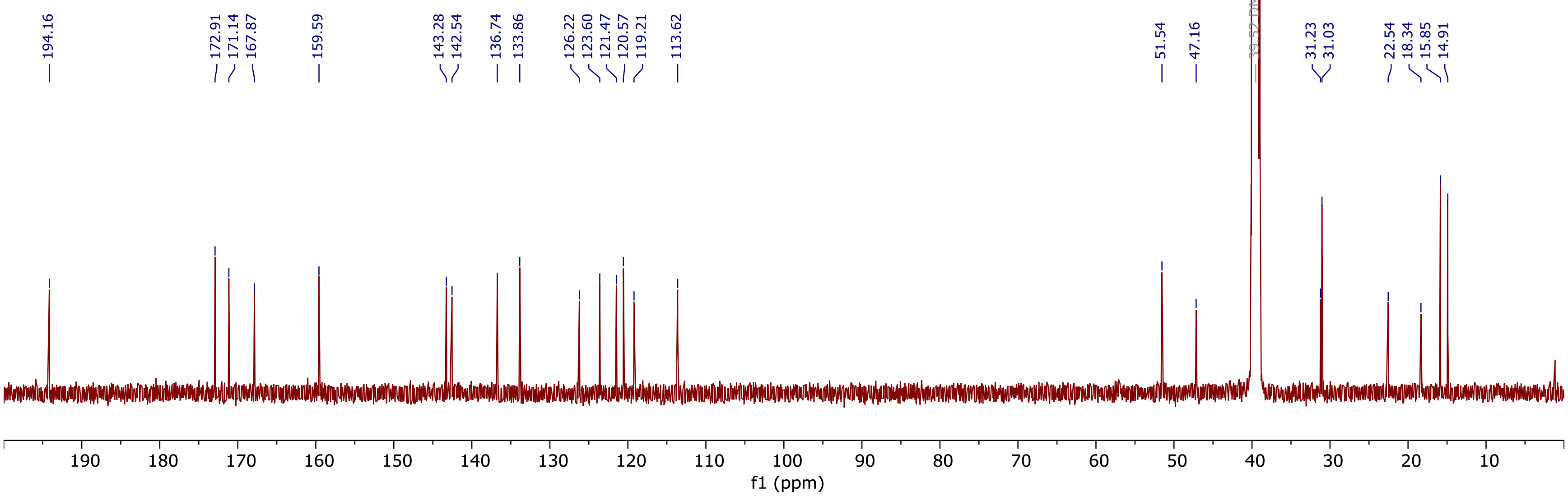

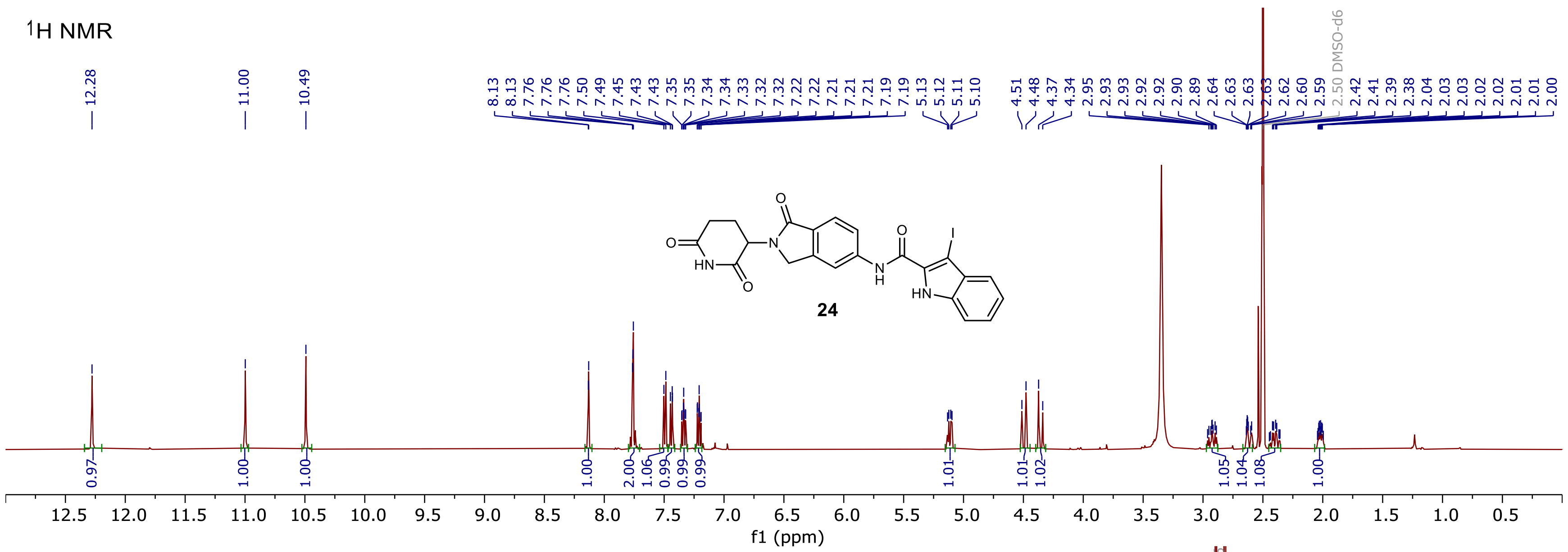
1H NMR
12.28
11.00
10.49
8.13 8.13 7.76 7.76 7.76 7.50 7.49 7.45 7.43 7.43 7.35 7.35 7.34 7.34 7.33 7.32 7.32 7.22 7.22 7.21 7.21 7.21 7.19 7.19
5.13 5.12 5.11 5.10
4.51 4.48 4.37 4.34
2.95 2.93 2.93 2.92 2.92 2.90 2.89 2.64 2.63 2.63 2.63 2.62 2.60 2.59
2.50 DMSO-d6
2.42 2.41 2.39 2.38 2.04 2.03 2.03 2.02 2.02 2.01 2.01 2.00
0.97 1.00 1.00 1.00 2.00 1.06 0.99 0.99 0.99 1.01 1.01 1.02 1.05 1.04 1.08 1.00
O
N
HN
O
O
N
H
O
I
HN
24
12.5 12.0 11.5 11.0 10.5 10.0 9.5 9.0 8.5 8.0 7.5 7.0 6.5 6.0 5.5 5.0 4.5 4.0 3.5 3.0 2.5 2.0 1.5 1.0 0.5
f1 (ppm)

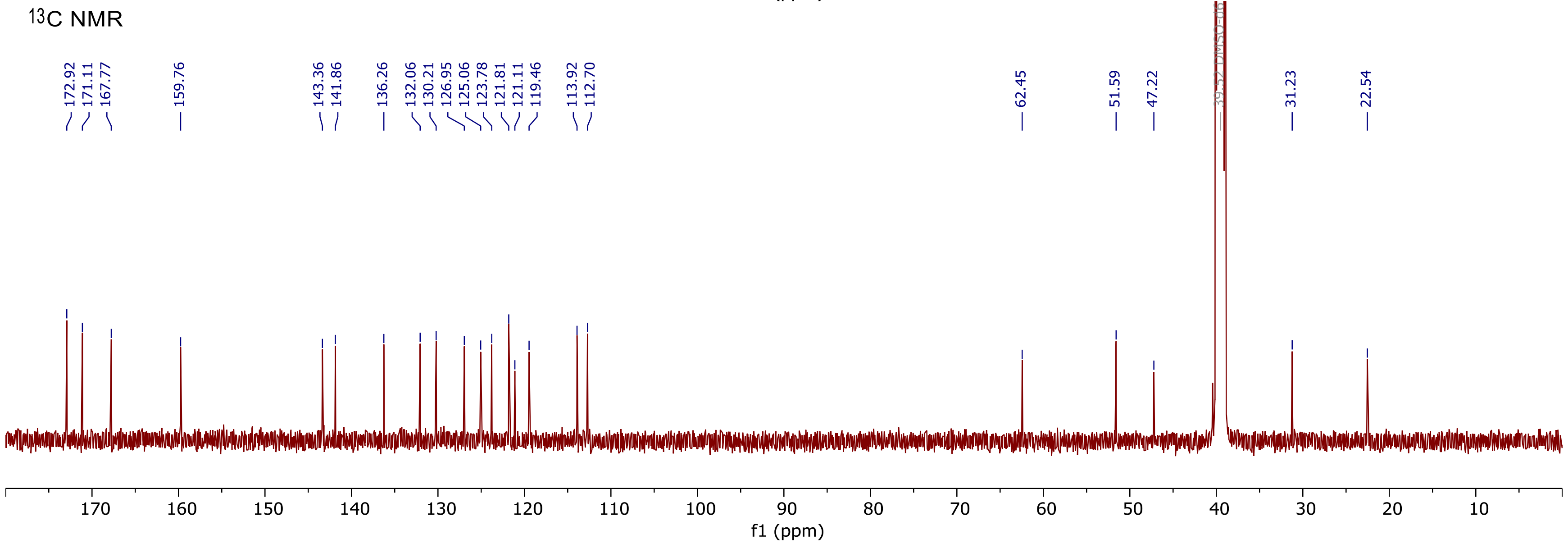
13C NMR
172.92 171.11 167.77
159.76
143.36 141.86
136.26
132.06 130.21 126.95 125.06 123.78 121.81 121.11 119.46
113.92 112.70
62.45
51.59
47.22
39.52 DMSO-d6
31.23
22.54
170 160 150 140 130 120 110 100 90 80 70 60 50 40 30 20 10
f1 (ppm)

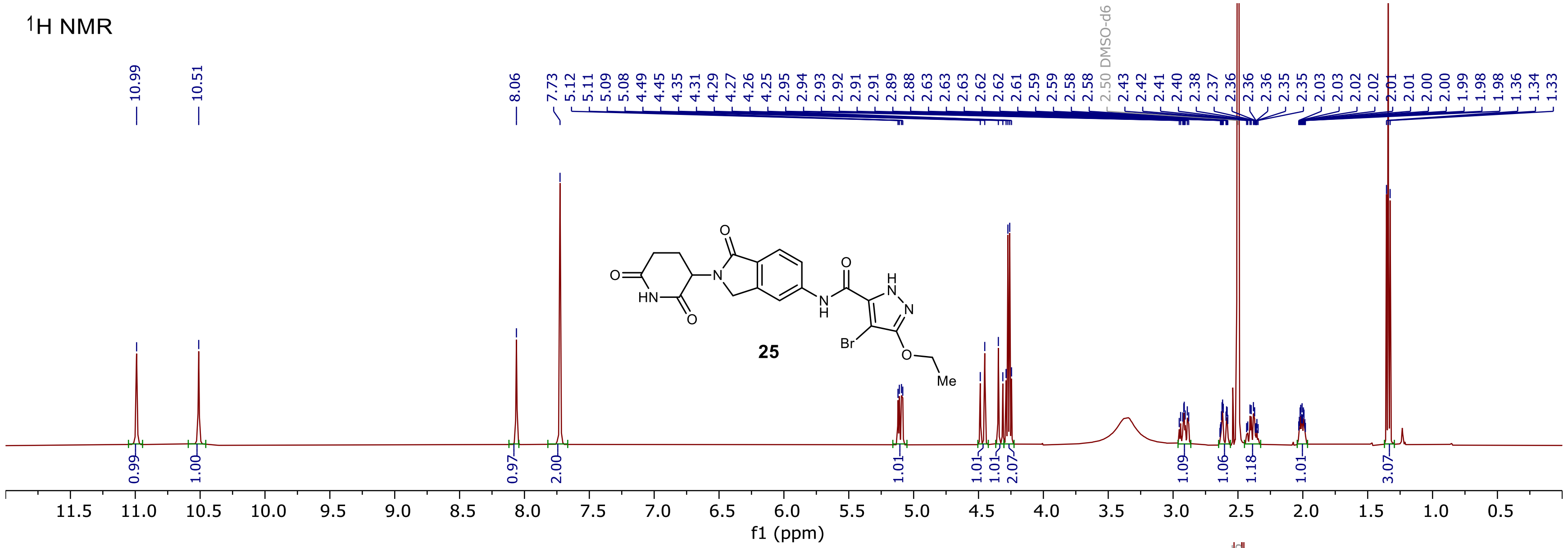
1H NMR
10.99
10.51
8.06
7.73
5.12
5.11
5.09
5.08
4.49
4.45
4.35
4.31
4.29
4.27
4.26
4.25
2.95
2.94
2.93
2.92
2.91
2.91
2.89
2.88
2.63
2.63
2.63
2.62
2.62
2.61
2.59
2.59
2.58
2.58
2.50 DMSO-d6
2.43
2.42
2.41
2.40
2.38
2.37
2.36
2.36
2.36
2.35
2.35
2.03
2.03
2.02
2.02
2.01
2.01
2.00
2.00
1.99
1.98
1.98
1.36
1.34
1.33
0.99
1.00
0.97
2.00
1.01
1.01
1.01
2.07
1.09
1.06
1.18
1.01
3.07
O
N
O
HN
O
N
H
O
H
N
N
Br
O
Me
25
f1 (ppm)

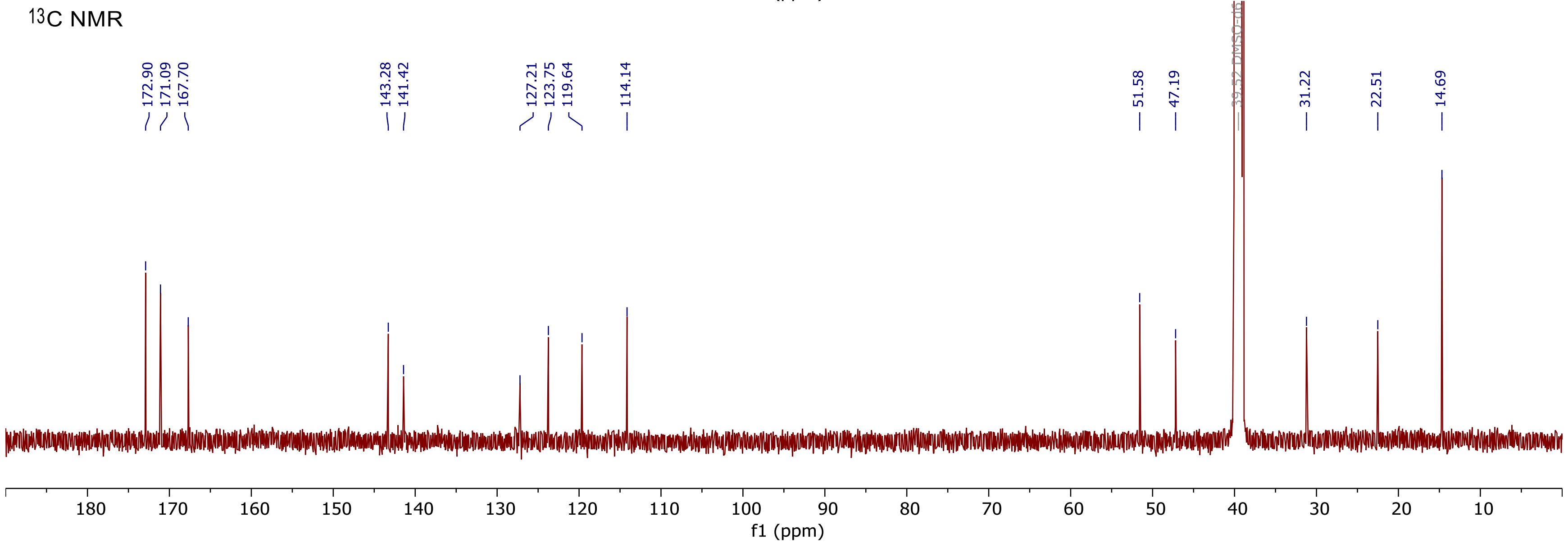
13C NMR
172.90
171.09
167.70
143.28
141.42
127.21
123.75
119.64
114.14
51.58
47.19
39.52 DMSO-d6
31.22
22.51
14.69
f1 (ppm)

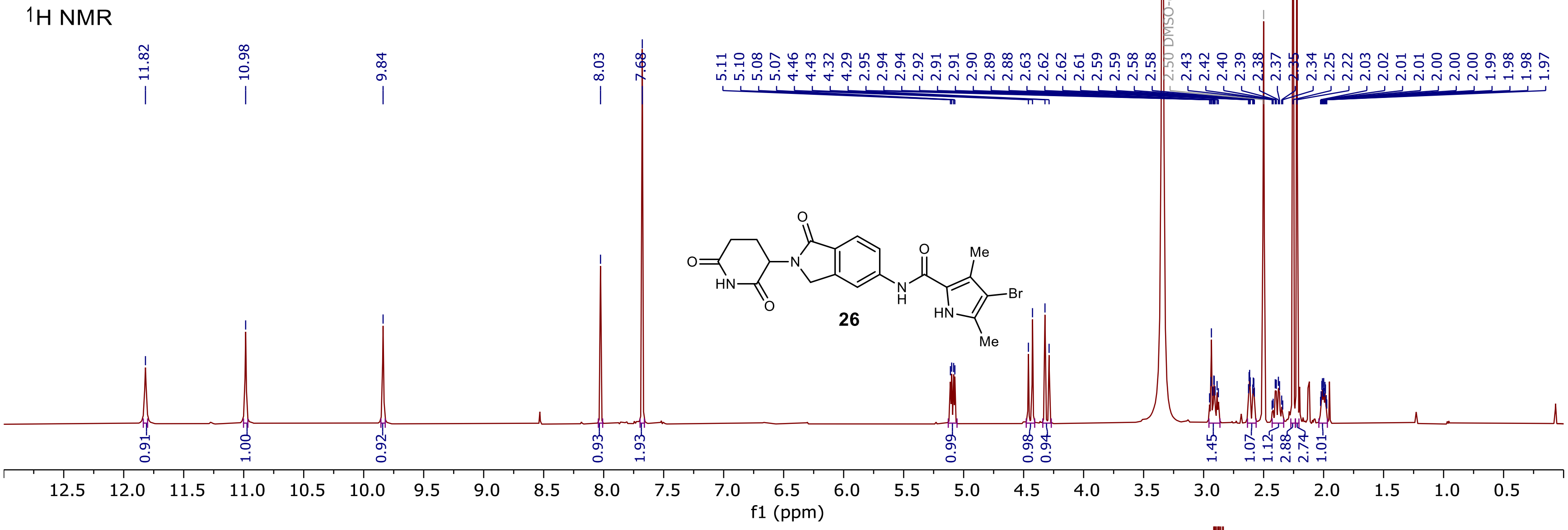

1H NMR
11.82
10.98
9.84
8.03
7.68
5.11 5.10 5.08 5.07 4.46 4.43 4.32 4.29 2.95 2.94 2.94 2.92 2.91 2.91 2.90 2.89 2.88 2.63 2.62 2.61 2.59 2.59 2.58 2.58 2.50 DMSO-d6 2.43 2.42 2.40 2.39 2.38 2.37 2.35 2.34 2.25 2.22 2.03 2.02 2.01 2.01 2.00 2.00 1.99 1.98 1.98 1.97
0.91 1.00 0.92 0.93 1.93 0.99 0.98 0.94 1.45 1.07 1.12 2.88 2.74 1.01
f1 (ppm)
Me
Br
Me
HN
HN
N
H
O
26

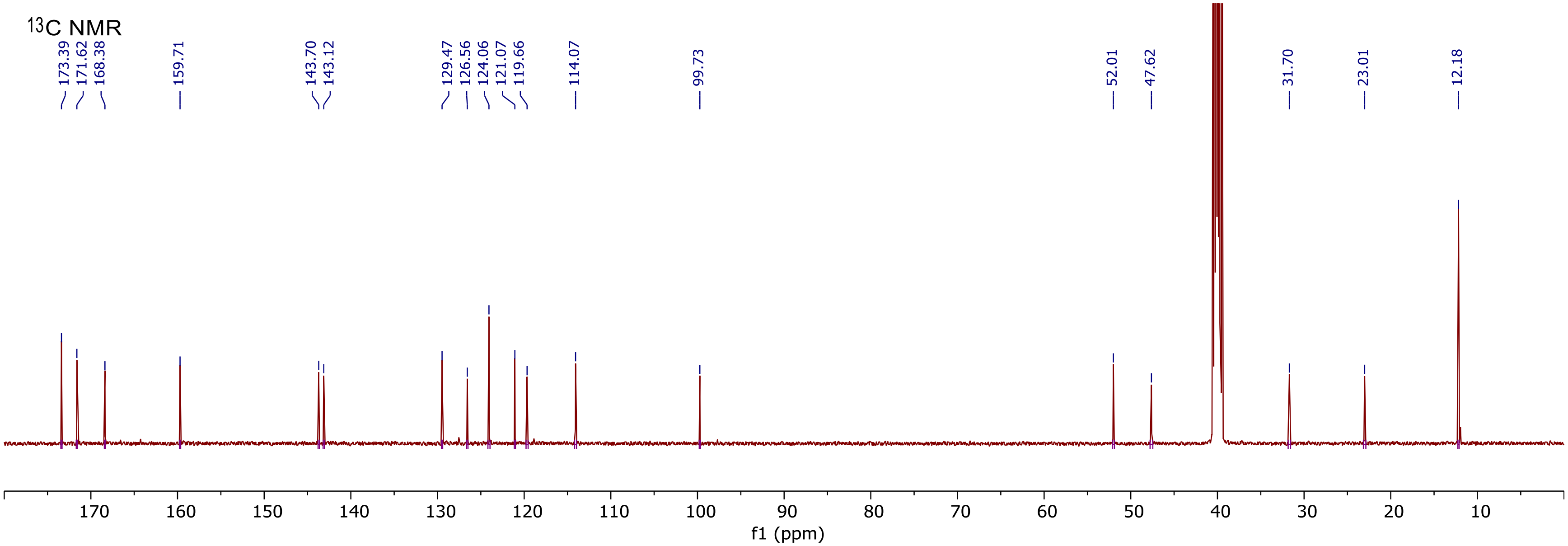

13C NMR
173.39 171.62 168.38
159.71
143.70 143.12
129.47 126.56 124.06 121.07 119.66
114.07
99.73
52.01 47.62
31.70
23.01
12.18
f1 (ppm)

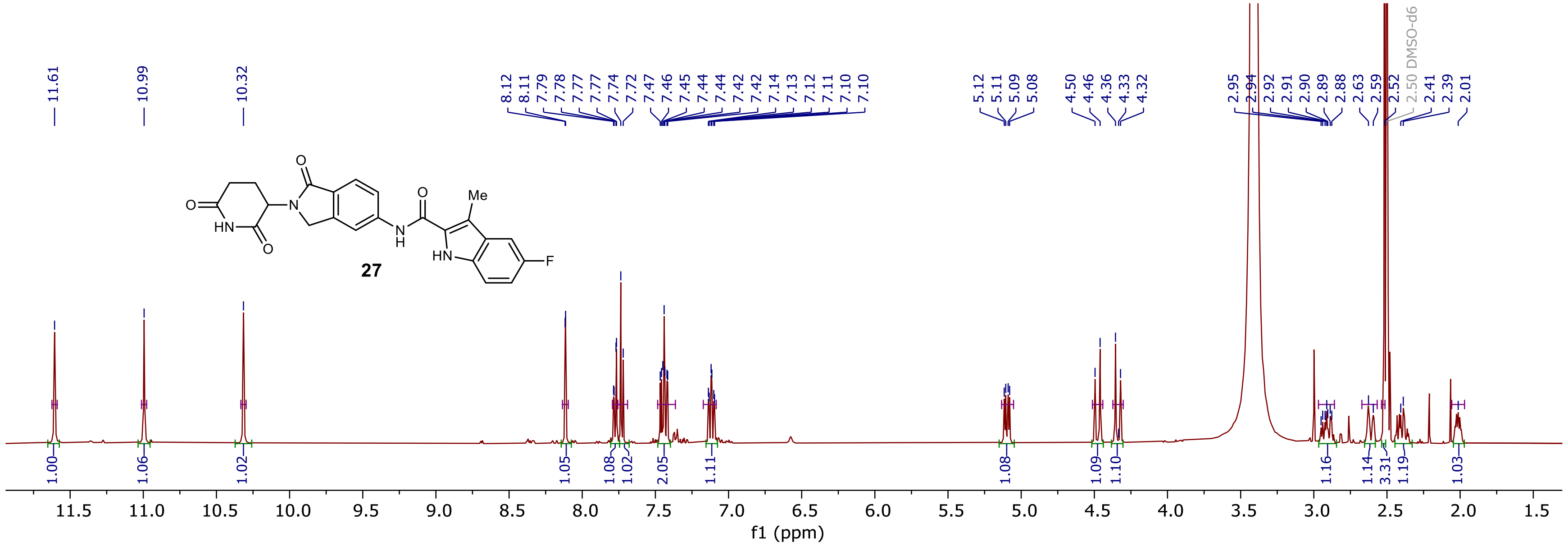
11.61
10.99
10.32
8.12 8.11 7.79 7.78 7.77 7.77 7.74 7.72 7.47 7.46 7.45 7.44 7.44 7.42 7.42 7.14 7.13 7.12 7.11 7.10 7.10
5.12 5.11 5.09 5.08
4.50 4.46 4.36 4.33 4.32
2.95 2.94 2.92 2.91 2.90 2.89 2.88 2.63 2.59 2.52 2.50 DMSO-d6 2.41 2.39 2.01
Me
O
O
N
HN
O
O
N
H
HN
F
27
1.00 1.06 1.02 1.05 1.08 1.02 2.05 1.11 1.08 1.09 1.10 1.16 1.14 3.31 1.19 1.03
11.5 11.0 10.5 10.0 9.5 9.0 8.5 8.0 7.5 7.0 6.5 6.0 5.5 5.0 4.5 4.0 3.5 3.0 2.5 2.0 1.5
f1 (ppm)

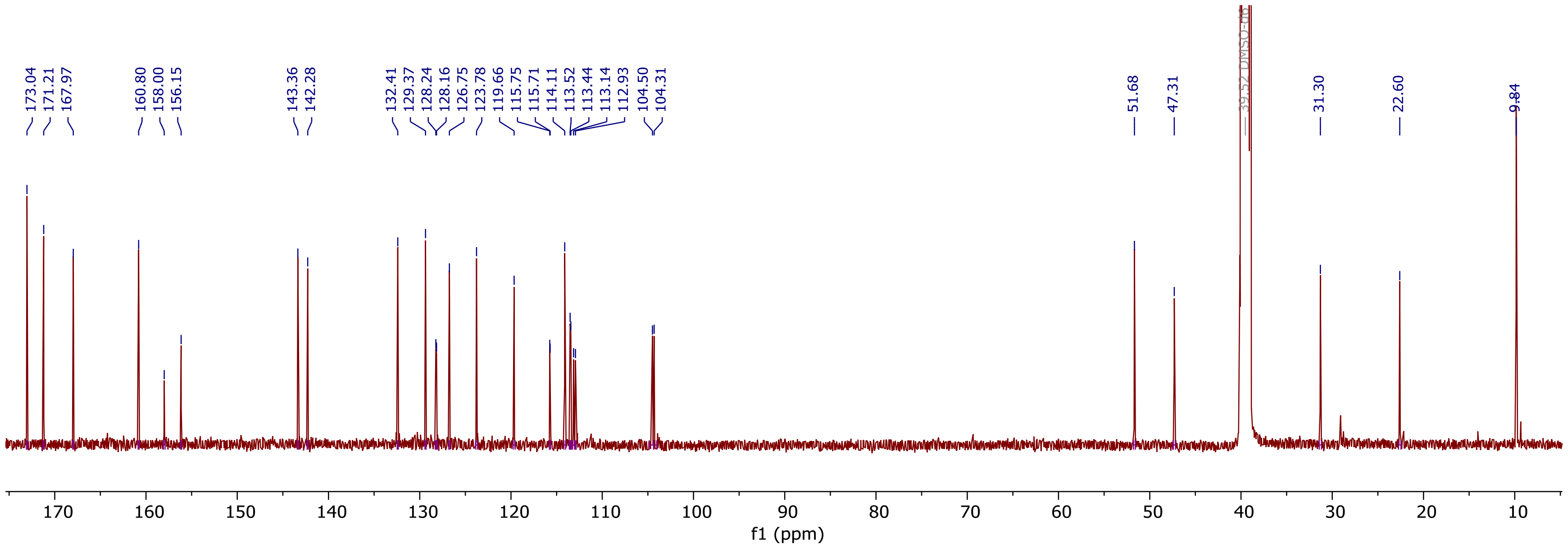
173.04 171.21 167.97
160.80 158.00 156.15
143.36 142.28
132.41 129.37 128.24 128.16 126.75 123.78 119.66 115.75 115.71 114.11 113.52 113.44 113.14 112.93 104.50 104.31
51.68
47.31
39.52 DMSO-d6
31.30
22.60
9.84
170 160 150 140 130 120 110 100 90 80 70 60 50 40 30 20 10
f1 (ppm)

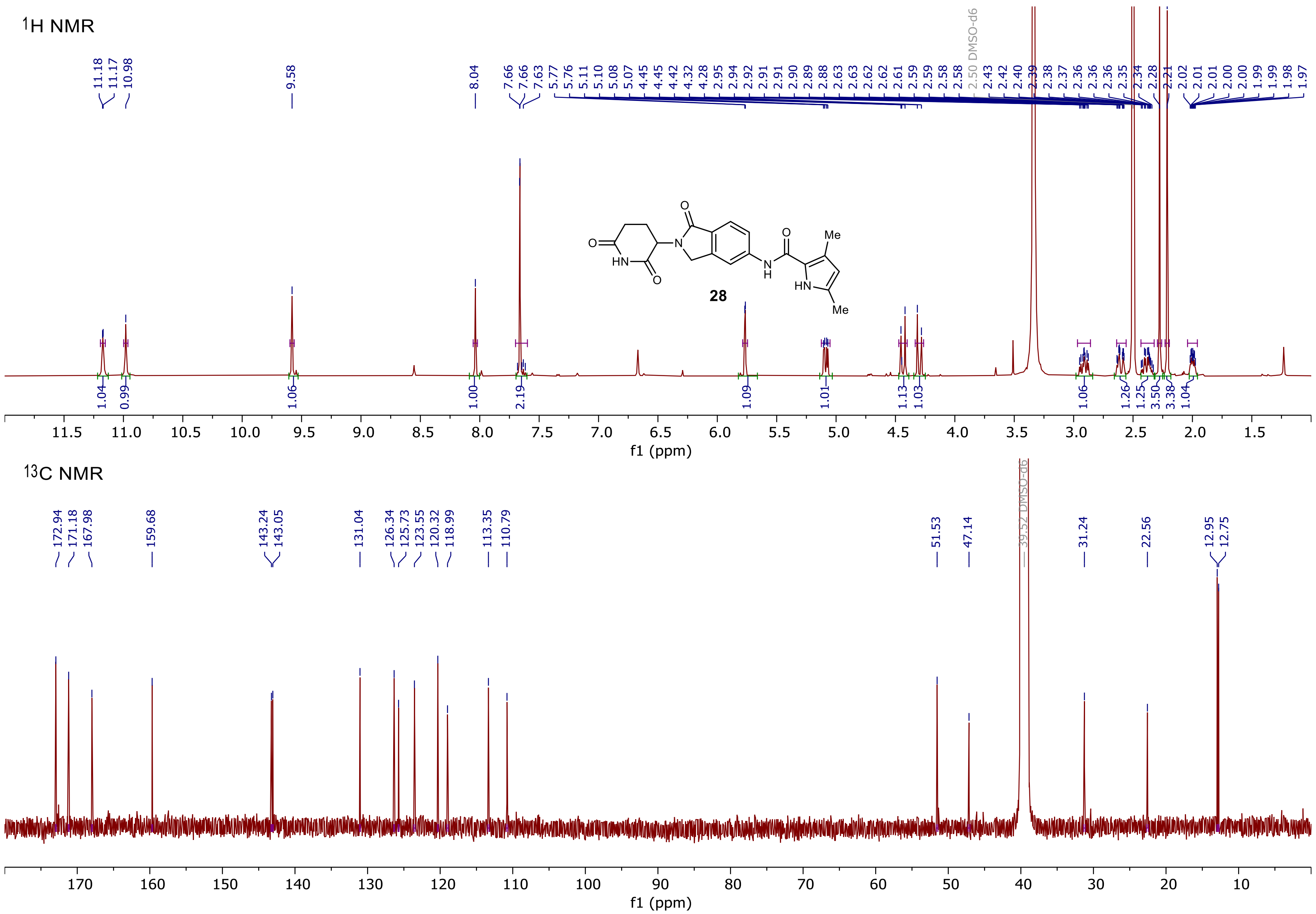

1H NMR
11.18
11.17
10.98
9.58
8.04
7.66
7.66
7.63
5.77
5.76
5.11
5.10
5.08
5.07
4.45
4.45
4.42
4.32
4.28
2.95
2.94
2.92
2.91
2.91
2.90
2.89
2.88
2.63
2.63
2.62
2.62
2.61
2.59
2.59
2.58
2.58
2.50 DMSO-d6
2.43
2.42
2.40
2.39
2.38
2.37
2.36
2.36
2.36
2.35
2.34
2.28
2.21
2.02
2.01
2.01
2.00
2.00
1.99
1.99
1.98
1.97
1.04
0.99
1.06
1.00
2.19
1.09
1.01
1.13
1.03
1.06
1.26
1.25
3.50
3.38
1.04
f1 (ppm)
O
O
HN
O
N
O
N
H
Me
HN
Me
28
13C NMR
172.94
171.18
167.98
159.68
143.24
143.05
131.04
126.34
125.73
123.55
120.32
118.99
113.35
110.79
51.53
47.14
39.52 DMSO-d6
31.24
22.56
12.95
12.75
f1 (ppm)

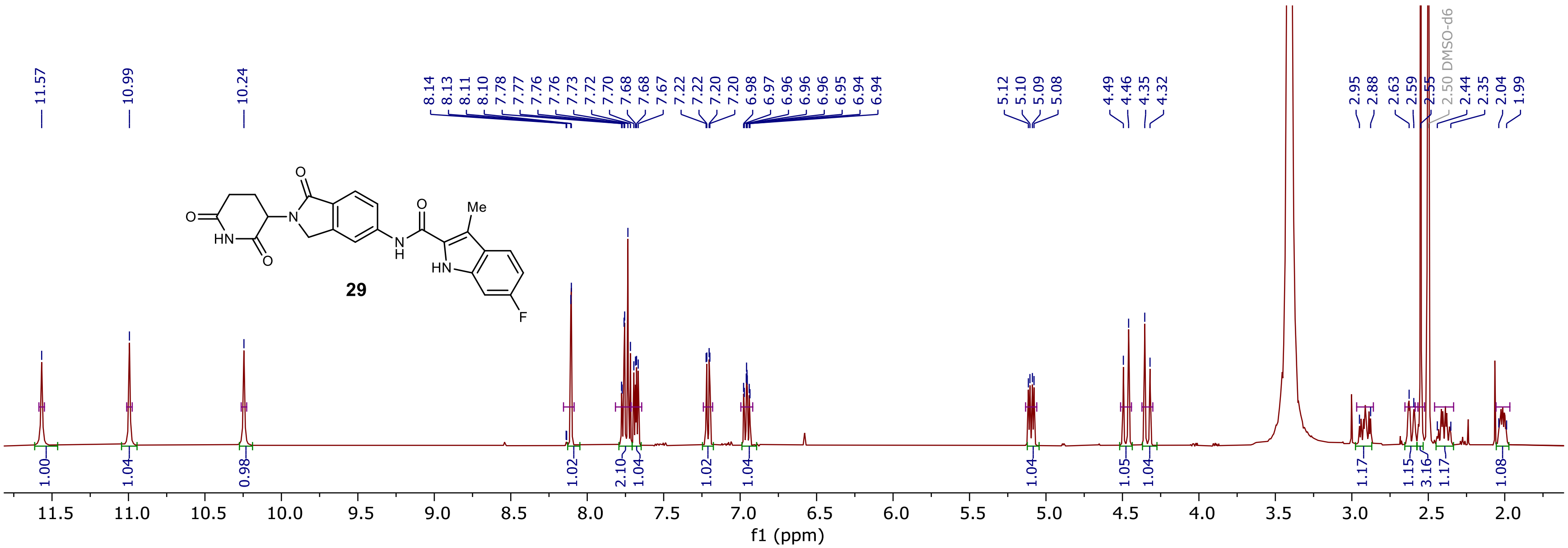
11.57
10.99
10.24
8.14 8.13 8.11 8.10 7.78 7.77 7.76 7.76 7.73 7.72 7.70 7.68 7.68 7.67 7.22 7.22 7.20 7.20 6.98 6.97 6.96 6.96 6.96 6.95 6.94 6.94
5.12 5.10 5.09 5.08
4.49 4.46 4.35 4.32
2.95 2.88 2.63 2.59 2.55 2.50 DMSO-d6 2.44 2.35 2.04 1.99
Me
O
N
HN
NH
H
F
29
1.00 1.04 0.98 1.02 2.10 1.04 1.02 1.04 1.04 1.05 1.04 1.17 1.15 3.16 1.17 1.08
f1 (ppm)

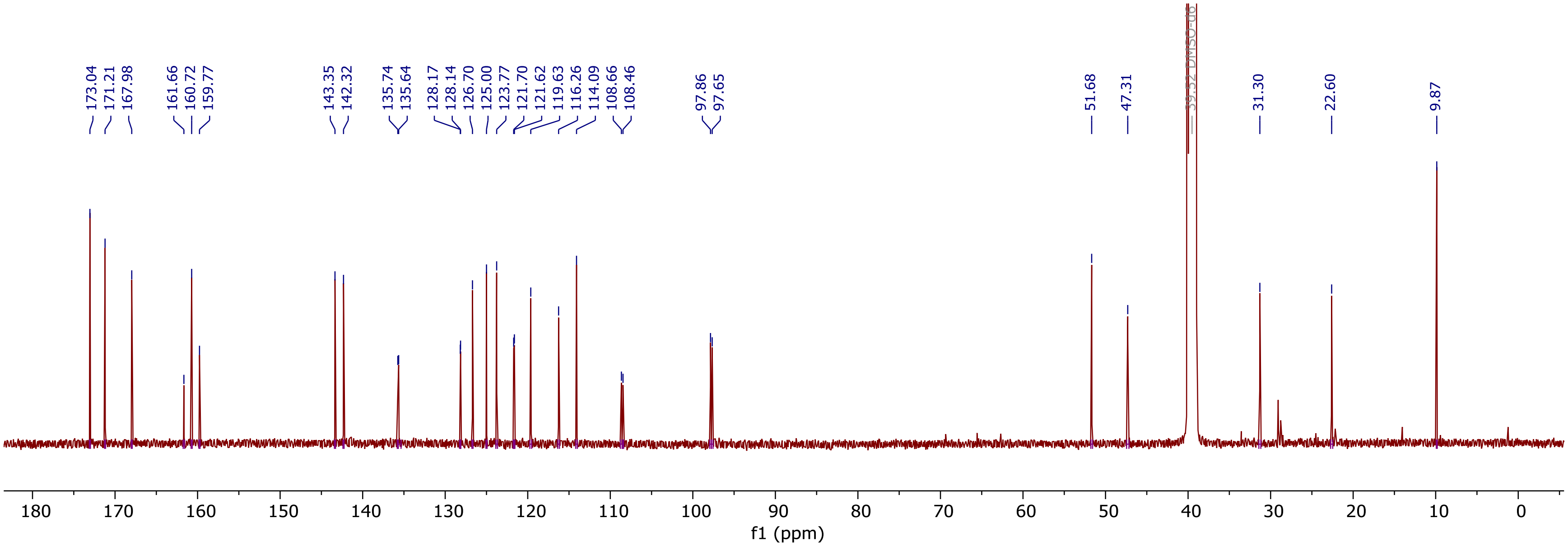
173.04 171.21 167.98 161.66 160.72 159.77 143.35 142.32 135.74 135.64 128.17 128.14 126.70 125.00 123.77 121.70 121.62 119.63 116.26 114.09 108.66 108.46 97.86 97.65
51.68 47.31 39.52 DMSO-d6 31.30 22.60 9.87
f1 (ppm)

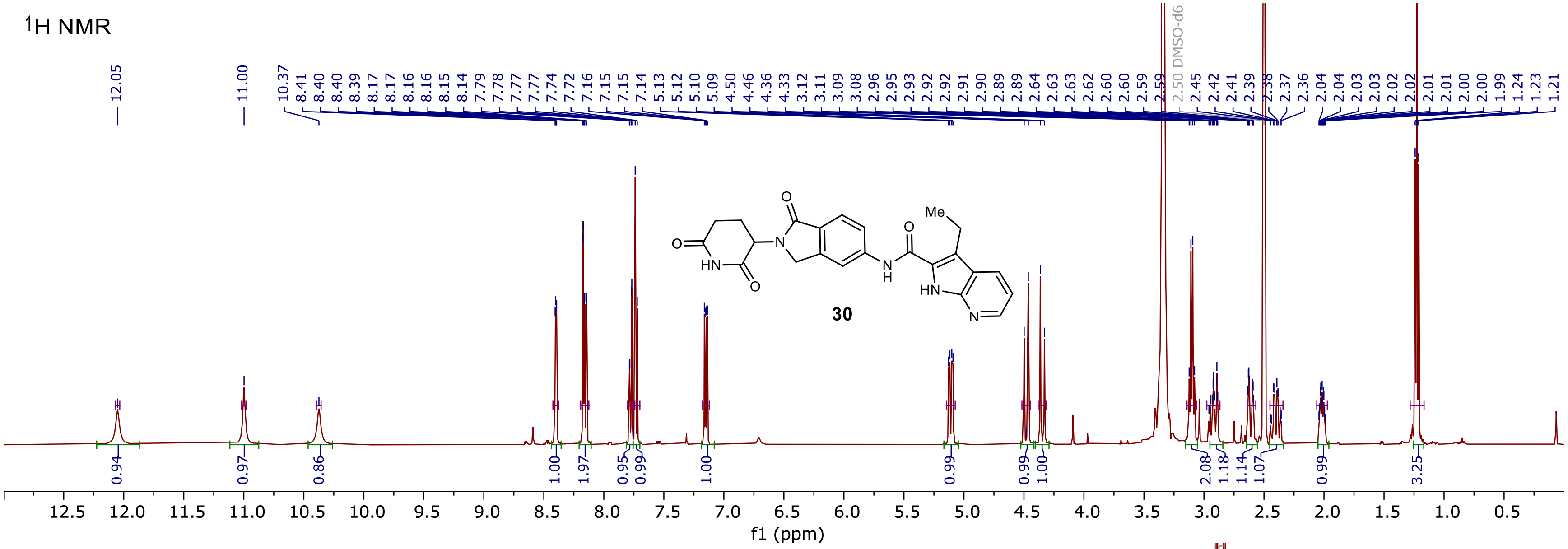

1H NMR
12.05
11.00
10.37 8.41 8.40 8.40 8.39 8.17 8.17 8.16 8.16 8.15 8.14 7.79 7.78 7.77 7.77 7.74 7.72 7.16 7.15 7.15 7.14 5.13 5.12 5.10 5.09 4.50 4.46 4.36 4.33 3.12 3.11 3.09 3.08 2.96 2.95 2.93 2.92 2.92 2.91 2.90 2.89 2.89 2.64 2.63 2.63 2.62 2.60 2.60 2.59 2.59
2.50 DMSO-d6
2.45 2.42 2.41 2.39 2.38 2.37 2.36 2.04 2.04 2.03 2.03 2.02 2.02 2.01 2.01 2.00 2.00 1.99 1.24 1.23 1.21
0.94 0.97 0.86 1.00 1.97 0.95 0.99 1.00 0.99 0.99 1.00 2.08 1.18 1.14 1.07 0.99 3.25
O
HN
O
N
O
N
H
O
Me
HN
N
30
12.5 12.0 11.5 11.0 10.5 10.0 9.5 9.0 8.5 8.0 7.5 7.0 6.5 6.0 5.5 5.0 4.5 4.0 3.5 3.0 2.5 2.0 1.5 1.0 0.5
f1 (ppm)

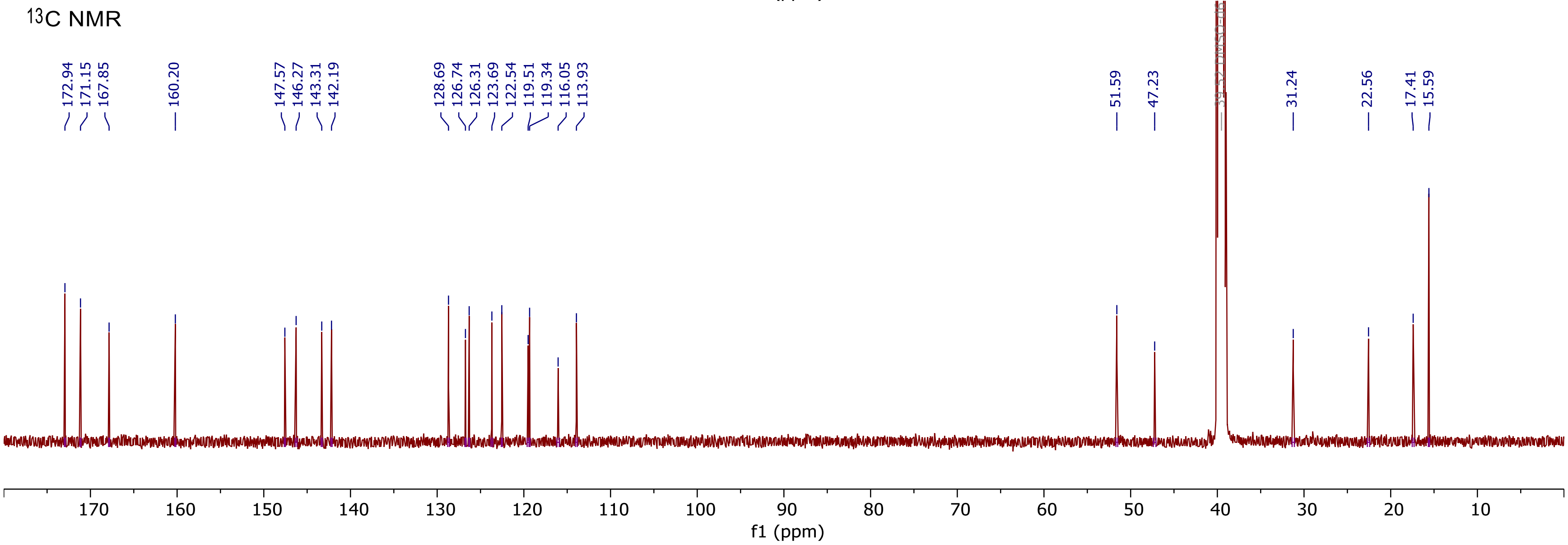

13C NMR
172.94 171.15 167.85
160.20
147.57 146.27 143.31 142.19
128.69 126.74 126.31 123.69 122.54 119.51 119.34 116.05 113.93
51.59
47.23
31.24
22.56
17.41 15.59
170 160 150 140 130 120 110 100 90 80 70 60 50 40 30 20 10
f1 (ppm)

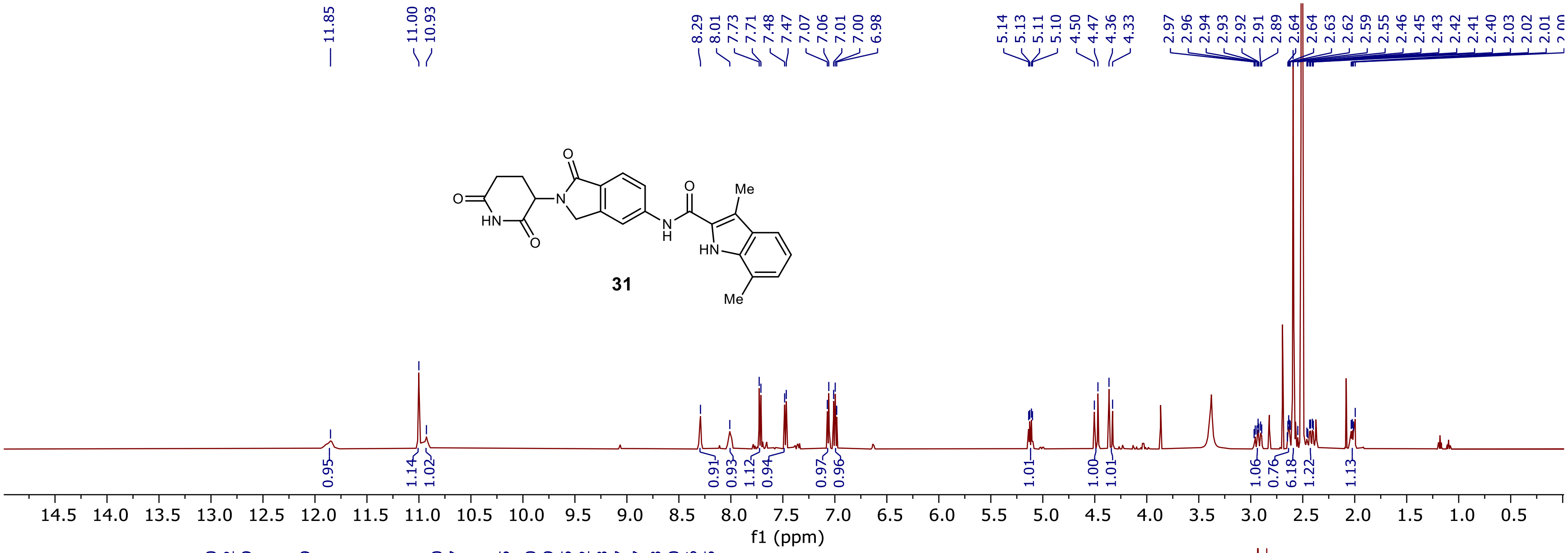
11.85
11.00
10.93
8.29
8.01
7.73
7.71
7.48
7.47
7.07
7.06
7.01
7.00
6.98
5.14
5.13
5.11
5.10
4.50
4.47
4.36
4.33
2.97
2.96
2.94
2.93
2.92
2.91
2.89
2.64
2.64
2.63
2.62
2.59
2.55
2.46
2.45
2.43
2.42
2.41
2.40
2.03
2.02
2.01
[illegible]
O
O
N
HN
O
N
H
O
Me
HN
Me
31
0.95
1.14
1.02
0.91
0.93
1.12
0.94
0.97
0.96
1.01
1.00
1.01
1.06
0.76
6.18
1.22
1.13
f1 (ppm)

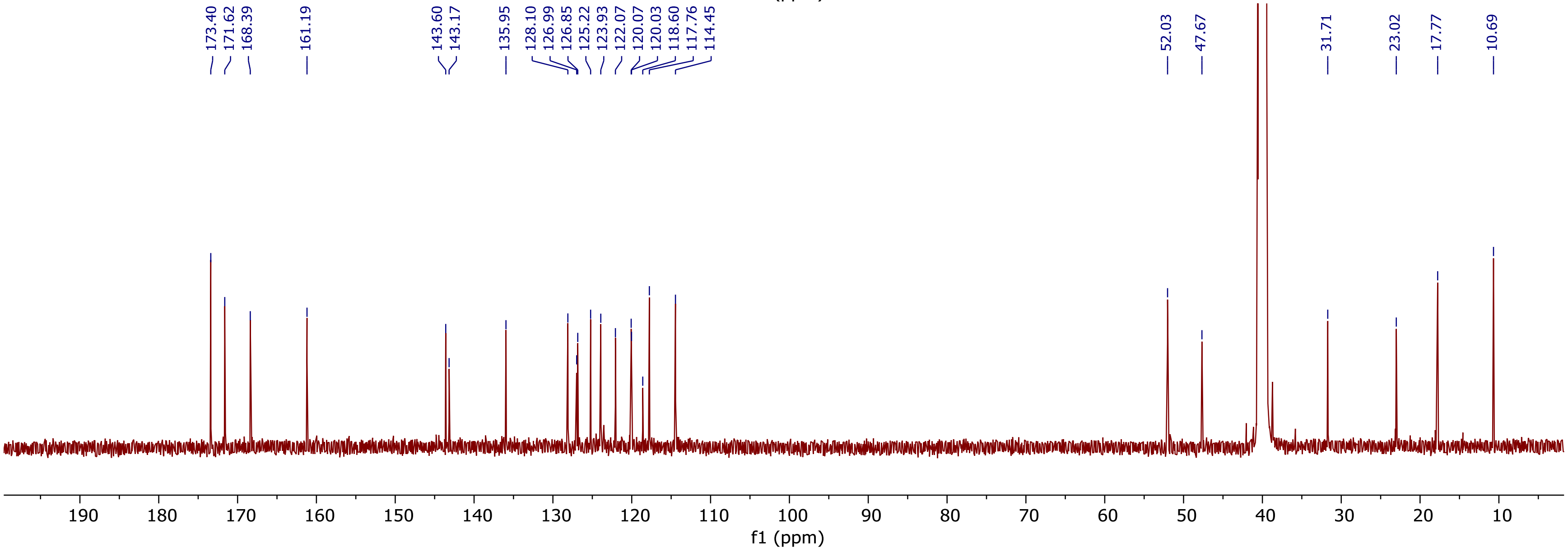
173.40
171.62
168.39
161.19
143.60
143.17
135.95
128.10
126.99
126.85
125.22
123.93
122.07
120.07
120.03
118.60
117.76
114.45
52.03
47.67
31.71
23.02
17.77
10.69
f1 (ppm)

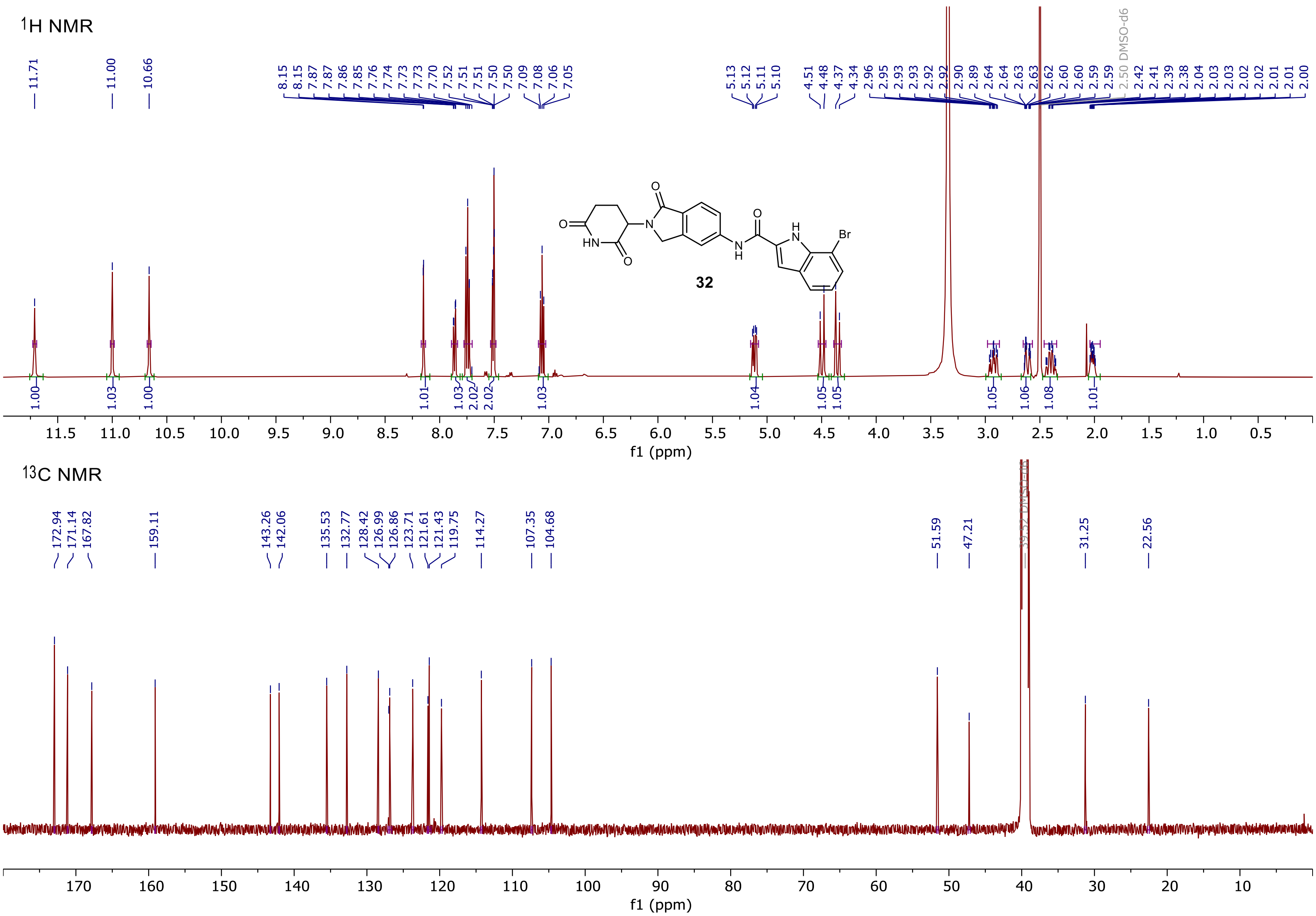
1H NMR
11.71
11.00
10.66
8.15 8.15 7.87 7.87 7.86 7.85 7.76 7.74 7.73 7.73 7.70 7.52 7.51 7.51 7.50 7.50 7.09 7.08 7.06 7.05
5.13 5.12 5.11 5.10
4.51 4.48 4.37 4.34
2.96 2.95 2.93 2.93 2.92 2.92 2.90 2.89 2.64 2.64 2.63 2.63 2.62 2.60 2.60 2.59 2.59
2.50 DMSO-d6
2.42 2.41 2.39 2.38 2.04 2.03 2.03 2.02 2.02 2.01 2.01 2.00
1.00 1.03 1.00 1.01 1.03 2.02 2.02 1.03 1.04 1.05 1.05 1.05 1.06 1.08 1.01
11.5 11.0 10.5 10.0 9.5 9.0 8.5 8.0 7.5 7.0 6.5 6.0 5.5 5.0 4.5 4.0 3.5 3.0 2.5 2.0 1.5 1.0 0.5
f1 (ppm)
O
HN
O
N
O
N
H
O
H
N
Br
32
13C NMR
172.94 171.14 167.82
159.11
143.26 142.06
135.53 132.77 128.42 126.99 126.86 123.71 121.61 121.43 119.75
114.27
107.35 104.68
51.59
47.21
39.52 DMSO-d6
31.25
22.56
170 160 150 140 130 120 110 100 90 80 70 60 50 40 30 20 10
f1 (ppm)

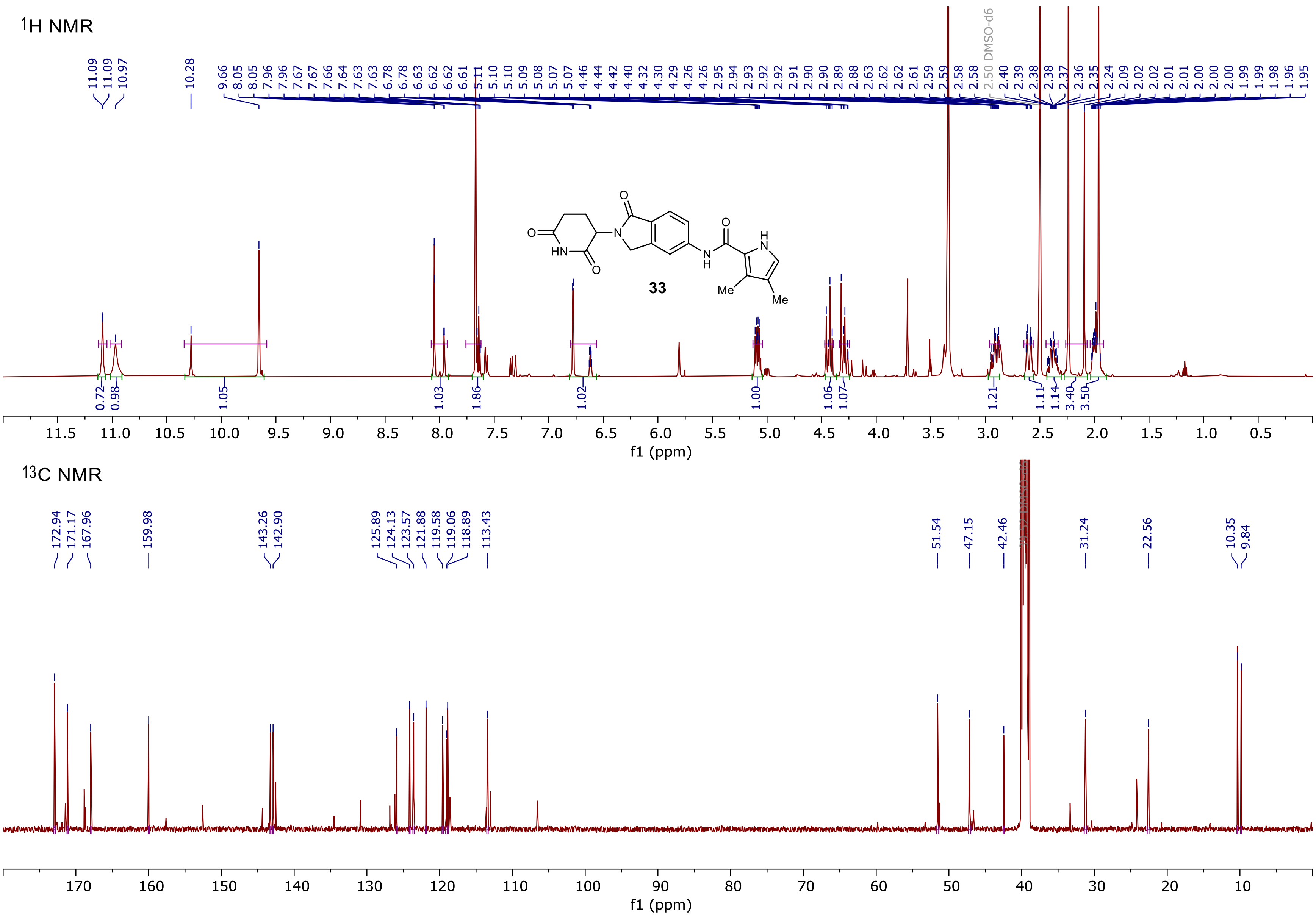
1H NMR
11.09
11.09
10.97
10.28
9.66
8.05
8.05
7.96
7.96
7.67
7.67
7.66
7.64
7.63
7.63
6.78
6.78
6.63
6.62
6.62
6.61
5.11
5.10
5.09
5.08
5.07
5.07
4.46
4.44
4.42
4.40
4.32
4.30
4.29
4.26
4.26
2.95
2.94
2.93
2.92
2.92
2.91
2.90
2.90
2.89
2.88
2.63
2.62
2.62
2.61
2.59
2.59
2.58
2.58
2.50 DMSO-d6
2.40
2.39
2.38
2.38
2.37
2.36
2.35
2.24
2.09
2.02
2.02
2.01
2.01
2.00
2.00
1.99
1.99
1.98
1.96
1.95
O
O
HN
N
O
N
H
H
N
33
Me
Me
0.72
0.98
1.05
1.03
1.86
1.02
1.00
1.06
1.07
1.21
1.11
1.14
3.40
3.50
11.5 11.0 10.5 10.0 9.5 9.0 8.5 8.0 7.5 7.0 6.5 6.0 5.5 5.0 4.5 4.0 3.5 3.0 2.5 2.0 1.5 1.0 0.5
f1 (ppm)
13C NMR
172.94
171.17
167.96
159.98
143.26
142.90
125.89
124.13
123.57
121.88
119.58
119.06
118.89
113.43
51.54
47.15
42.46
31.24
22.56
10.35
9.84
170 160 150 140 130 120 110 100 90 80 70 60 50 40 30 20 10
f1 (ppm)

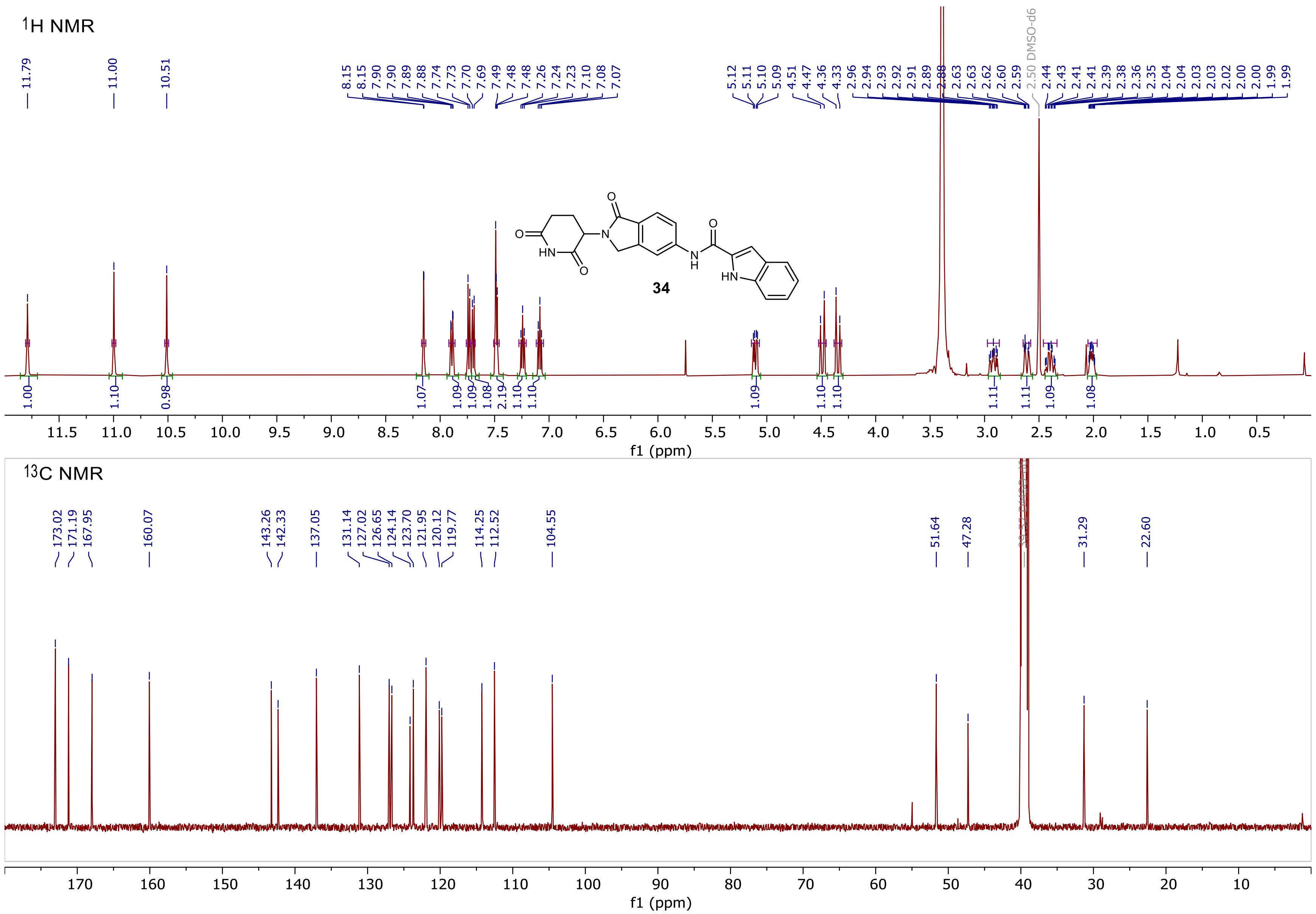
1H NMR
11.79
11.00
10.51
8.15 8.15 7.90 7.90 7.89 7.88 7.74 7.73 7.70 7.69 7.49 7.48 7.48 7.26 7.24 7.23 7.10 7.08 7.07
5.12 5.11 5.10 5.09 4.51 4.47 4.36 4.33 2.96 2.94 2.93 2.92 2.91 2.89 2.88 2.63 2.63 2.62 2.60 2.59
2.50 DMSO-d6
2.44 2.43 2.41 2.41 2.39 2.38 2.36 2.35 2.04 2.04 2.03 2.03 2.02 2.00 2.00 1.99 1.99
1.00 1.10 0.98 1.07 1.09 1.09 1.08 2.19 1.10 1.10 1.09 1.10 1.10 1.11 1.11 1.09 1.08
O
O
N
O
HN
O
N
H
HN
34
11.5 11.0 10.5 10.0 9.5 9.0 8.5 8.0 7.5 7.0 6.5 6.0 5.5 5.0 4.5 4.0 3.5 3.0 2.5 2.0 1.5 1.0 0.5
f1 (ppm)
13C NMR
173.02 171.19 167.95 160.07 143.26 142.33 137.05 131.14 127.02 126.65 124.14 123.70 121.95 120.12 119.77 114.25 112.52 104.55
51.64 47.28 31.29 22.60
170 160 150 140 130 120 110 100 90 80 70 60 50 40 30 20 10
f1 (ppm)

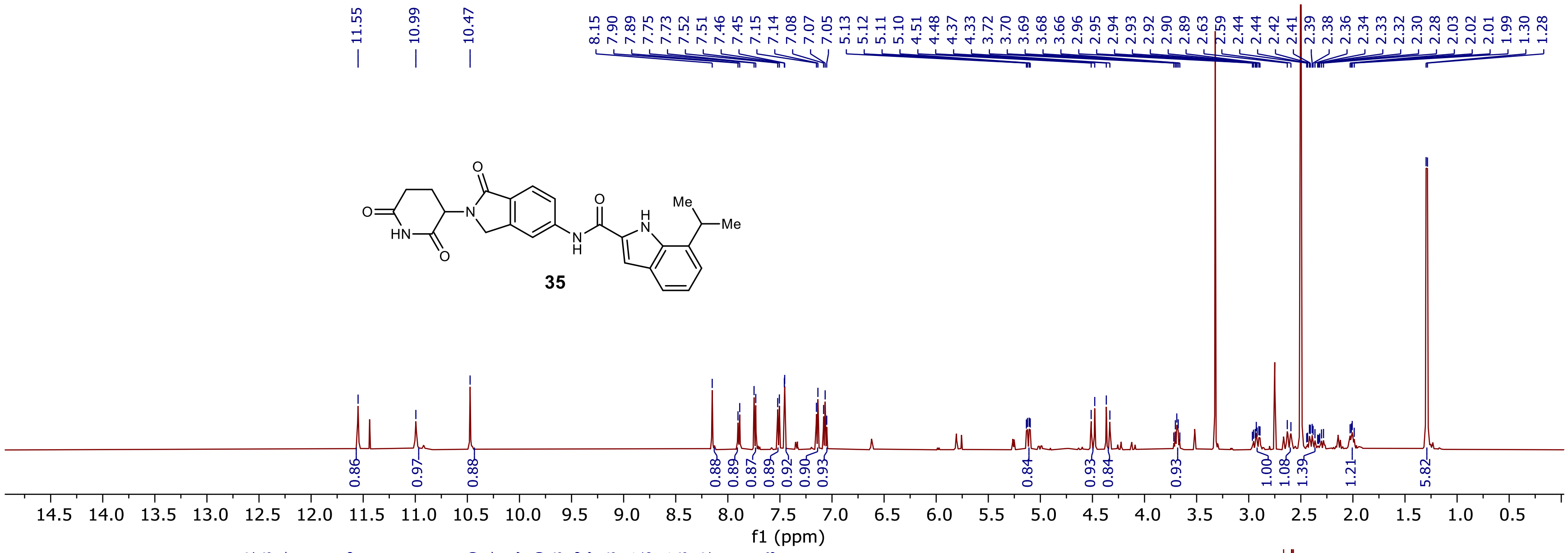
11.55
10.99
10.47
8.15 7.90 7.89 7.75 7.73 7.52 7.51 7.46 7.45 7.15 7.14 7.08 7.07 7.05 5.13 5.12 5.11 5.10 4.51 4.48 4.37 4.33 3.72 3.70 3.69 3.68 3.66 2.96 2.95 2.94 2.93 2.92 2.90 2.89 2.63 2.59 2.44 2.44 2.42 2.41 2.39 2.38 2.36 2.34 2.33 2.32 2.30 2.28 2.03 2.02 2.01 1.99 1.30 1.28
O
HN
N
O
O
H
N
H
N
Me
Me
35
0.86 0.97 0.88 0.88 0.89 0.87 0.89 0.92 0.90 0.93 0.84 0.93 0.84 0.93 1.00 1.08 1.39 1.21 5.82
14.5 14.0 13.5 13.0 12.5 12.0 11.5 11.0 10.5 10.0 9.5 9.0 8.5 8.0 7.5 7.0 6.5 6.0 5.5 5.0 4.5 4.0 3.5 3.0 2.5 2.0 1.5 1.0 0.5
f1 (ppm)

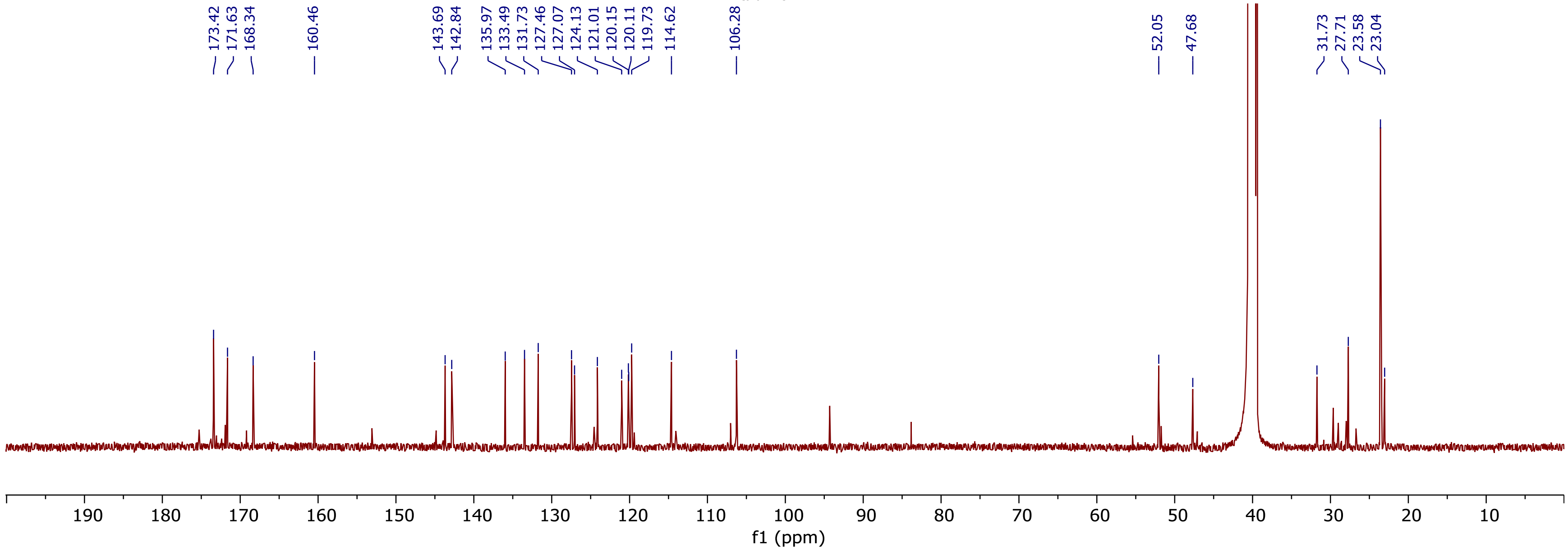
173.42 171.63 168.34
160.46
143.69 142.84
135.97 133.49 131.73 127.46 127.07 124.13 121.01 120.15 120.11 119.73
114.62
106.28
52.05
47.68
31.73 27.71 23.58 23.04
190 180 170 160 150 140 130 120 110 100 90 80 70 60 50 40 30 20 10
f1 (ppm)

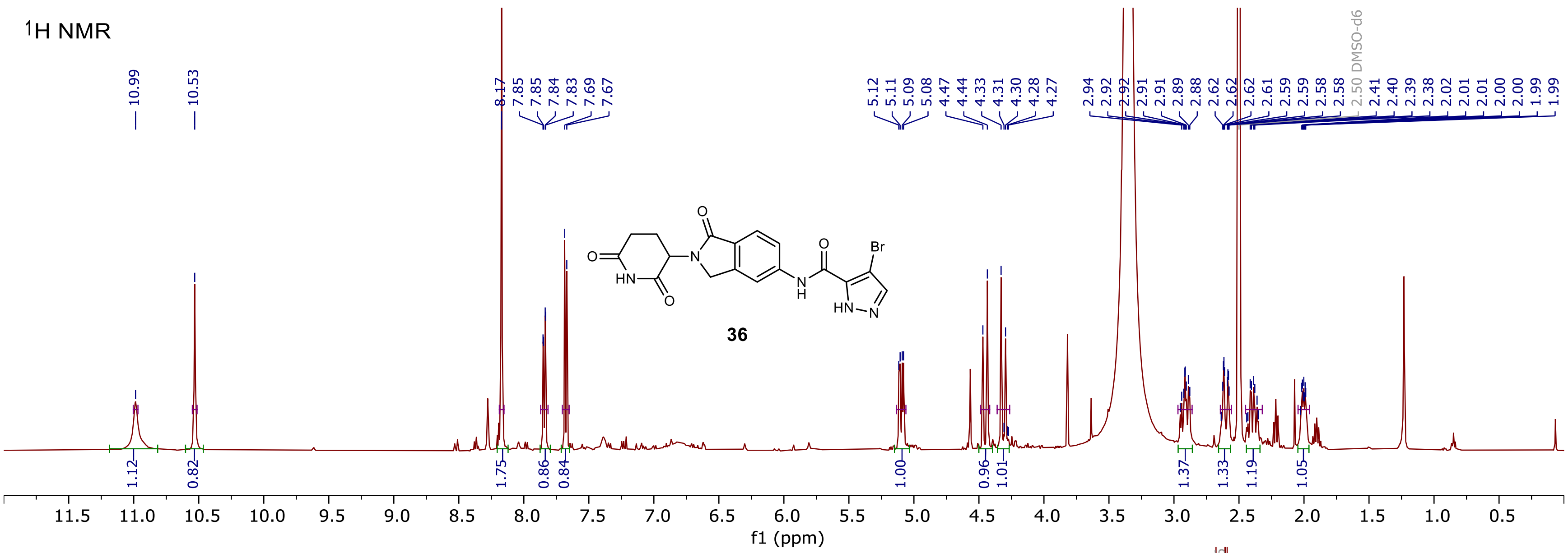
1H NMR
10.99
10.53
8.17
7.85
7.85
7.84
7.83
7.69
7.67
5.12
5.11
5.09
5.08
4.47
4.44
4.33
4.31
4.30
4.28
4.27
2.94
2.92
2.92
2.91
2.91
2.89
2.88
2.62
2.62
2.62
2.61
2.59
2.59
2.58
2.58
2.50 DMSO-d6
2.41
2.40
2.39
2.38
2.02
2.01
2.01
2.00
2.00
1.99
1.99
O
O
N
HN
O
N
H
O
Br
HN
N
36
1.12
0.82
1.75
0.86
0.84
1.00
0.96
1.01
1.37
1.33
1.19
1.05
11.5
11.0
10.5
10.0
9.5
9.0
8.5
8.0
7.5
7.0
6.5
6.0
5.5
5.0
4.5
4.0
3.5
3.0
2.5
2.0
1.5
1.0
0.5
f1 (ppm)

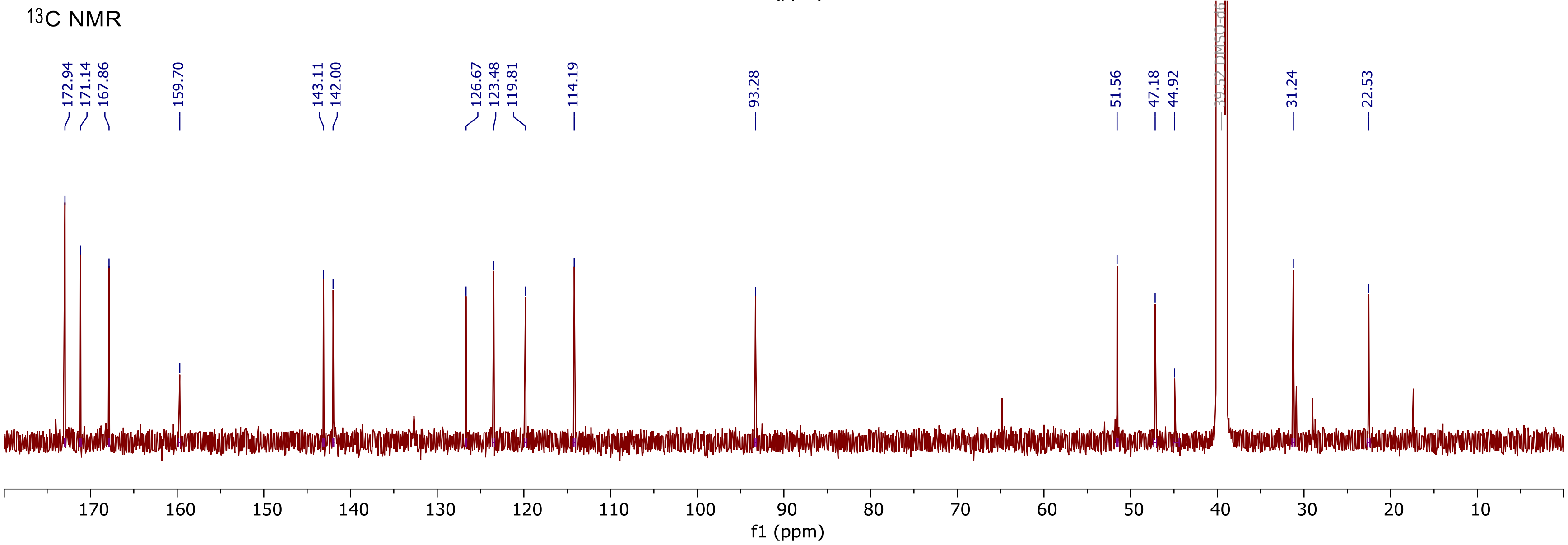
13C NMR
172.94
171.14
167.86
159.70
143.11
142.00
126.67
123.48
119.81
114.19
93.28
51.56
47.18
44.92
39.52 DMSO-d6
31.24
22.53
170
160
150
140
130
120
110
100
90
80
70
60
50
40
30
20
10
f1 (ppm)

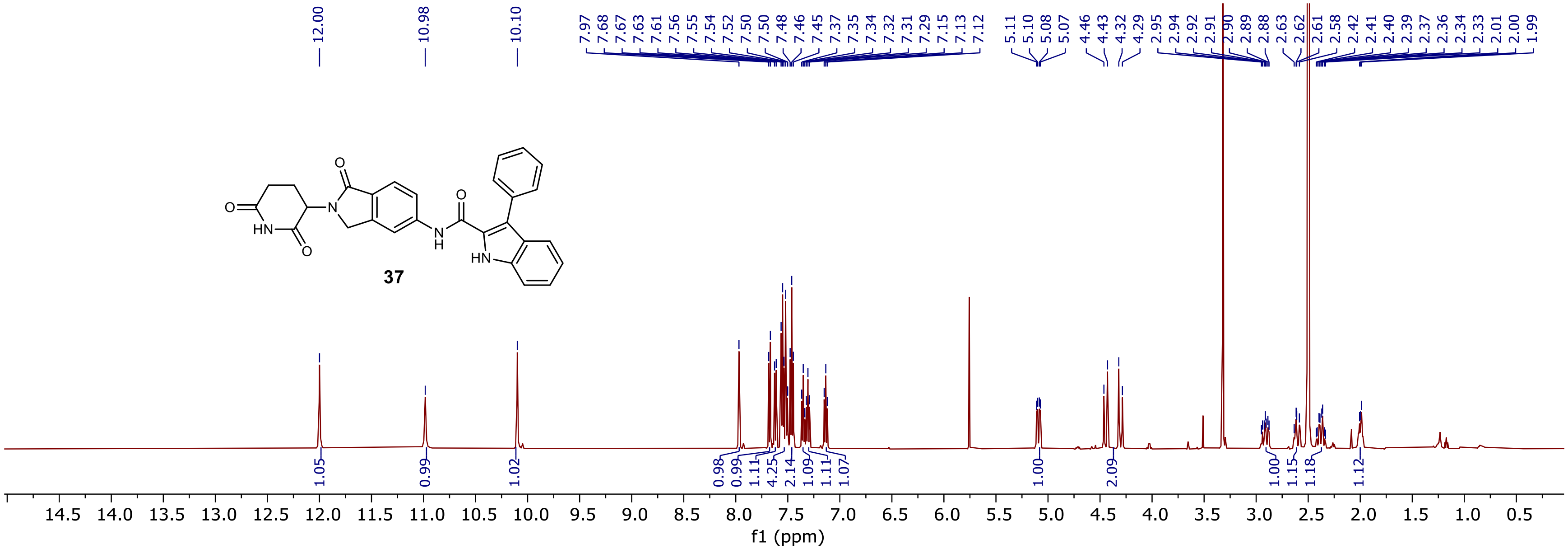
37
HN
N
H
O
12.00
10.98
10.10
7.97 7.68 7.67 7.63 7.61 7.56 7.55 7.54 7.52 7.50 7.50 7.48 7.46 7.45 7.37 7.35 7.34 7.32 7.31 7.29 7.15 7.13 7.12
5.11 5.10 5.08 5.07 4.46 4.43 4.32 4.29 2.95 2.94 2.92 2.91 2.90 2.89 2.88 2.63 2.62 2.61 2.58 2.42 2.41 2.40 2.39 2.37 2.36 2.34 2.33 2.01 2.00 1.99
1.05 0.99 1.02 0.98 0.99 1.11 4.25 2.14 1.09 1.11 1.07 1.00 2.09 1.00 1.15 1.18 1.12
14.5 14.0 13.5 13.0 12.5 12.0 11.5 11.0 10.5 10.0 9.5 9.0 8.5 8.0 7.5 7.0 6.5 6.0 5.5 5.0 4.5 4.0 3.5 3.0 2.5 2.0 1.5 1.0 0.5
f1 (ppm)

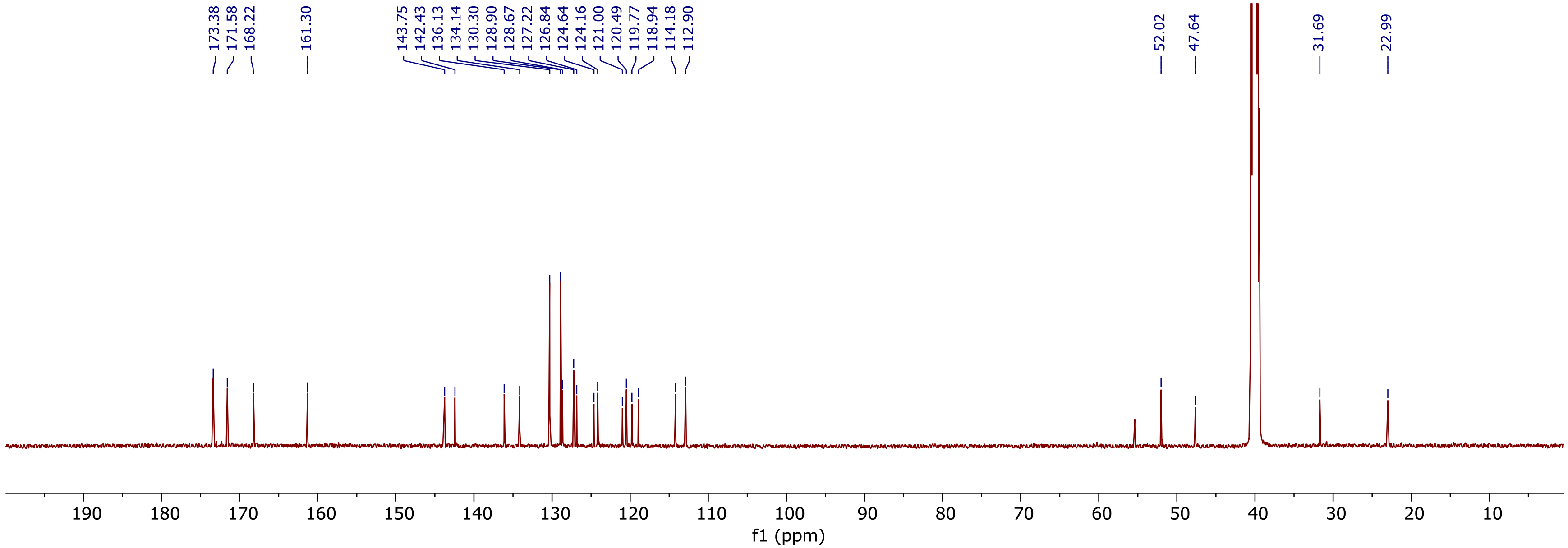
173.38 171.58 168.22 161.30
143.75 142.43 136.13 134.14 130.30 128.90 128.67 127.22 126.84 124.64 124.16 121.00 120.49 119.77 118.94 114.18 112.90
52.02 47.64 31.69 22.99
190 180 170 160 150 140 130 120 110 100 90 80 70 60 50 40 30 20 10
f1 (ppm)

^{1}H NMR

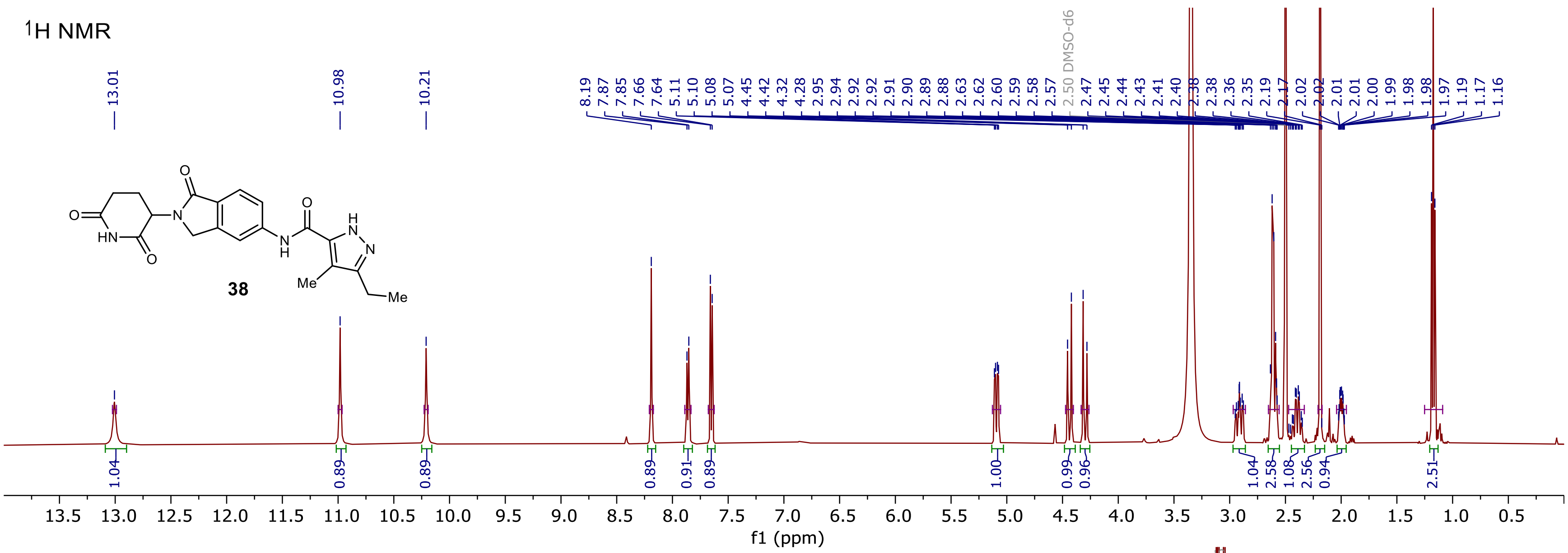

38

^{13}C NMR

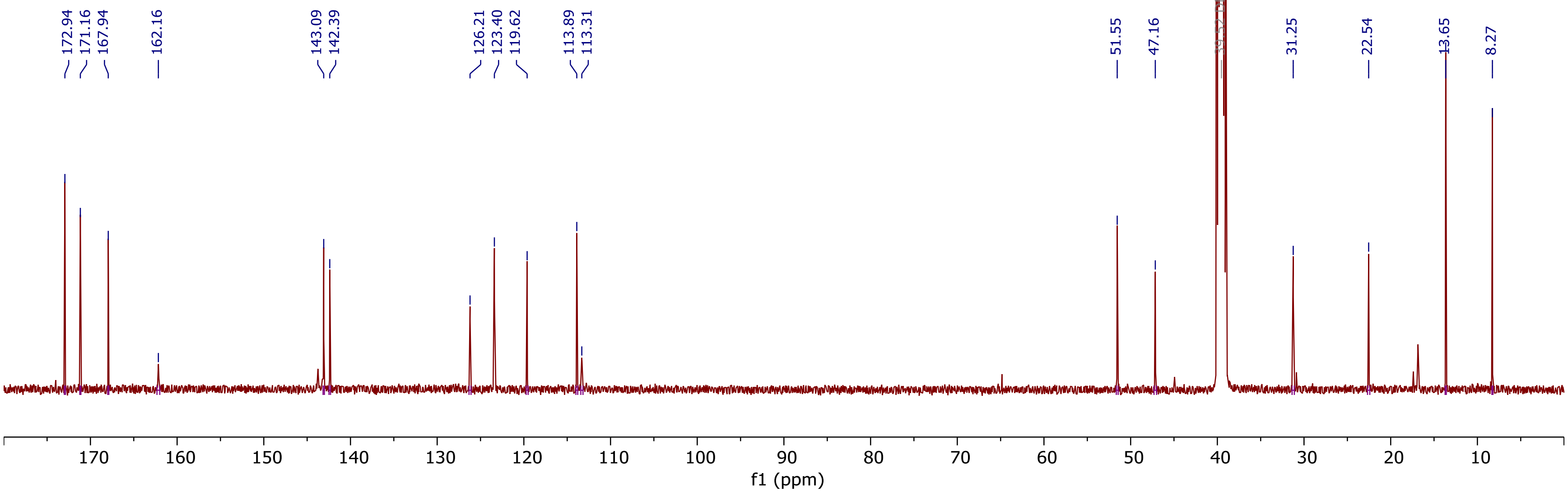

^{1}H NMR

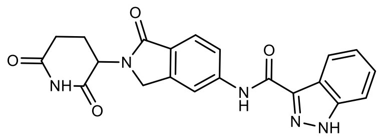

39

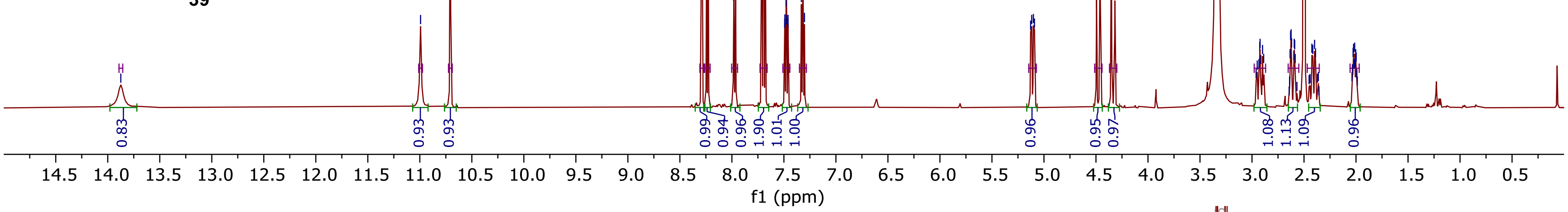

^{13}C NMR

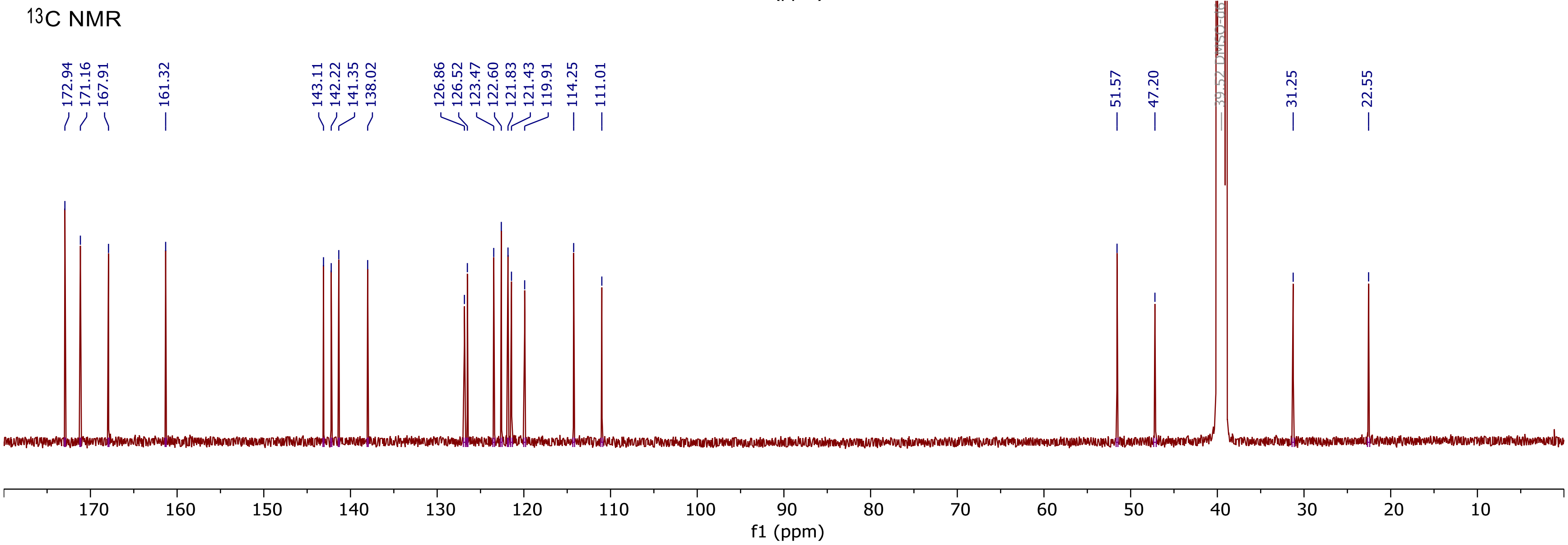

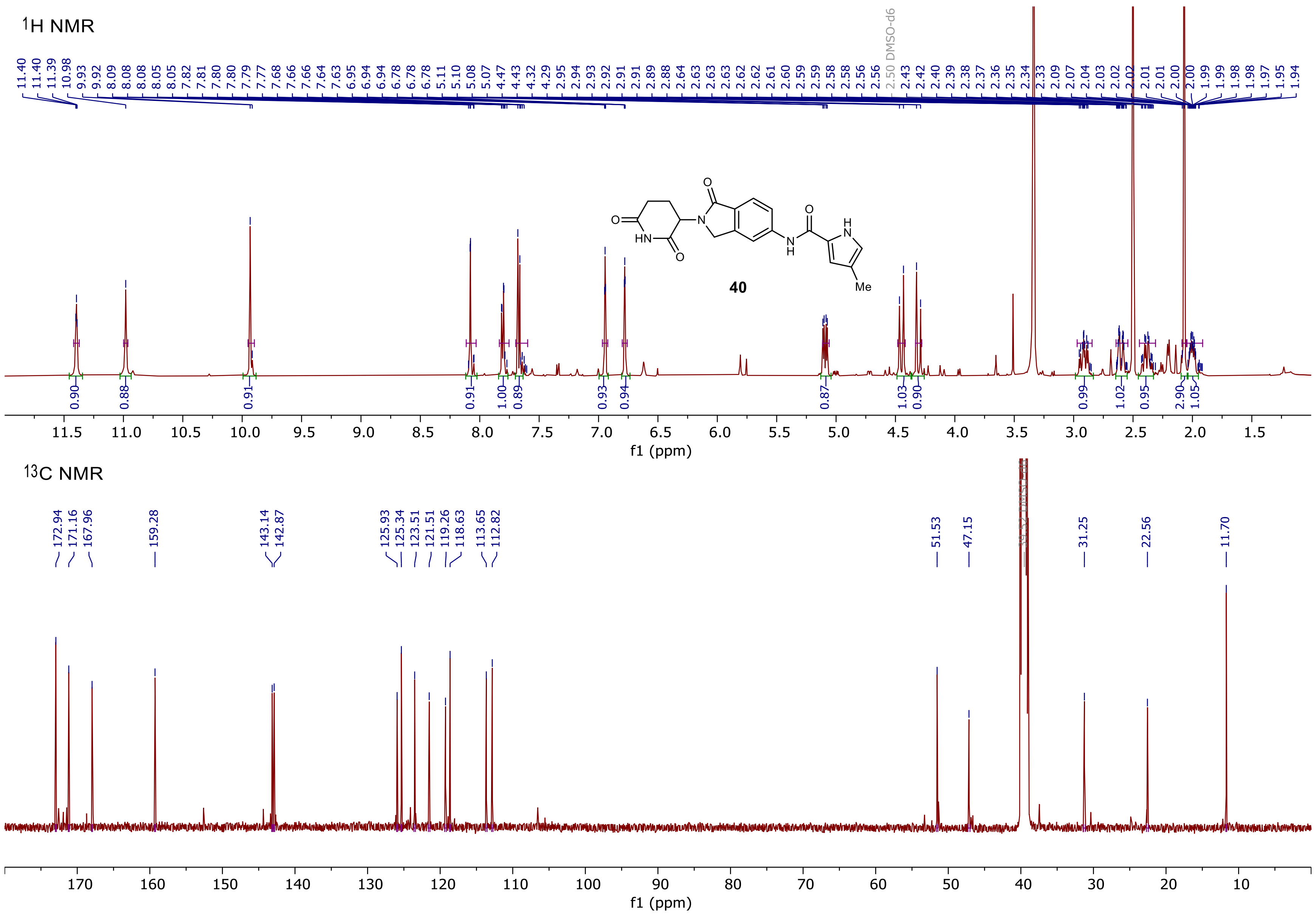
1H NMR
40
Me
f1 (ppm)
13C NMR
172.94
171.16
167.96
159.28
143.14
142.87
125.93
125.34
123.51
121.51
119.26
118.63
113.65
112.82
51.53
47.15
31.25
22.56
11.70
f1 (ppm)

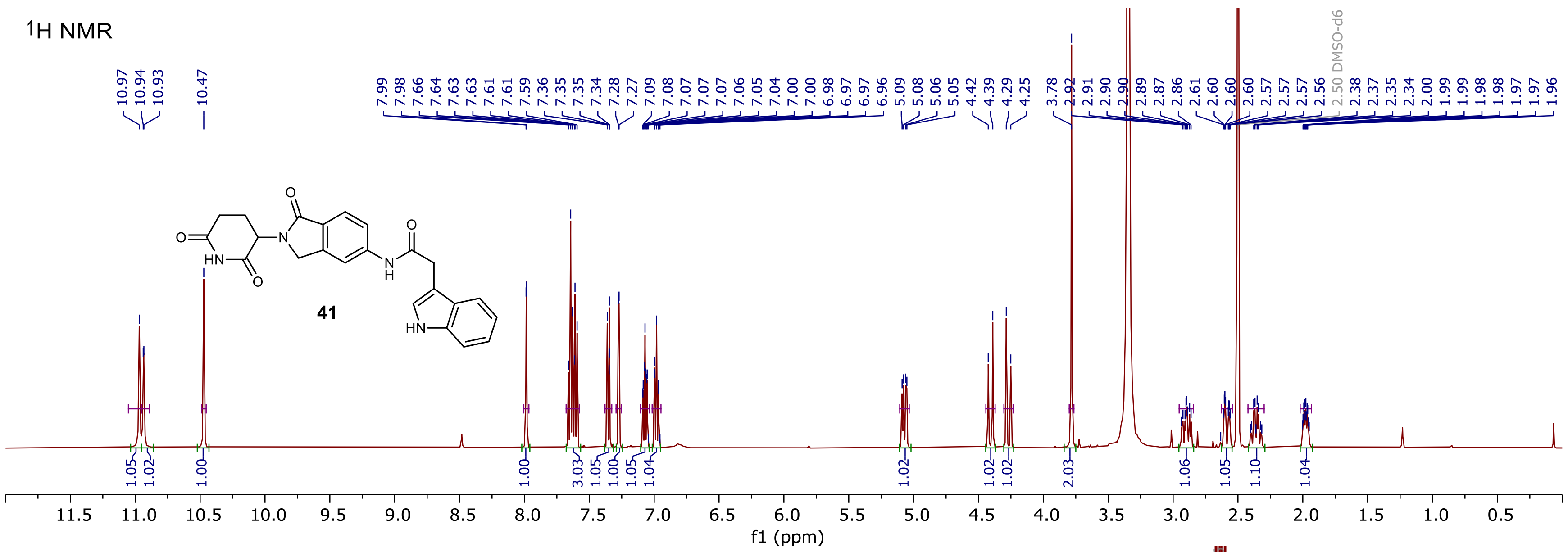

1H NMR
41
2.50 DMSO-d6
f1 (ppm)

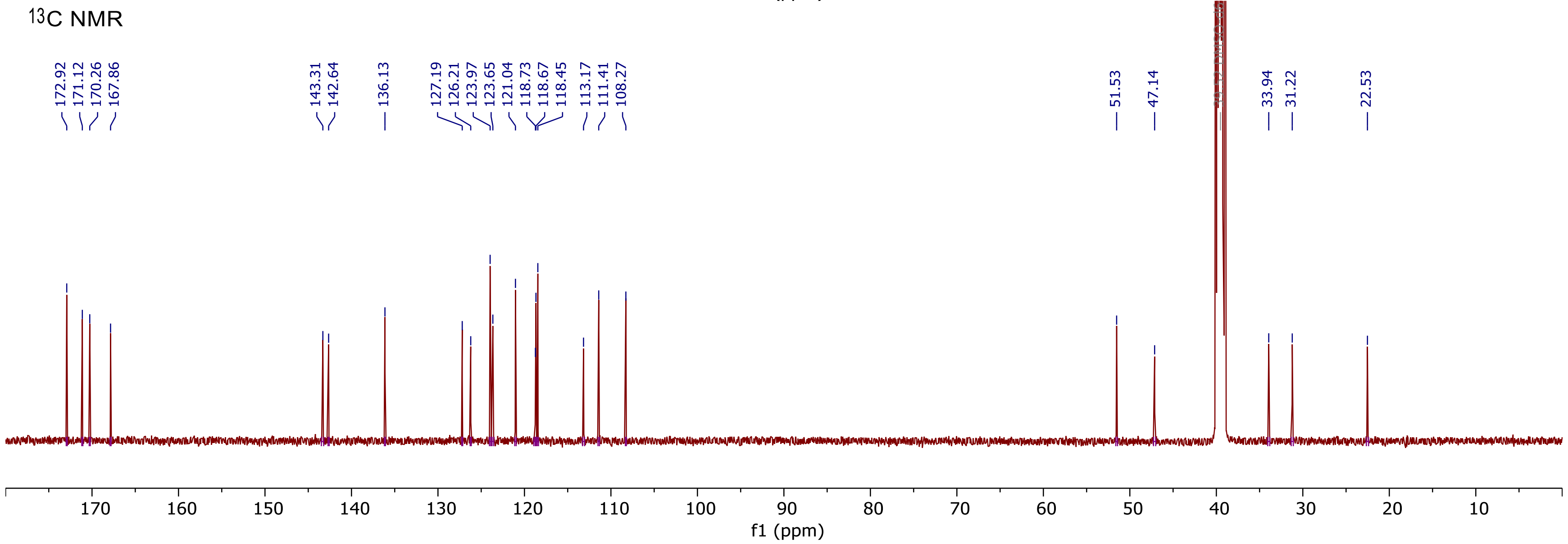

13C NMR
f1 (ppm)

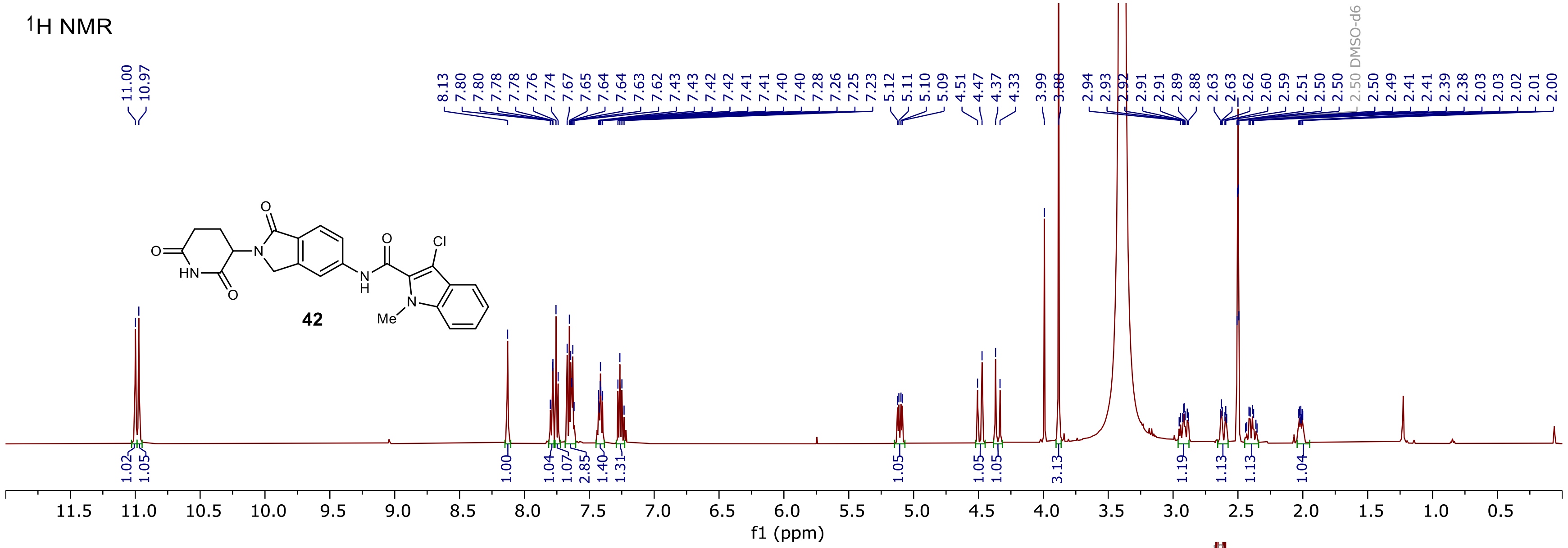

1H NMR
11.00
10.97
8.13
7.80
7.80
7.78
7.78
7.76
7.74
7.67
7.65
7.64
7.64
7.63
7.62
7.43
7.43
7.42
7.41
7.41
7.40
7.40
7.28
7.26
7.25
7.23
5.12
5.11
5.10
5.09
4.51
4.47
4.37
4.33
3.99
3.88
2.94
2.93
2.92
2.91
2.91
2.89
2.88
2.63
2.63
2.62
2.60
2.59
2.51
2.50
2.50
2.50 DMSO-d6
2.50
2.49
2.41
2.41
2.39
2.38
2.03
2.03
2.02
2.01
2.00
O
O
N
HN
O
N
H
O
Cl
N
Me
42
1.02
1.05
1.00
1.04
1.07
2.85
1.40
1.31
1.05
1.05
1.05
3.13
1.19
1.13
1.13
1.04
11.5 11.0 10.5 10.0 9.5 9.0 8.5 8.0 7.5 7.0 6.5 6.0 5.5 5.0 4.5 4.0 3.5 3.0 2.5 2.0 1.5 1.0 0.5
f1 (ppm)

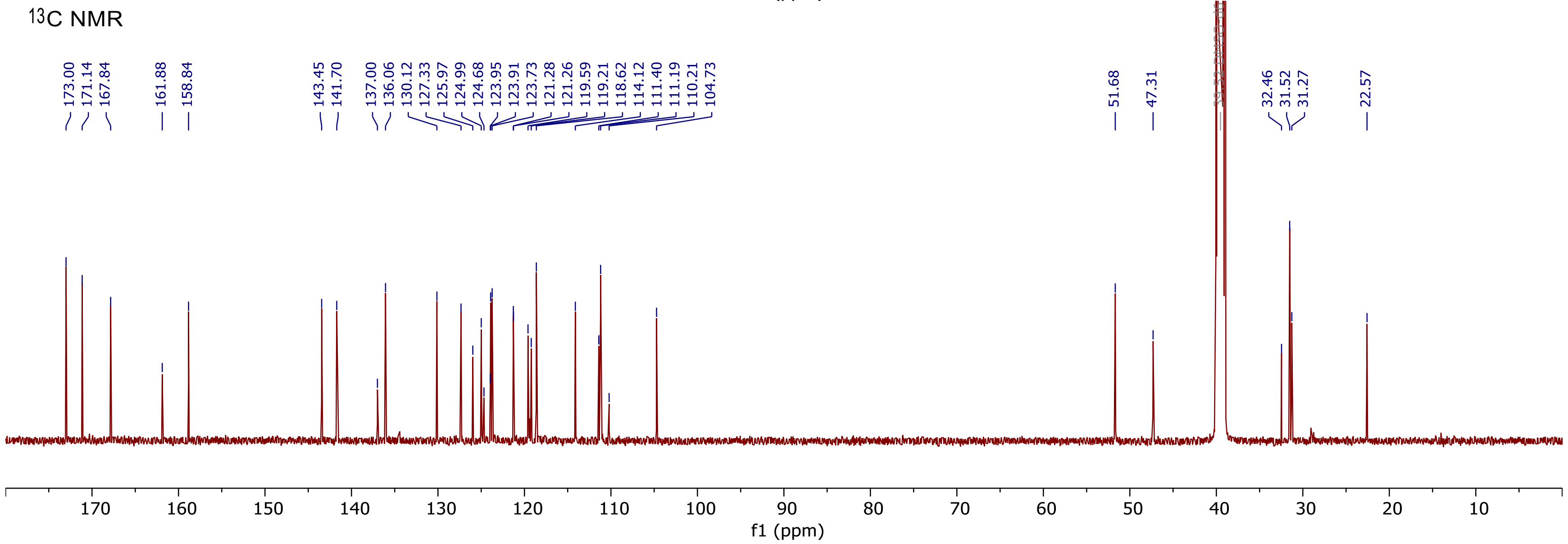

13C NMR
173.00
171.14
167.84
161.88
158.84
143.45
141.70
137.00
136.06
130.12
127.33
125.97
124.99
124.68
123.95
123.91
123.73
121.28
121.26
119.59
119.21
118.62
114.12
111.40
111.19
110.21
104.73
51.68
47.31
32.46
31.52
31.27
22.57
170 160 150 140 130 120 110 100 90 80 70 60 50 40 30 20 10
f1 (ppm)
